# Supplementary material for: Dearomatization of aromatic asmic isocyanides to complex cyclohexadienes
Source: Nat Commun. 2022 Oct 28;13:6444. doi: 10.1038/s41467-022-33807-7 (PMC9616822; doi:10.1038/s41467-022-33807-7)
Supplement: Supplementary file 1 — Supplementary Information [file 41467_2022_33807_MOESM1_ESM.pdf]

Supplementary Information

Dearomatization of Aromatic Asmic Isocyanides to Complex Cyclohexadienes

Bilal Altundas,[1] Embarek Alwedi,[2] Zhihui Song,[3] Achyut Ranjan Gogoi,[4] Ryan Dykstra,[3] Osvaldo Gutierrez,*[4] Fraser F. Fleming*[1]

[1] Department of Chemistry, Drexel University, 3400 Chestnut St., Philadelphia PA 19104

[2] 90 E. Scott Ave, Merck Inc., Rahway, NJ 07065

[3] Department of Chemistry and Biochemistry, University of Maryland, 8051 Reagents Drive, College Park, MD 20742.

[4] University of Texas A&M, Department of Chemistry, University College Station, Texas TX 77843-3255.

Compound	Procedure	^{1}H- and ^{13}C-NMR
**Supplementary Methods**	S10	
General method for the synthesis of formamides	S10	
General formamide dehydration method	S10	
General isocyanide alkylation procedure with LDA	S10	
General isocyanide alkylation procedure with LDA for alkylations with dihaloalkanes	S11	
General isocyanide alkylation procedure with BuLi-LDA	S11	
General isocyanide alkylation procedure with BuLi-LDA for alkylations with dihaloalkanes	S12	

General isocyanide alkylation procedure with LiHMDS S12

General isocyanide alkylation procedure with LiHMDS for alkylations with dihaloalkanes S13

General method for the dearomatization of Asmic derivatives with HMTETA S13

General method for the dearomatization of Asmic derivatives with TMEDA-LiCl S13

Syntheses

N-(((2-Fluorophenyl)thio)methyl)formamide (**i**)

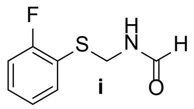

S14 S45

N-(((5-Chloro-2-methoxyphenyl)thio)methyl)formamide (**ii**)

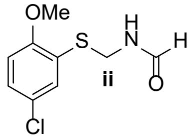

S15 S46

N-(((2,6-Dimethoxyphenyl)thio)methyl)formamide (**iii**)

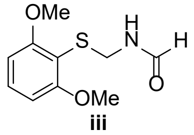

S15 S47

N-(((2-(Trifluoromethoxy)phenyl)thio)methyl)formamide (**iv**)

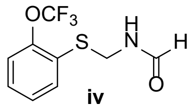

S16 S48

N-(((2-methoxynaphthalen-1-yl)thio)methyl)formamide (**v**)

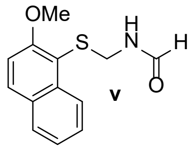

S16 S49

(2-Fluorophenyl)(isocyanomethyl)sulfane (**vi**)

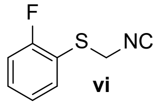

S17 S50

(5-Chloro-2-methoxyphenyl)(isocyanomethyl)sulfane (**vii**)

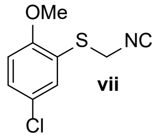

S18 S51

(2,6-Dimethoxyphenyl)(isocyanomethyl)sulfane (**viii**)

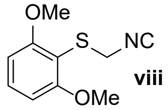

S18 S52

(Isocyanomethyl)(2-(trifluoromethoxy)phenyl)sulfane (**ix**)

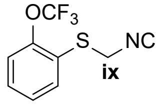

S18 S53

(Isocyanomethyl)(2-methoxynaphthalen-1-yl)sulfane (**x**)

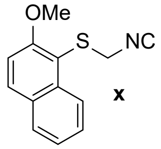

S19 S54

(1-Isocyanocyclopentyl)(2-methoxyphenyl)sulfane (**19a**)

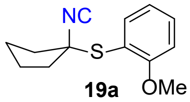

S19 S55

(2-Isocyanopropan-2-yl)(2-methoxyphenyl)sulfane (**19b**)

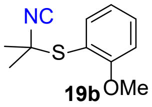

S20 S56

(4-Isocyanoheptan-4-yl)(2-methoxyphenyl)sulfane (**19c**)

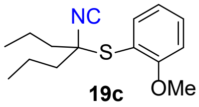

S20 S57

(7-Isocyanotridecan-7-yl)(2-methoxyphenyl)sulfane (**19d**)

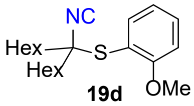

S21 S58

(1-Isocyanocyclohexyl)(2-methoxyphenyl)sulfane (**19e**) S21 S59

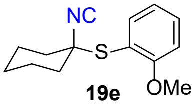

19e

(1-Isocyanocycloheptyl)(2-methoxyphenyl)sulfane (**19f**) S21 S60

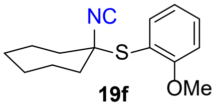

19f

1-Benzyl-4-(1-isocyanocyclopentyl)-2-methoxycyclohexa-2,5-diene-1-carbonitrile (**22a**) S22 S61

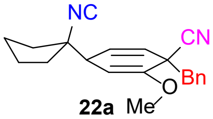

22a

1-(4-Bromobenzyl)-4-(1-isocyanocyclopentyl)-2-methoxycyclohexa-2,5-diene-1-carbonitrile (**22b**) S23 S62

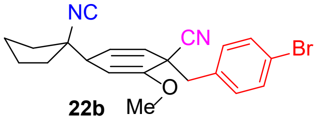

22b

1-Allyl-4-(1-isocyanocyclopentyl)-2-methoxycyclohexa-2,5-diene-1-carbonitrile (**22c**) S23 S63

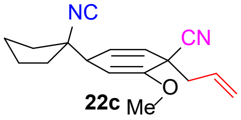

22c

4-(1-Isocyanocyclopentyl)-2-methoxy-1-propylcyclohexa-2,5-diene-1-carbonitrile (**22d**) S24 S64

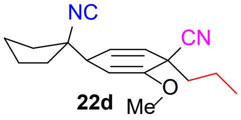

22d

4-Benzyl-1'-isocyano-3-methoxy-[1,1'-bi(cyclohexane)]-2,5-diene-4-carbonitrile (**22e**) S25 S65

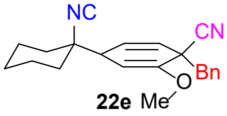

22e

1-Benzyl-4-(1-isocyanocycloheptyl)-2-methoxycyclohexa-2,5-diene-1-carbonitrile (**22f**) S25 S66

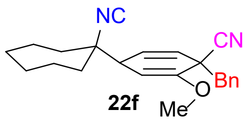

22f

1-Benzyl-4-(2-isocyanopropan-2-yl)-2-methoxycyclohexa-2,5-diene-1-carbonitrile (**22g**) S26 S67

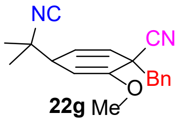

22g

1-Benzyl-4-(4-isocyanoheptan-4-yl)-2-methoxycyclohexa-2,5-diene-1-carbonitrile (**22h**) S26 S68

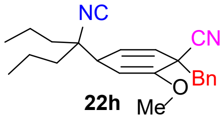

22h

1-Benzyl-4-(7-isocyano-6,8-dioxotridecan-7-yl)-2-methoxycyclohexa-2,5-diene-1-carbonitrile (**22i**) S27 S69

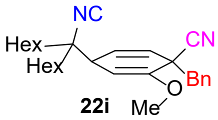

22i

4-(1-Isocyanocyclopentyl)-2-methoxycyclohexa-1,5-diene-1-carbo-nitrile (**23a**) S28 S70

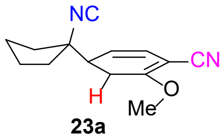

23a

4-(1-Isocyanocyclopentyl)-2-methoxycyclohexa-1,5-diene-1-carbo-nitrile-3-*d* (**23b**) S28 S71

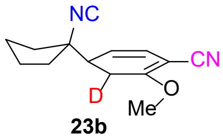

23b

4-(7-isocyano-6,8-dioxotridecan-7-yl)-2-methoxycyclohexa-1,5-diene-1-carbonitrile (**23c**) S29 S72

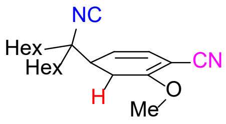

23c

(2-Fluorophenyl)(1-isocyanocyclopentyl)sulfane (**24a**) S29 S73

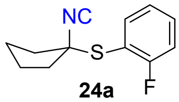

(2-Fluorophenyl)(1-isocyanocyclohexyl)sulfane (**24b**) S30 S74

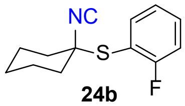

(2-Fluorophenyl)(1-isocyanocycloheptyl)sulfane (**24c**) S30 S75

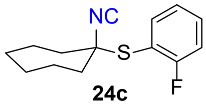

(2-Fluorophenyl)(2-isocyanopropan-2-yl)sulfane (**24d**) S31 S76

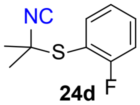

(2-Fluorophenyl)(4-isocyanoheptan-4-yl)sulfane (**24e**) S31 S77

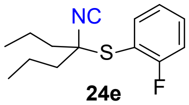

(2-Fluorophenyl)(2-isocyano-1,3-diphenylpropan-2-yl)sulfane (**24f**) S32 S78

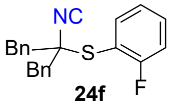

(5-Chloro-2-methoxyphenyl)(1-isocyanocyclopentyl)sulfane (**24g**) S32 S79

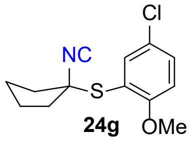

(2,6-Dimethoxyphenyl)(1-isocyanocyclopentyl)sulfane (**24h**) S33 S80

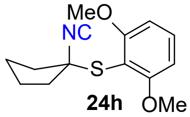

(1-Isocyanocyclopentyl)(2-(trifluoromethoxy)phenyl)sulfane (**24i**) S33 S81

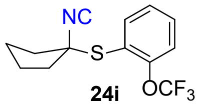

24i

(1-isocyanocyclopentyl)(2-methoxynaphthalen-1-yl)sulfane (**24j**)

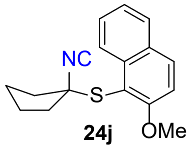

24j

S34 S82

(2-Isocyanopropan-2-yl)(2-methoxynaphthalen-1-yl)sulfane (**24k**)

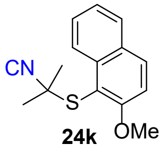

24k

S34 S83

1-Benzyl-2-fluoro-4-(1-isocyanocyclopentyl)cyclohexa-2,5-diene-1-carbonitrile (**25a**)

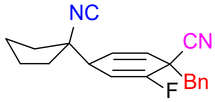

25a

S35 S84

1-Allyl-2-fluoro-4-(1-isocyanocyclopentyl)cyclohexa-2,5-diene-1-carbonitrile (**25b**)

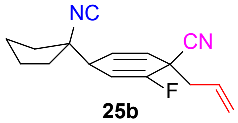

25b

S36 S85

4-Benzyl-3-fluoro-1'-isocyano-[1,1'-bi(cyclohexane)]-2,5-diene-4-carbonitrile (**25c**)

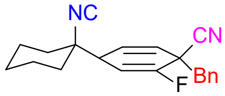

25c

S36 S86

1-Benzyl-2-fluoro-4-(1-isocyanocycloheptyl)cyclohexa-2,5-diene-1-carbonitrile (**25d**)

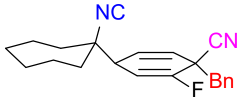

25d

S37 S87

1-Benzyl-2-fluoro-4-(4-isocyanoheptan-4-yl)cyclohexa-2,5-diene-1-carbonitrile (**25e**) S37 S88

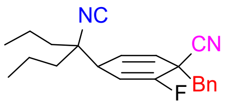

25e

1-Benzyl-2-fluoro-4-(2-isocyanopropan-2-yl)cyclohexa-2,5-diene-1-carbonitrile (**25f**) S38 S89

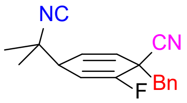

25f

1-Benzyl-2-fluoro-4-(2-isocyano-1,3-diphenylpropan-2-yl)cyclohexa-2,5-diene-1-carbonitrile (**25g**) S39 S90

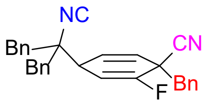

25g

1-Benzyl-5-chloro-4-(1-isocyanocyclopentyl)-2-methoxycyclohexa-2,5-diene-1-carbonitrile (**25h**) S39 S91

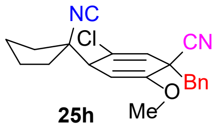

25h

1-Benzyl-4-(1-isocyanocyclopentyl)-2,6-dimethoxycyclohexa-2,5-diene-1-carbonitrile (**25i**) S40 S92

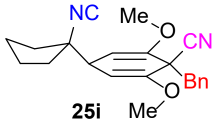

25i

((1-Benzyl-4-(1-isocyanocyclopentyl)-2-methoxy-1,4-dihydro-naphthalen-1-yl)-l3-methylene)-l2-azane (**25j**) S40 S93

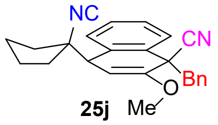

25j

((4-(2-Isocyanopropan-2-yl)-2-methoxy-3,4-dihydronaphthalen-1-yl)-l3-methylene)-l2-azane (**25k**) S41 S94

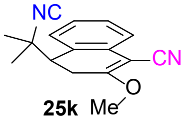

25k

1-Benzyl-4-(4-isocyanoheptan-4-yl)-2-(trifluoromethoxy)-cyclohexa-2,5-diene-1-carbonitrile (**25l**)

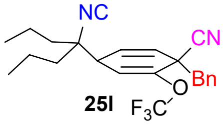

25l S42 S95

4,4-Diphenyl-2,2-dipropyl-3,4-dihydro-2H-pyrrole (**27**)

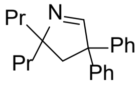

27 S42 S96

2-Methyl-2-phenyl-1-thia-4-azaspiro[4.4]non-3-ene (**28**)

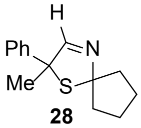

28 S43 S97

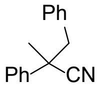

30 S43 S98

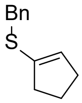

31 S43 S99

DFT Calculations Data S100

Computational Details S100

Supplementary Discussion S101

Energetics with Different Methods and Conformational Search S101

Supplementary References S105

Supplementary Methods

General method for the synthesis of formamides. Paraformaldehyde (4 eq), formamide (7.5 eq) and formic acid (5 eq) were sequentially added to neat thiol (1 eq) and then the flask was heated to 90-100 °C in an oil bath for 3-21 h. The reaction was monitored by TLC. Upon completion, the mixture was cooled and then diluted with cold water and EtOAc. The mixture was extracted with EtOAc (x 4), the organic extracts were then combined, washed with brine (x 3), dried (Na_2SO_4), and concentrated. The resulting crude formamide was dry loaded onto Celite and purified by automated flash column chromatography on silica gel using a Reveleris purification system with 50-100% EtOAc:Hexanes as the mobile phase.

General formamide dehydration method. Neat *i*-Pr_2NEt (5 equiv) was added to a 0 °C, CH_2Cl_2 (0.8 M) solution of the formamide (1 equiv). Neat $POCl_3$ (2.5 equiv) was added dropwise maintaining the temperature between 0-5 °C for 2-3 h followed by careful addition of saturated aqueous $NaHCO_3$ solution until a pH of 8-9. CH_2Cl_2 was added until an approximately equal volume as the aqueous phase, the phases were separated, and then the aqueous phase was extracted with CH_2Cl_2 (x 3). The combined organic extracts were concentrated, redissolved in EtOAc, and then washed with brine (x 3), dried (Na_2SO_4), filtered, and concentrated to afford pure isocyanide. Unless otherwise stated, the isocyanide was used without further purification. Purifications of impure isocyanides were performed by dry loading the isocyanide on Celite followed by purification via automated flash column chromatography on silica gel using a Reveleris purification system with 0-10% EtOAc:Hexanes as the mobile phase.

General isocyanide alkylation procedure with LDA. A THF (0.2 M) solution of the isocyanide (1 equiv) was added, dropwise, to a -78 °C THF solution of LDA (1.1 equiv), generated from diisopropylamine (1.1 equiv) and BuLi (1.1 equiv). After 15 min, neat electrophile (1.05 - 1.1 equiv) was rapidly added and, after 10 min, the cooling bath was removed, and the reaction

allowed to warm to rt. After 1-2 h, the reaction mixture was cooled to -78 °C and then a THF solution of LDA (1.1 equiv) was added dropwise. After 15 min, neat electrophile (1.05 – 1.1 equiv) was rapidly added. After 15 min the cooling bath was removed, the reaction allowed to warm to rt, and stirred 12-24 h. Upon completion, as judged by TLC analysis, saturated, aqueous NH_4Cl was added, the phases were separated, and the aqueous phase was extracted with EtOAc (3 x 25 mL). The combined organic extract was dried (Na_2SO_4) and concentrated. The crude isocyanide was dry loaded onto Celite and purified by automated flash column chromatography on silica gel using a Reveleris purification system with 0-10% EtOAc:Hexanes as the mobile phase.

General isocyanide alkylation procedure with LDA for alkylations with dihaloalkanes. For alkylations employing dihaloalkane electrophiles, a slightly modified procedure was used. After addition of LDA, the electrophile (1.05 – 1.1 equiv) was rapidly added and, after 10 min, the cooling bath was removed and then the reaction was allowed to warm to rt. After 1-2 h, the reaction mixture was cooled to -78 °C and then a THF solution of LDA (1.1 equiv) was added dropwise. After 15 min the cooling bath was removed, the reaction was allowed to warm to rt over 12-24 h.

General isocyanide alkylation procedure with BuLi-LDA. A hexanes solution of BuLi (1.05 - 1.1 equiv) was added dropwise to a -78 °C, THF (0.2 M) solution of the isocyanide (1 equiv). After 15 min, neat electrophile (1.05 - 1.1 equiv) was rapidly added. After 10 min, the cooling bath was removed, and then the reaction was allowed to warm to rt. After 1-2 h, the reaction mixture was cooled to -78 °C and then a THF solution of LDA (1.1 equiv) was added dropwise. After 15 min, neat electrophile (1.05 - 1.1 equiv) was rapidly added. After 15 min the cooling bath was removed and then the reaction was allowed to warm to rt over 12-24 h. Upon completion of the alkylation, as judged by TLC analysis, saturated aqueous NH_4Cl was added, the phases were separated, and then the aqueous phase was extracted with EtOAc (x3). The combined organic

extract was dried (Na_2SO_4), concentrated, and then dry loaded on Celite and purified by automated flash column chromatography on silica gel using a Reveleris purification system with 0-10% EtOAc:Hexanes as the mobile phase.

General isocyanide alkylation procedure with BuLi-LDA for alkylations with dihaloalkanes. Following the general BuLi-LDA procedure with the modification that after addition of BuLi, the electrophile (1.05 – 1.1 equiv) was rapidly added. After 15 min, the cooling bath was removed, and then the reaction allowed to warm to rt. After 1-2 h, the reaction mixture was cooled to -78 °C and then a THF solution of LDA (1.1 equiv) was added dropwise. After 15 min, the cooling bath was removed and then the reaction was allowed to warm to rt over 12-24 h.

General isocyanide alkylation procedure with LiHMDS. A THF solution of LiHMDS (1.1 equiv) was added dropwise to a -78 °C, THF (0.2 M) solution of the isocyanide (1 equiv). After 15 min, neat electrophile (1.05 - 1.1 equiv) was rapidly added. After 10 min, the cooling bath was removed, and then the reaction was allowed to warm to rt. After 1-2 h, the reaction mixture was cooled to -78 °C and then LiHMDS (1.1 equiv) was added dropwise. After 15 min, neat electrophile (1.05 - 1.1 equiv) was rapidly added. After 15 min the cooling bath was removed, the reaction allowed to warm to rt, and stirred 12-24 h. Upon completion of the alkylation, as judged by TLC analysis, saturated aqueous NH_4Cl was added, the phases were separated, and then the aqueous phase was extracted with EtOAc (x3). The combined organic extract was dried (Na_2SO_4), concentrated, and then dry loaded on Celite and purified by automated flash column chromatography on silica gel using a Reveleris purification system with 0-10% EtOAc:Hexanes as the mobile phase.

General isocyanide alkylation procedure with LiHMDS for alkylations with dihaloalkanes. Following the general LiHMDS procedure with the modification that after addition of LiHMDS,

the electrophile (1.05 – 1.1 equiv) was rapidly added. After 15 min, the cooling bath was removed, and then the reaction was allowed to warm to rt. After 1-2 h, the reaction mixture was cooled to -78 °C and then LiHMDS (1.1 equiv) was added dropwise. After 15 min, the cooling bath was removed and then the reaction allowed to warm to rt over 12-24 h.

General method for the dearomatization of Asmic derivatives with HMTETA. Neat HMTETA (1.1 equiv) was added to a -78 °C, THF (0.1 M) solution of the isocyanide (1 equiv). After 5 min, a hexanes solution of BuLi (1.1 equiv) was added dropwise over ~20-30 seconds followed, after 10 min, by rapid addition of the neat electrophile (1.1 equiv). After 1 h, the cooling bath was removed, and the reaction was then allowed to warm to rt over 4 h. Upon completion, as judged by TLC analysis, saturated, aqueous NH_4Cl was added, the phases were separated, and the aqueous phase was extracted with CH_2Cl_2 (x 3). The combined organic extract was dried (Na_2SO_4), concentrated, and then dry loaded on Celite and purified by automated flash column chromatography on silica gel using a Reveleris purification system with 0-15% EtOAc:Hexanes as the mobile phase.

General method for the dearomatization of Asmic derivatives with TMEDA-LiCl. A THF solution of LiCl (0.786 M, 2 equiv) and neat TMEDA (1.1 equiv) were added sequentially to a -78 °C, THF solution of the isocyanide (1 equiv). After 5 min, a hexanes solution of BuLi (1.1 equiv) was added dropwise over ~10 seconds, followed, after 10 - 90 min, by rapid addition of the neat electrophile (1.1 equiv). After 1 h, the cooling bath was removed, and then the reaction was warmed to rt and stirred for 16 h. Upon completion, as judged by TLC analysis, saturated, aqueous, NH_4Cl was added, the phases were separated, and the aqueous phase was extracted with CH_2Cl_2 (x 3). The combined organic phase was dried (Na_2SO_4), concentrated, and then dry loaded onto

Celite and purified by automated flash column chromatography on silica gel using a Reveleris purification system with 0-15% EtOAc:Hexanes as the mobile phase.

Syntheses

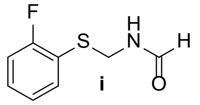

***N*-(((2-Fluorophenyl)thio)methyl)formamide (i):** 2-fluorobenzenethiol (3 g, 23.4 mmol) was submitted to the general formamide synthesis procedure with formamide (7.00 mL, 175.6 mmol), paraformaldehyde (2.81 g, 93.6 mmol), and formic acid (4.42 mL, 117 mmol) for 21 h at 90 °C to afford, after purification on silica gel (0 -50% EtOAc:Hexanes) 2.48 g (57 %) of **i** as a clear oil: IR (ATR): 3277, 3052, 2872, 1663 cm^{-1}; The ^1H NMR (500 MHz, $CDCl_3$) spectrum was a 4:1 mixture of two rotamers. For the major rotamer: δ 8.13 (d, *J* = 1.5 Hz, 1H), 7.48 (td, *J* = 7.7, 1.7 Hz, 1H), 7.31 (dddd, *J* = 8.2, 7.7, 5.2, 1.7 Hz, 1H), 7.12 (td, *J* = 7.7, 1.3 Hz, 1H), 7.11 (ddd, *J* = 9.5, 8.2, 1.3 Hz, 1H), 6.04 (br s, 1H), 4.70 (d, *J* = 6.4 Hz, 2H). For the minor rotamer: δ 7.78 (d, *J* = 11.6 Hz, 1H), 7.47 (ddd, *J* = 9.7, 7.5, 1.9 Hz, 1H), 7.38 (dddd, *J* = 8.3, 7.5, 5.2, 1.9 Hz, 1H), 7.16 (td, *J* = 4.1, 1.3 Hz, 1H), 7.14 (t, *J* = 2.1, 1.3 Hz, 1H), 5.94 (br s, 1H), 4.58 (d, *J* = 7.1 Hz, 2H). ^{13}C {^1H} (101 MHz, $CDCl_3$) for the major rotamer: δ 162.3 (d, *J* = 245.4 Hz), 160.8, 134.7 (d, *J* = 1.3 Hz), 130.4 (d, *J* = 8.1 Hz), 125.0 (d, *J* = 3.8 Hz), 120.4 (d, *J* = 18.0 Hz), 116.2 (d, *J* = 23.0 Hz), 41.9 (q, *J* = 3.4 Hz); for the minor rotamer: δ 163.7, 162.9 (d, *J* = 246.6 Hz), 136.7 (br. d, *J* = 0.8 Hz), 131.6 (d, *J* = 8.1 Hz), 125.2 (d, *J* = 3.8 Hz), 118.5 (d, *J* = 18.0 Hz), 116.5 (d, *J* = 23.0 Hz), 47.0 (q, *J* = 3.4 Hz); HRMS (+APCI) m/z [$M+H^+$] Calcd. for C_8H_9ONFS, 186.0383; found, 186.0384.

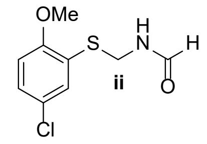

***N*-(((5-Chloro-2-methoxyphenyl)thio)methyl)formamide (ii):** The general formamide synthesis procedure was performed with 5-chloro-2-methoxybenzenethiol[1] (1 g, 5.73 mmol), formamide (1.7 mL, 42.9 mmol), paraformaldehyde (688 mg, 22.3 mmol), and formic acid (1.08 mL, 28.6 mmol) for 5 h at 100 °C to afford, after purification on silica gel using a Reveleris purification system (40 g cartridge, 50-100% EtOAc:Hexanes) 1.01 g (76 %) of **ii** as a white solid: mp: 79-81 °C; IR (ATR): 3274, 3035, 2938, 2867, 2840, 1661 cm^{-1}. The ^{1}H NMR (400 MHz, $CDCl_3$) spectrum was a 4:1 mixture of two rotamers. For the major rotamer: δ 8.13 (d, *J* = 1.4 Hz, 1H), 7.38 (d, *J* = 2.7 Hz, 1H), 7.23 (dd, *J* = 8.8, 2.7 Hz, 1H), 6.82 (d, *J* = 8.8 Hz, 1H), 6.09 (s, 1H), 4.70 (d, *J* = 6.4 Hz, 2H), 3.90 (s, 3H). For the minor rotamer: δ 7.83 (d, *J* = 11.7 Hz, 1H), 7.40 (d, *J* = 2.7 Hz, 1H), 7.30 (dd, *J* = 8.8, 2.7 Hz, 1H), 6.85 (d, *J* = 8.8 Hz, 1H), 6.00 (s, 1H), 4.59 (d, *J* = 7.0 Hz, 2H), 3.90 (s, 3H). ^{13}C {^{1}H} NMR (101 MHz, $CDCl_3$) for the major rotamer: δ 160.7, 157.3, 132.6, 129.1, 126.0, 123.1, 112.3, 56.5, 40.7; for the minor rotamer: δ 163.7, 158.2, 135.1, 130.4, 126.1, 121.2, 112.5, 56.5, 46.2; HRMS (+APCI) m/z [$M+H^+$] Calcd. for $C_9H_{11}O_2NClS$, 232.0193; found, 232.0194.

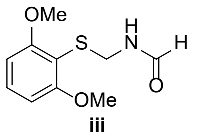

***N*-(((2,6-Dimethoxyphenyl)thio)methyl)formamide (iii):** The general formamide synthesis procedure was followed with 2,6-dimethoxybenzenethiol[2] (1 g, 5.87 mmol), formamide (1.77 mL, 44.4 mmol), paraformaldehyde (716 mg, 23.9 mmol), and formic acid (1.11 mL, 29.5 mmol) for 10 h at 100 °C to afford, after purification on silica gel using a Reveleris purification system (40 g cartridge, 50-100% EtOAc:Hexanes) 734 mg (55 %) of **iii** as a clear oil whose ^{1}H NMR was identical to that of material previously isolated[3]**:** IR (ATR): 3311, 3006, 2941, 2838, 1662 cm^{-1}; The ^{1}H NMR (400 MHz, $CDCl_3$) spectrum was a 2:1 mixture of two rotamers. For the major rotamer: δ 8.06 (d, *J* = 1.4 Hz, 1H), 7.29 (t, *J* = 8.4 Hz, 1H), 6.60 (d, *J* =

8.4 Hz, 2H), 6.14 (br s, 1H), 4.59 (dd, J = 6.0, 0.6 Hz, 2H), 3.90 (s, 6H). For the minor rotamer: δ 7.69 (d, J = 11.7 Hz, 1H), 7.32 (t, J = 8.4 Hz, 1H), 6.60 (d, J = 8.4 Hz, 2H), 5.94 (br s, 1H), 4.50 (d, J = 7.0 Hz, 2H), 3.89 (s, 6H); $^{13}C\ \{^1H\}$ NMR (101 MHz, $CDCl_3$) for the major rotamer: 163.7, 160.6, 130.7, 108.4, 104.5, 56.5, 42.2; for the minor rotamer: δ 161.2, 161.0, 131.3, 106.6, 104.4, 56.4, 46.2; HRMS (+APCI) m/z [M+H$^+$] Calcd. for $C_{10}H_{14}O_3NS$, 228.0688; found, 228.0692.

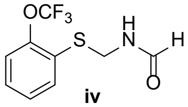

iv

***N*-(((2-(Trifluoromethoxy)phenyl)thio)methyl)formamide (iv):** 2-trifluoromethoxybenzenethiol (1 g, 5.15 mmol) was submitted to the general formamide synthesis procedure with formamide (1.58 mL, 39.7 mmol), paraformaldehyde (634 mg, 21.1 mmol), and formic acid (1.01 mL, 26.8 mmol) for 5 h at 115 °C to afford, after purification on silica gel using a Reveleris purification system (25 g cartridge, 50-100% EtOAc:Hexanes) 865 mg (67 %) of **iv** as a white solid: mp: 37-39 °C; IR (ATR): 3273, 3038, 2878, 1662 cm^{-1}; The ^1H NMR (400 MHz, $CDCl_3$) spectrum was a 5:1 mixture of two rotamers. For the major rotamer: δ 8.13 (d, J = 1.4 Hz, 1H), 7.56 – 7.53 (m, 1H), 7.34 – 7.24 (m, 3H), 6.00 (br s, 1H), 4.74 (d, J = 6.3 Hz, 2H). For the minor rotamer: δ 7.77 (d, J = 11.6 Hz, 1H), 7.42 (ddd, J = 8.2, 7.3, 1.7 Hz, 1H), 7.38 – 7.29 (m, 3H), 5.91 (br s, 1H), 4.58 (d, J = 7.1 Hz, 2H). $^{13}C\{^1H\}$ NMR (101 MHz, $CDCl_3$) for the major rotamer: δ 160.8, 148.6 (q, J = 1.6 Hz), 136.5, 132.9, 129.2, 127.7, 121.4 (q, J = 1.6 Hz), 120.6 (q, J = 259.0 Hz), 41.1. For the minor rotamer: δ 163.6, 150.0 (q, J = 1.4 Hz), 130.9, 127.8, 127.3, 125.2, 121.8 (q, J = 1.4 Hz), 120.6 (q, J = 258.4 Hz). 46.9; HRMS (+APCI) m/z [M+H$^+$] Calcd. for $C_9H_9O_2NF_3S$, 252.0300; found, 252.0301.

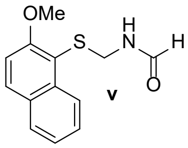

v

***N*-(((2-methoxynaphthalen-1-yl)thio)methyl)formamide (v)**: Following the general formamide synthesis procedure with 2-methoxynaphthalene-1-thiol[2]

(2.74 g, 14.42 mmol), formamide (4.31 mL, 108.17 mmol), paraformaldehyde (1.73 g, 57.69 mmol), and acetic acid (4.12 mL, 72.11 mmol) for 4.5 h at 110 °C afforded, after purification on silica gel using a Reveleris purification system (25 g cartridge, 50-100% EtOAc:Hexanes) 2.68 g (75 %) of **v** as a yellow solid: mp: 118-119 °C; IR (ATR): 3285, 3053, 2939, 1661 cm^{-1}; The ^{1}H NMR (400 MHz, $CDCl_3$) spectrum was a 3:1 mixture of two rotamers. For the major rotamer: δ 8.59-8.57 (m, 1H), 7.99 (d, *J* = 1.5 Hz, 1H), 7.90 (d, *J* = 9.0 Hz, 1H), 7.85-7.75 (m, 1H), 7.55 (ddd, *J* = 8.5, 6.8, 1.2 Hz, 1H), 7.39 (ddd, *J* = 8.1, 6.8, 1.2 Hz, 1H), 7.32 (d, *J* = 9.0 Hz, 1H), 5.95 (br s, 1H), 4.66 (dd, *J* = 6.2, 0.5 Hz, 2H), 4.07 (s, 3H). For the minor rotamer: δ 8.59.8.48 (m, 1H), 7.92 (d, *J* = 8.2 Hz, 1H), 7.82-7.79 (m, 1H), 7.57 (ddd, *J* = 8.7, 7.7, 1.3 Hz, 1H), 7.58 – 7.53 (m, 1H), 7.40 (ddd, *J* = 8.2, 6.8, 1.2 Hz, 1H), 7.32 (d, *J* = 8.2 Hz, 1H), 5.82 (br s, 1H), 4.54 (d, *J* = 7.1 Hz, 2H), 4.05 (s, 3H). $^{13}C\{^{1}H\}$ NMR (101 MHz, $CDCl_3$) for the major rotamer: δ 160.6, 158.6, 136.6, 131.5, 129.6, 128.4, 127.7, 125.3, 124.3, 113.3, 113.0, 57.0, 42.5. For the minor rotamer: δ 163.6, 159.0, 136.3, 132.1, 129.6, 128.6, 128.0, 125.0, 124.4, 114.2, 112.4, 56.9, 46.9. HRMS (ES+) m/z [$M+Na^{+}$] Calcd. for $C_{13}H_{13}NO_2SNa$, 270.0565; found, 270.0574.

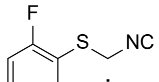
vi

(2-Fluorophenyl)(isocyanomethyl)sulfane (vi): Dehydration of **i** (2.45 g, 13.2 mmol) according to the general dehydration procedure with $POCl_3$ (3.4 mL, 36.4 mmol) and *i*-Pr_2NEt (15 mL, 86 mmol) for 2 h at 0 °C, afforded 1.73 g (78 %) of **vi** as an amber oil: IR (ATR): 3072, 2989, 2946, 2138 cm^{-1}; ^{1}H NMR (400 MHz, $CDCl_3$) δ 7.60 (td, *J* = 7.5, 1.7 Hz, 1H), 7.47 – 7.36 (m, 1H), 7.20 (td, *J* = 7.5, 1.7 Hz, 1H), 7.16 (td, *J* = 9.4, 8.3, 1.7 Hz, 1H), 4.55 (s, 2H); ^{13}C $\{^{1}H\}$ (101 MHz, $CDCl_3$) δ 162.5 (d, *J* = 248.0 Hz), 160.1 (t, *J* = 4.5 Hz), 135.8, 131.9 (d, *J* = 8.1 Hz), 125.3 (d, *J* = 3.9 Hz), 118.2 (d, *J* = 17.7 Hz), 116.5 (d, *J* = 22.6 Hz), 43.8 (td, *J* = 6.9, 3.3 Hz); HRMS (+APCI) m/z [$M-NC^{+}$] Calcd. for C_7H_6FS, 141.0168; found, 141.0170.

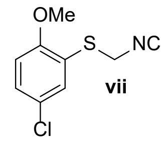
vii

(5-Chloro-2-methoxyphenyl)(isocyanomethyl)sulfane (vii): Dehydration of **ii** (889 mg, 3.84 mmol) according to the general dehydration procedure with $POCl_3$ (0.90 mL, 9.6 mmol) and *i*-Pr_2NEt (3.34 mL, 19.2 mmol) for 3 h at 0 °C afforded, after purification on silica gel using a Reveleris purification system (25 g cartridge, 0-10% EtOAc:Hexanes) 531 mg (65 %) of **vii** as a clear oil: IR (ATR): 3011, 2971, 2940, 2841, 2138 cm^{-1}; ^1H NMR (400 MHz, $CDCl_3$) δ 7.48 (d, J = 2.6 Hz, 1H), 7.32 (dd, J = 8.8, 2.6 Hz, 1H), 6.86 (d, J = 8.8 Hz, 1H), 4.59 (s, 2H), 3.90 (s, 3H); ^{13}C {1H} (101 MHz, $CDCl_3$) δ 159.6, 157.5, 133.7, 130.5, 126.2, 120.7, 112.3, 56.4, 42.6 (t, J = 6.3 Hz); HRMS (+APCI) m/z [M-NC^+] Calcd. for C_8H_8OClS, 186.9978; found, 186.9979. *NOTE:* Compound **vii** is unstable at room temperature; in air **vii** hydrolyzes to the formamide **ii**. Storage at -20 °C resulted in 5-10% formation of **ii**. Subsequent alkylations of **vii** were conducted immediately after purification.

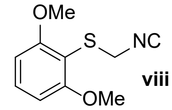
viii

(2,6-Dimethoxyphenyl)(isocyanomethyl)sulfane[3] (viii): Dehydration of **iii** (422 mg, 1.86 mmol) according to the general dehydration procedure with $POCl_3$ (0.43 mL, 4.6 mmol) and *i*-Pr_2NEt (1.94 mL, 11.1 mmol) for 3 h at 0 °C afforded, after purification on silica gel using a Reveleris purification system (25 g cartridge, 0-10% EtOAc:Hexanes) 330 mg (85 %) of **viii** as a white solid exhibiting spectra identical to that of the previously published material.[3]

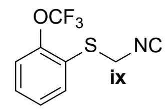
ix

(Isocyanomethyl)(2-(trifluoromethoxy)phenyl)sulfane (ix): Dehydration of **iv** (864 mg, 3.44 mmol) according to the general dehydration procedure $POCl_3$ (0.8

mL, 8.6 mmol) and *i*-Pr_2NEt (3 mL, 17.2 mmol) for 3 h at 0 °C afforded 800 mg (99 %) of **ix** as an amber oil which was used without further purification: IR (ATR): 3071, 2933, 2868, 2140 cm^{-1}; ^{1}H NMR (400 MHz, $CDCl_3$) δ 7.68 (dd, *J* = 8.0, 1.5 Hz, 1H), 7.45 (ddd, *J* = 8.0, 7.1, 1.5 Hz, 1H), 7.37 (ddd, *J* = 4.7, 3.3, 1.5 Hz, 1H), 7.35 (ddd, *J* = 4.7, 3.3, 1.5 Hz, 1H), 4.57 (s, 2H); ^{13}C {1H} (101 MHz, $CDCl_3$) δ 160.4, 149.1 (q, *J* = 1.5 Hz), 134.7, 130.9, 127.9, 125.1, 121.6 (q, *J* = 1.5 Hz), 120.6 (q, *J* = 259.2 Hz), 43.4 (t, *J* = 6.6 Hz); HRMS (+APCI) m/z [M-NC^+] Calcd. for $C_8H_6OF_3S$, 207.0086; found, 207.0086. *NOTE:* Compound **ix** is unstable at room temperature; in air **ix** rapidly hydrolyzes to the formamide **iv**. Storage under nitrogen atmosphere at -20 °C extends shelf life for 1 week after which hydrolysis is observed. Attempts to purify on normal silica gel or deactivated C-2 silica gel[4] resulted in significant loss in yield. Subsequent alkylations of **ix** were conducted immediately without further purification.

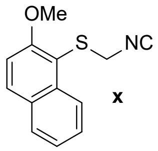

(Isocyanomethyl)(2-methoxynaphthalen-1-yl)sulfane (x): Dehydration of **v** (1.21 g, 4.89 mmol) according to the general dehydration procedure with $POCl_3$ (1.14 mL, 12.22 mmol) and *i*-Pr_2NEt (5.11 mL, 29.33 mmol) for 3 h at 0 °C afforded 1.1 g (99%) of **x** as an amber solid after aqueous workup. The material exhibited spectra indicative of a pure material without need for further purification; the spectral data was identical to that of the previously published material.[3] Purification of **x** on silica gel using a Reveleris purification system (25 g cartridge, 0-10% EtOAc:Hexanes) afforded 738 mg (66 %) of **x** as a pale yellow solid.

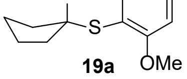

(1-Isocyanocyclopentyl)(2-methoxyphenyl)sulfane (19a): Alkylation of Asmic[5] (**18**) (1 g, 5.58 mmol) with 1-chloro-4-iodobutane (0.72 mL, 5.86 mmol, 1.05 equiv) according to the general alkylation procedure with LDA and dihalides (12.28 mmoL,

2.2 equiv) for 24 h at rt afforded, after purification on silica gel using a Reveleris purification system (25 g cartridge, 0-10% EtOAc:Hexanes), 1.13 g (86 %) of **19a** as a colorless oil exhibiting spectra identical to that of the previously published material.[6]

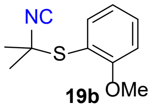

(2-Isocyanopropan-2-yl)(2-methoxyphenyl)sulfane (19b): Alkylation of Asmic[5] (**18**) (500 mg, 2.79 mmol) with methyl iodide (0.38 mL, 6.14 mmol, 2.2 equiv) according to the general alkylation procedure with LDA (6.14 mmol, 2.2 equiv) for 24 h at rt afforded, after purification on silica gel using a Reveleris purification system (25 g cartridge, 0-10% EtOAc:Hexanes), 483 mg (84 %) of **19b** as a waxy solid: IR (ATR): 3064, 2983, 2838, 2124 cm^{-1}; ^{1}H NMR (400 MHz, $CDCl_3$) δ 7.67 (dd, *J* = 7.5, 1.7 Hz, 1H), 7.45 (ddd, *J* = 8.3, 7.5, 1.7 Hz, 1H), 7.01 (td, *J* = 7.5, 1.2 Hz, 1H), 6.98 (dd, *J* = 8.3, 1.2 Hz, 1H), 3.88 (s, 3H), 1.69 (br s, 6H); $^{13}C\{^{1}H\}$ NMR (101 MHz, $CDCl_3$) δ 161.2, 155.8 (t, *J* = 4.1 Hz), 139.8, 132.5, 121.3, 117.8, 111.5, 64.0 (t, *J* = 5.4 Hz), 55.9, 30.7; HRMS (+APCI) m/z [M-NC^{+}] Calcd. for $C_{11}H_{13}OS$, 181.0681; found, 181.0684.

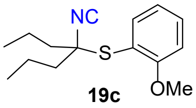

(4-Isocyanoheptan-4-yl)(2-methoxyphenyl)sulfane (19c): Alkylation of Asmic[5] (**18**) (500 mg, 2.79 mmol) with 1-iodopropane (0.6 mL, 6.1 mmol, 2.2 equiv) according to the general alkylation procedure with LDA (6.14 mmol, 2.2 equiv) for 24 h at rt afforded, after purification on silica gel using a Reveleris purification system (25 g cartridge, 0-10% EtOAc:Hexanes), 641 mg (87 %) of **19c** as a colorless oil exhibiting spectra identical to that of the previously published material.[7]

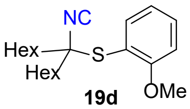

19d

(7-Isocyanotridecan-7-yl)(2-methoxyphenyl)sulfane (19d): Alkylation of Asmic[5] (**18**) (500 mg, 2.79 mmol) with 1-iodohexane (0.86 mL, 5.86 mmol, 2.1 equiv) according to the general alkylation procedure with LDA (6.14 mmol, 2.2 equiv) for 24 h at rt afforded, after purification on silica gel using a Reveleris purification system (25 g cartridge, 0-10% EtOAc:Hexanes), 682 mg (70 %) of **19d** as a colorless oil: IR (ATR): 2954, 2929, 2857, 2119 cm^{-1}; 1H NMR (400 MHz, $CDCl_3$) δ 7.65 (dd, *J* = 7.6, 1.8 Hz, 1H), 7.43 (ddd, *J* = 8.4, 7.6, 1.8 Hz, 1H), 7.00 (td, *J* = 7.6, 1.2 Hz, 1H), 6.95 (dd, *J* = 8.4, 1.2 Hz, 1H), 3.87 (s, 3H), 1.76 (dd, *J* = 9.6, 6.9 Hz, 4H), 1.66 – 1.41 (m, 4H), 1.37 – 1.23 (m, 12H), 0.89 (br. t, *J* = 7.0 Hz, 6H); 13C {1H} NMR (101 MHz, $CDCl_3$) δ 161.4, 156.4 (t, *J* = 4.3 Hz), 139.9, 132.3, 121.3, 117.5, 111.3, 72.0 (t, *J* = 4.3 Hz), 55.9, 39.4, 31.8, 29.2, 24.2, 22.8, 14.2; HRMS (+APCI) m/z [M-NC^+] Calcd. for $C_{20}H_{33}OS$, 321.2246; found, 321.2253.

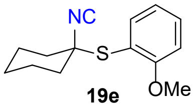

19e

(1-Isocyanocyclohexyl)(2-methoxyphenyl)sulfane[6] (19e): Alkylation of Asmic[5] (**18**) (1 g, 5.58 mmol) with 1-chloro-5-iodopentane (0.86 mL, 6.14 mmol, 1.1 equiv) according to the general alkylation procedure with LDA and dihalides (12.28 mmol, 2.2 equiv) for 24 h at rt afforded, after purification on silica gel using a Reveleris purification system (25 g cartridge, 0-10% EtOAc:Hexanes), 1.03 g (74 %) of **19e** as a white solid exhibiting spectra identical to that of the previously published material.[6]

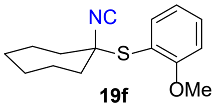

19f

(1-Isocyanocycloheptyl)(2-methoxyphenyl)sulfane[6] (19f): Alkylation of Asmic[5] (**18**) (500 mg, 2.79 mmol) with 1-chloro-6-iodohexane (0.47 mL, 3.07 mmol, 1.1 equiv) according to the general alkylation procedure with LDA (6.14 mmol, 2.2 equiv) and dihalides for 24 h at rt afforded, after purification on silica gel using a Reveleris purification

system (25 g cartridge, 0-10% EtOAc:Hexanes), 290 mg (40 %) of **19f** as a colorless oil exhibiting spectra identical to that of the previously published material.[6]

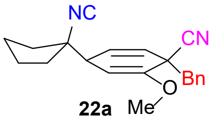

1-Benzyl-4-(1-isocyanocyclopentyl)-2-methoxycyclohexa-2,5-diene-1-carbonitrile (22a): The general exchange-dearomatization with HMTETA was employed with **19a** (30 mg, 0.13 mmol), HMTETA (39 μL, 0.14 mmol), BuLi (0.09 mL, 1.6 M, 0.14 mmol), and benzyl bromide (17 μL, 0.14 mmol) to afford, after purification on silica gel using a Reveleris purification system (12 g cartridge, 0-15% EtOAc:hexanes), 16 mg (78 %) of **22a** as a colorless oil: IR (ATR): 3032, 2961, 2877, 2238, 2127, 1691 cm^{-1}; ^1H NMR (500 MHz, $CDCl_3$) δ 7.28 – 7.23 (m, 3H), 7.13 – 7.07 (m, 2H), 5.89 (ddd, J = 10.0, 3.3, 1.6 Hz, 1H), 5.75 (dd, J = 10.0, 1.6 Hz, 1H), 4.78 (dd, J = 3.3, 1.6 Hz, 1H), 3.71 (s, 3H), 3.25 (ABq, Δν = 60 Hz, J = 13.2 Hz, 2H), 2.81 - 2.78 (m, 1H), 1.97 - 1.82 (m, 4H), 1.74 - 1.61 (m, 4H); ^{13}C {^1H} NMR (101 MHz, $CDCl_3$) δ 155.6 (t, J = 4.4 Hz), 151.8, 134.0, 130.7, 128.2, 128.2, 127.5, 127.3, 126.6, 120.3, 94.7, 71.5 (t, J = 5.0 Hz), 55.1, 43.9, 43.8, 41.8, 37.5, 37.5, 24.0, 23.9; HRMS (+APCI) m/z [$M+H^+$] Calcd. for $C_{21}H_{23}ON_2$, 319.1804; found, 319.1807. **Slow addition protocol:** Neat HMTETA (90 μL, 0.33 mmol) was added to a THF solution (2 mL) of **19a** (70 mg, 0.3 mmol) that was cooled then to -78 °C. After 5 minutes, a hexanes solution of BuLi (1 mL, 0.34 M, 0.33 mmol) was added dropwise over 10 min, then after 10 min, neat benzyl bromide (39 μL, 0.33 mmol) was rapidly added. After 1 h at -78 °C, the reaction was quenched according to the general exchange-dearomatization procedure to afford, after purification on silica gel using a Reveleris purification system (12 g cartridge, 0-15% EtOAc:hexanes), 39 mg (81 %) of **22a**. **1 mmol scale procedure:** The general exchange-dearomatization with HMTETA was employed with **19a** (240 mg, 1.03 mmol), HMTETA (0.31 mL, 1.13 mmol), BuLi (0.96 mL, 1.6 M, 1.54 mmol), and benzyl bromide (0.18

mL, 1.54 mmol) to afford, after purification on silica gel using a Reveleris purification system (12 g cartridge, 0-15% EtOAc:hexanes), 97 mg (59 %) of **22a**.

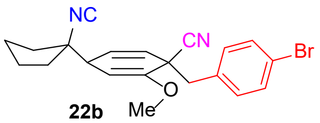

22b

1-(4-Bromobenzyl)-4-(1-isocyanocyclopentyl)-2-methoxycyclohexa-2,5-diene-1-carbonitrile (22b): The general exchange-dearomatization with HMTETA was employed with **19a** (70 mg, 0.3 mmol), HMTETA (90 µL, 0.33 mmol), BuLi (0.21 mL, 1.6 M, 0.33 mmol), and 4-bromobenzyl bromide (83 mg, 0.33 mmol) to afford, after purification on silica gel using a Reveleris purification system (12 g cartridge, 0-15% EtOAc:hexanes), 47 mg (79 %) of **22b** as a white solid: mp: 119-121 °C; IR (ATR): 2958, 2875, 2237, 2127, 1691 cm^{-1}; ^{1}H NMR (400 MHz, $CDCl_3$) δ 7.38 (d, *J* = 8.3 Hz, 2H), 6.97 (d, *J* = 8.3 Hz, 2H), 5.91 (ddd, *J* = 10.1, 3.3, 1.7 Hz, 1H), 5.73 (dd, *J* = 10.1, 1.7 Hz, 1H), 4.79 (dd, *J* = 3.3, 1.7 Hz, 1H), 3.70 (s, 3H), 3.20 (ABq, Δν = 68.1 Hz, *J* = 13.3 Hz, 2H), 2.84-2.81 (m, 1H), 2.00 - 1.80 (m, 4H), 1.76 - 1.60 (m, 4H); ^{13}C {^{1}H} NMR (101 MHz, $CDCl_3$) δ 155.8 (t, *J* = 4.4 Hz), 151.4, 133.1, 132.3, 131.4, 127.8, 126.3, 121.8, 120.0, 95.1, 71.5 (t, *J* = 5.0 Hz), 55.1, 43.9, 43.2, 41.6, 37.6, 37.6, 23.95, 23.90; HRMS (+APCI) m/z [$M+H^{+}$] Calcd. for $C_{21}H_{22}ON_2Br$, 397.0910; found, 397.0916.

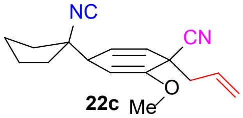

22c

1-Allyl-4-(1-isocyanocyclopentyl)-2-methoxycyclohexa-2,5-diene-1-carbonitrile (22c): The general exchange-dearomatization with HMTETA was employed with **19a** (70 mg, 0.3 mmol), HMTETA (90 µL, 0.33 mmol), BuLi (0.21 mL, 1.6 M, 0.33 mmol), and allyl bromide (29 µL, 0.33 mmol) to afford, after purification on silica gel using a Reveleris purification system (12 g cartridge, 0-15% EtOAc:hexanes), 29 mg (72 %) of

22c as a colorless oil: IR (ATR): 3079, 3011, 2960, 2877, 2841, 2238, 2127, 1690 cm^{-1}; ^{1}H NMR (400 MHz, $CDCl_3$) δ 6.00 (ddd, *J* = 10.0, 3.4, 1.8 Hz, 1H), 5.78 (dd, *J* = 10.0, 1.8 Hz, 1H), 5.61 (dddd, *J* = 17.0, 10.0, 7.7, 6.8 Hz, 1H), 5.19 – 5.08 (m, 2H), 4.91 (dd, *J* = 3.4, 1.8 Hz, 1H), 3.67 (s, 3H), 3.20 (tq, *J* = 3.4, 1.8 Hz, 1H), 2.71 (ddt, *J* = 13.6, 7.7, 13.7, 1.2 Hz, 1H), 2.69 (ddt, *J* = 13.6, 6.8, 1.1 Hz, 1H), 2.03 – 1.85 (m, 4H), 1.83 – 1.66 (m, 4H); ^{13}C {^{1}H} NMR (101 MHz, $CDCl_3$) δ 155.7 (t, *J* = 5.1 Hz), 152.2, 130.8, 127.2, 126.9, 120.4, 120.1, 94.3, 71.7 (t, *J* = 5.1 Hz), 55.2, 44.1, 42.2, 40.4, 37.6, 37.5, 24.0, 23.9; HRMS (+APCI) m/z [$M+H^{+}$] Calcd. for $C_{17}H_{21}ON_2$, 269.1648; found, 269.1651.

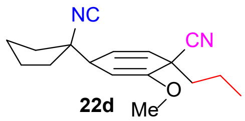

4-(1-Isocyanocyclopentyl)-2-methoxy-1-propylcyclohexa-2,5-diene-1-carbonitrile (22d): The general exchange-dearomatization with HMTETA was employed with **19a** (70 mg, 0.3 mmol), HMTETA (90 μL, 0.33 mmol), BuLi (0.21 mL, 1.6 M, 0.33 mmol), and propyl iodide (32 μL, 0.33 mmol) to afford, after purification on silica gel using a Reveleris purification system (12 g cartridge, 0-15% EtOAc:hexanes), 24 mg (59 %) of **22d** as a colorless oil: IR (ATR): 3041, 2961, 2937, 2875, 2236, 2126, 1690 cm^{-1}; ^{1}H NMR (400 MHz, $CDCl_3$) δ 6.00 (ddd, *J* = 10.0, 3.7, 1.7 Hz, 1H), 5.76 (dd, *J* = 10.0, 1.7 Hz, 1H), 4.90 (dd, *J* = 3.7, 1.7 Hz, 1H), 3.67 (s, 3H), 3.22 (tq, *J* = 3.7, 1.7 Hz, 1H), 2.05 (ddd, *J* = 13.2, 11.9, 4.8 Hz, 1H), 2.00 – 1.86 (m, 4H), 1.79 (ddd, *J* = 13.2, 11.9, 4.8 Hz, 1H), 1.75 – 1.67 (m, 4H), 1.37 – 1.09 (m, 2H), 0.91 (t, *J* = 7.3 Hz, 3H); ^{13}C {^{1}H} NMR (101 MHz, $CDCl_3$) δ 155.7 (t, *J* = 4.5 Hz), 152.5, 127.6, 127.1, 120.7, 94.1, 71.7 (t, *J* = 5.3 Hz), 55.2, 44.1, 40.6, 39.8, 37.5, 24.01, 23.97, 17.3, 13.9; HRMS (+APCI) m/z [$M+H^{+}$] Calcd. for $C_{16}H_{22}ON$, 244.1695; found, 244.1697.

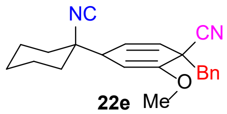

22e

4-Benzyl-1'-isocyano-3-methoxy-[1,1'-bi(cyclohexane)]-2,5-diene-4-carbonitrile (22e): The general exchange-dearomatization with HMTETA was employed with **19e** (75 mg, 0.3 mmol), HMTETA (91 μL, 0.33 mmol), BuLi (0.21 mL, 1.6 M, 0.33 mmol), and benzyl bromide (40 μL, 0.33 mmol) to afford, after purification on silica gel using a Reveleris purification system (12 g cartridge, 0-15% EtOAc:hexanes), 27 mg (54 %) of **22e** as a colorless oil: IR (ATR): 3031, 2934, 2236, 2127, 1690 cm^{-1}; ^{1}H NMR (400 MHz, $CDCl_3$) δ 7.30 – 7.21 (m, 3H), 7.15 – 7.06 (m, 2H), 5.91 (ddd, *J* = 10.0, 3.4, 1.5 Hz, 1H), 5.74 (dd, *J* = 10.0, 1.5 Hz, 1H), 4.83 (dd, *J* = 3.4, 1.5 Hz, 1H), 3.72 (s, 3H), 3.25 (ABq, Δν = 41.8 Hz, *J* = 13.2 Hz, 2H), 2.79 – 2.72 (m, 1H), 1.77 – 1.58 (m, 7H), 1.45 - 1.21 (m, 2H), 1.14 – 0.99 (m, 1H); ^{13}C {^{1}H} NMR (101 MHz, $CDCl_3$) δ 156.5 (t, *J* = 4.0 Hz), 151.7, 134.1, 130.6, 128.3, 127.6, 126.9, 126.8, 120.2, 94.2, 65.5 (t, *J* = 4.8 Hz), 55.1, 45.8, 43.7, 41.7, 33.4, 33.0, 25.1, 21.7, 21.6; HRMS (+APCI) m/z [$M+H^+$] Calcd. for $C_{22}H_{25}ON_2$, 333.1961; found, 333.1961.

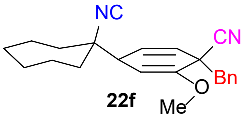

22f

1-Benzyl-4-(1-isocyanocycloheptyl)-2-methoxycyclohexa-2,5-diene-1-carbonitrile (22f): The general exchange-dearomatization with HMTETA was employed with **19f** (79 mg, 0.3 mmol), HMTETA (90 μL, 0.33 mmol), BuLi (0.21 mL, 1.6 M, 0.33 mmol), and benzyl bromide (40 μL, 0.33 mmol) to afford, after purification on silica gel using a Reveleris purification system (12 g cartridge, 0-15% EtOAc:hexanes), 45 mg (86 %) of **22f** as a colorless oil: IR (ATR): 3032, 2929, 2235, 2126, 1690 cm^{-1}; ^{1}H NMR (400 MHz, $CDCl_3$) δ 7.30 – 7.21 (m, 3H), 7.13 – 7.06 (m, 2H), 5.90 (ddd, *J* = 10.1, 3.3, 1.8 Hz, 1H), 5.73 (dd, *J* = 10.1, 1.8 Hz, 1H), 4.83 (dd, *J* = 3.3, 1.8 Hz, 1H), 3.72 (s, 3H), 3.24 (ABq, Δν = 39.7 Hz, *J* = 13.2 Hz, 2H), 2.81 – 2.74 (m, 1H), 1.83 - 1.38 (m, 12H); ^{13}C {^{1}H} NMR (101 MHz, $CDCl_3$) δ 155.8 (t, *J* = 4.3 Hz), 151.7, 134.0, 130.6, 128.3, 127.6, 127.2, 126.7, 120.3, 94.7, 68.06 (t, *J* = 5.0 Hz),

55.1, 47.7, 43.7, 41.8, 37.3, 36.6, 27.55, 27.54, 22.84, 22.77; HRMS (+APCI) m/z [M+H$^+$] Calcd. for $C_{23}H_{27}ON_2$, 347.2117; found, 347.2117.

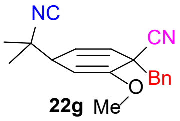

22g

1-Benzyl-4-(2-isocyanopropan-2-yl)-2-methoxycyclohexa-2,5-diene-1-carbonitrile (22g): The general exchange-dearomatization with HMTETA was employed with **19b** (63 mg, 0.3 mmol), HMTETA (91 μL, 0.33 mmol), BuLi (0.21 mL, 1.6 M, 0.33 mmol), and benzyl bromide (40 μL, 0.33 mmol) to afford, after purification on silica gel using a Reveleris purification system (12 g cartridge, 0-15% EtOAc:hexanes), 32 mg (72 %) of **22g** as a colorless oil: IR (ATR): 3033, 2986, 2938, 2236, 2130, 1689 cm^{-1}; ^{1}H NMR (400 MHz, $CDCl_3$) δ 7.29 – 7.23 (m, 3H), 7.14 – 7.07 (m, 2H), 5.88 (ddd, J = 10.1, 3.6, 1.7 Hz, 1H), 5.76 (dd, J = 10.1, 1.7 Hz, 1H), 4.79 (dd, J = 3.6, 1.7 Hz, 1H), 3.72 (s, 3H), 3.25 (ABq, Δν = 40.6 Hz, J = 13.2, 2H), 2.79 – 2.73 (m, 1H), 1.35 (t, J = 2.1 Hz, 3H), 1.31 (t, J = 2.1 Hz, 3H); ^{13}C {1H} (101 MHz, $CDCl_3$) δ 154.9 (t, J = 5.2 Hz), 151.9, 133.9, 130.6, 128.2, 127.6, 126.9, 126.7, 120.1, 94.2, 60.4 (t, J = 5.2 Hz), 55.1, 45.9, 43.7, 41.7, 26.3, 25.9; HRMS (+APCI) m/z [M+H$^+$] Calcd. for $C_{19}H_{21}ON_2$, 293.1648; found, 293.1649.

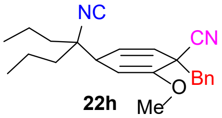

22h

1-Benzyl-4-(4-isocyanoheptan-4-yl)-2-methoxycyclohexa-2,5-diene-1-carbonitrile (22h): The general exchange-dearomatization with HMTETA was employed with **19c** (80 mg, 0.33 mmol), HMTETA (91 μL, 0.33 mmol), BuLi (0.21 mL, 1.6 M, 0.33 mmol), and benzyl bromide (40 μL, 0.33 mmol) to afford, after purification on silica gel using a Reveleris purification system (12 g cartridge, 0-15% EtOAc:hexanes), 36 mg (68 %) of **22h** as a colorless oil: IR (ATR): 3033, 2962, 2934, 2236, 2126, 1690 cm^{-1}; ^{1}H NMR (400 MHz,

$CDCl_3$) δ 7.30 – 7.22 (m, 3H), 7.14 – 7.06 (m, 2H), 5.83 (ddd, *J* = 10.1, 3.4, 1.5 Hz, 1H), 5.72 (dd, *J* = 10.1, 1.5 Hz, 1H), 4.70 (dd, *J* = 3.4, 1.5 Hz, 1H), 3.72 (s, 3H), 3.24 (ABq, Δν = 35.4 Hz, *J* = 13.2 Hz, 2H), 2.89 – 2.82 (m, 1H), 1.54 – 1.31 (m, 8H), 0.97 – 0.89 (m, 6H); ^{13}C {1H} (101 MHz, $CDCl_3$) δ 155.9 (t, *J* = 4.6 Hz), 151.6, 134.0, 130.6, 128.2, 127.6, 127.1, 126.6, 120.1, 94.3, 66.7 (t, *J* = 4.9 Hz), 55.1, 43.8, 42.5, 41.8, 38.1, 38.0, 17.06, 17.05, 14.29, 14.27; HRMS (+APCI) m/z [M+H$^+$] Calcd. for $C_{23}H_{29}ON_2$, 349.2274; found, 349.2274.

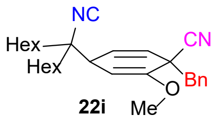

22i

1-Benzyl-4-(7-isocyano-6,8-dioxotridecan-7-yl)-2-methoxycyclohexa-2,5-diene-1-carbonitrile (22i): The general exchange-dearomatization with HMTETA was employed with **19d** (105 mg, 0.3 mmol), HMTETA (90 µL, 0.33 mmol), BuLi (0.21 mL, 1.6 M, 0.33 mmol), and benzyl bromide (40 µL, 0.33 mmol) to afford, after purification on silica gel using a Reveleris purification system (12 g cartridge, 0-10% EtOAc:hexanes), 38 mg (58 %) of **22i** as a colorless oil: IR (ATR): 3032, 2929, 2235, 2125, 1690, 1652 cm^{-1}; ^1H NMR (400 MHz, $CDCl_3$) δ 7.29 – 7.23 (m, 3H), 7.14 – 7.08 (m, 2H), 5.82 (ddd, *J* = 10.1, 3.4, 1.7 Hz, 1H), 5.72 (dd, *J* = 10.1, 1.7 Hz, 1H), 4.70 (dd, *J* = 3.4, 1.7 Hz, 1H), 3.71 (s, 3H), 3.23 (ABq, Δν = 27.1 Hz, *J* = 13.2 Hz, 2H), 2.89 – 2.83 (m, 1H), 1.54 – 1.18 (m, 20H), 0.88 (br. t, *J* = 6.8 Hz, 6H); ^{13}C {1H} (101 MHz $CDCl_3$) δ 155.8 (t, *J* = 5.1 Hz), 151.7, 134.0, 130.7, 128.2, 127.6, 127.1, 126.6, 120.1, 94.3, 66.8 (t, *J* = 5.1 Hz), 55.1, 43.8, 42.4, 41.8, 35.9, 35.8, 31.80, 31.79, 29.46, 29.44, 23.67, 23.66, 22.74, 22.72, 14.2; HRMS (+APCI) m/z [M+H$^+$] Calcd. for $C_{29}H_{41}ON_2$, 433.3213; found, 433.3214.

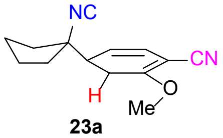

23a

4-(1-Isocyanocyclopentyl)-2-methoxycyclohexa-1,5-diene-1-carbo-nitrile (23a): The general exchange-dearomatization with HMTETA was employed with **19a** (70 mg, 0.3 mmol), HMTETA (90 μL, 0.33 mmol), and a saturated, aqueous NH_4Cl solution (excess) to afford, after purification on silica gel using a Reveleris purification system (12 g cartridge, 0-15% EtOAc:hexanes), 25 mg (73 %) of **23a** as a colorless oil: IR (ATR): 2952, 2211, 2127, 1640, 1586 cm^{-1}; ^{1}H NMR (400 MHz, $CDCl_3$) δ 6.00 (dd, J = 9.7, 2.0 Hz, 1H), 5.48 (dd, J = 9.7, 2.7 Hz, 1H), 4.09 (s, 3H), 2.74 – 2.49 (m, 3H), 2.15 – 1.55 (m, 8H); ^{13}C {1H} NMR (101 MHz, $CDCl_3$) δ 168.9, 156.9 (t, J = 4.3 Hz), 125.6, 118.8, 117.9, 83.2, 71.1 (t, J = 5.1 Hz), 57.8, 42.2, 38.4, 37.6, 29.2, 23.3; HRMS (+APCI) m/z [M+H$^+$] Calcd. for $C_{14}H_{17}ON_2$, 229.1335; found, 229.1338.

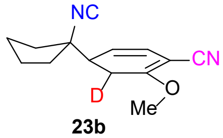

23b

4-(1-Isocyanocyclopentyl)-2-methoxycyclohexa-1,5-diene-1-carbo-nitrile-3-*d* (23b): The general exchange-dearomatization with HMTETA was employed with **19a** (70 mg, 0.3 mmol), HMTETA (90 μL, 0.33 mmol), and deuterium oxide (0.1 mL) to afford, after purification on silica gel using a Reveleris purification system (12 g cartridge, 0-15% EtOAc:hexanes), 25 mg (73 %) of **23b** as a colorless oil: IR (ATR): 3056, 2951, 2213, 2127, 1639, 1582 cm^{-1}; ^{1}H NMR (400 MHz, $CDCl_3$) δ 6.00 (dd, J = 9.8, 2.0 Hz, 1H), 5.48 (dd, J = 9.8, 2.6 Hz, 1H), 4.09 (s, 3H), 2.72 – 2.60 (m, 2H), 2.14 –1.87 (m, 3H), 1.84 –1.59 (m, 3H); ^{13}C {1H} (101 MHz, $CDCl_3$) δ 168.8, 157.0 (t, J = 4.2 Hz), 125.6 (d, J = 2.3 Hz), 118.8, 117.9, 83.2, 71.1 (t, J = 4.8 Hz), 57.9 (q, J = 3.1 Hz), 42.3, 38.4, 37.7, 29.0 (t, J = 19.9 Hz), 23.4; HRMS (+APCI) m/z [M-NC$^+$] Calcd. for $C_{13}H_{15}DON$, 203.1289; found, 203.1289.

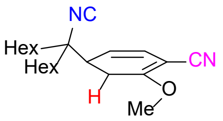

23c

4-(7-isocyano-6,8-dioxotridecan-7-yl)-2-methoxycyclohexa-1,5-diene-1-carbonitrile (23c): The general exchange-dearomatization with HMTETA was employed with **19d** (105 mg, 0.3 mmol), HMTETA (90 µL, 0.33 mmol), BuLi (0.24 mL, 1.4 M, 0.33 mmol), and a saturated, aqueous NH_4Cl solution (1 mL) to afford, after purification on silica gel using a Reveleris purification system (12 g cartridge, 0-15% EtOAc:hexanes), 28 mg (54 %) of **23c** as a colorless oil: IR (ATR): 2953, 2928, 2213, 2126, 1641, 1588 cm^{-1}; ^{1}H NMR (400 MHz, $CDCl_3$) δ 5.98 (dd, J = 9.8, 2.3 Hz, 1H), 5.44 (dd, J = 9.8, 3.7 Hz, 1H), 4.09 (s, 3H), 2.89 – 2.78 (m, 1H), 2.53 (dd, J = 10.3, 2.1 Hz, 2H), 1.57 – 1.52 (m, 2H), 1.46 – 1.27 (m, 18H), 0.90 (br. t, J = 6.8 Hz, 6H); ^{13}C {1H} (101 MHz, $CDCl_3$) δ 168.8, 156.3 (t, J = 4.1 Hz), 125.4, 118.1, 117.9, 83.2, 66.6 (t, J = 4.8 Hz) 57.9, 40.0, 35.3, 35.2, 31.79, 31.78, 29.50, 29.46, 28.0, 23.64, 23.62, 22.74, 22.73, 14.20, 14.19; HRMS (+APCI) m/z [M+H$^+$] Calcd. for $C_{22}H_{35}ON_2$, 343.2743; found, 343.2752.

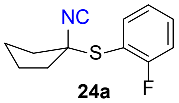

24a

(2-Fluorophenyl)(1-isocyanocyclopentyl)sulfane (24a): Alkylation of **vi** (753 mg, 4.5 mmol) with 1-chloro-4-iodobutane (0.58 mL, 4.73 mmol) following the general alkylation procedure with BuLi (2.96 mL, 1.6 M, 4.73 mmol) and LDA (4.95 mmol, 1.1 equiv) with dihalides for 24 h at rt afforded, after purification on silica gel using a Reveleris purification system (25 g cartridge, 0-10% EtOAc:Hexanes), 700 mg (70 %) of **24a** as a colorless oil: IR (ATR): 3073, 2956, 2120 cm^{-1}; ^{1}H NMR (400 MHz, $CDCl_3$) δ 7.74 (td, J = 7.5, 1.8 Hz, 1H), 7.46 (dddd, J = 8.3, 7.5, 5.1, 1.8 Hz, 1H), 7.22 (td, J = 7.5, 1.8 Hz, 1H), 7.18 (td, J = 8.3, 1.8 Hz, 1H), 2.24 – 2.02 (m, 4H), 1.98 – 1.83 (m, 4H); ^{13}C {1H} NMR (101 MHz, $CDCl_3$) δ 163.8 (d, J = 248.8 Hz), 157.2, 139.1, 132.8 (d, J = 8.1 Hz), 125.0 (d, J = 4.2 Hz), 117.7 (d, J = 18.5 Hz),

116.3 (d, J = 23.7 Hz), 72.4 (t, J = 5.5 Hz), 42.0, 23.3.; HRMS (+APCI) m/z [M-NC$^+$] Calcd. for $C_{11}H_{12}FS$, 195.0638; found, 195.0641.

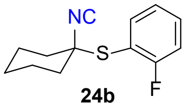

24b

(2-Fluorophenyl)(1-isocyanocyclohexyl)sulfane (24b): Alkylation of **vi** (200 mg, 1.20 mmol) with 1-chloro-5-iodopentane (0.18 mL, 1.32 mmol) following the general alkylation procedure with BuLi (0.98 mL, 1.34 M, 1.32 mmol) and LDA (1.32 mmol, 1.1 equiv) with dihalides for 24 h at rt afforded, after purification on silica gel using a Reveleris purification system (12 g cartridge, 0-10% EtOAc:Hexanes), 135 mg (48 %) of **24b** as a colorless oil: IR (ATR): 2939, 2862, 2120 cm^{-1}; ^{1}H NMR (400 MHz, $CDCl_3$) δ 7.72 (td, J = 7.4, 1.8 Hz, 1H), 7.47 (dddd, J = 8.3, 7.4, 5.1, 1.8 Hz, 1H), 7.22 (td, J = 7.4, 1.3 Hz, 1H), 7.17 (td, J = 8.3, 1.3 Hz, 1H), 2.00 (dt, J = 13.3, 3.8 Hz, 2H), 1.86 – 1.55 (m, 7H), 1.38 – 1.20 (m, 1H); ^{13}C {1H} NMR (101 MHz, $CDCl_3$) δ 164.1 (d, J = 248.9 Hz), 157.5, 139.9, 132.9 (d, J = 8.3 Hz), 125.0 (d, J = 4.1 Hz), 116.2 (d, J = 23.7 Hz), 116.1 (d, J = 18.4 Hz), 68.6 (t, J = 5.3 Hz), 38.2, 24.7, 22.4; HRMS (+APCI) m/z [M-NC$^+$] Calcd. for $C_{12}H_{14}FS$, 209.0794; found, 209.0797.

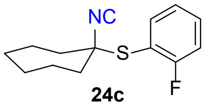

24c

(2-Fluorophenyl)(1-isocyanocycloheptyl)sulfane (24c): Alkylation of **vi** (200 mg, 1.20 mmol) with 1-chloro-6-iodohexane (0.2 mL, 1.32 mmol) following the general alkylation procedure with BuLi (0.78 mL, 1.6 M, 1.26 mmol) and LDA (1.32 mmol, 1.1 equiv) with dihalides for 24 h at rt afforded, after purification on silica gel using a Reveleris purification system (12 g cartridge, 0-10% EtOAc:Hexanes), 120 mg (40 %) of **24c** as a colorless oil: IR (ATR): 3072, 2932, 2118 cm^{-1}; ^{1}H NMR (500 MHz, $CDCl_3$) δ 7.73 (td, J = 7.6, 1.8 Hz, 1H), 7.47 (dddd, J = 8.5, 7.6, 5.1, 1.8 Hz, 1H), 7.22 (td, J = 7.6, 1.4 Hz, 1H), 7.18 (td, J = 8.5, 1.4 Hz, 1H), 2.10 (dd, J = 15.0, 7.0 Hz, 2H), 2.01 – 1.92 (m, 2H), 1.76 – 1.54 (m, 8H); ^{13}C

{1H} NMR (101 MHz, $CDCl_3$) δ 164.1 (d, *J* = 248.8 Hz), 156.9, 139.9, 133.0 (d, *J* = 8.3 Hz), 125.0 (d, *J* = 4.0 Hz), 117.1 (d, *J* = 18.4 Hz), 116.3 (d, *J* = 23.7 Hz), 71.7 (t, *J* = 5.2 Hz), 41.4, 28.2, 22.7; HRMS (+APCI) m/z [M-NC^+] Calcd. for $C_{13}H_{16}FS$, 223.0951; found, 223.0954.

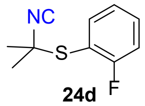

24d

(2-Fluorophenyl)(2-isocyanopropan-2-yl)sulfane (24d): Alkylation of **vi** (200 mg, 1.2 mmol) with methyl iodide (0.16 mL, 2.6 mmol) following the general alkylation procedure with LiHMDS (2.6 mL, 2.6 mmol, 2.2 equiv) for 24 h at rt afforded, after purification on silica gel using a Reveleris purification system (12 g cartridge, 0-10% EtOAc:Hexanes), 167 mg (72 %) of **24d** as a colorless oil: IR (ATR): 2986, 2936, 2124 cm^{-1}; ^1H NMR (400 MHz, $CDCl_3$) δ 7.72 (td, *J* = 7.6, 1.8 Hz, 1H), 7.49 (dddd, *J* = 8.5, 7.6, 5.1, 1.8 Hz, 1H), 7.24 (td, *J* = 7.6, 1.3 Hz, 1H), 7.20 (td, *J* = 8.5, 1.3 Hz, 1H), 1.72 (s, 6H); ^{13}C {1H} (101 MHz, $CDCl_3$) δ 164.0 (d, *J* = 248.9 Hz), 156.7, 139.7, 133.2 (d, *J* = 8.3 Hz), 125.1 (d, *J* = 4.2 Hz), 117.0 (d, *J* = 18.4 Hz), 116.4 (d, *J* = 23.9 Hz), 64.1 (t, *J* = 5.6 Hz), 30.7; HRMS (+APCI) m/z [M-NC^+] Calcd. for $C_9H_{10}FS$, 169.0481; found, 169.0483.

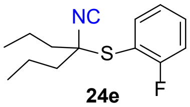

24e

(2-Fluorophenyl)(4-isocyanoheptan-4-yl)sulfane (24e): Alkylation of **vi** (750 mg, 4.49 mmol) with 1-iodopropane (0.92 mL, 9.42 mmol) following the general alkylation procedure with LDA (9.86 mmol, 2.2 equiv) for 24 h at rt afforded, after purification on silica gel using a Reveleris purification system (25 g cartridge, 0-10% EtOAc:Hexanes), 901 mg (80 %) of **24e** as a colorless oil: IR (ATR): 2935, 2118 cm^{-1}; ^1H NMR (400 MHz, $CDCl_3$) δ 7.70 (td, *J* = 7.4, 1.8 Hz, 1H), 7.47 (dddd, *J* = 8.5, 7.4, 5.1, 1.8 Hz, 1H), 7.22 (td, *J* = 7.4, 1.3 Hz, 1H), 7.18 (td, *J* = 8.5, 1.3 Hz, 1H), 1.84 – 1.40 (m, 8H), 0.95 (t, *J* = 7.3 Hz, 6H); ^{13}C {1H} (101 MHz, $CDCl_3$) δ 164.3 (d, *J* = 248.8 Hz), 157.4, 139.9, 132.9 (d, *J* = 8.4 Hz), 125.0 (d, *J* = 4.1 Hz), 116.7

(d, J = 18.5 Hz), 116.3 (d, J = 23.7 Hz), 72.0 (t, J = 5.2 Hz), 41.4, 17.7, 13.9; HRMS (+APCI) m/z [M-NC^+] Calcd. for $C_{13}H_{18}FS$, 225.1107; found, 225.1111.

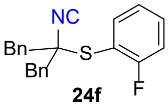

24f

(2-Fluorophenyl)(2-isocyano-1,3-diphenylpropan-2-yl)sulfane (24f): Alkylation of **vi** (300 mg, 1.79 mmol) with benzyl bromide (0.46 mL, 3.94 mmol) following the general alkylation procedure with LiHMDS (3.94 mL, 3.94 mmol, 2.2 equiv) for 24 h at rt afforded, after purification on silica gel using a Reveleris purification system (25 g cartridge, 0-10% EtOAc:Hexanes), 375 mg (60 %) of **24f** as a white solid: m.p: 141-143 °C; IR (ATR): 3063, 3031, 2926, 2120 cm^{-1}; ^{1}H NMR (400 MHz, $CDCl_3$) δ 7.60 (td, J = 7.4, 1.8 Hz, 1H), 7.47 (tdd, J = 7.4, 5.1, 1.8 Hz, 1H), 7.38 – 7.28 (m, 10H), 7.23 – 7.16 (m, 2H), 3.10 (ABq, Δν = 51.8 Hz, J = 14.0 Hz, 4H); ^{13}C {1H} (101 MHz, $CDCl_3$) δ 164.3 (d, J = 248.8 Hz), 160.4, 139.8, 134.0, 133.1 (d, J = 8.3 Hz), 128.5, 128.0, 125.2 (d, J = 4.1 Hz), 116.41 (d, J = 18.4 Hz), 116.38 (d, J = 23.8 Hz), 71.4, 46.4; HRMS (+APCI) m/z [M-NC^+] Calcd. for $C_{21}H_{18}FS$, 321.1107; found, 321.1111.

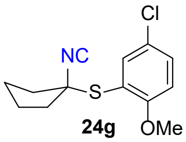

24g

(5-Chloro-2-methoxyphenyl)(1-isocyanocyclopentyl)sulfane (24g): Alkylation of **vii** (300 mg, 1.4 mmol) with 1-chloro-4-iodobutane (0.19 mL, 1.54 mmol) following the general alkylation procedure with LDA (3.08 mmol, 2.2 equiv) and dihalides for 24 h at rt afforded, after purification on silica gel using a Reveleris purification system (25 g cartridge, 0-10% EtOAc:Hexanes), 331 mg (88 %) of **24g** as a colorless oil: IR (ATR): 2962, 2121 cm^{-1}; ^{1}H NMR (400 MHz, $CDCl_3$) δ 7.63 (d, J = 2.7 Hz, 1H), 7.37 (dd, J = 8.8, 2.7 Hz, 1H), 6.89 (d, J = 8.8 Hz, 1H), 3.87 (s, 3H), 2.25 – 2.00 (m, 4H), 1.96 – 1.81 (m, 4H); ^{13}C {1H} (101 MHz, $CDCl_3$) δ 159.4, 156.9 (br. t, J = 3.5 Hz), 137.7, 131.6, 125.7, 120.5, 112.5, 72.11 (t, J = 5.5 Hz),

56.3, 42.1, 23.4; HRMS (+APCI) m/z [M-NC$^+$] Calcd. for $C_{12}H_{14}OClS$, 241.0448; found, 241.0449.

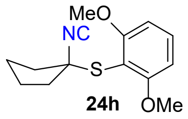
24h

(2,6-Dimethoxyphenyl)(1-isocyanocyclopentyl)sulfane (24h): Alkylation of **viii** (300 mg, 1.43 mmol) with 1-chloro-4-iodobutane (0.18 mL, 1.51 mmol) following the general alkylation procedure with LDA (3.16 mmol, 2.2 equiv) and dihalides for 24 h at rt afforded, after purification on silica gel using a Reveleris purification system (12 g cartridge, 0-10% EtOAc:Hexanes), 171 mg (45 %) of **24h** as a colorless oil: IR (ATR): 3002, 2966, 2941, 2838, 2875, 2124 cm^{-1}; ^{1}H NMR (400 MHz, $CDCl_3$) δ 7.37 (t, J = 8.4 Hz, 1H), 6.62 (d, J = 8.4 Hz, 2H), 3.87 (s, 6H), 2.18 – 2.04 (m, 4H), 1.99 – 1.77 (m, 4H); ^{13}C {1H} (101 MHz, $CDCl_3$) δ 162.7, 155.8 (t, J = 4.0 Hz), 132.5, 106.3, 104.3, 72.40 (t, J = 5.5 Hz), 56.3, 42.1, 23.4; HRMS (+APCI) m/z [M-NC$^+$] Calcd. for $C_{13}H_{17}O_2S$, 237.0943; found, 237.0945.

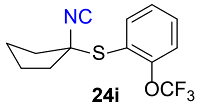
24i

(1-Isocyanocyclopentyl)(2-(trifluoromethoxy)phenyl)sulfane (24i): Alkylation of **ix** (420 mg, 1.8 mmol) with 1-chloro-4-iodobutane (0.24 mL, 1.98 mmol) following the general alkylation procedure with LDA (3.96 mmol, 2.2 equiv) and dihalides for 24 h at rt afforded, after purification on silica gel using a Reveleris purification system (25 g cartridge, 0-10% EtOAc:Hexanes), 397 mg (77 %) of **24i** as a colorless oil: IR (ATR): 3077, 2973, 2121, 1248, 1206, 1159 cm^{-1}; ^{1}H NMR (500 MHz, $CDCl_3$) δ 7.88 (dd, J = 8.2, 1.7 Hz, 1H), 7.50 (ddd, J = 8.2, 7.5, 1.7 Hz, 1H), 7.41 – 7.35 (m, 2H), 2.21 – 2.05 (m, 4H), 1.97 – 1.83 (m, 4H); ^{13}C {1H} (101 MHz, $CDCl_3$) δ 157.4, 150.8 (q, J = 1.6 Hz), 138.8, 131.9, 127.6, 124.5, 120.6 (q, J = 258.8), 121.3 (q, J = 1.6 Hz), 72.4 (t, J = 5.7 Hz), 42.0, 23.3; HRMS (+APCI) m/z [M-NC$^+$] Calcd.

for $C_{12}H_{12}OF_3S$, 261.0555; found, 261.05585. *NOTE*: Compound **24i** was stored under nitrogen at -20 °C because decomposition occurred at room temperature.

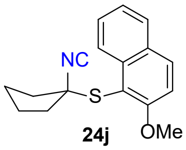

24j

(1-isocyanocyclopentyl)(2-methoxynaphthalen-1-yl)sulfane (24j): Alkylation of **x** (300 mg, 1.31 mmol) with 1-chloro-4-iodobutane (0.18 mL, 1.44 mmol) following the general alkylation procedure with LDA (3.96 mmol, 2.2 equiv) and dihalides for 5 h at rt afforded, after purification on silica gel using a Reveleris purification system (25 g cartridge, 0-10% EtOAc:Hexanes), 262 mg (71 %) of **24j** as a white solid: mp: 84-85 °C; IR (ATR): 3061, 2961, 2121 cm^{-1}; ^{1}H NMR (400 MHz, $CDCl_3$) δ 8.68 (dt, *J* = 8.4, 0.8 Hz, 1H), 7.96 (d, *J* = 9.1 Hz, 1H), 7.80 (dt, *J* = 8.4, 0.8 Hz, 1H), 7.57 (ddd, *J* = 8.4, 6.8, 1.4 Hz, 1H), 7.39 (ddd, *J* = 8.4, 6.8, 1.4 Hz, 1H), 7.35 (d, *J* = 9.1 Hz, 1H), 4.03 (s, 3H), 2.23 – 2.03 (m, 4H), 1.98 – 1.78 (m, 4H); ^{13}C {1H} NMR (101 MHz, $CDCl_3$) δ 160.4, 156.5, 137.5, 133.0, 129.5, 128.3, 127.7, 125.8, 124.2, 112.9, 111.9, 72.64 (t, *J* = 5.3 Hz) , 56.6, 42.3, 23.4; HRMS (EI +) m/z [M^+] Calcd. for $C_{17}H_{17}NOS$, 283.1031; found, 283.1039. *NOTE:* Compound **24j** was found to decompose at rt so was stored under nitrogen at -20 °C and used in subsequent reactions as soon as possible after purification.

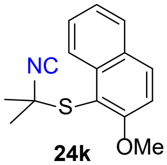

24k

(2-Isocyanopropan-2-yl)(2-methoxynaphthalen-1-yl)sulfane (24k): Alkylation of **x** (300 mg, 1.31 mmol) with methyl iodide (0.18 mL, 2.88 mmol, 2.2 equiv) according to the general alkylation procedure with LDA (2.88 mmol, 2.2 equiv) for 2 h at rt afforded, after purification on silica gel using a Reveleris purification system (12 g cartridge, 0-10% EtOAc:Hexanes), 140 mg (41 %) of **24k** as a clear oil: IR (ATR): 3058, 2980, 2123 cm^{-1}; ^{1}H NMR (400 MHz, $CDCl_3$) δ 8.66 (dd, *J* = 8.4, 1.4 Hz, 1H), 7.97 (d, *J* = 9.1 Hz, 1H),

7.80 (dd, J = 8.4, 1.4 Hz, 1H), 7.57 (ddd, J = 8.4, 6.8, 1.4 Hz, 1H), 7.39 (ddd, J = 8.4, 6.8, 1.4 Hz, 1H), 7.34 (d, J = 9.1 Hz, 1H), 4.03 (s, 3H), 1.72 (s, 6H); ^{13}C {1H} NMR (101 MHz, $CDCl_3$) δ 160.4, 155.98 (t, J = 3.5 Hz), 137.5, 133.3, 129.4, 128.3, 127.7, 126.0, 124.2, 112.8, 111.1, 64.07 (t, J = 5.0 Hz), 56.6, 31.1; HRMS (ES +) m/z [M+H$^+$] Calcd. for $C_{15}H_{16}NOS$, 258.0953; found, 258.0960. *NOTE:* Compound **24k** was found to decompose at rt so was stored under nitrogen at -20 °C and used in subsequent reactions as soon as possible after purification.

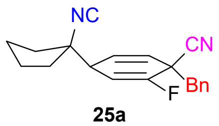

25a

1-Benzyl-2-fluoro-4-(1-isocyanocyclopentyl)cyclohexa-2,5-diene-1-carbonitrile (25a): The general exchange-dearomatization with TMEDA-LiCl was employed with **24a** (30 mg, 0.14 mmol), TMEDA (22 µL, 0.15 mmol), LiCl in THF (0.35 mL, 0.79 M, 0.27 mmol), BuLi (0.1 mL, 1.49 M, 0.15 mmol), and benzyl bromide (18 µL, 0.15 mmol) to afford, after purification on silica gel using a Reveleris purification system (12 g cartridge, 0-15% EtOAc:hexanes), 17 mg (82 %) of **25a** as a colorless oil: IR (ATR): 3033, 2963, 2240, 2127 cm^{-1}; ^1H NMR (500 MHz, $CDCl_3$) δ 7.33 – 7.27 (m, 3H), 7.19 – 7.15 (m, 2H), 5.90 (ddt, J = 10.0, 3.6, 1.8 Hz, 1H), 5.77 (ddd, J = 10.0, 9.0, 1.8 Hz, 1H), 5.51 (ddd, J = 16.5, 3.6, 1.8 Hz, 1H), 3.26 (ABq, Δν = 16.5 Hz, J = 13.4 Hz, 2H), 2.87 (dddd, J = 5.1, 3.6, 1.8, 1.8 Hz, 1H), 2.01 – 1.80 (m, 4H), 1.77 – 1.59 (m, 4H); ^{13}C {1H} NMR (101 MHz, $CDCl_3$) δ 156.7 (t, J = 4.3 Hz), 154.9 (d, J = 262.3 Hz), 132.9, 130.7, 128.5, 128.0, 127.3 (d, J = 2.3 Hz), 126.1 (d, J = 4.1 Hz), 118.3 (d, J = 2.3 Hz), 104.6 (d, J = 16.7 Hz), 70.8 (td, J = 5.4, 2.3 Hz), 44.4 (d, J = 6.3 Hz), 43.1, 40.7 (d, J = 23.3 Hz), 37.8, 37.6, 23.8, 23.7; HRMS (+APCI) m/z [M+H$^+$] Calcd. for $C_{20}H_{20}N_2F$, 307.1605; found, 307.1604.

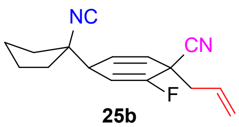

1-Allyl-2-fluoro-4-(1-isocyanocyclopentyl)cyclohexa-2,5-diene-1-carbonitrile (25b): The general exchange-dearomatization with TMEDA-LiCl was employed with **24a** (67 mg, 0.3 mmol), TMEDA (50 µL, 0.33 mmol), LiCl in THF (0.77 mL, 0.79 M), BuLi (0.21 mL, 1.6 M, 0.33 mmol), and allyl bromide (28 µL, 0.33 mmol) to afford, after purification on silica gel using a Reveleris purification system (12 g cartridge, 0-15% EtOAc:hexanes), 21 mg (54 %) of **25b** as a clear oil: IR (ATR): 3082, 2954, 2237, 2128 cm^{-1}; ^{1}H NMR (400 MHz, $CDCl_3$) δ 6.02 (ddt, *J* = 9.9, 3.8, 2.3, 1.6 Hz, 1H), 5.82 (ddd, *J* = 9.9, 8.8, 1.6 Hz, 1H), 5.75 – 5.60 (m, 1H), 5.60 (ddd, *J* = 16.5, 3.8, 1.6 Hz, 1H), 5.29 – 5.17 (m, 2H), 3.29 – 3.20 (m, 1H), 2.78 – 2.62 (m, 2H), 2.08 – 1.85 (m, 4H), 1.83 – 1.67 (m, 4H); ^{13}C {^{1}H} (101 MHz, $CDCl_3$) δ 156.8 (t, *J* = 4.2 Hz), 155.2 (d, *J* = 263.4 Hz), 129.6, 127.2 (d, *J* = 2.3 Hz), 126.3 (d, *J* = 4.1 Hz), 121.6, 118.1 (d, *J* = 2.7 Hz), 104.1 (d, *J* = 16.7 Hz), 71.0 (td, *J* = 5.5, 2.5 Hz), 44.7 (d, *J* = 6.3 Hz), 41.3, 37.7 (d, *J* = 17.2 Hz), 23.8, 23.7; HRMS (+APCI) m/z [M-NC^{+}] Calcd. for $C_{15}H_{17}NF$, 230.1340; found, 230.1337.

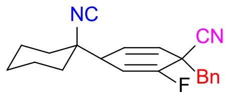

4-Benzyl-3-fluoro-1'-isocyano-[1,1'-bi(cyclohexane)]-2,5-diene-4-carbonitrile (25c): The general exchange-dearomatization with TMEDA-LiCl was employed with **24b** (40 mg, 0.17 mmol), TMEDA (28 µL, 0.19 mmol), LiCl in THF (0.44 mL, 0.78 M, 0.19 mmol), BuLi (0.2 mL, 1.34 M, 0.2 mmol), and benzyl bromide (32 µL, 0.27 mmol) to afford, after purification on silica gel using a Reveleris purification system (12 g cartridge, 0-15% EtOAc:hexanes), 15 mg (56 %) of **25c** as a colorless oil: IR (ATR): 3031, 2936, 2239, 2128 cm^{-1}; 1H NMR (500 MHz, $CDCl_3$) δ 7.32 – 7.27 (m, 3H), 7.20 – 7.14 (m, 2H), 5.94 (ddt, *J* = 10.0, 3.7, 1.9 Hz, 1H), 5.77 (ddd, *J* = 10.0, 8.8, 1.9 Hz, 1H), 5.54 (ddd, *J* = 16.8, 3.7, 1.9 Hz, 1H), 3.26 (ABq, Δν = 11.8 Hz, *J* = 13.5 Hz, 2H), 2.87 – 2.83 (m, 1H), 1.77 – 1.58 (m, 7H), 1.40 – 1.27 (m,

2H), 1.14 – 1.01 (m, 1H); ^{13}C {1H} NMR (101 MHz, CDCl$_3$) δ 157.3, 154.7 (d, *J* = 262.1 Hz), 132.9, 130.7, 128.5, 128.0 126.9 (d, *J* = 2.3 Hz), 126.2 (d, *J* = 3.8 Hz), 118.3 (d, *J* = 2.7 Hz), 104.1 (d, *J* = 16.8 Hz), 64.8, 46.2 (d, *J* = 6.2 Hz), 42.8, 40.7 (d, *J* = 23.4 Hz), 33.5, 33.2, 24.9, 21.60, 21.58; HRMS (+APCI) m/z [M-NC$^+$] Calcd. for $C_{20}H_{21}NF$, 294.1652; found, 294.1657.

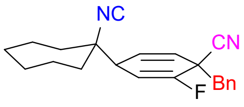

25d

1-Benzyl-2-fluoro-4-(1-isocyanocycloheptyl)cyclohexa-2,5-diene-1-carbonitrile (25d): The general exchange-dearomatization with TMEDA-LiCl was employed with **24c** (50 mg, 0.2 mmol), TMEDA (45 µL, 0.3 mmol), LiCl in THF (0.51 mL, 0.79 M, 0.4 mmol), BuLi (0.21 mL, 1.45M, 0.3 mmol), and benzyl bromide (36 µL, 0.3 mmol) to afford, after purification on silica gel using a Reveleris purification system (12 g cartridge, 0-15% EtOAc:hexanes), 29 mg (88 %) of **25d** as a white solid: mp: 41-43 °C; IR (ATR): 3032, 2929, 2239, 2126 cm^{-1}; ^{1}H NMR (500 MHz, CDCl$_3$) δ 7.32 – 7.27 (m, 3H), 7.19 – 7.14 (m, 2H), 5.94 (ddt, *J* = 10.0, 3.7, 1.9 Hz, 1H), 5.76 (td, *J* = 10.0, 8.8, 1.9 Hz, 1H), 5.54 (ddd, *J* = 16.8, 3.7, 1.9 Hz, 1H), 3.25 (ABq, Δν = 10.5 Hz, *J* = 13.5 Hz, 2H), 2.90 – 2.84 (m, 1H), 1.79 (dt, *J* = 14.4, 7.2 Hz, 2H), 1.75 – 1.55 (m, 8H), 1.54 – 1.42 (m, 2H); ^{13}C {1H} NMR (101 MHz, CDCl$_3$) δ 156.8 (t, *J* = 4.3 Hz), 154.8 (d, *J* = 262.3 Hz), 132.8, 130.7, 128.5, 128.0, 127.3 (d, *J* = 2.3 Hz), 126.1 (d, *J* = 4.1 Hz), 118.3 (d, *J* = 2.3 Hz), 104.5 (d, *J* = 16.7 Hz), 67.4 (td, *J* = 5.2, 2.3 Hz), 48.1 (d, *J* = 6.3 Hz), 42.9, 40.7 (d, *J* = 23.3 Hz), 37.18, 36.99, 27.4, 22.64, 22.58; HRMS (+APCI) m/z [M-NC$^+$] Calcd. for $C_{21}H_{23}NF$, 308.1809; found, 308.1813.

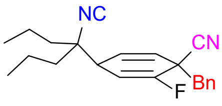

25e

1-Benzyl-2-fluoro-4-(4-isocyanoheptan-4-yl)cyclohexa-2,5-diene-1-carbonitrile (25e): The general exchange-dearomatization with TMEDA-LiCl was

employed with **24f** (50 mg, 0.20 mmol), TMEDA (33 μL, 0.22 mmol), LiCl in THF (0.5 mL, 0.79 M, 0.40 mmol), BuLi (0.14 mL, 1.6 M, 0.22 mmol), and benzyl bromide (26 μL, 0.22 mmol) to afford, after purification on silica gel using a Reveleris purification system (12 g cartridge, 0-15% EtOAc:hexanes), 28 mg (84 %) of **25e** as a colorless oil: IR (ATR): 3032, 2962, 2240, 2126 cm^{-1}; ^{1}H NMR (400 MHz, $CDCl_3$) δ 7.33 – 7.26 (m, 3H), 7.20 – 7.13 (m, 2H), 5.86 (ddt, *J* = 10.0, 3.5, 2.3 Hz, 1H), 5.75 (ddd, *J* = 10.0, 8.7, 1.9 Hz, 1H), 5.43 (ddd, *J* = 16.6, 3.5, 1.9 Hz, 1H), 3.25 (ABq, Δν = 5.6 Hz, *J* = 14.3 Hz, 2H), 3.00 – 2.92 (m, 1H), 1.58 – 1.27 (m, 8H), 0.98 – 0.89 (m, 6H); ^{13}C {^{1}H} NMR (101 MHz, $CDCl_3$) δ 156.8 (t, *J* = 4.2 Hz), 154.7 (d, *J* = 262.0 Hz), 132.9, 130.7, 128.5, 128.0, 127.2 (d, *J* = 1.9 Hz), 126.1 (d, *J* = 4.0 Hz), 118.1 (d, *J* = 2.3 Hz), 104.2 (d, *J* = 16.9 Hz), 66.1 (td, *J* = 5.1, 2.3 Hz), 43.0 (d, *J* = 6.1 Hz), 42.9, 40.8 (d, *J* = 23.3 Hz), 38.16, 38.14, 17.01, 16.95, 14.19, 14.17; HRMS (+APCI) m/z [M-NC^+] Calcd. for $C_{21}H_{25}NF$, 310.1965; found, 310.1971.

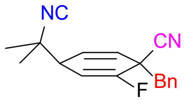

25f

1-Benzyl-2-fluoro-4-(2-isocyanopropan-2-yl)cyclohexa-2,5-diene-1-carbonitrile (25f): The general exchange-dearomatization with TMEDA-LiCl was employed with **24e** (50 mg, 0.26 mmol), TMEDA (42 μL, 0.28 mmol), LiCl in THF (0.65 mL, 0.79 M, 0.51 mmol), BuLi (0.18 mL, 1.6M, 0.28 mmol), and benzyl bromide (33 μL, 0.28 mmol) to afford, after purification on silica gel using a Reveleris purification system (12 g cartridge, 0-15% EtOAc:hexanes), 27 mg (75 %) of **25f** as a colorless oil: IR (ATR): 3033, 2988, 2240, 2130 cm^{-1}; ^{1}H NMR (500 MHz, $CDCl_3$) δ 7.32 – 7.28 (m, 3H), 7.19 – 7.15 (m, 2H), 5.91 (ddt, *J* = 10.2, 3.6, 1.8 Hz, 1H), 5.79 (ddd, *J* = 10.2, 8.8, 1.8 Hz, 1H), 5.51 (ddd, *J* = 16.6, 3.6, 1.8 Hz, 1H), 3.26 (ABq, Δν = 12.1 Hz, *J* = 13.5 Hz, 2H), 2.89 – 2.83 (m, 1H), 1.38 – 1.33 (m, 6H); ^{13}C {^{1}H} NMR (101 MHz, $CDCl_3$) δ 156.0 (t, *J* = 4.1 Hz), 155.0 (d, *J* = 262.4 Hz), 132.8, 130.7, 128.6, 128.0,

126.8 (d, J = 2.3 Hz), 126.4 (d, J = 3.9 Hz), 118.2 (d, J = 2.3 Hz), 104.1 (d, J = 16.9 Hz), 59.8 (td, J = 5.5, 2.3 Hz), 46.4 (d, J = 6.3 Hz), 42.9, 40.7 (d, J = 23.1 Hz), 26.4, 26.2; HRMS (+APCI) m/z [M+H$^+$] Calcd. for $C_{18}H_{18}N_2F$, 281.1448; found, 281.1451.

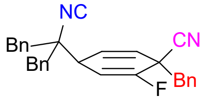

25g

1-Benzyl-2-fluoro-4-(2-isocyano-1,3-diphenylpropan-2-yl)cyclohexa-2,5-diene-1-carbonitrile (25g): The general exchange-dearomatization with TMEDA-LiCl was employed with **24g** (50 mg, 0.14 mmol), TMEDA (24 µL, 0.16 mmol), LiCl in THF (0.37 mL, 0.79 M, 0.29 mmol), BuLi (0.1 mL, 1.6 M, 0.16 mmol), and benzyl bromide (19 µL, 0.16 mmol) to afford, after purification on silica gel using a Reveleris purification system (12 g cartridge, 0-15% EtOAc:hexanes), 23 mg (74 %) of **25g** as a white solid: mp: 141-143 °C; IR (ATR): 3064, 2960, 2241, 2129 cm^{-1}; ^{1}H NMR (400 MHz, $CDCl_3$) δ 7.38 –7.12 (m, 15H), 6.04 (dq, J = 10.1, 3.4, 2.1 Hz, 1H), 5.81 (td, J = 10.1, 9.0, 2.1 Hz, 1H), 5.65 (dt, J = 16.6, 3.4, 2.1 Hz, 1H), 3.24 (ABq, Δν = 31.4 Hz, J = 13.4 Hz, 2H), 2.88 – 2.68 (m, 5H); 13C {1H} NMR (101 MHz, $CDCl_3$) δ 160.0, 155.2 (d, J = 262.8 Hz), 134.0 (d, J = 6.2 Hz), 132.7, 130.8, 130.7, 130.6, 128.71, 128.69, 128.5, 128.1, 127.86, 127.84, 126.9 (d, J = 2.3 Hz), 126.5 (d, J = 4.0 Hz), 118.0 (d, J = 2.3 Hz), 103.9 (d, J = 17.3 Hz), 66.8, 43.1, 41.8 (d, J = 6.1 Hz), 41.6, 41.4, 41.0. 40.8; HRMS (+APCI) m/z [M-NC$^+$] Calcd. for $C_{29}H_{25}NF$, 406.1965; found, 406.1973.

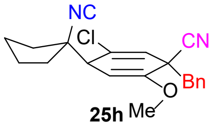

25h

1-Benzyl-5-chloro-4-(1-isocyanocyclopentyl)-2-methoxycyclohexa-2,5-diene-1-carbonitrile (25h): The general exchange-dearomatization with HMTETA was employed with **24h** (80 mg, 0.3 mmol), HMTETA (90 µL, 0.33 mmol), BuLi (0.21 mL, 1.6 M, 0.33 mmol), and benzyl bromide (39 µL, 0.33 mmol) to afford, after purification on

silica gel using a Reveleris purification system (12 g cartridge, 0-10% EtOAc:hexanes), 36 mg (69 %) of **25h** as a colorless oil: IR (ATR): 3081, 2968, 2239, 2126 cm^{-1}; ^{1}H NMR (400 MHz, $CDCl_3$) δ 7.33 – 7.24 (m, 3H), 7.14 – 7.11 (m, 2H), 5.95 (br. d, *J* = 0.5 Hz, 1H), 4.80 (d, *J* = 4.5 Hz, 1H), 3.72 (s, 3H), 3.28 (ABq, Δν = 19.6 Hz, *J* = 13.3 Hz, 2H), 3.00 – 2.94 (m, 1H), 2.32 – 2.17 (m, 1H), 2.16 – 2.02 (m, 1H), 2.02 – 1.86 (m, 2H), 1.86 – 1.68 (m, 4H); ^{13}C {^{1}H} NMR (101 MHz, $CDCl_3$) δ 157.8 (t, *J* = 4.0 Hz), 151.0, 133.5, 133.4, 130.6, 128.4, 127.8, 125.8, 119.2, 95.9, 72.5 (t, *J* = 5.4 Hz), 55.6, 49.7, 44.1, 43.1, 39.4, 39.2, 23.2, 22.0; HRMS (+APCI) m/z [$M+H^+$] Calcd. for $C_{21}H_{22}ON_2Cl$, 353.1415; found, 353.1421.

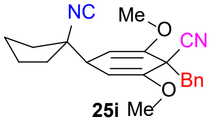

1-Benzyl-4-(1-isocyanocyclopentyl)-2,6-dimethoxycyclohexa-2,5-diene-1-carbonitrile (25i): The general exchange-dearomatization with HMTETA was employed with **24i** (80 mg, 0.3 mmol), HMTETA (91 μL, 0.33 mmol), BuLi (0.21 mL, 1.6 M, 0.33 mmol), and benzyl bromide (40 μL, 0.33 mmol) to afford, after purification on silica gel using a Reveleris purification system (12 g cartridge, 0-10% EtOAc:hexanes), 43 mg (81 %) of **25i** as a colorless oil: IR (ATR): 3032, 2959, 2241, 2127 cm^{-1}; ^{1}H NMR (400 MHz, $CDCl_3$) δ 7.23 – 7.15 (m, 3H), 6.97 – 6.90 (m, 2H), 4.74 (d, *J* = 3.5 Hz, 2H), 3.71 (s, 6H), 3.45 (s, 2H), 2.63 (tt, *J* = 3.5, 1.5 Hz, 1H), 1.93 – 1.76 (m, 4H), 1.72 – 1.59 (m, 4H); ^{13}C {^{1}H} NMR (101 MHz, $CDCl_3$) δ 155.1 (t, *J* = 4.3 Hz), 150.0, 134.1, 130.2, 128.0, 127.4, 119.8, 95.4, 72.0 (t, *J* = 5.0 Hz), 55.3, 45.2, 42.2, 40.2, 37.5, 24.2; HRMS (+APCI) m/z [$M+H^+$] Calcd. for $C_{22}H_{25}N_2O_2$, 349.1910; found, 349.1911.

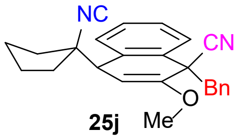

((1-Benzyl-4-(1-isocyanocyclopentyl)-2-methoxy-1,4-dihydro-naphthalen-1-yl)-l3-methylene)-l2-azane (25j): The general exchange-

dearomatization with HMTETA was employed with **24j** (85 mg, 0.3 mmol), HMTETA (90 µL, 0.33 mmol), BuLi (0.21 mL, 1.6 M, 0.33 mmol), and benzyl bromide (40 µL, 0.33 mmol) to afford, after purification on silica gel using a Reveleris purification system (12 g cartridge, 0-10% EtOAc:hexanes), 43 mg (78 %) of **25j** as a colorless oil: IR (ATR): 3063, 3032, 2960, 2878, 2237, 2126 cm^{-1}; ^{1}H NMR (400 MHz, $CDCl_3$) δ 7.78 (br d, J = 8.1 Hz, 1H), 7.48 (br t, J = 8.1 Hz, 1H), 7.35-7.29 (m, 1H), 7.28-7.24 (m, 1H), 7.06 (ddt, J = 8.5, 6.2, 1.8, 0.9 Hz, 1H), 7.01 – 6.91 (m, 2H), 6.41 – 6.37 (m, 2H), 4.97 (d, J = 4.8 Hz, 1H), 3.76 (s, 3H), 3.60 (ABq, Δν = 157.6 Hz, J = 13.0 Hz, 2H), 3.43-3.36 (m, 1H), 1.95 – 1.77 (m, 4H), 1.77 – 1.59 (m, 4H); ^{13}C {^{1}H} (101 MHz, $CDCl_3$) δ 157.02 (t, J = 4.3 Hz), 149.7, 133.7, 133.0, 132.8, 130.0, 129.2, 128.6, 128.2, 127.8, 127.6, 127.2, 121.8, 96.3, 74.57 (t, J = 5.0 Hz), 55.0, 46.6, 45.1, 44.1, 38.0, 37.4, 22.4, 22.3; HRMS (ES+) m/z [M+Na^{+}] Calcd. for $C_{25}H_{24}N_2ONa$, 391.1782; found, 391.1782.

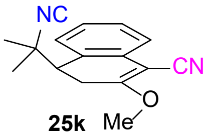

25k

((4-(2-Isocyanopropan-2-yl)-2-methoxy-3,4-dihydronaphthalen-1-yl)-l3-methylene)-l2-azane (25k): The general exchange-dearomatization with HMTETA was employed with **24k** (40 mg, 0.16 mmol), HMTETA (47 µL, 0.17 mmol), BuLi (0.11 mL, 1.6 M, 0.17 mmol), and a saturated, aqueous NH_4Cl solution (1 mL) to afford, after purification on silica gel using a Reveleris purification system (12 g cartridge, 0-15% EtOAc:hexanes), 15 mg (78 %) of **25k** as a colorless oil: IR (ATR): 2988, 2946, 2216, 2129, 1619 cm^{-1}; ^{1}H NMR (400 MHz, $CDCl_3$) δ 7.45 (d, J = 7.8 Hz, 1H), 7.34 (ddd, J = 12.0, 7.8, 4.2 Hz, 1H), 7.18 (d, J = 4.2 Hz, 2H), 4.15 (s, 3H), 3.12 (d, J = 18.5 Hz, 1H), 3.09 (d, J = 8.7 Hz, 1H), 2.87 (dd, J = 18.5, 8.7 Hz, 1H), 1.40 (br. t, J = 2.1 Hz, 3H), 1.27 (br. t, J = 2.1 Hz, 3H); ^{13}C {^{1}H} (101 MHz, $CDCl_3$) δ 169.3, 155.83 (t, J = 4.3 Hz), 131.4, 130.8, 129.1, 127.0, 126.4, 124.4, 116.0, 87.9, 61.73

(t, J = 5.0 Hz), 57.7, 46.2, 28.7, 27.7, 25.3; HRMS (ES+) m/z [$M+Na^+$] Calcd. for $C_{16}H_{16}N_2ONa$, 275.1160; found, 275.1154.

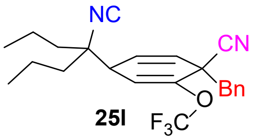

25l

1-Benzyl-4-(4-isocyanoheptan-4-yl)-2-(trifluoromethoxy)-cyclohexa-2,5-diene-1-carbonitrile (25l): A THF solution of **19c** (30 mg, 0.11 mmol) and **24i** (33 mg, 0.11 mmol) was submitted to the general exchange-dearomatization TMEDA-LiCl procedure with TMEDA (38 μL, 0.25 mmol), LiCl in THF (0.58 mL, 0.786 M, 0.46 mmol), BuLi (0.16 mL, 1.6 M, 0.25 mmol), and benzyl bromide (30 μL, 0.25 mmol) to afford, after purification on silica gel using a Reveleris purification system (12 g cartridge, 0-10% EtOAc:hexanes), 23 mg (50 %) of **25l** as a colorless oil: IR (ATR): 3033, 2965, 2936, 2877, 2240, 2125, 1246, 1212, 1172 cm^{-1}; ^1H NMR (400 MHz, $CDCl_3$) δ 7.33 – 7.27 (m, 3H), 7.20 – 7.13 (m, 2H), 5.86 (ddd, J = 10.1, 3.6, 1.8 Hz, 1H), 5.77 (dd, J = 10.1, 1.8 Hz, 1H), 5.65 (dp, J = 3.6, 1.8 Hz, 1H), 3.24 (dd, Δν = 15.8 Hz, J = 13.4 Hz, 2H), 2.93 – 2.90 (m, 1H), 1.53 – 1.28 (m, 8H), 0.94 (br. t, J = 6.9 Hz, 6H); ^{13}C {^1H} (101 MHz, $CDCl_3$) δ 157.1 (t, J = 4.2 Hz), 143.2, 129.6 (q, J = 213 Hz), 128.1, 126.2, 124.4, 120.5 (q, J = 260.7 Hz), 118.1, 110.9, 66.2 (t, J = 5.3 Hz), 43.4, 42.9, 41.6, 38.33, 38.30, 16.99, 16.96, 14.18, 14.16; HRMS (+APCI) m/z [$M-NC^+$] Calcd. for $C_{22}H_{25}ONF_3$, 376.1882; found, 376.1893.

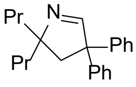

27

4,4-Diphenyl-2,2-dipropyl-3,4-dihydro-2H-pyrrole (27): A THF solution of **19c** (80 mg, 0.3 mmol) and 1,1-diphenylethylene (60 μL, 0.33 mmol) was submitted to the general exchange-dearomatization HMTETA procedure with HMTETA (91 μL, 0.33 mmol), BuLi (0.24 mL, 1.4 M, 0.33 mmol), and benzyl bromide (40 μL, 0.33 mmol) to afford, after

purification on silica gel using a Reveleris purification system (12 g cartridge, 0-10% EtOAc:hexanes), 21 mg (23 %) of **27** as a colorless oil: IR (ATR): 3060, 3024, 2957, 2932, 2871, 1634, 1599 cm^{-1}; ^{1}H NMR (400 MHz, $CDCl_3$) δ 7.78 (s, 1H), 7.35 – 7.26 (m, 4H), 7.26 – 7.12 (m, 6H), 2.43 (s, 2H), 1.54 (ddd, *J* = 13.2, 12.1, 4.8 Hz, 2H), 1.42 (ddd, *J* = 13.2, 12.1, 4.8 Hz, 2H), 1.41 – 1.20 (m, 4H), 0.85 (t, *J* = 7.1 Hz, 6H) ; ^{13}C {^{1}H} NMR (101 MHz, $CDCl_3$) δ 166.1, 146.1, 128.7, 127.4, 126.7, 80.3, 66.6, 46.7, 41.9, 17.8, 14.9; HRMS (+APCI) m/z [$M+H^+$] Calcd. for $C_{22}H_{28}N$, 306.2216; found, 306.2216.

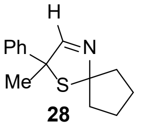

28

2-Methyl-2-phenyl-1-thia-4-azaspiro[4.4]non-3-ene (28): Thiazoline **28** was prepared according to a previously reported literature procedure[8] with sodium hydrogen sulfide hydrate (44 mg, 0.59 mmol), cyclopentanone (0.12 mL, 1.3 mmol), 25% aqueous ammonia solution (0.27 mL, 6.42 M), and 2-chloro-2-phenylpropionaldehyde[9] (100 mg, 0.59 mmol) to afford, after purification on silica gel using a Reveleris purification system (12 g cartridge, 0-15% EtOAc:hexanes), 60 mg (44 %) of **28** as a clear, light-yellow oil: IR (ATR): 3059, 3024, 2965, 1647, 1599 cm^{-1}; ^{1}H NMR (400 MHz, $CDCl_3$) δ 7.40 – 7.32 (m, 4H), 7.28 – 7.23 (m, 1H), 7.08 (s, 1H), 2.34 – 2.19 (m, 2H), 2.15 – 2.03 (m, 2H), 1.99 – 1.87 (m, 2H), 1.94 (s, 3H), 1.87 – 1.75 (m, 2H); ^{13}C {^{1}H} NMR (101 MHz, $CDCl_3$) δ 163.2, 143.4, 128.8, 127.5, 126.6, 98.2, 70.5, 44.2, 43.6, 28.2, 24.8, 24.7; HRMS (+APCI) m/z [$M+H^+$] Calcd. for $C_{14}H_{18}NS$, 232.1154; found, 232.1155.

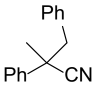

30

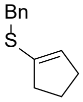

31

2-Methyl-2,3-diphenylpropanenitrile (30) and Benzyl(cyclopent-1-en-1-yl)sulfane (31): A hexanes solution of BuLi (0.3 mL, 1.6 M, 0.48 mmol) was added dropwise to a -78 °C, THF solution (2.2 mL, 0.1 M) of thiazoline **28** (50 mg, 0.22 mmol). After 1 h, neat benzyl bromide (56 μL, 0.48 mmol) was added and then the reaction was allowed

to warm to rt. After 24 h, saturated, aqueous NH_4Cl was added. The mixture was diluted with CH_2Cl_2, the phases were separated, and the aqueous phase extracted with CH_2Cl_2 (2 x 25 mL). The combined organic extracts were dried (Na_2SO_4) and concentrated. The crude material was dry loaded onto Celite and purified by automated flash column chromatography on silica gel using a Reveleris purification system with 0-2% EtOAc:Hexanes as the mobile phase to afford 28 mg (58%) of **30** as a colorless oil exhibiting spectra identical to that of the previously published material[10] and 24 mg (59%) of **31** as a colorless oil: IR (ATR): 2930, 1598 cm^{-1}; ^{1}H NMR (400 MHz, $CDCl_3$) δ 7.39 – 7.27 (m, 4H), 7.27 – 7.20 (m, 1H), 5.44 (p, *J* = 2.1 Hz, 1H), 3.97 (s, 2H), 2.50 – 2.30 (m, 4H), 2.01 – 1.84 (m, 2H); ^{13}C {^{1}H} NMR (101 MHz, $CDCl_3$) δ 137.7, 137.1, 128.9, 128.7, 127.3, 123.6, 37.0, 36.3, 33.0, 23.7; HRMS (+APCI) m/z [$M\text{-}H^{+}$] Calcd. for $C_{12}H_{13}S$, 189.0732; found, 189.0732.

Supplementary Figure 1. ^{1}H NMR of Compound **i** at 25 °C

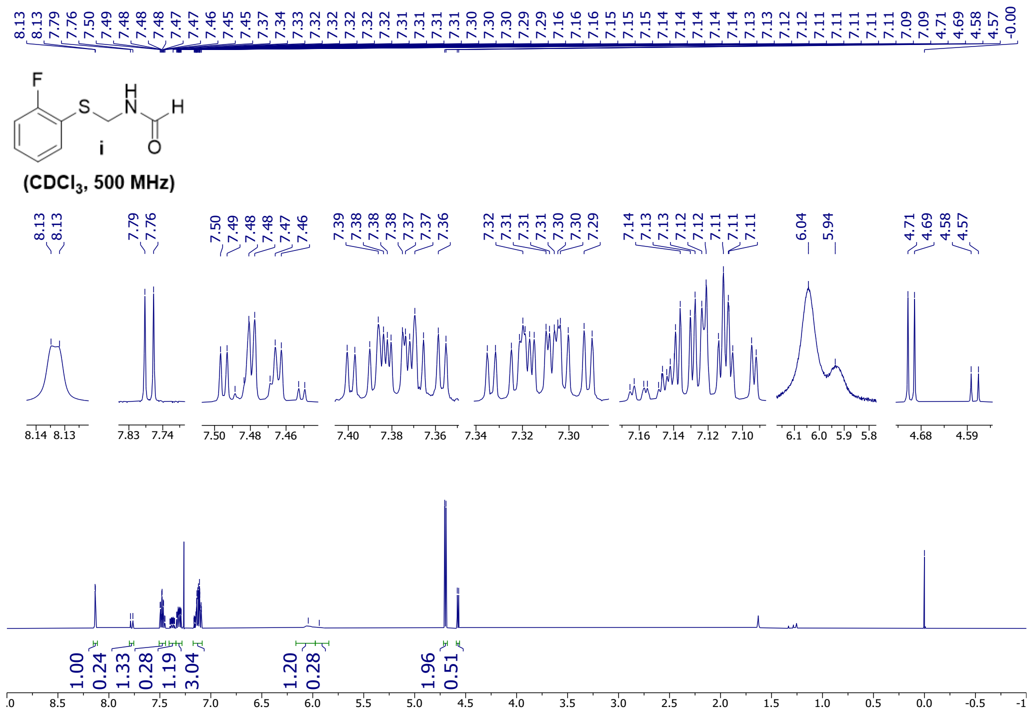

Supplementary Figure 2. ^{13}C NMR of Compound **i** at 25 °C

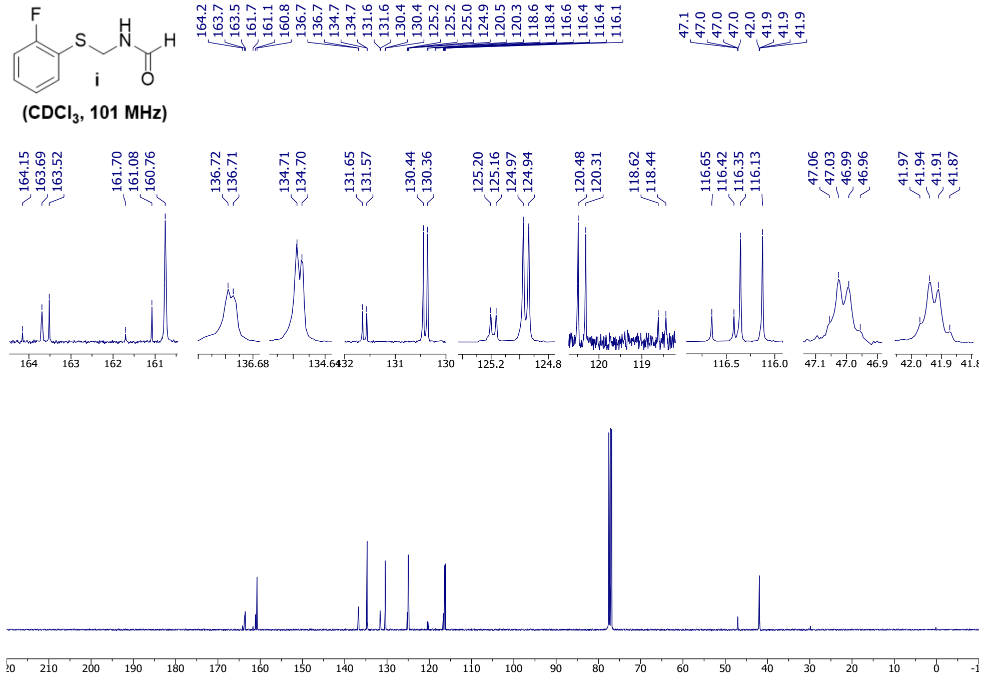

Supplementary Figure 3. ^{1}H NMR of Compound **ii** at 25 °C

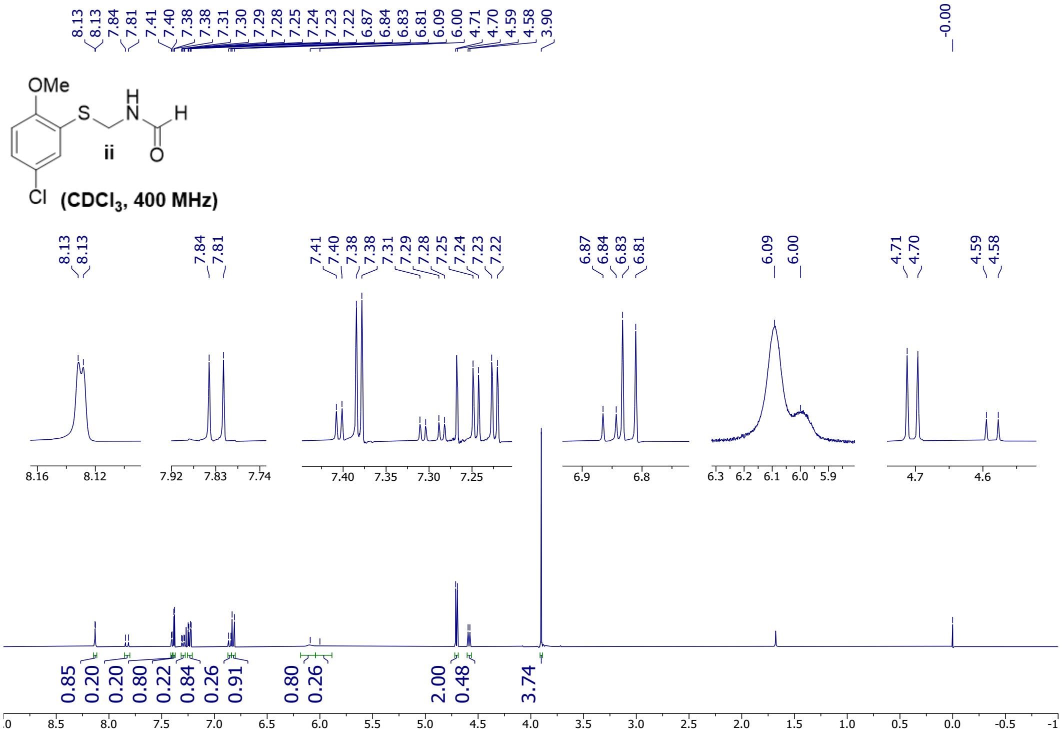

Supplementary Figure 4. ^{13}C NMR of Compound **ii** at 25 °C

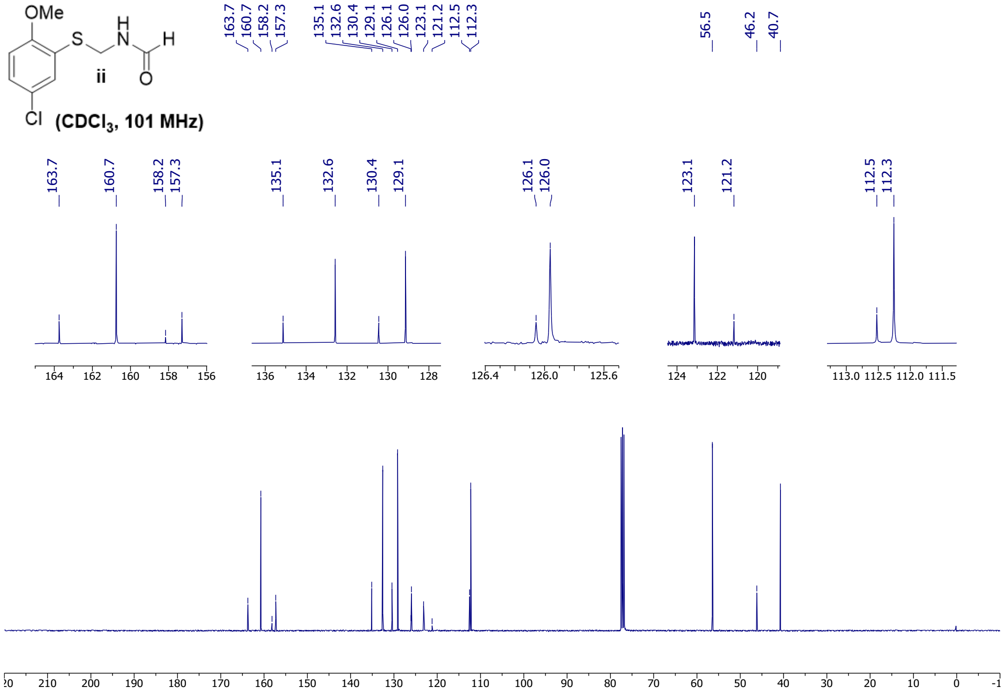

Supplementary Figure 5. ^{1}H NMR of Compound iii at 25 °C

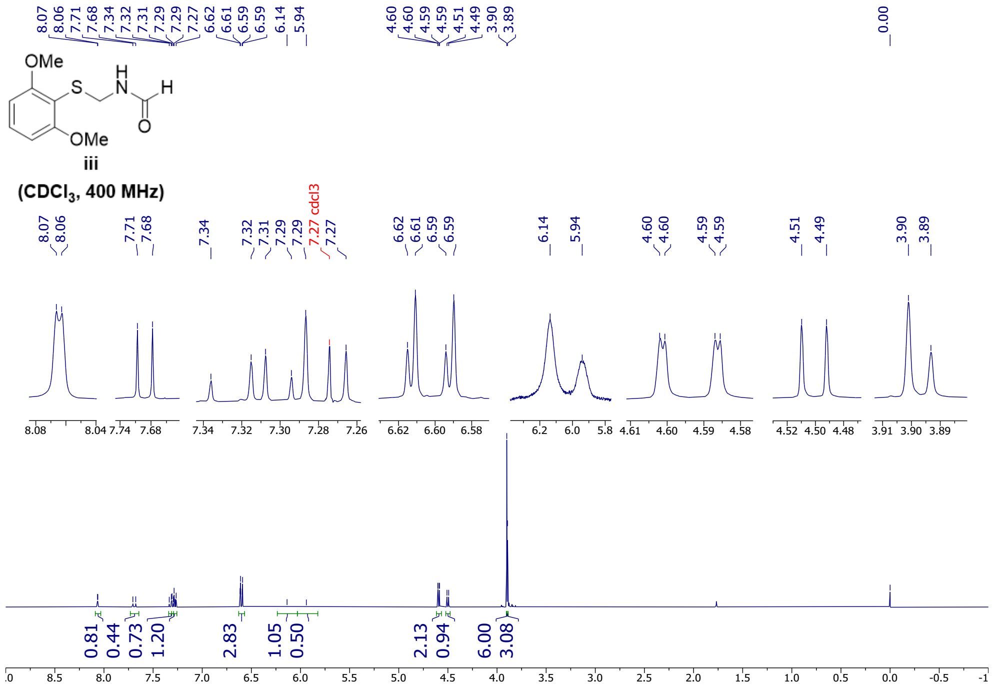

Supplementary Figure 6. ^{13}C NMR of Compound iii at 25 °C

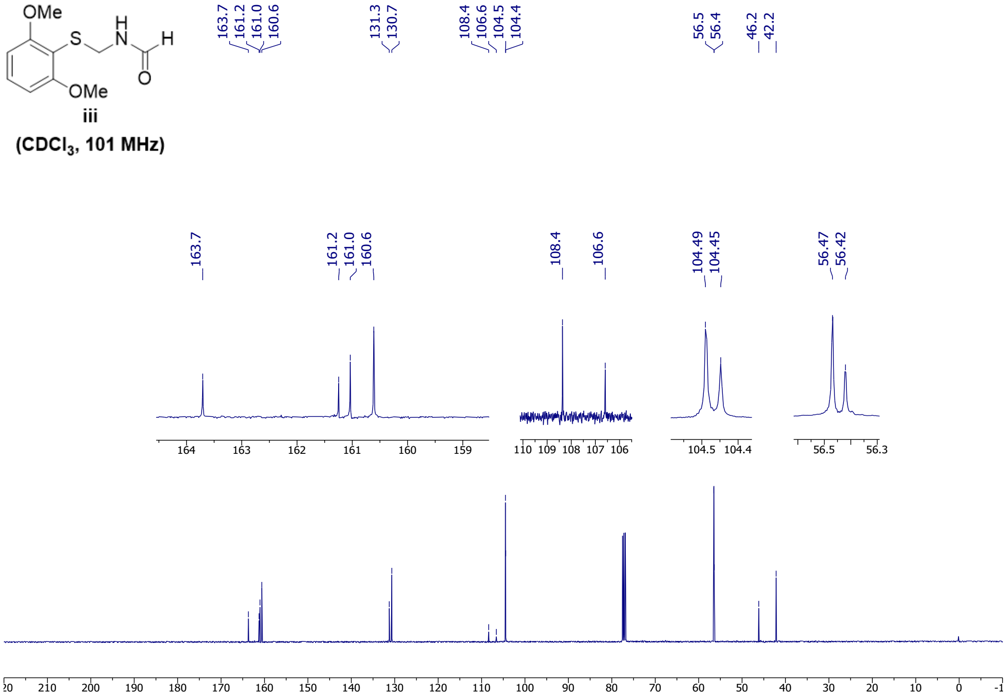

Supplementary Figure 7. ^{1}H NMR of Compound **iv** at 25 °C

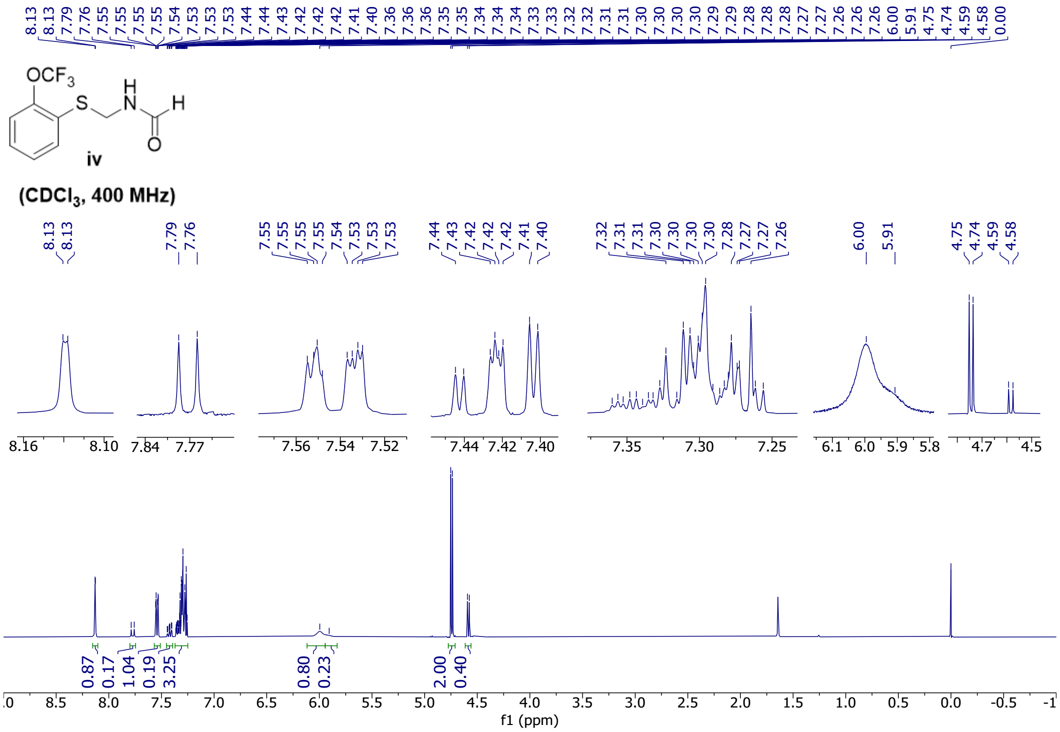

Supplementary Figure 8. ^{13}C NMR of Compound **iv** at 25 °C

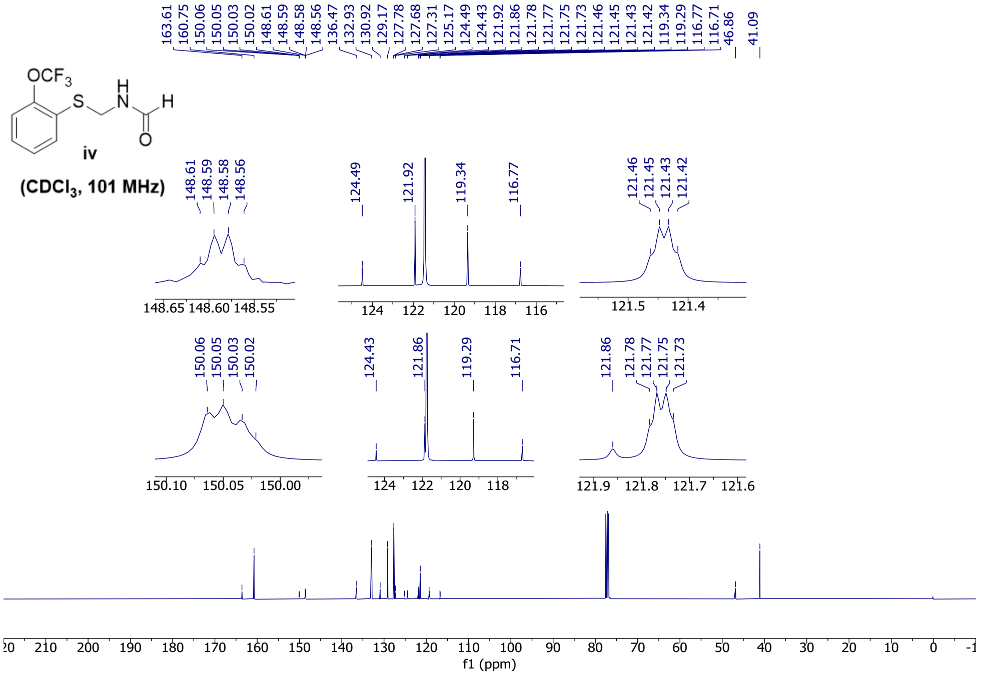

Supplementary Figure 9. ^{1}H NMR of Compound **v** at 25 °C

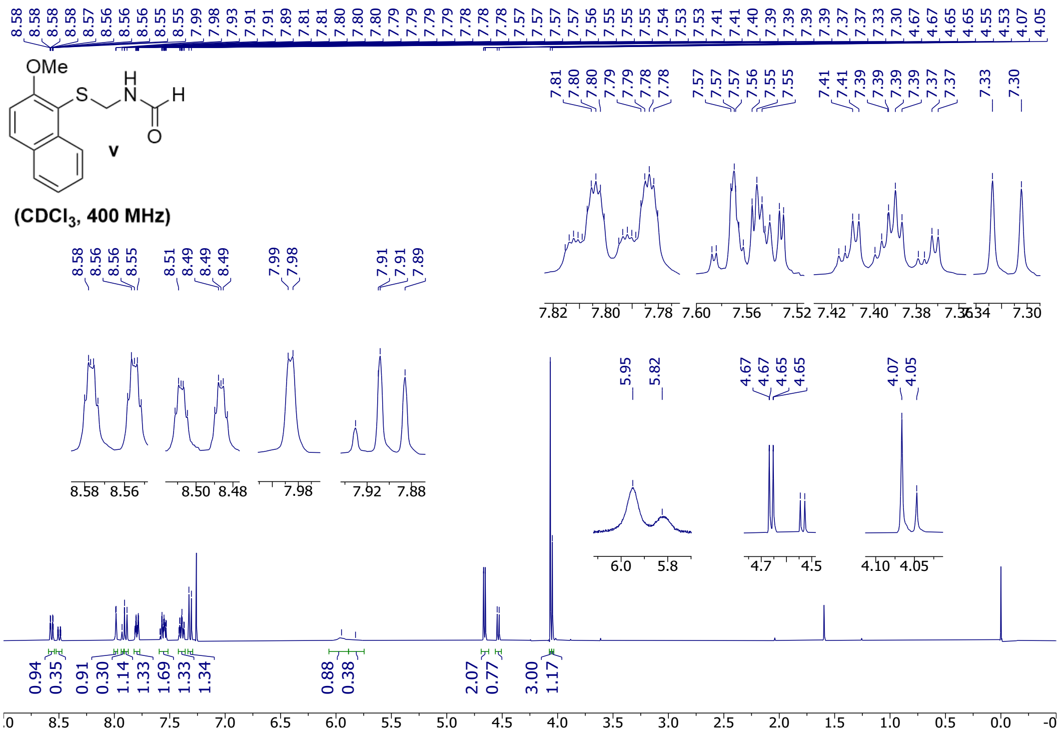

Supplementary Figure 10. ^{13}C NMR of Compound **v** at 25 °C

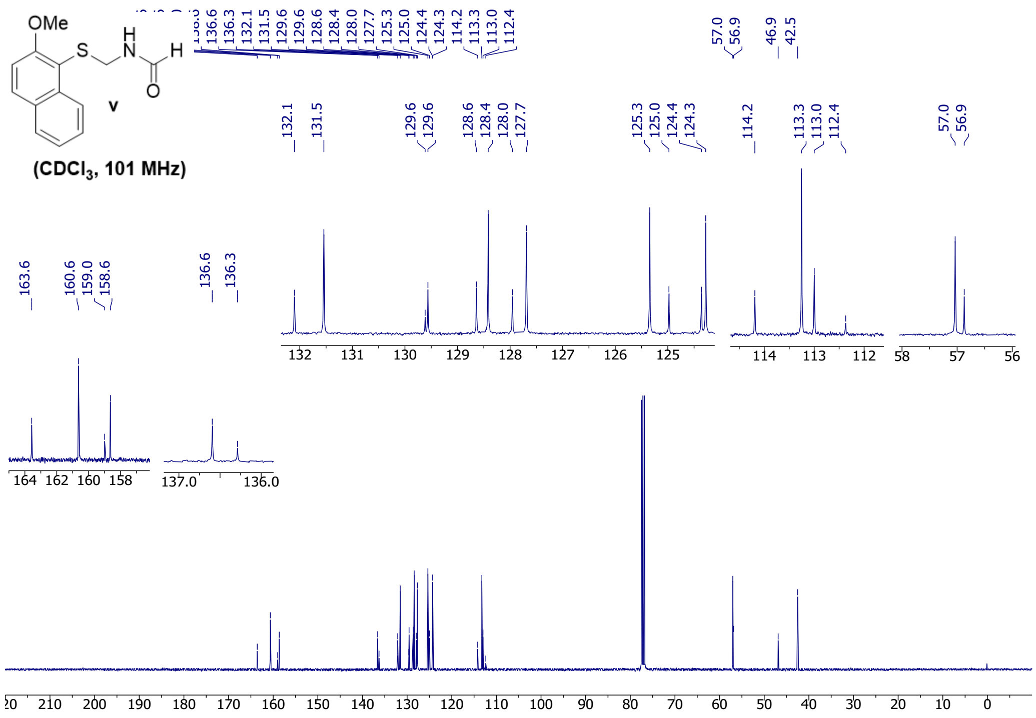

Supplementary Figure 11. ^{1}H NMR of Compound **vi** at 25 °C

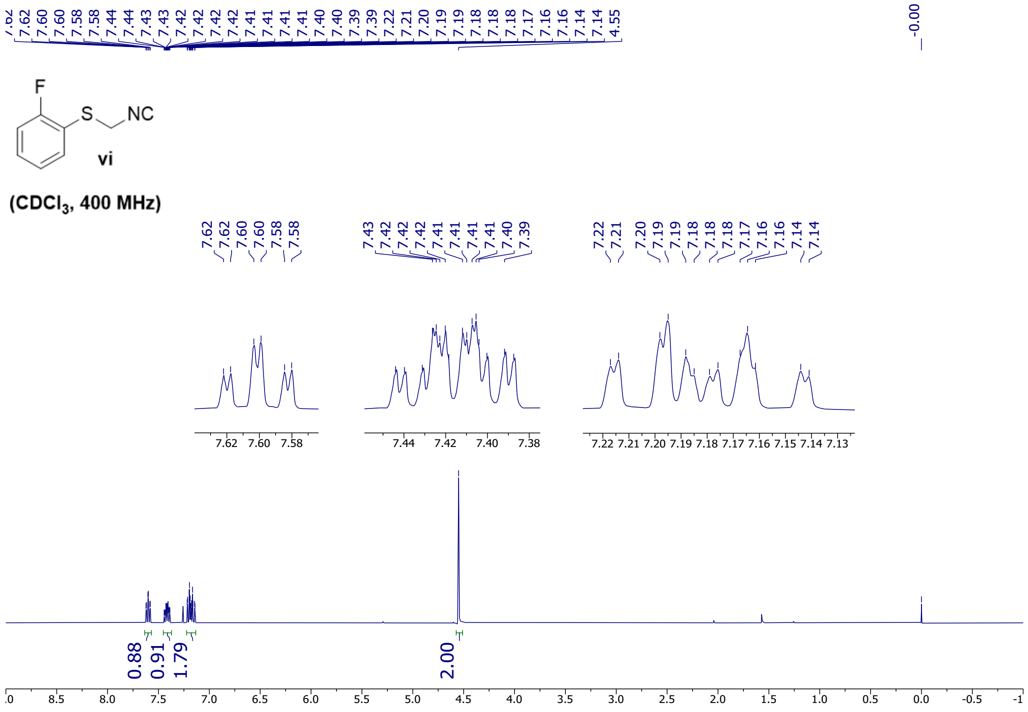

Supplementary Figure 12. ^{13}C NMR of Compound **vi** at 25 °C

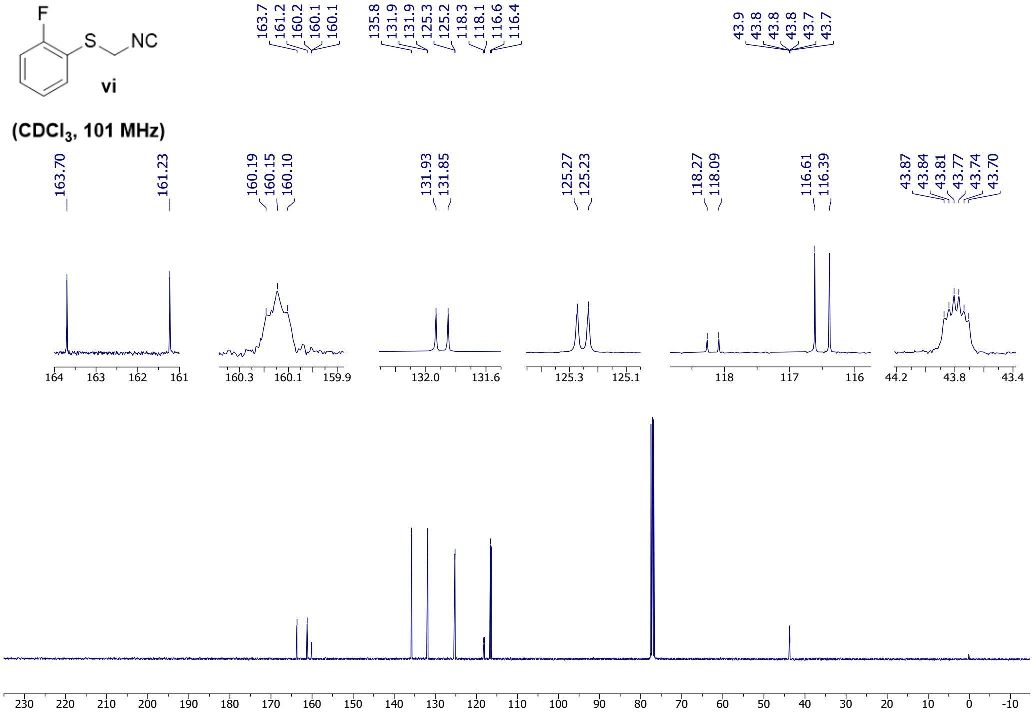

Supplementary Figure 13. ^{1}H NMR of Compound **vii** at 25 °C

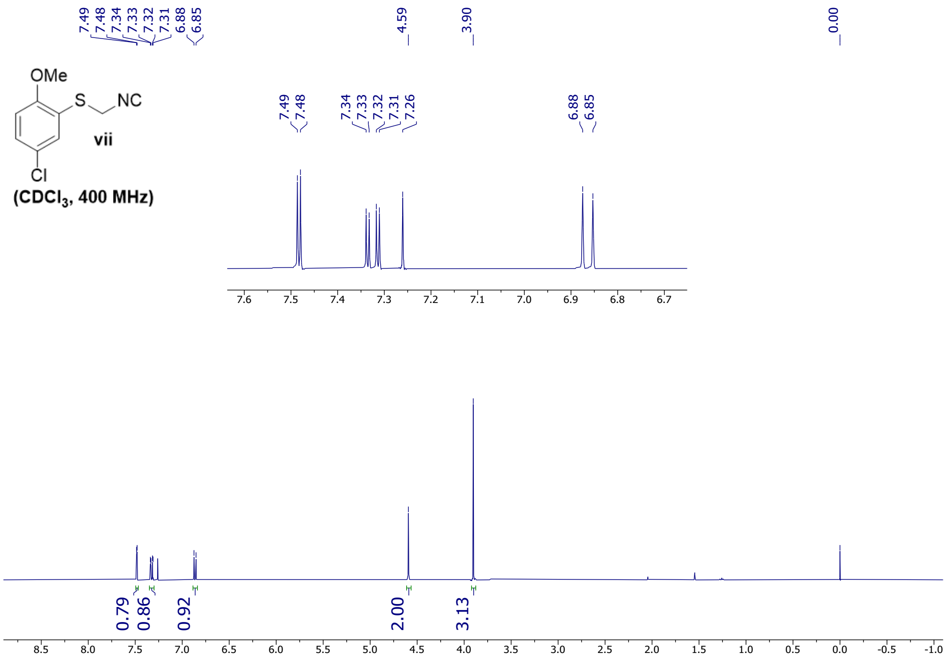

Supplementary Figure 14. ^{13}C NMR of Compound **vii** at 25 °C

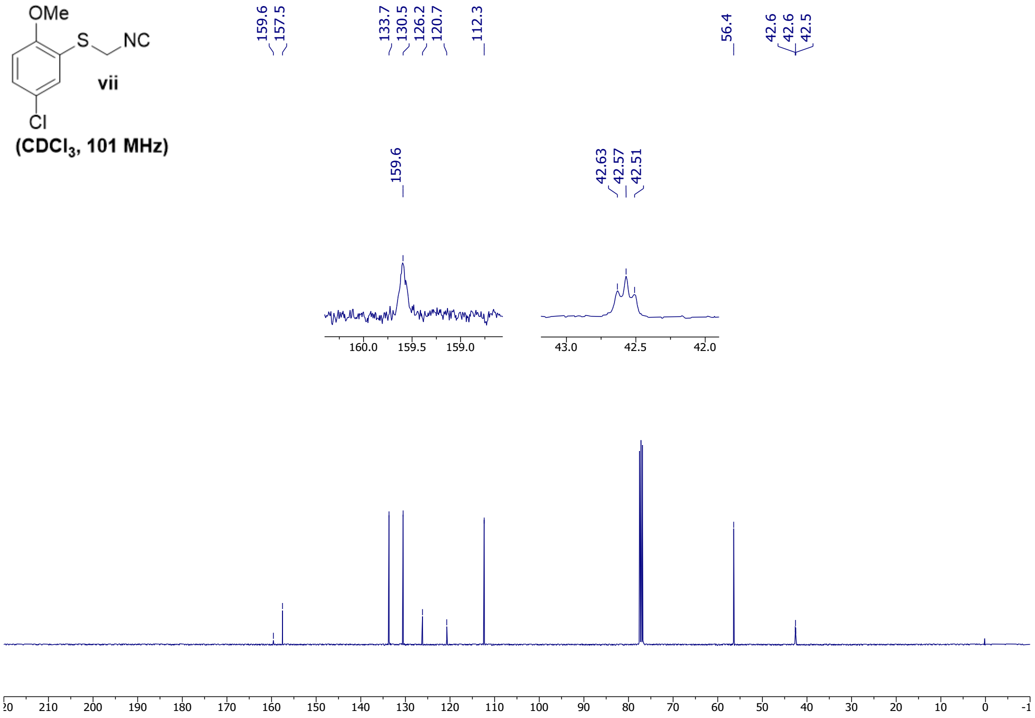

Supplementary Figure 15. ^{1}H NMR of Compound **viii** at 25 °C

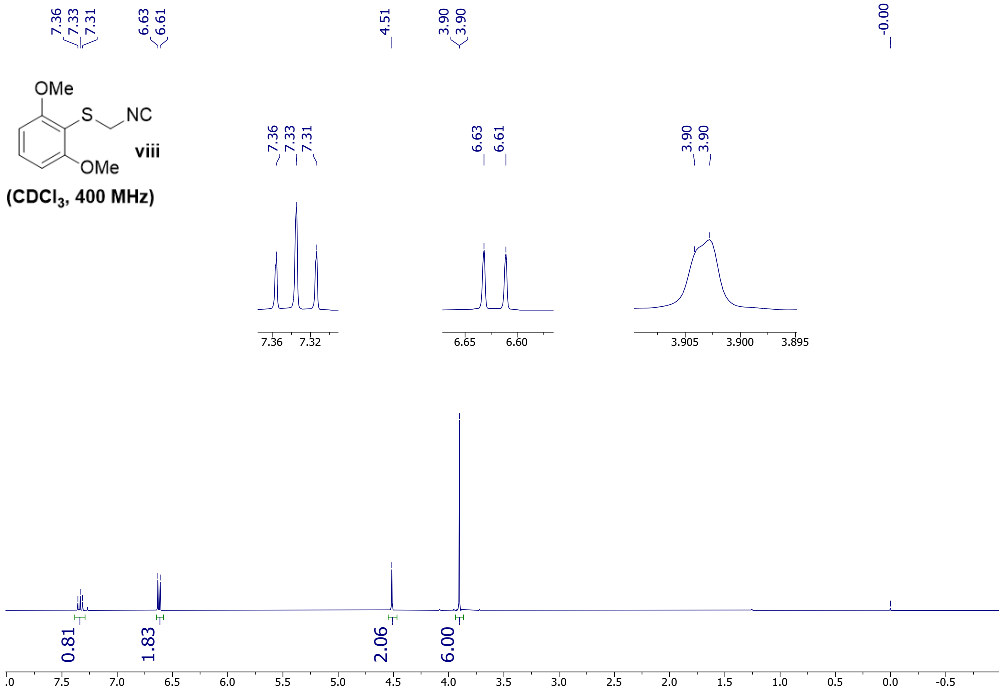

Supplementary Figure 16. ^{13}C NMR of Compound **viii** at 25 °C

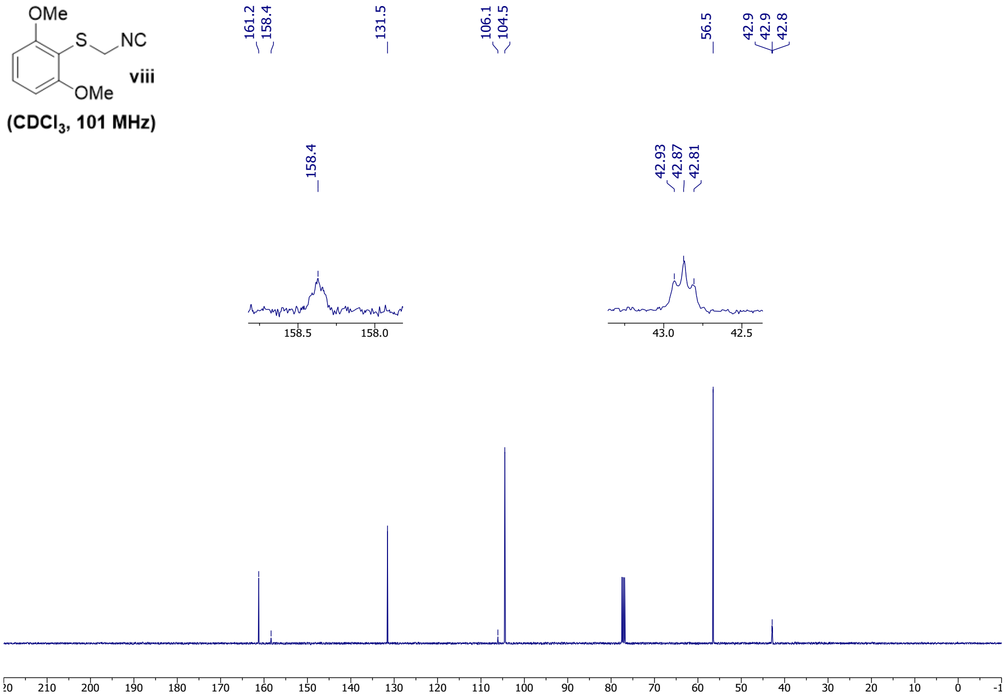

Supplementary Figure 17. ^{1}H NMR of Compound **ix** at 25 °C

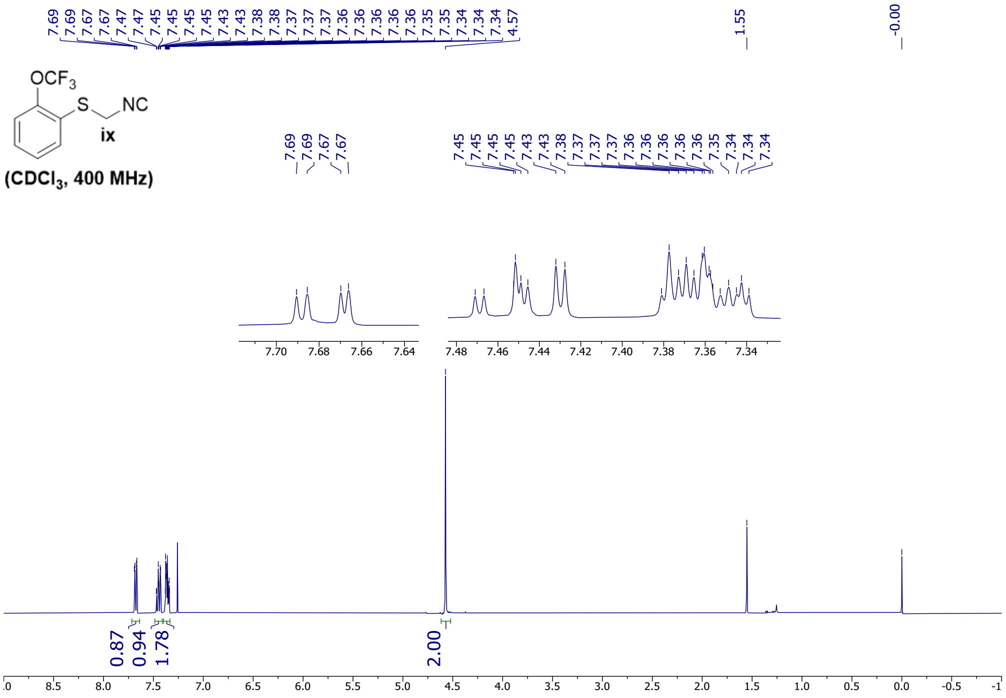

Supplementary Figure 18. ^{13}C NMR of Compound **ix** at 25 °C

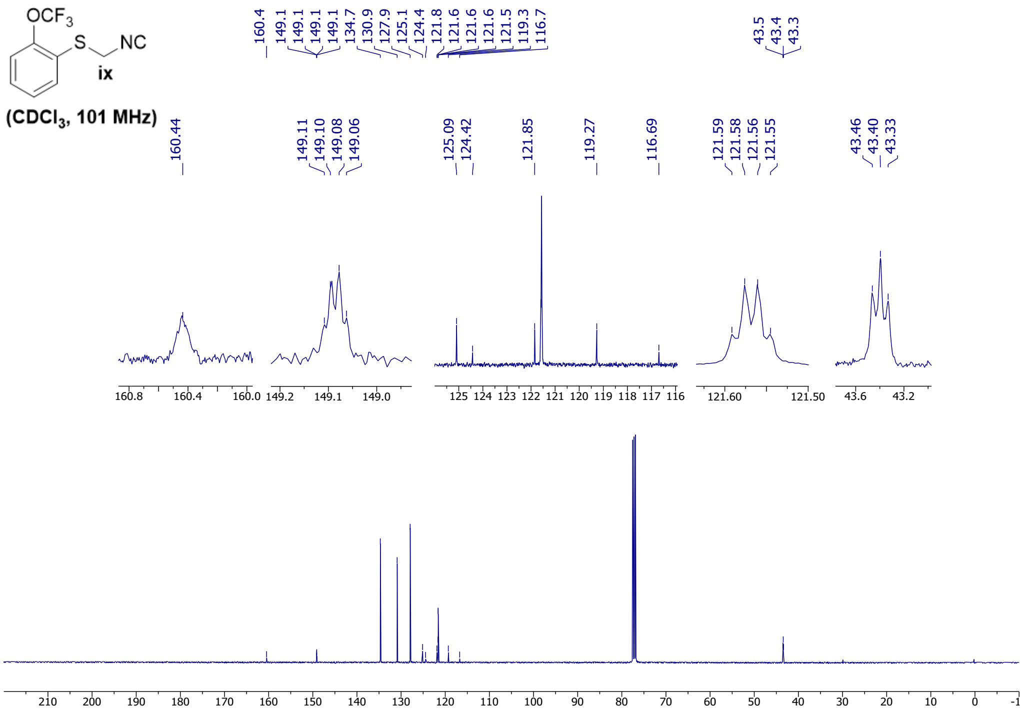

Supplementary Figure 19. ^{1}H NMR of Compound **x** at 25 °C

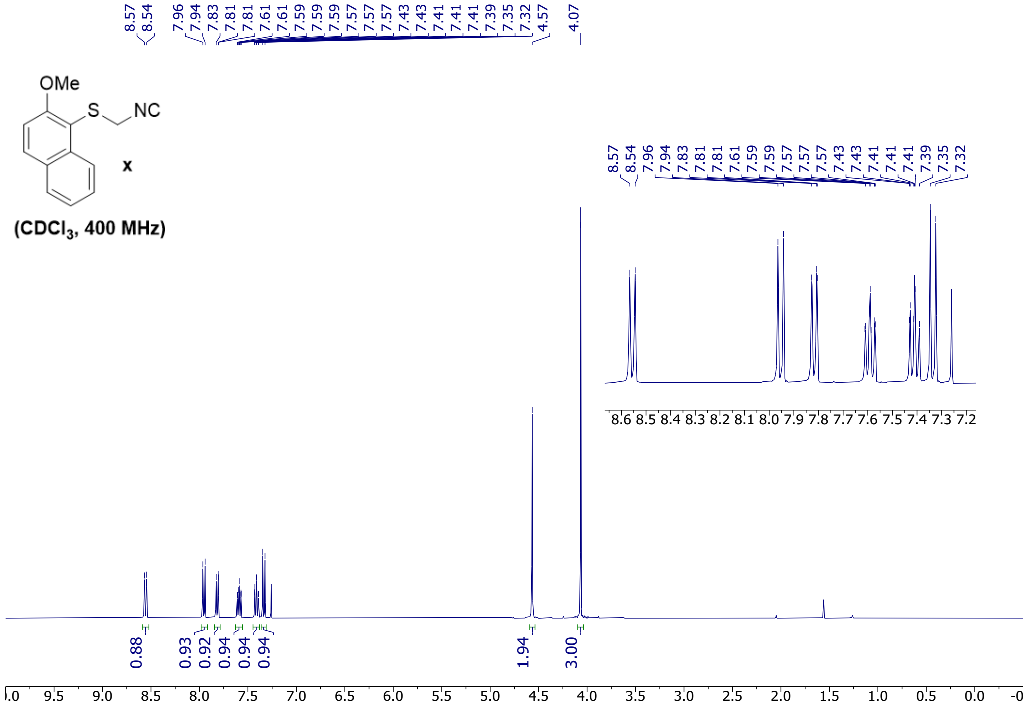

Supplementary Figure 20. ^{13}C NMR of Compound **x** at 25 °C

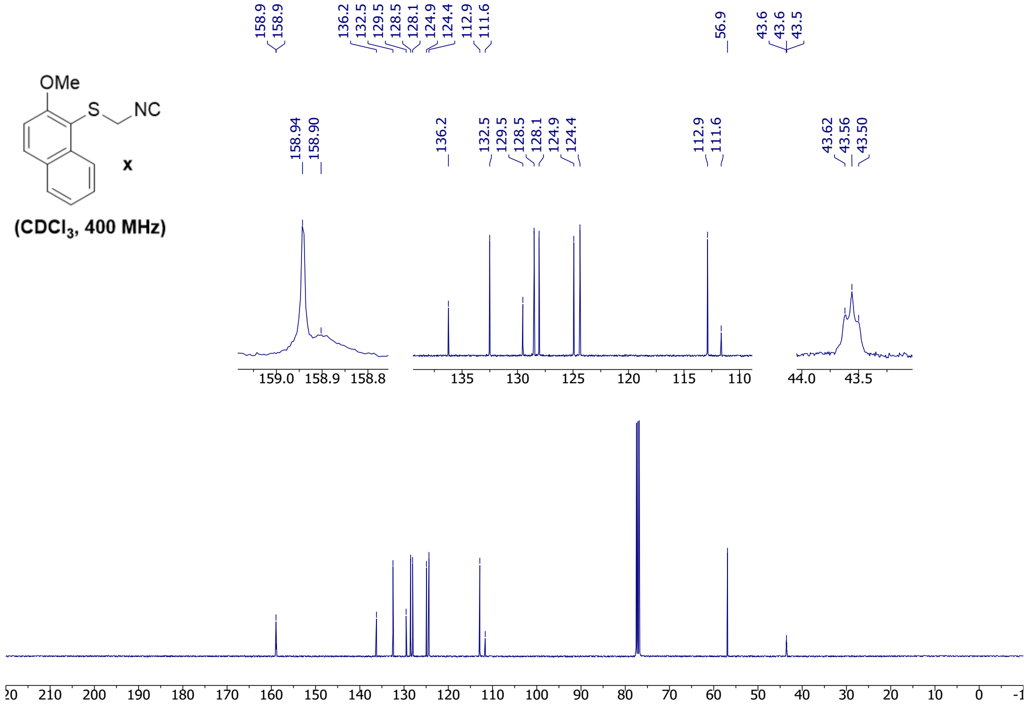

Supplementary Figure 21. ^{1}H NMR of Compound **19a** at 25 °C

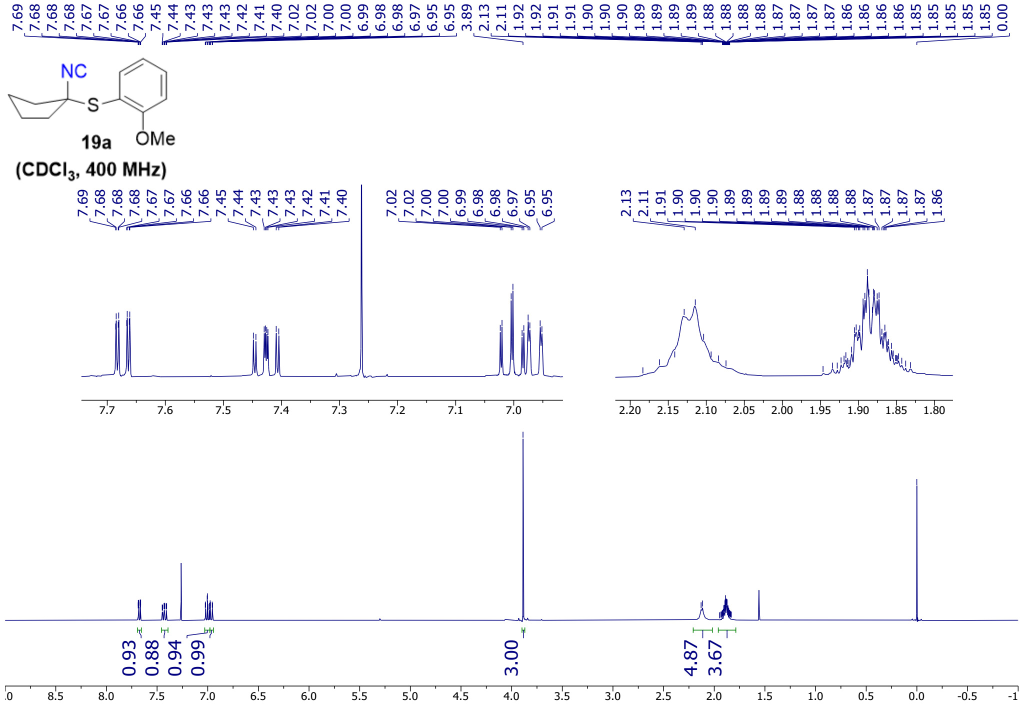

Supplementary Figure 22. ^{13}C NMR of Compound **19a** at 25 °C

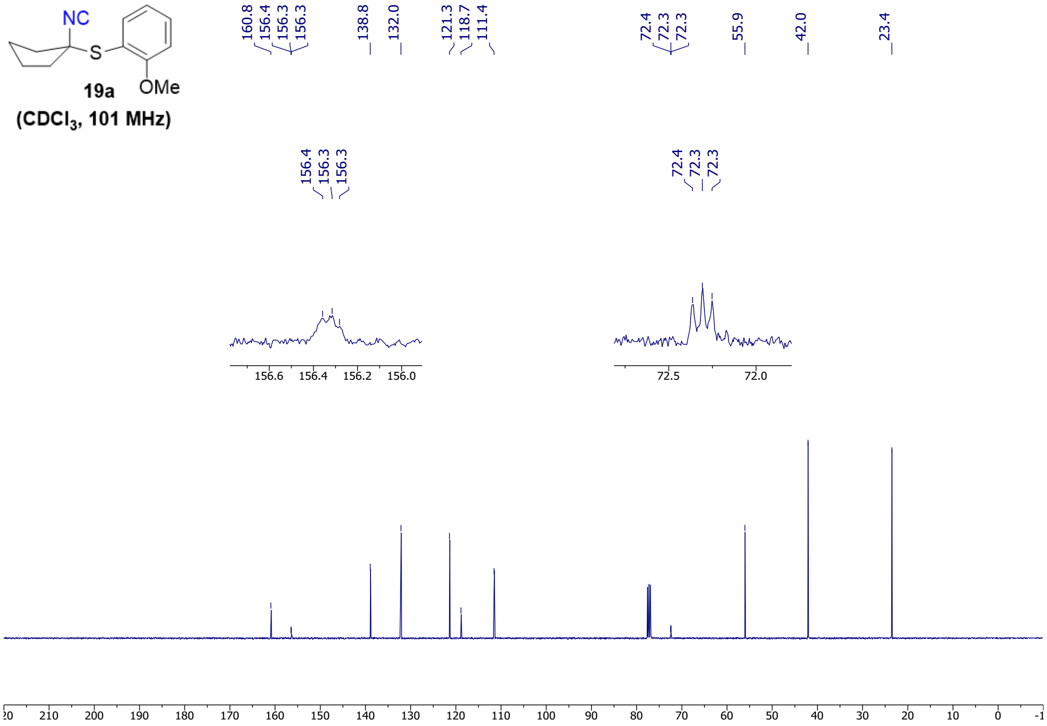

Supplementary Figure 23. ^{1}H NMR of Compound **19b** at 25 °C

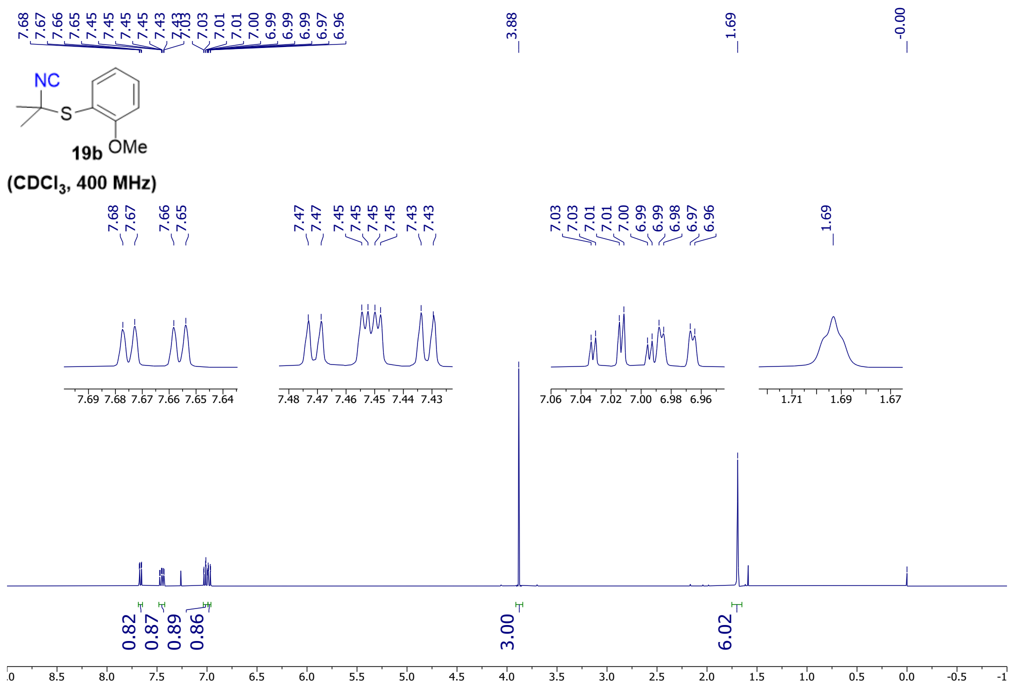

Supplementary Figure 24. ^{13}C NMR of Compound **19b** at 25 °C

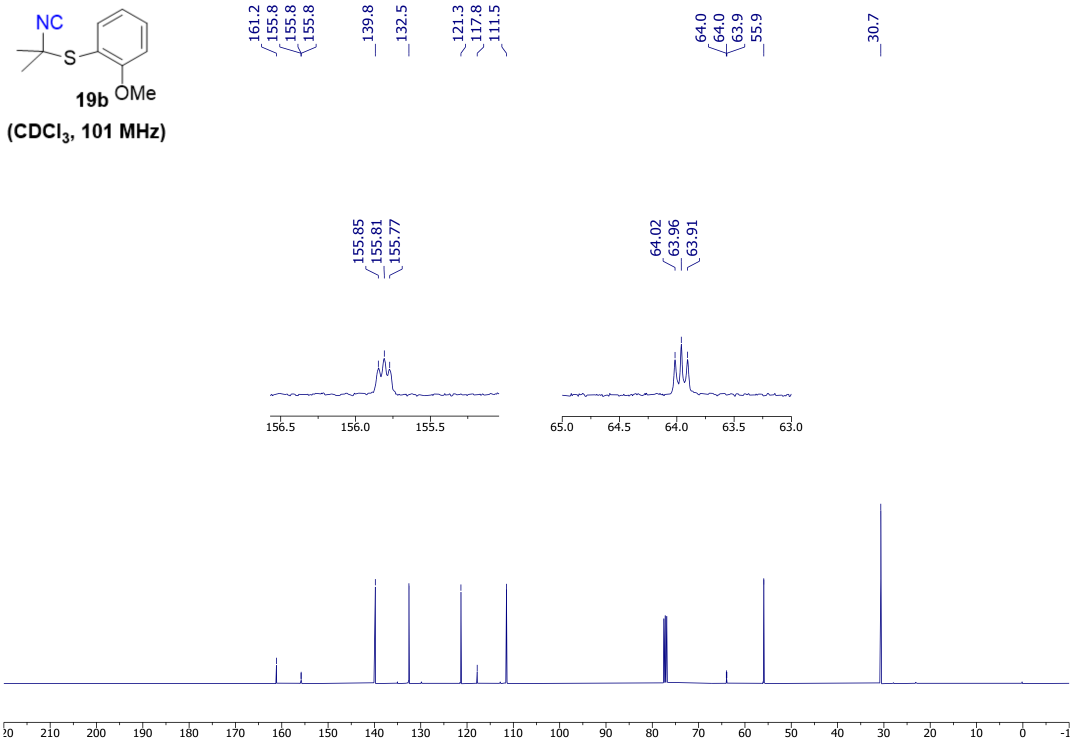

Supplementary Figure 25. ^{1}H NMR of Compound **19c** at 25 °C

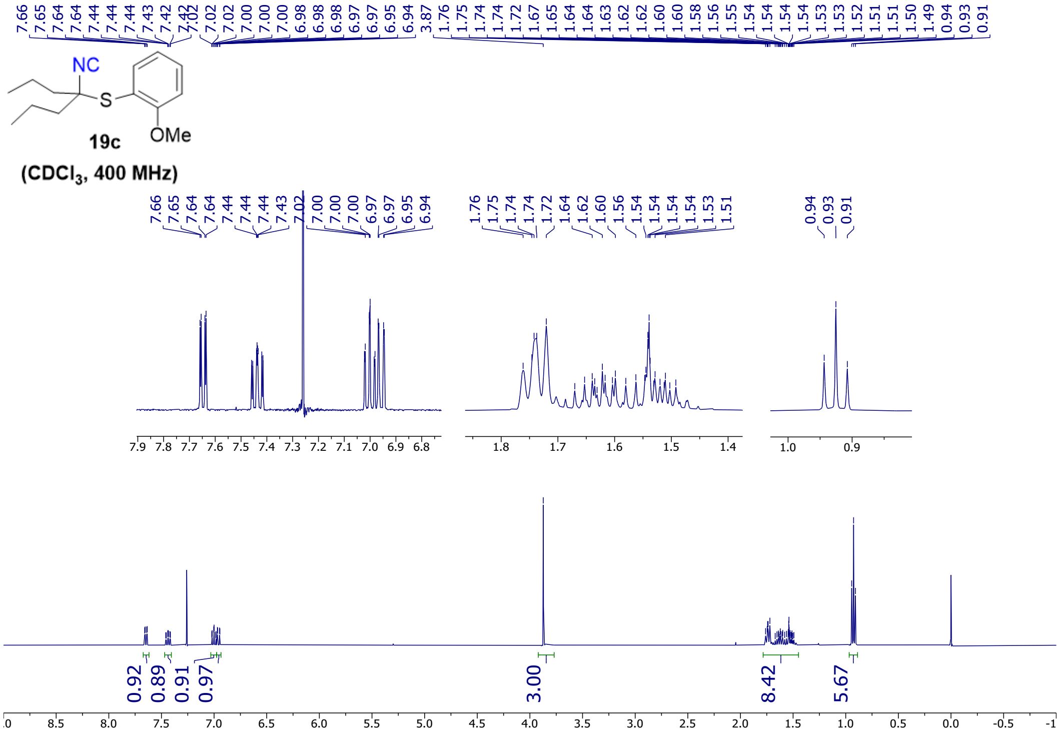

Supplementary Figure 26. ^{13}C NMR of Compound **19c** at 25 °C

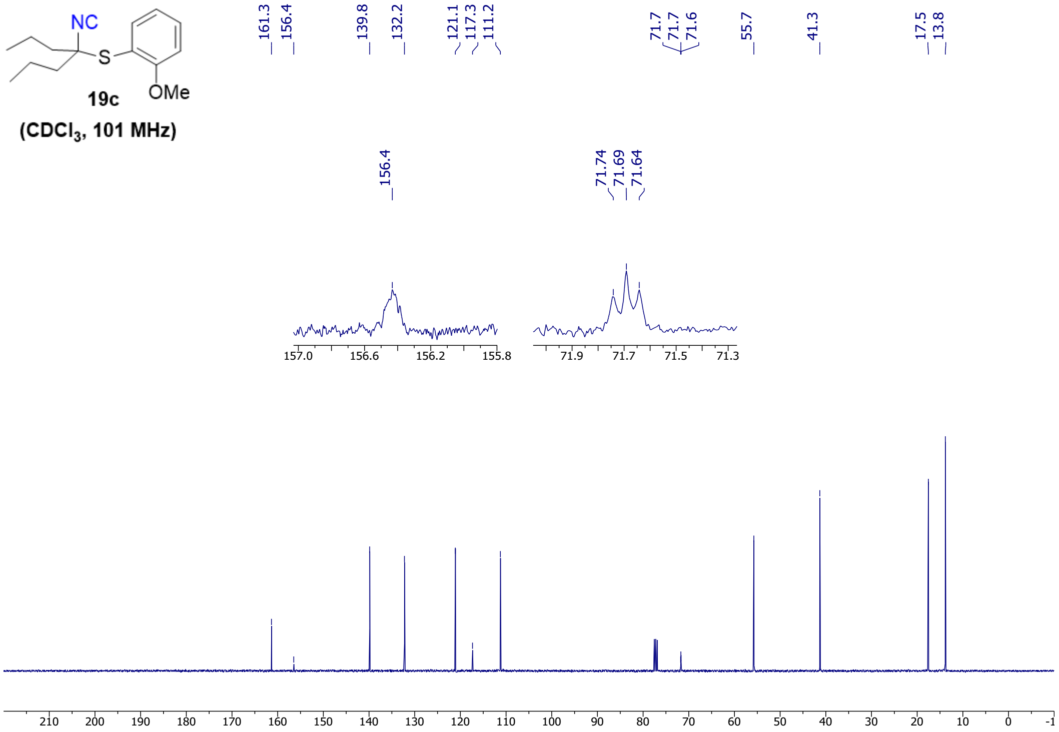

Supplementary Figure 27. ^{1}H NMR of Compound **19d** at 25 °C

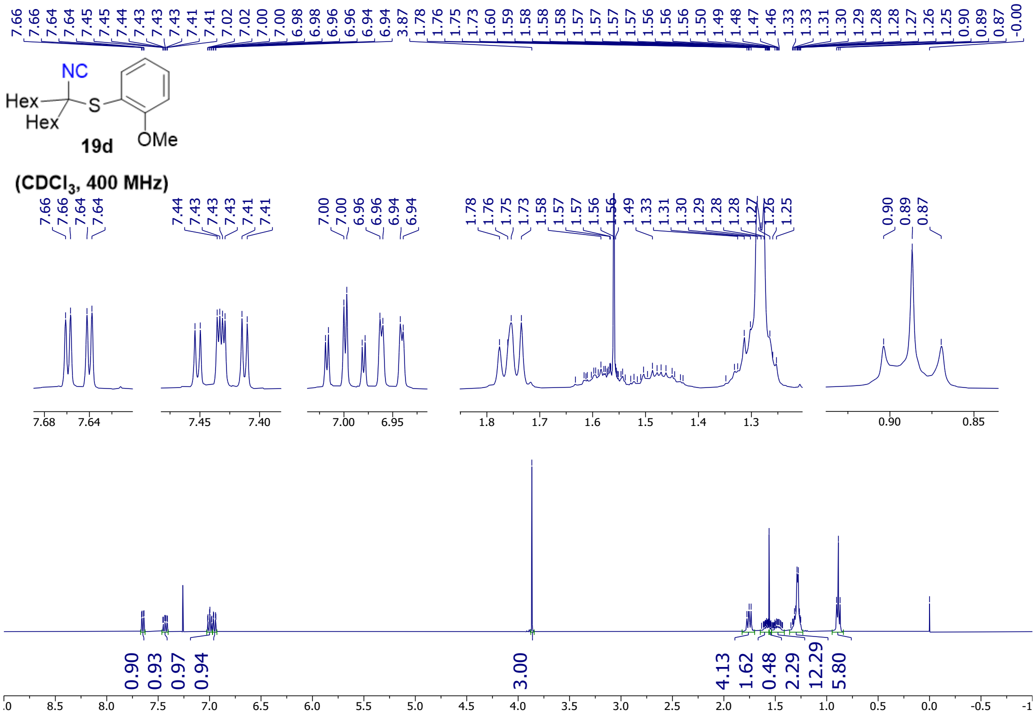

Supplementary Figure 28. ^{13}C NMR of Compound **19d** at 25 °C

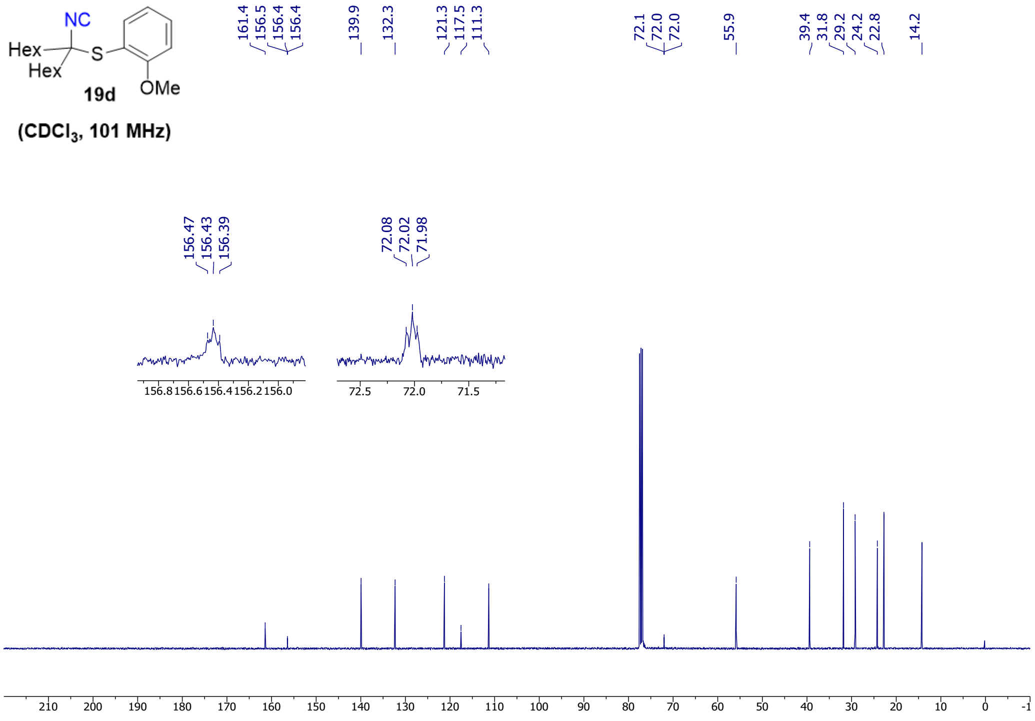

Supplementary Figure 29. ^{1}H NMR of Compound **19e** at 25 °C

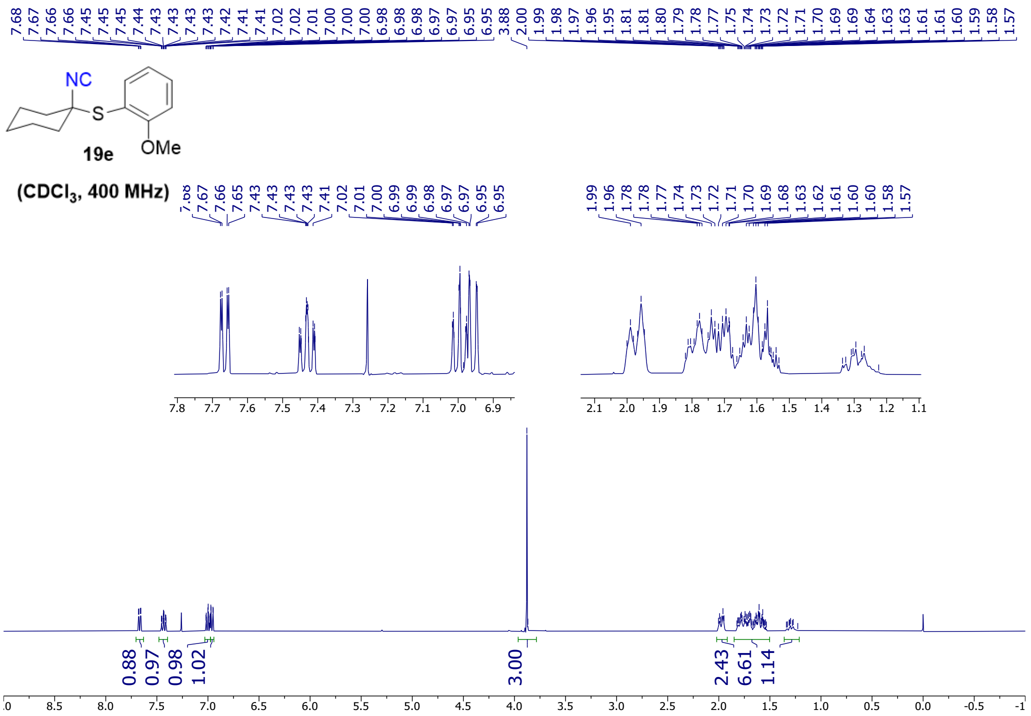

Supplementary Figure 30. ^{13}C NMR of Compound **19e** at 25 °C

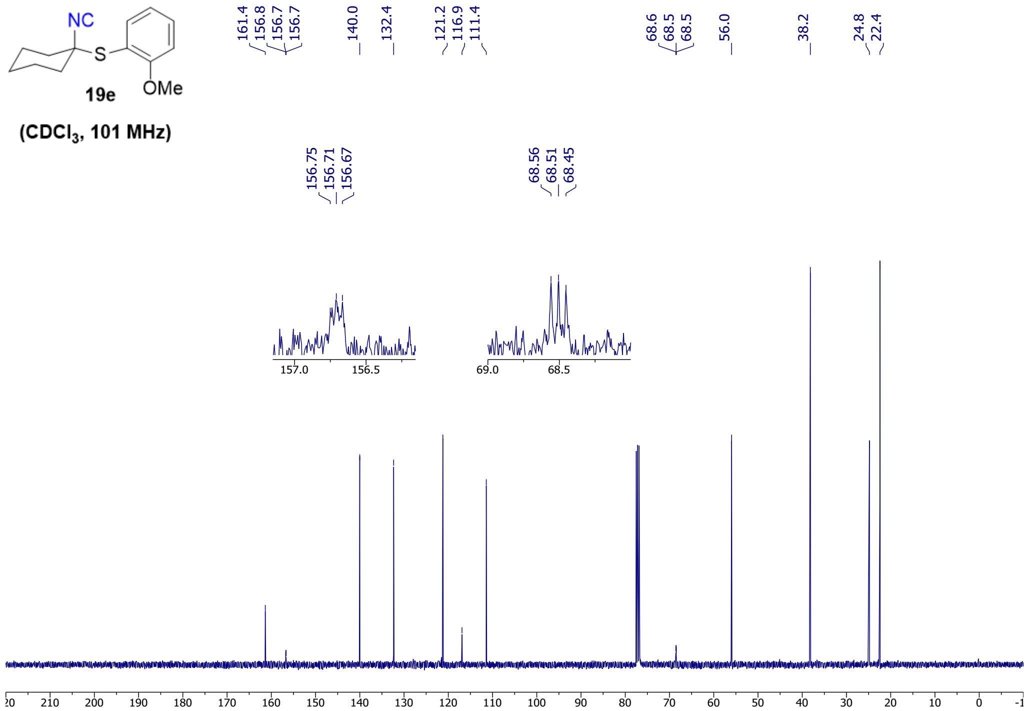

Supplementary Figure 31. ^{1}H NMR of Compound **19f** at 25 °C

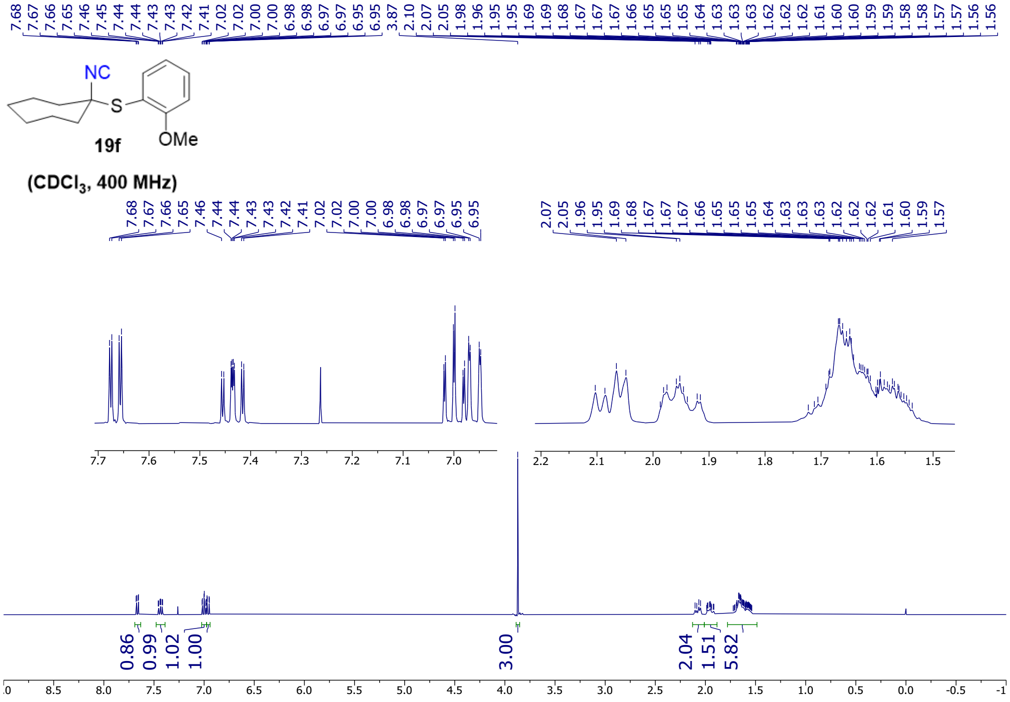

Supplementary Figure 32. ^{13}C NMR of Compound **19f** at 25 °C

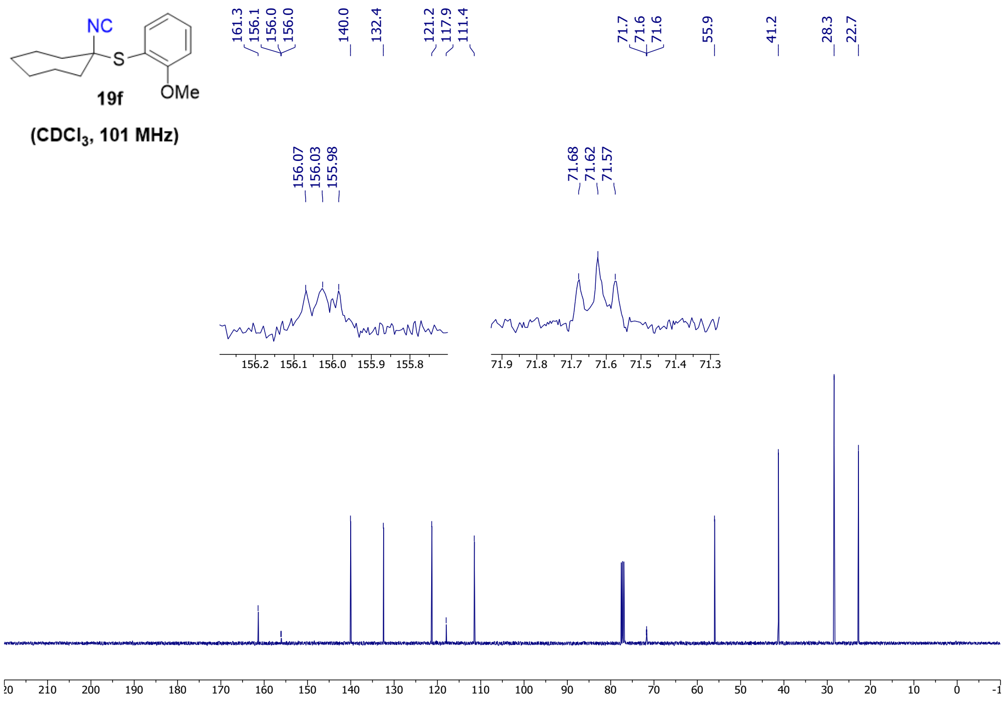

Supplementary Figure 33. ^{1}H NMR of Compound **22a** at 25 °C

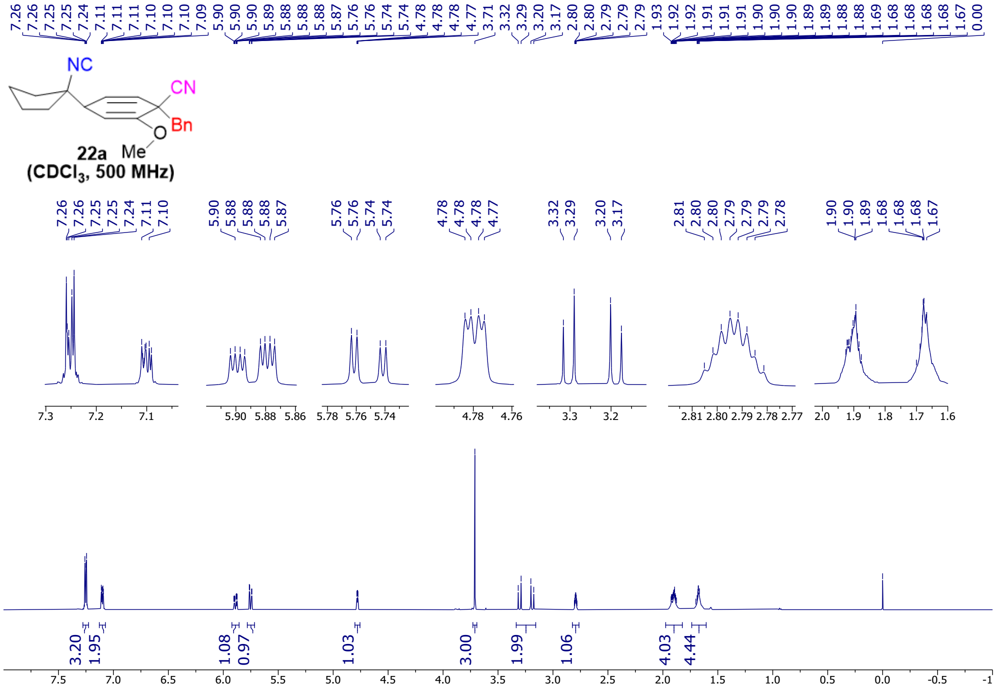

Supplementary Figure 34. ^{13}C NMR of Compound **22a** at 25 °C

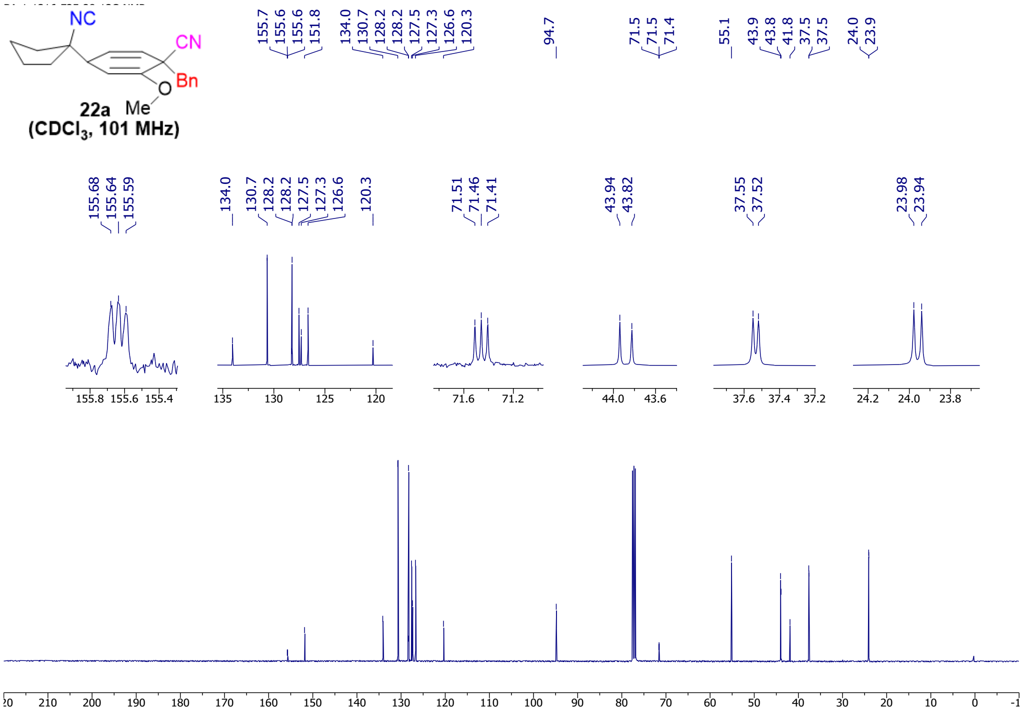

Supplementary Figure 35. ^{1}H NMR of Compound **22b** at 25 °C

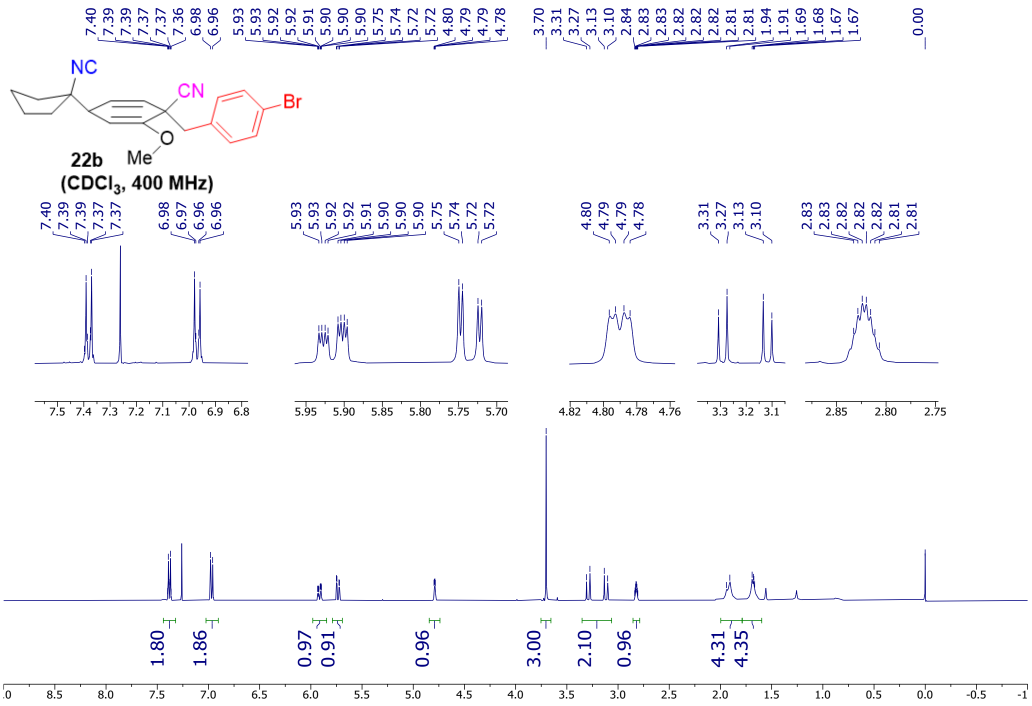

Supplementary Figure 36. ^{13}C NMR of Compound **22b** at 25 °C

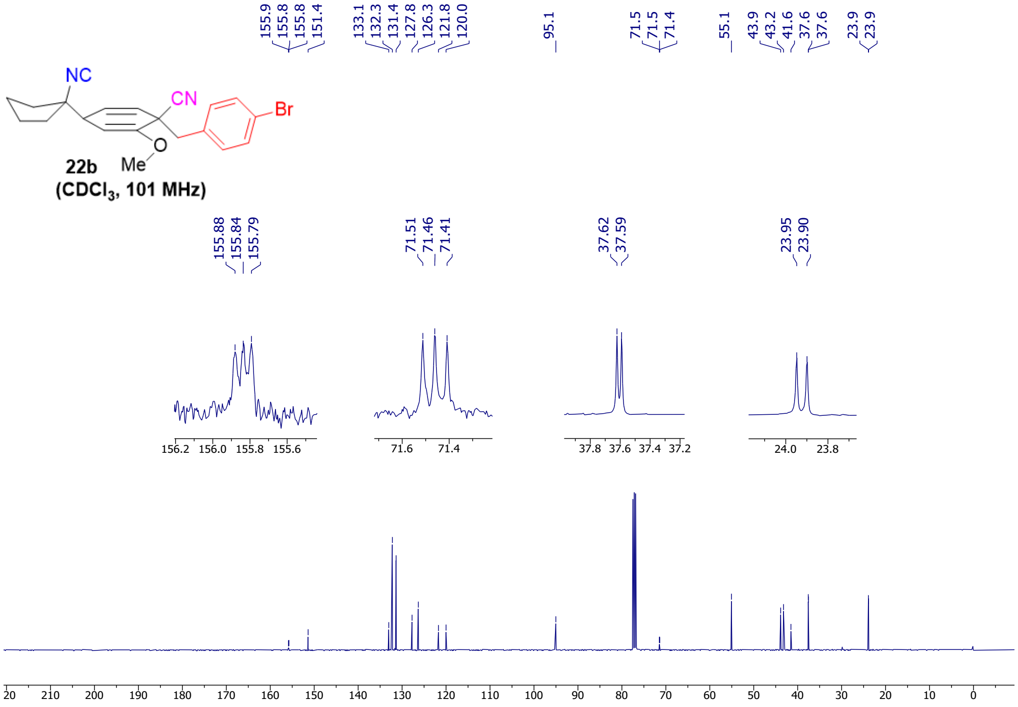

Supplementary Figure 37. ^{1}H NMR of Compound **22c** at 25 °C

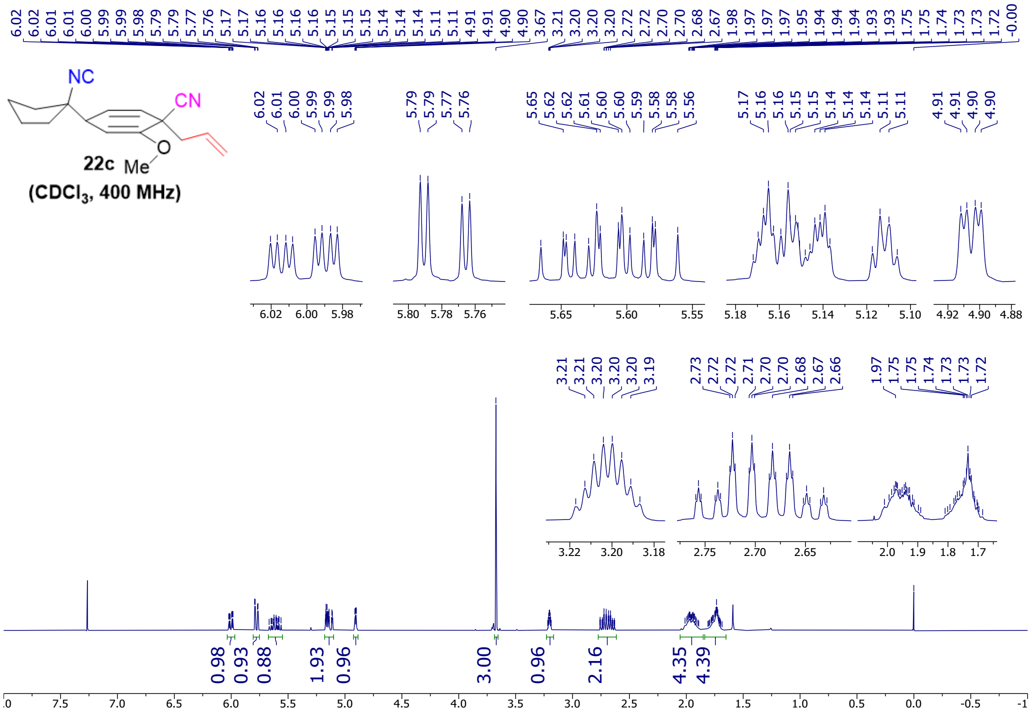

Supplementary Figure 38. ^{13}C NMR of Compound **22c** at 25 °C

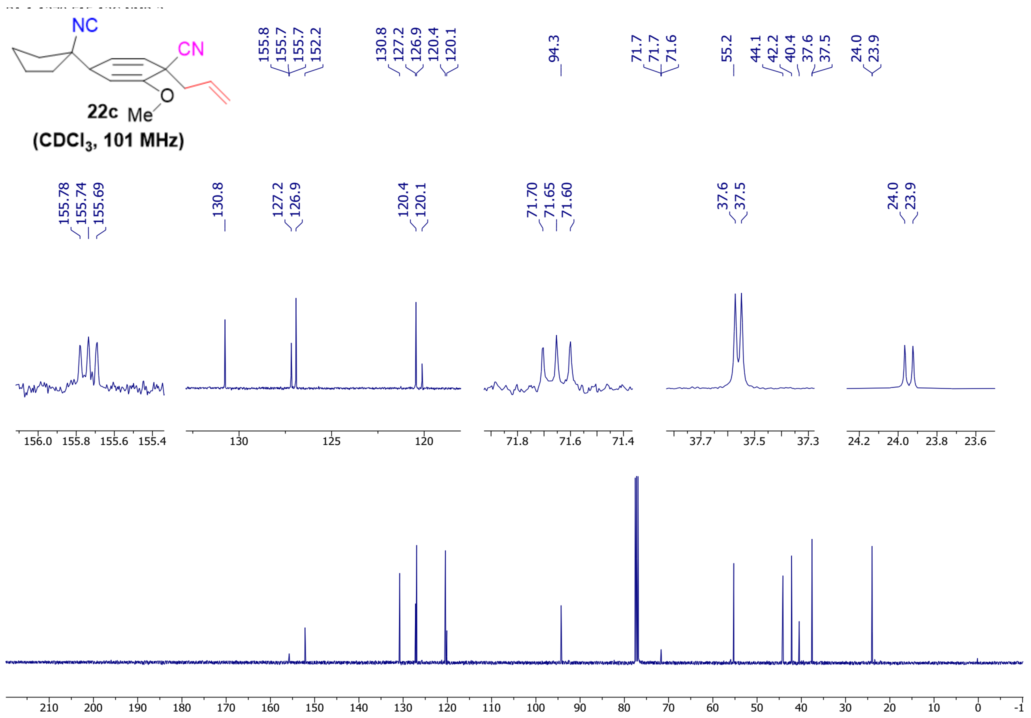

Supplementary Figure 39. ^{1}H NMR of Compound **22d** at 25 °C

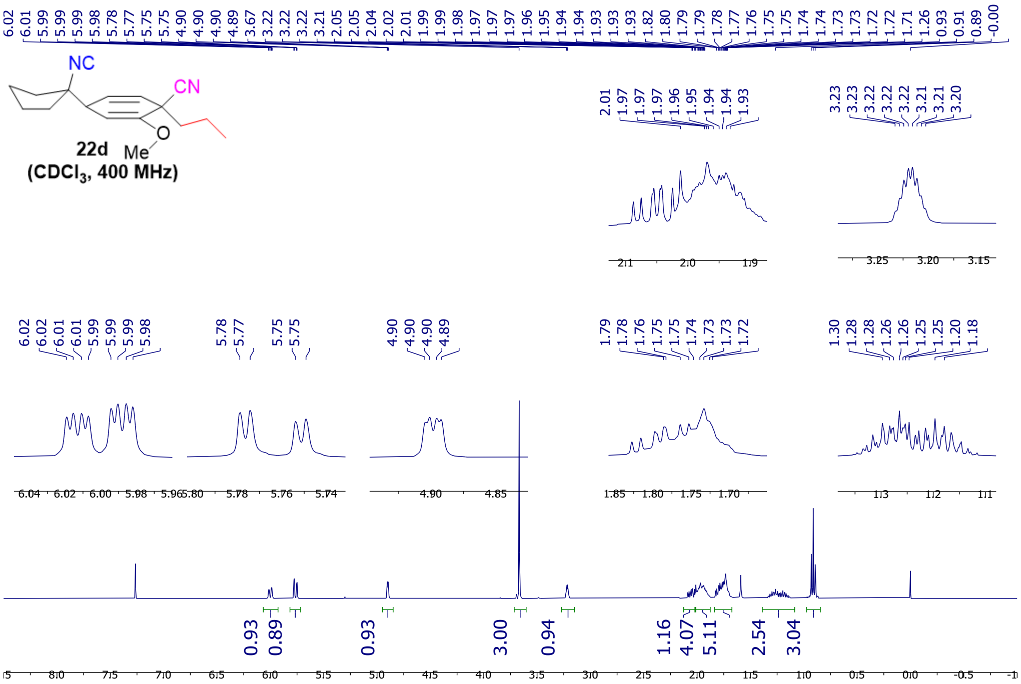

Supplementary Figure 40. ^{13}C NMR of Compound **22d** at 25 °C

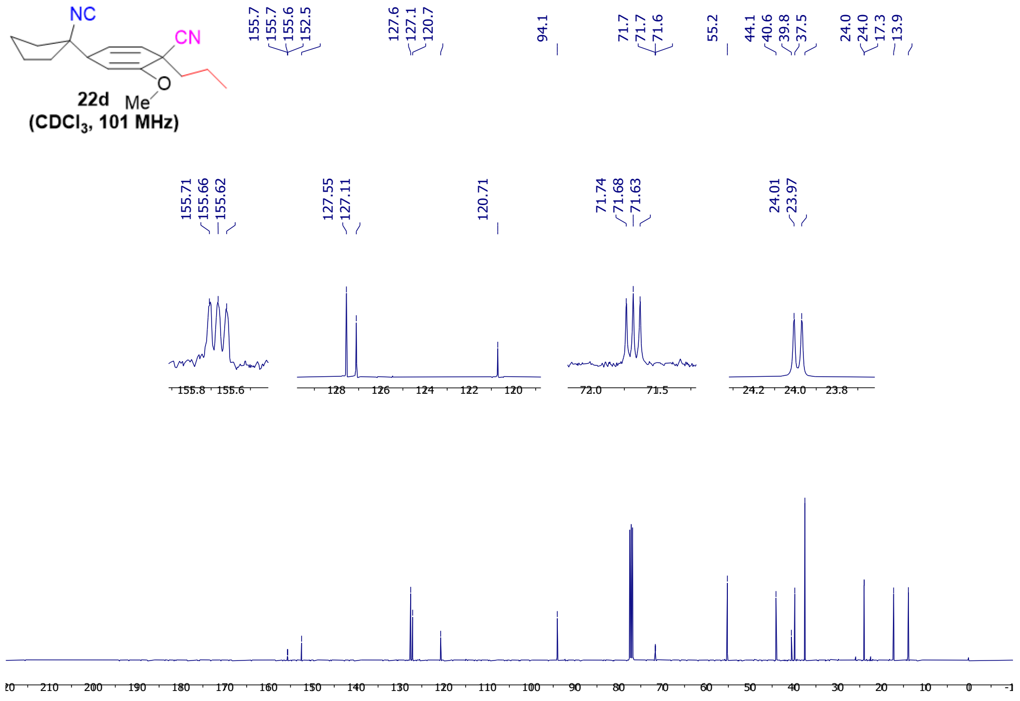

Supplementary Figure 41. ^{1}H NMR of Compound **22e** at 25 °C

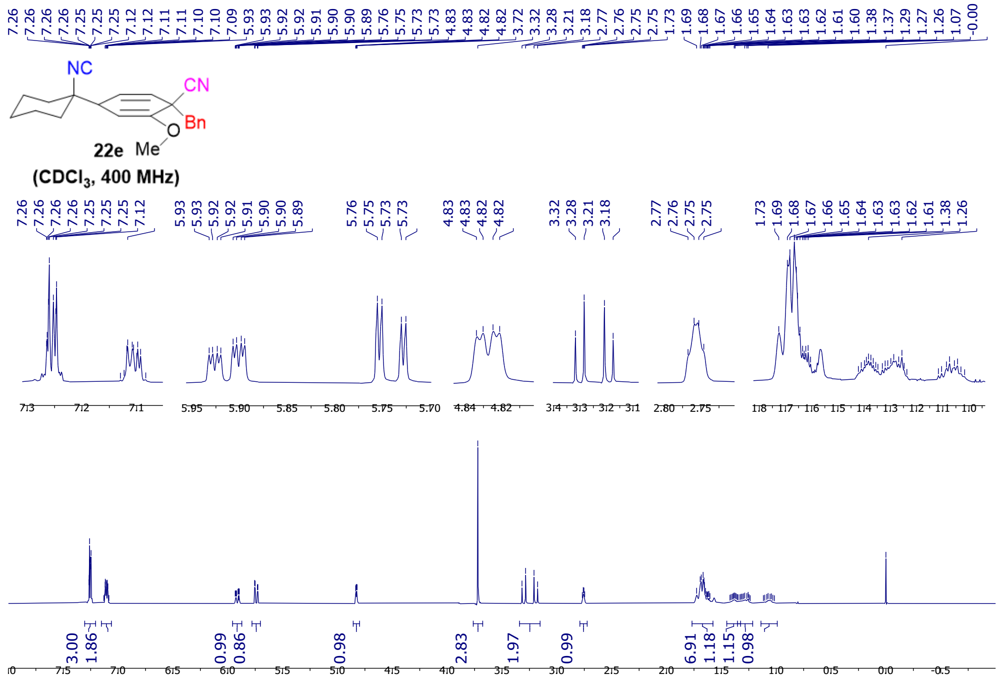

Supplementary Figure 42. ^{13}C NMR of Compound **22e** at 25 °C

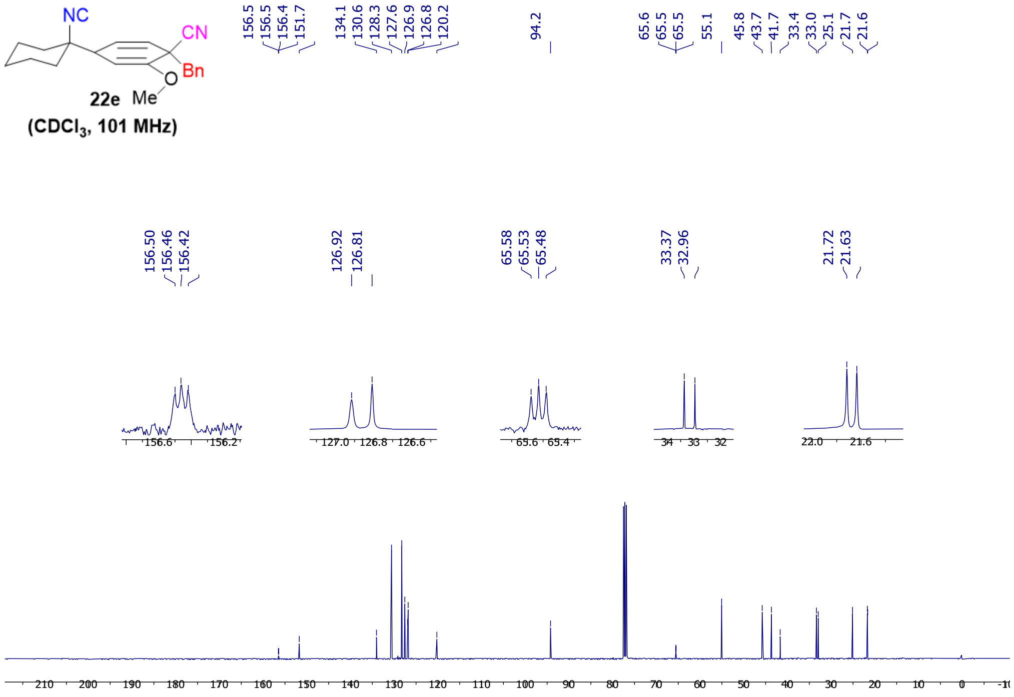

Supplementary Figure 43. ^{1}H NMR of Compound **22f** at 25 °C

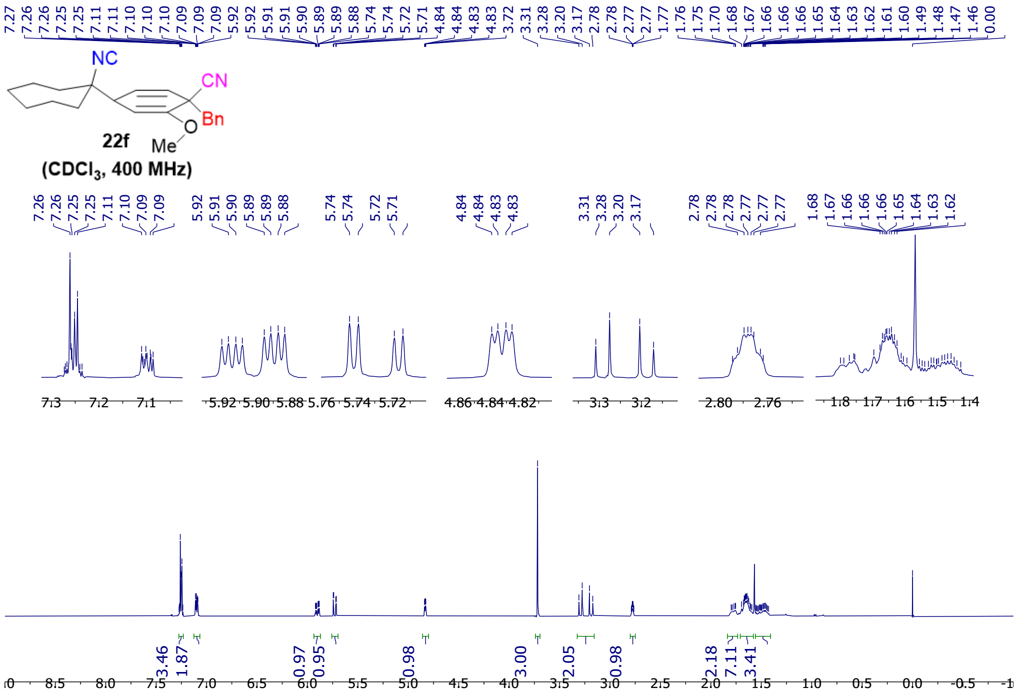

Supplementary Figure 44. ^{13}C NMR of Compound **22f** at 25 °C

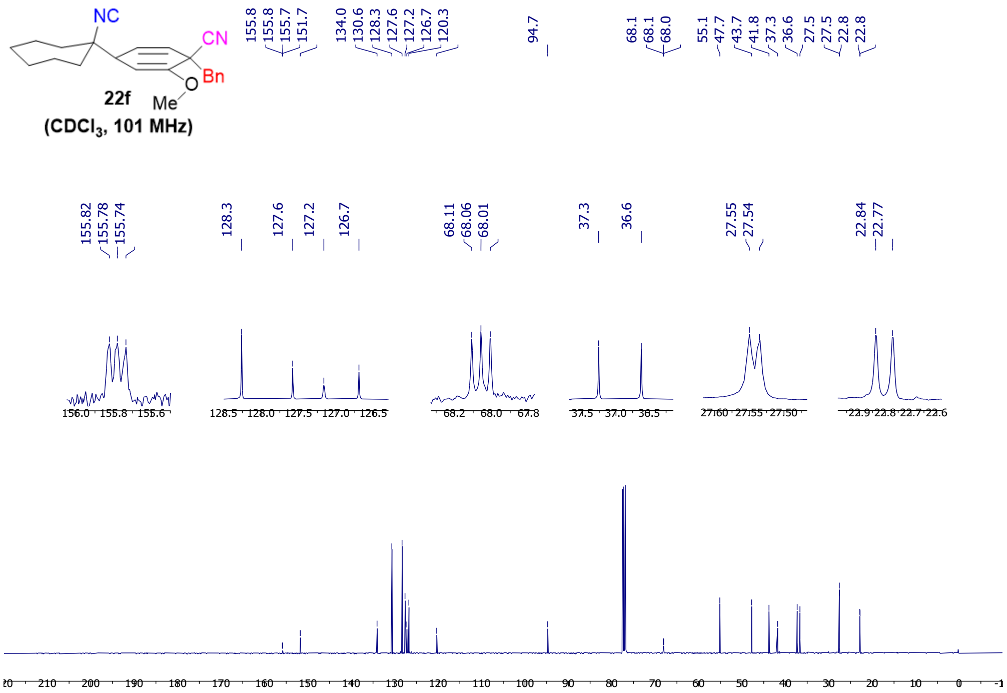

Supplementary Figure 45. ^{1}H NMR of Compound **22g** at 25 °C

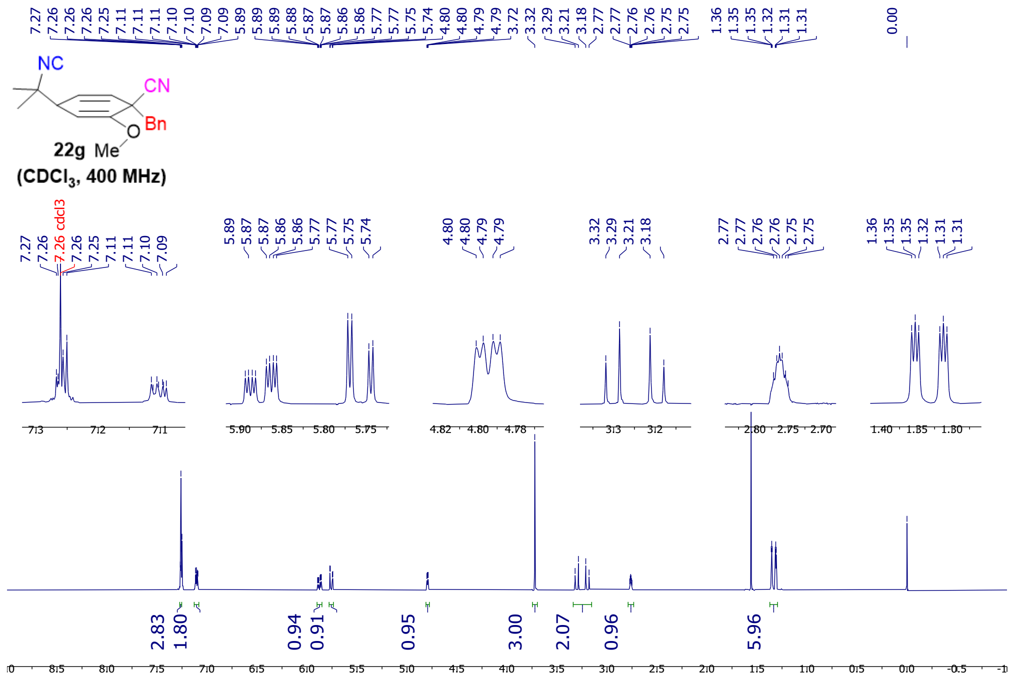

Supplementary Figure 46. ^{13}C NMR of Compound **22g** at 25 °C

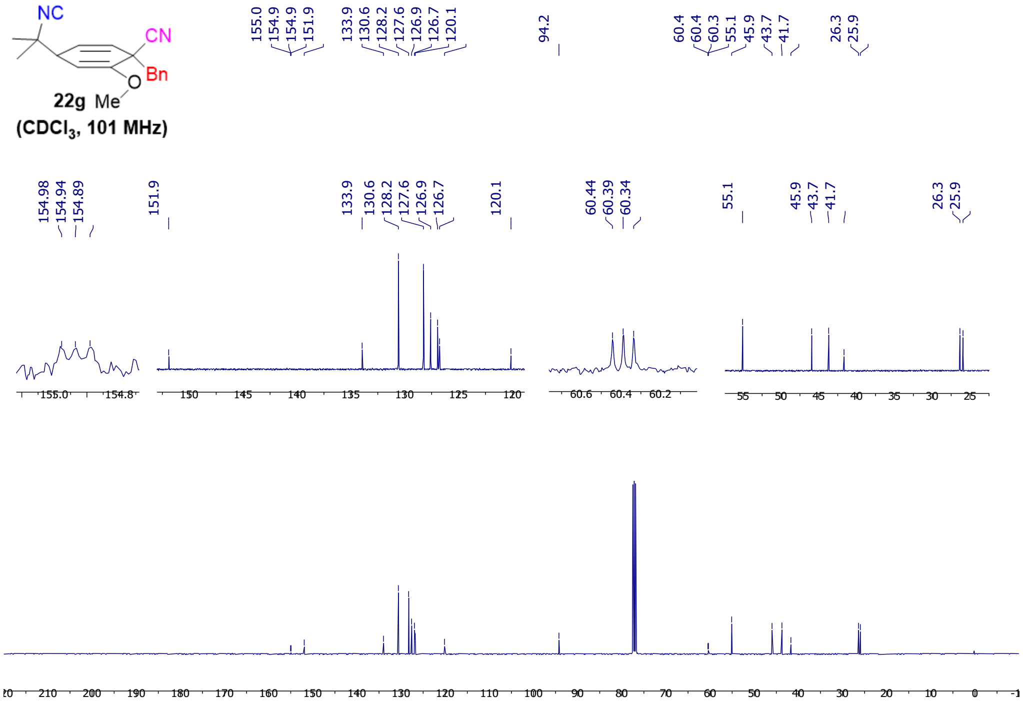

Supplementary Figure 47. ^{1}H NMR of Compound **22h** at 25 °C

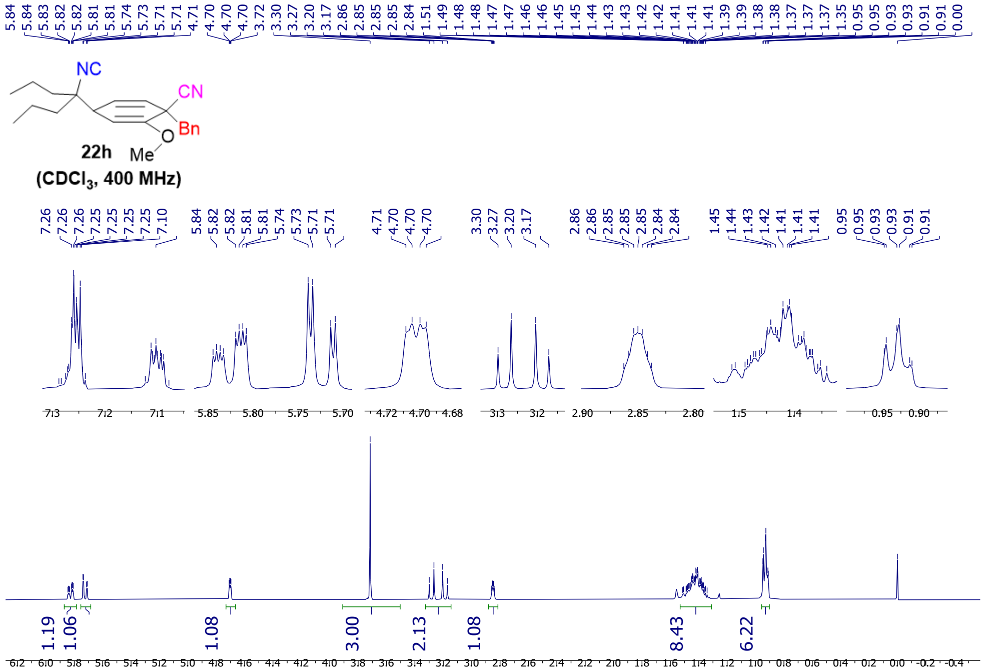

Supplementary Figure 48. ^{13}C NMR of Compound **22h** at 25 °C

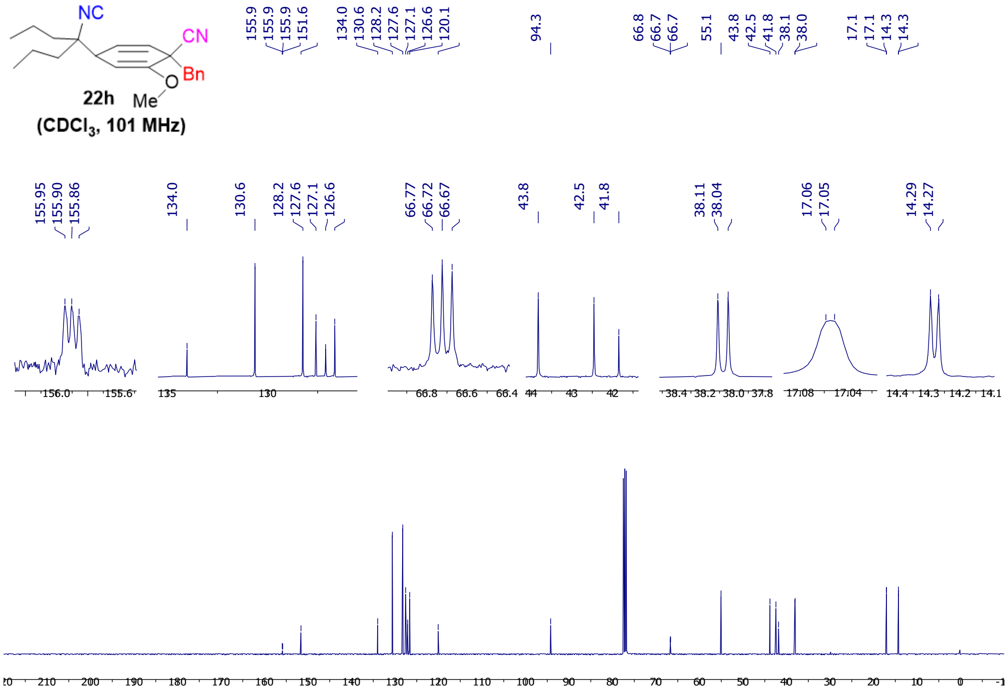

Supplementary Figure 49. ^{1}H NMR of Compound **22i** at 25 °C

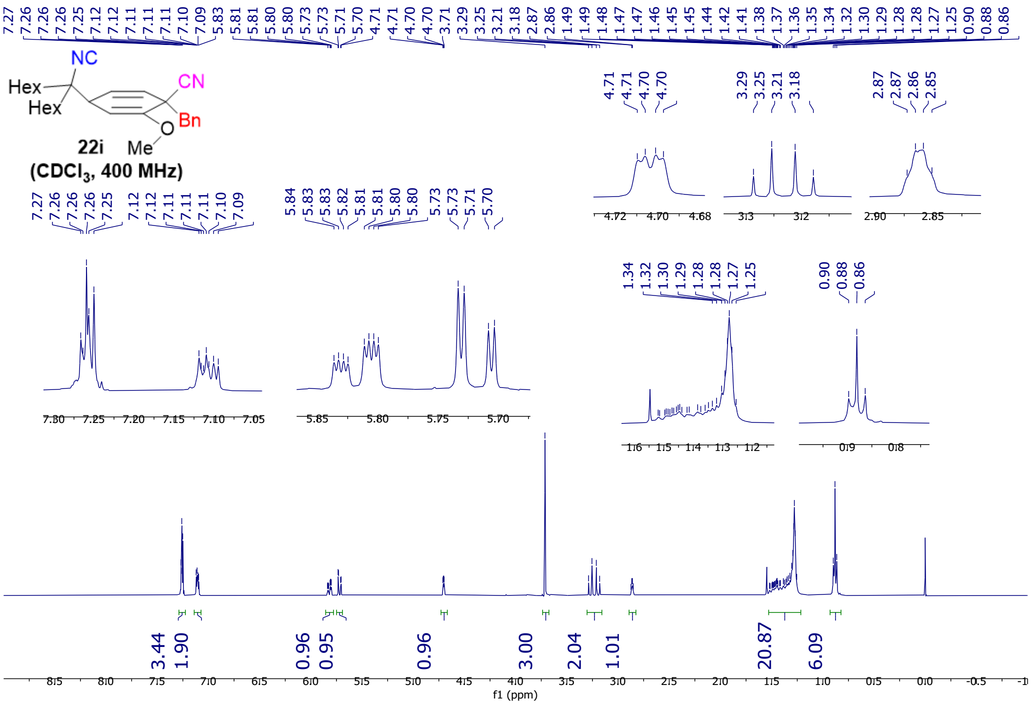

Supplementary Figure 50. ^{13}C NMR of Compound **22i** at 25 °C

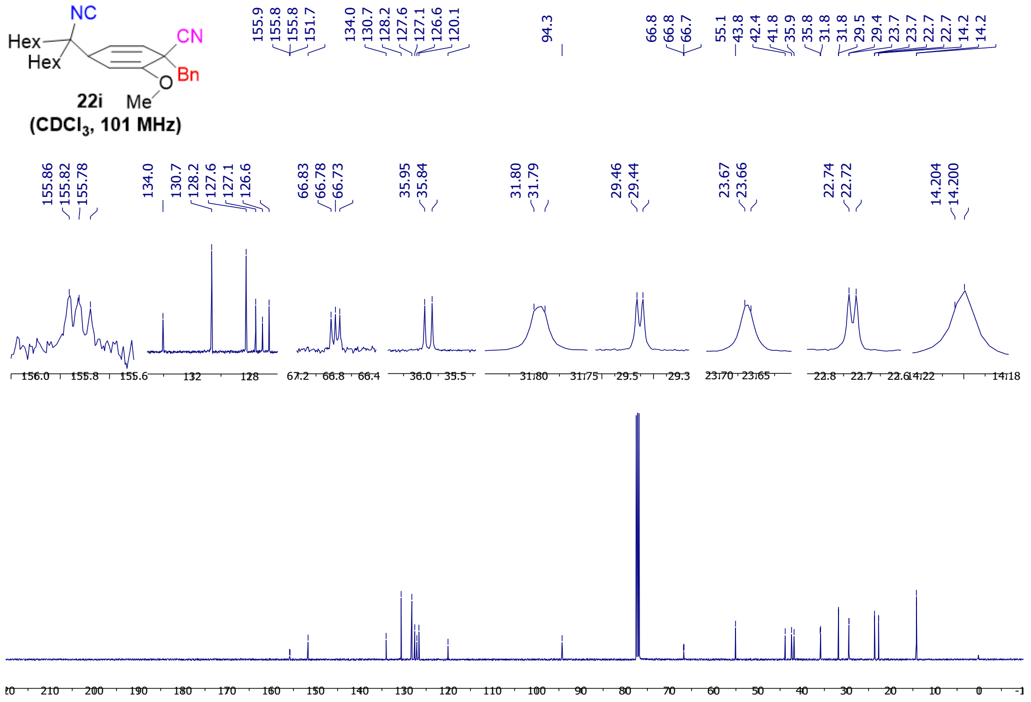

Supplementary Figure 51. ^{1}H NMR of Compound **23a** at 25 °C

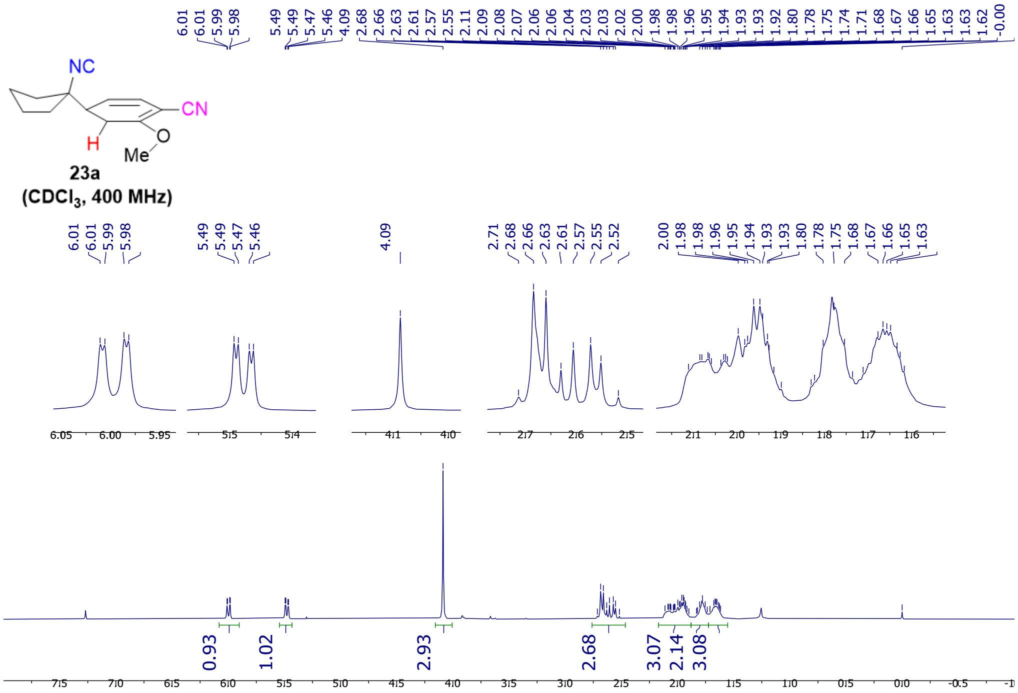

Supplementary Figure 52. ^{13}C NMR of Compound **23a** at 25 °C

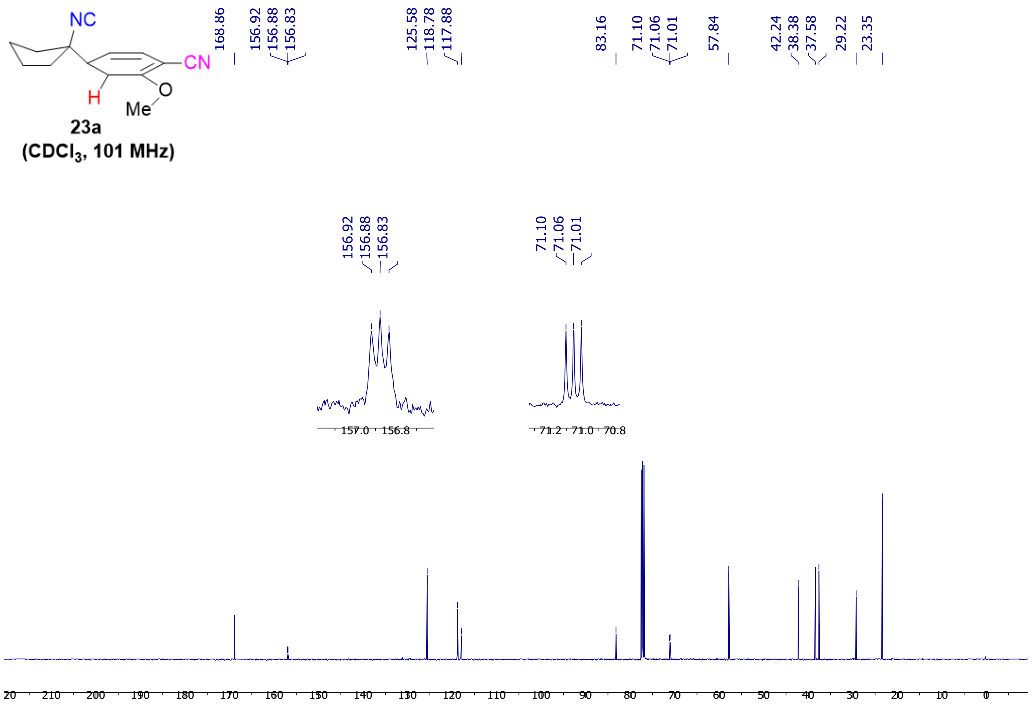

Supplementary Figure 53. ^{1}H NMR of Compound **23b** at 25 °C

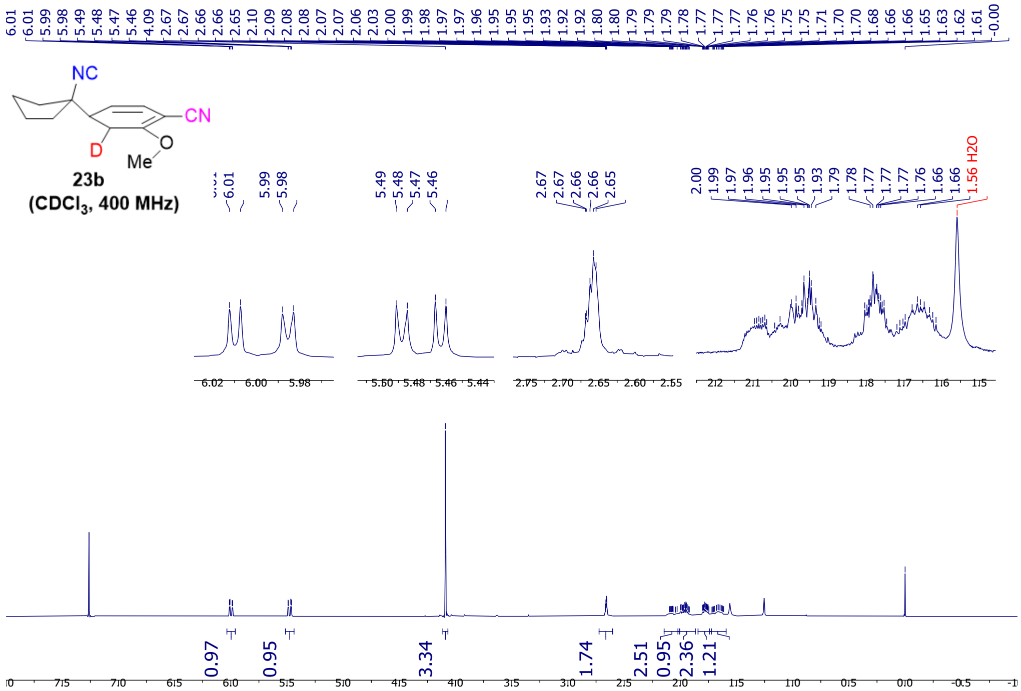

Supplementary Figure 54. ^{13}C NMR of Compound **23b** at 25 °C

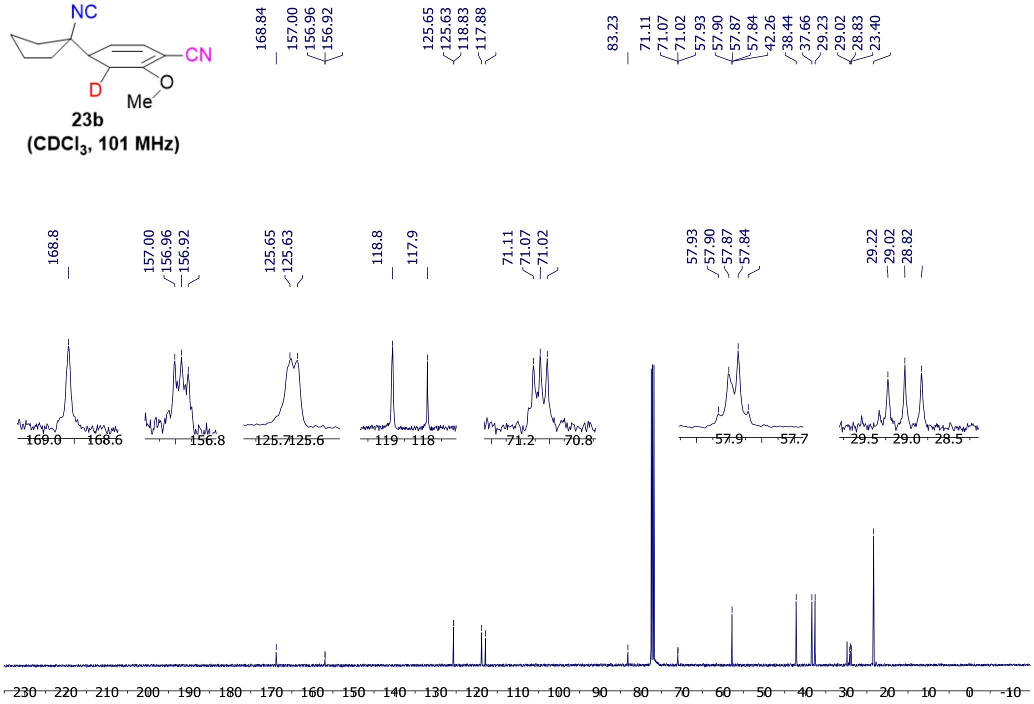

Supplementary Figure 55. ^{1}H NMR of Compound **23c** at 25 °C

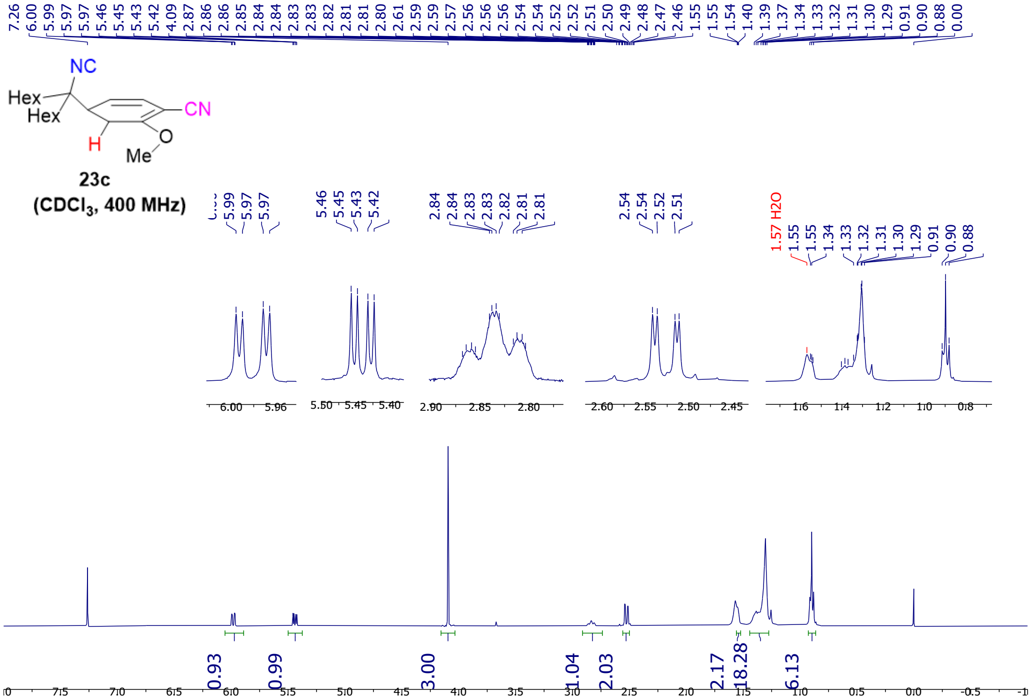

Supplementary Figure 56. ^{13}C NMR of Compound **23c** at 25 °C

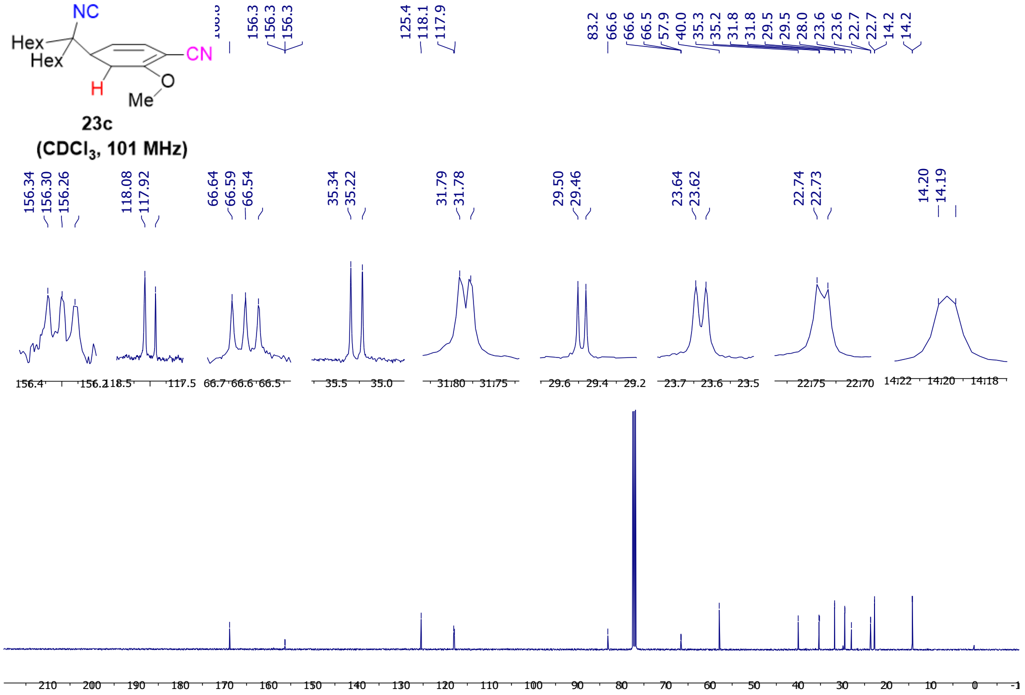

Supplementary Figure 57. ^{1}H NMR of Compound **24a** at 25 °C

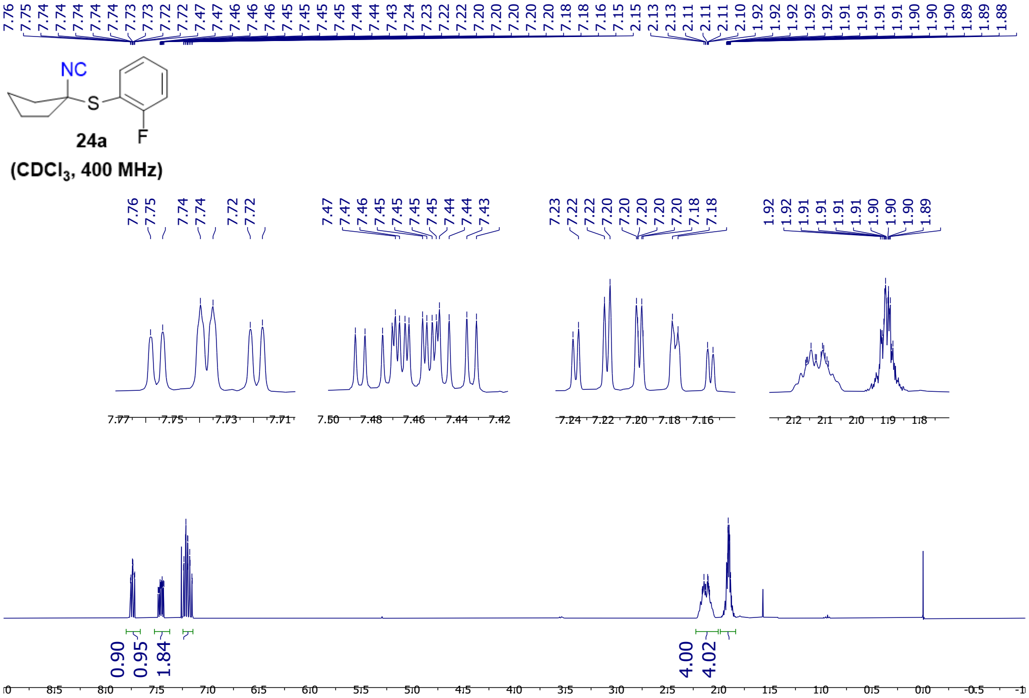

Supplementary Figure 58. ^{13}C NMR of Compound **24a** at 25 °C

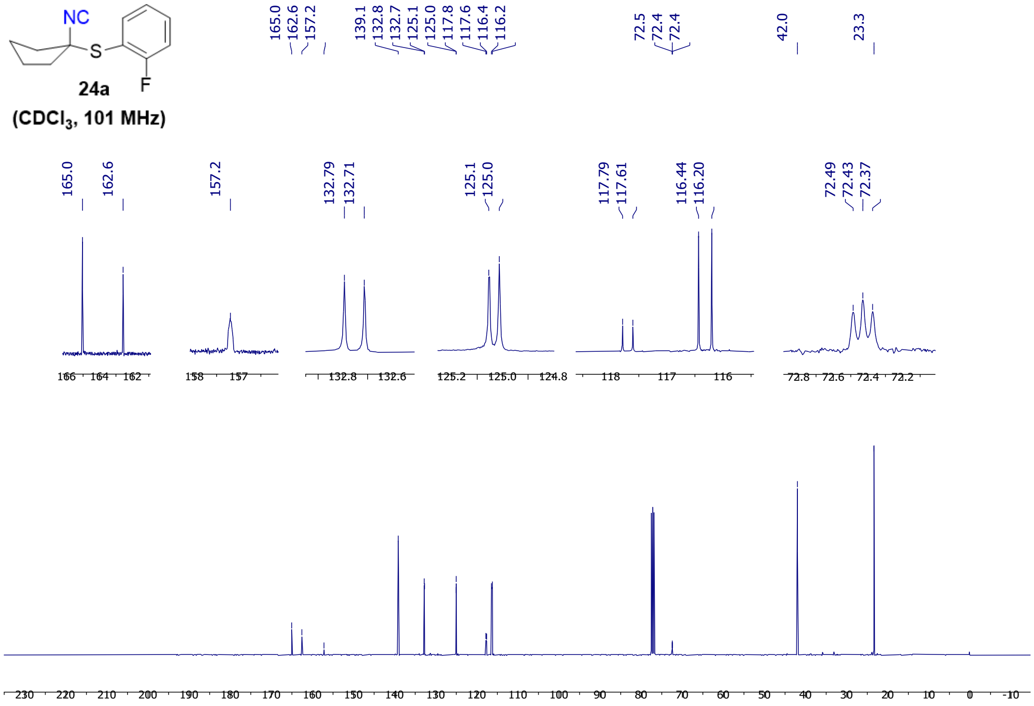

Supplementary Figure 59. ^{1}H NMR of Compound **24b** at 25 °C

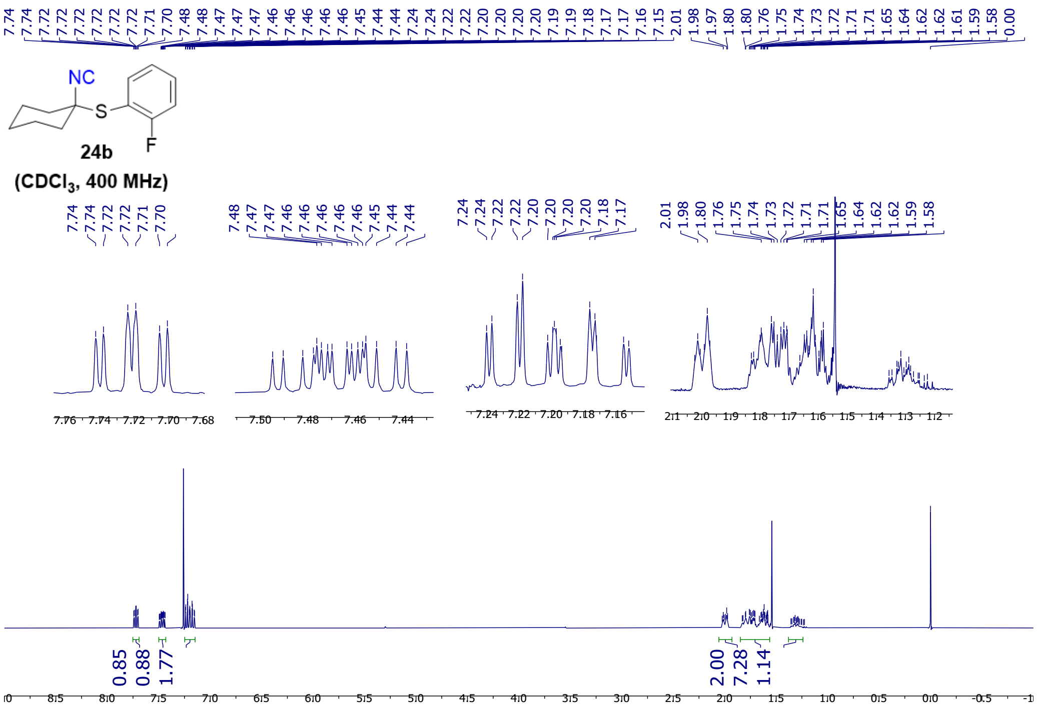

Supplementary Figure 60. ^{13}C NMR of Compound **24b** at 25 °C

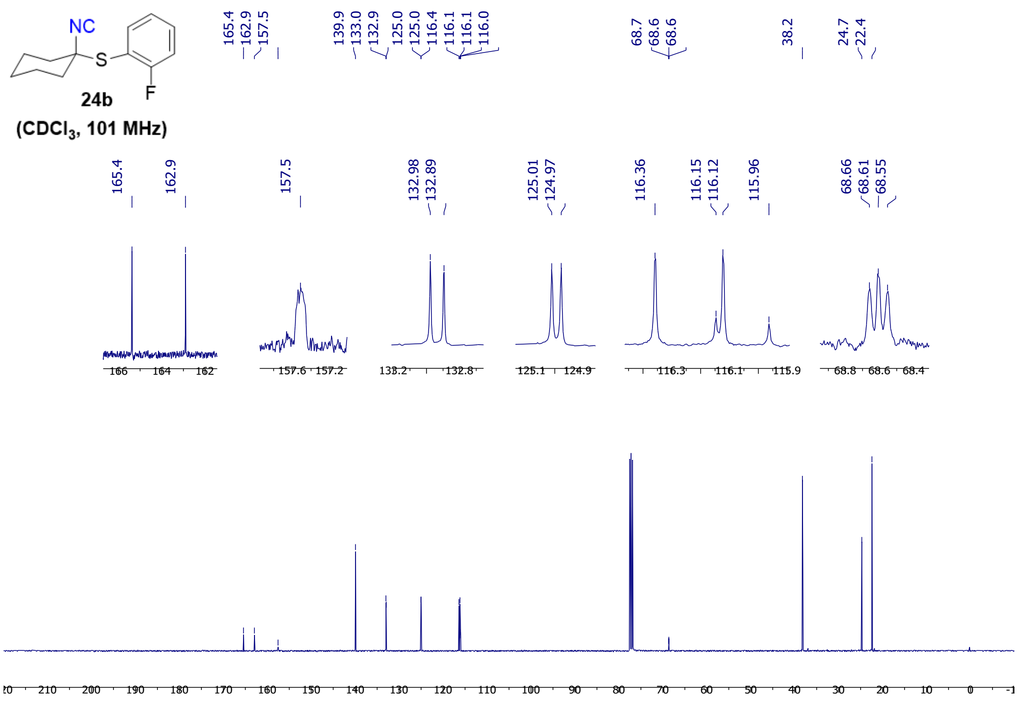

Supplementary Figure 61. ^{1}H NMR of Compound **24c** at 25 °C

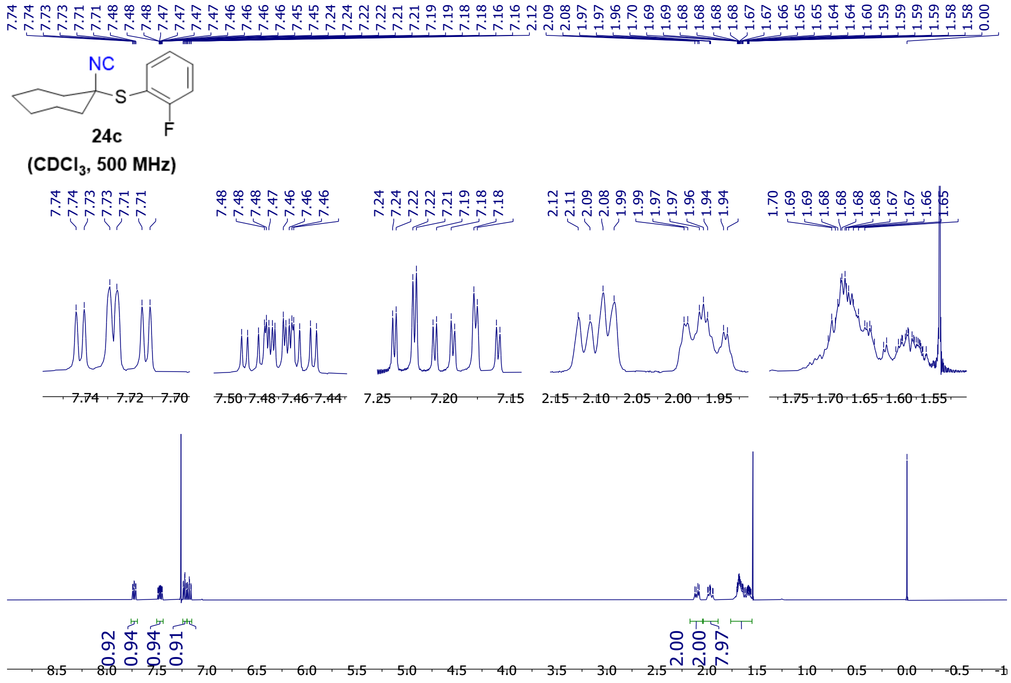

Supplementary Figure 62. ^{13}C NMR of Compound **24c** at 25 °C

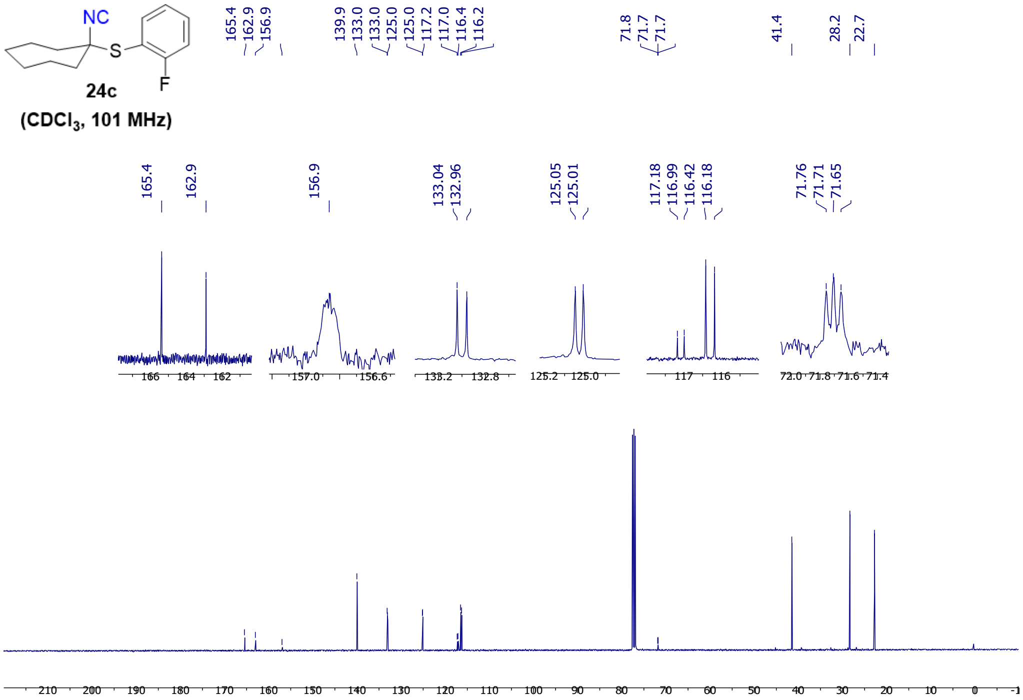

Supplementary Figure 63. ^{1}H NMR of Compound **24d** at 25 °C

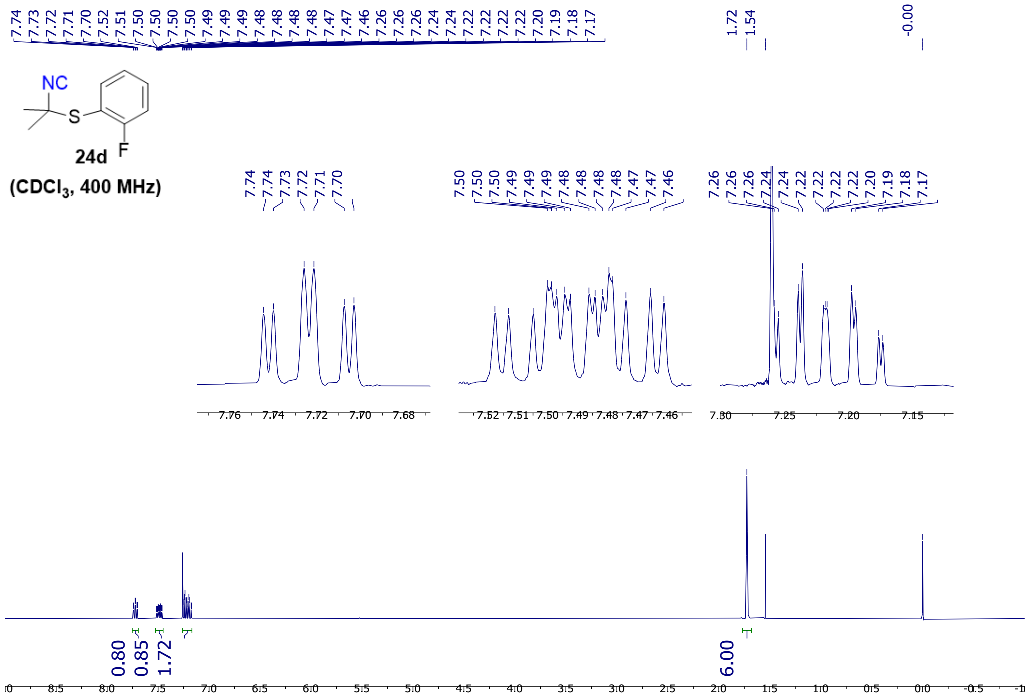

Supplementary Figure 64. ^{13}C NMR of Compound **24d** at 25 °C

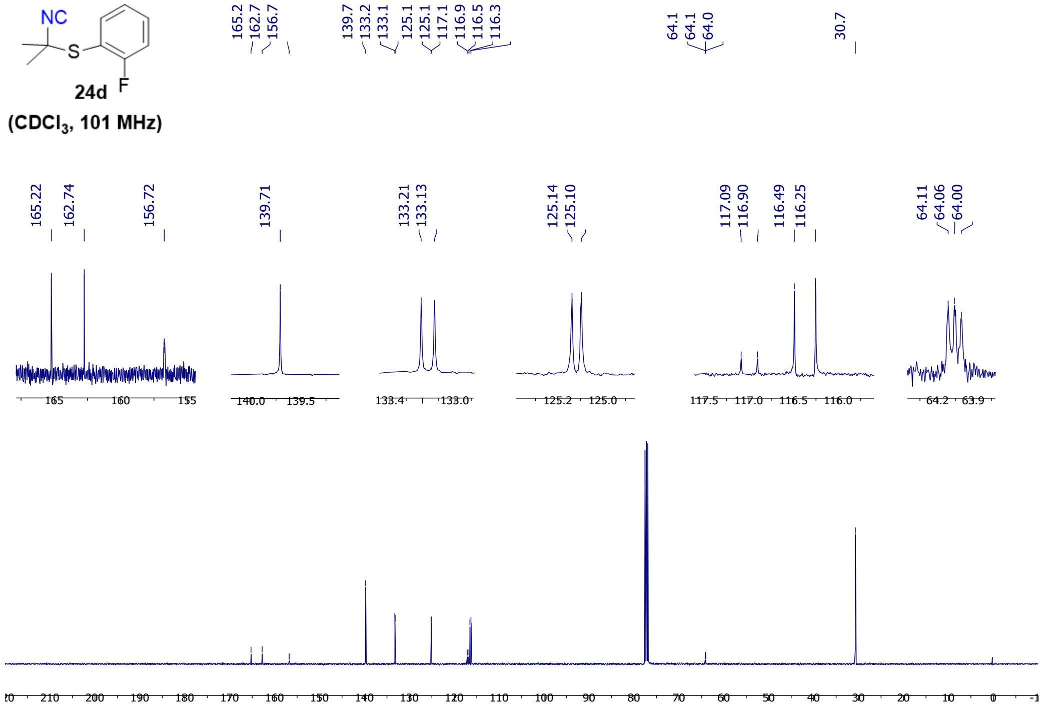

Supplementary Figure 65. ^{1}H NMR of Compound **24e** at 25 °C

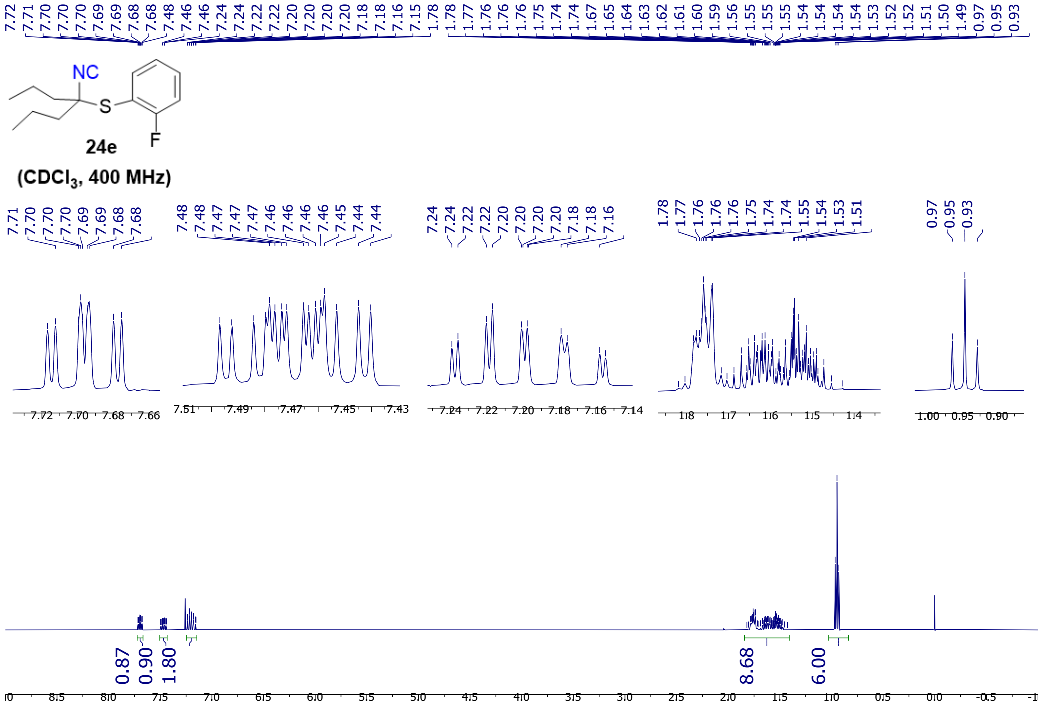

Supplementary Figure 66. ^{13}C NMR of Compound **24e** at 25 °C

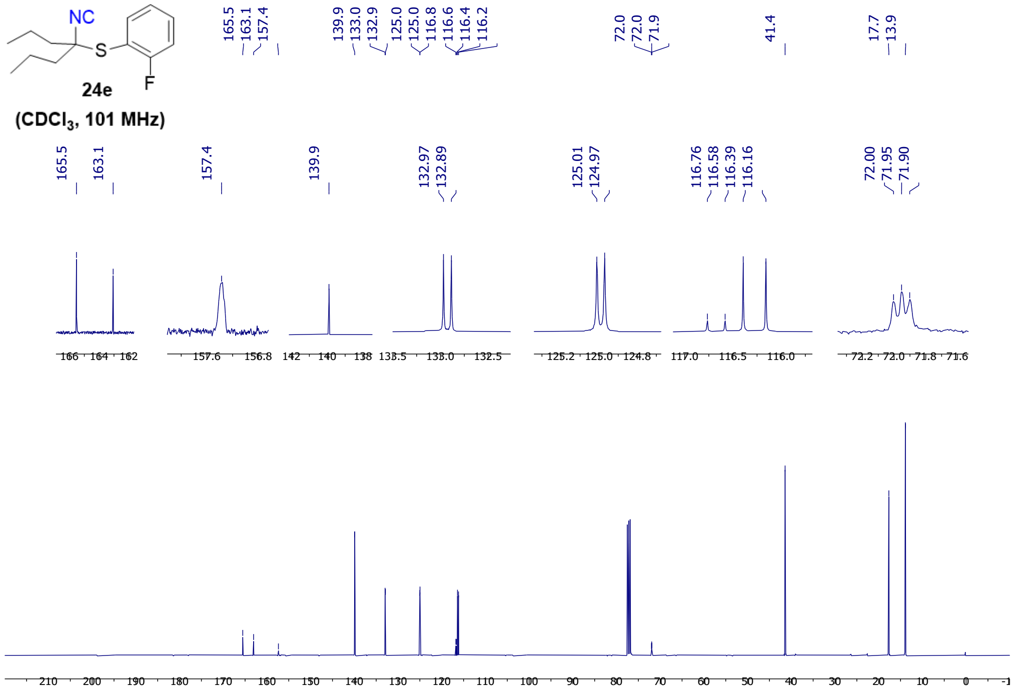

Supplementary Figure 67. ^{1}H NMR of Compound **24f** at 25 °C

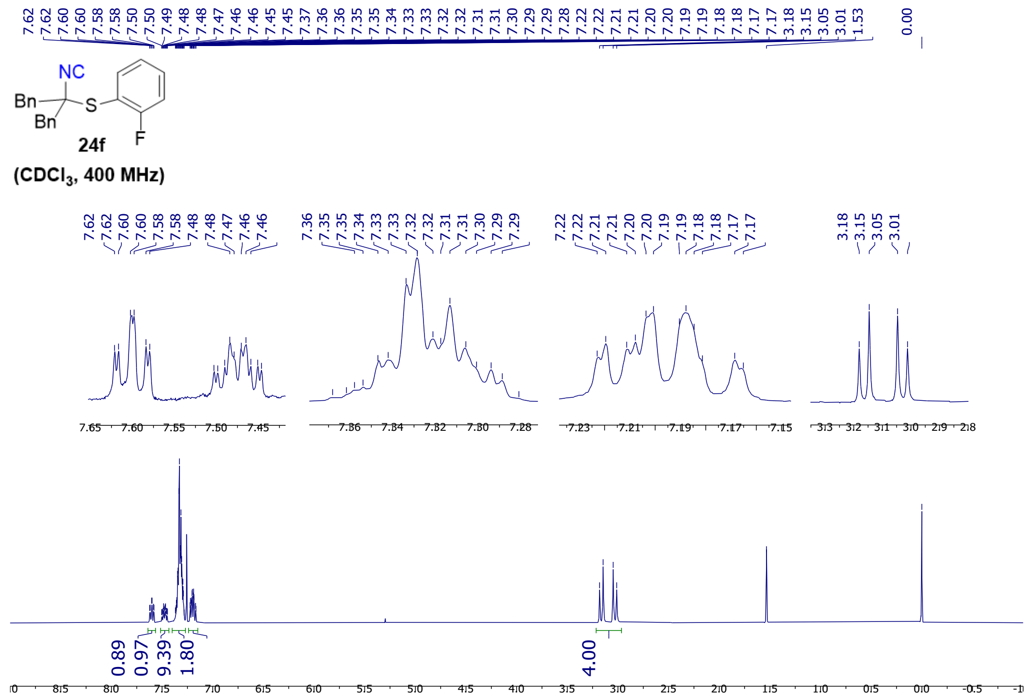

Supplementary Figure 68. ^{13}C NMR of Compound **24f** at 25 °C

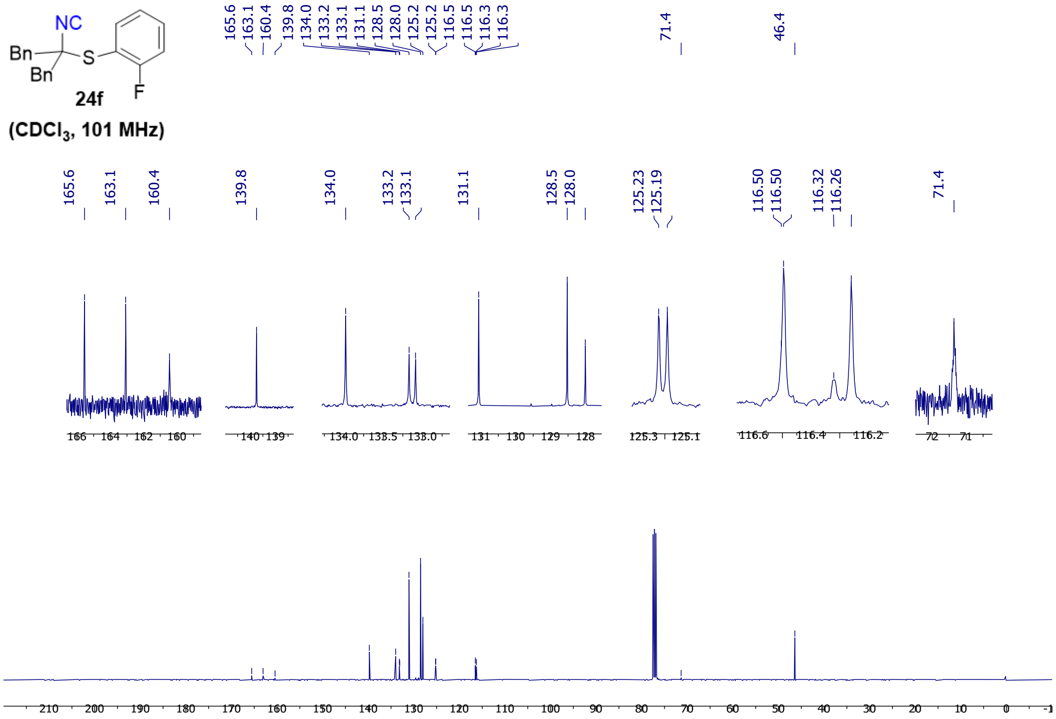

Supplementary Figure 69. ^{1}H NMR of Compound **24g** at 25 °C

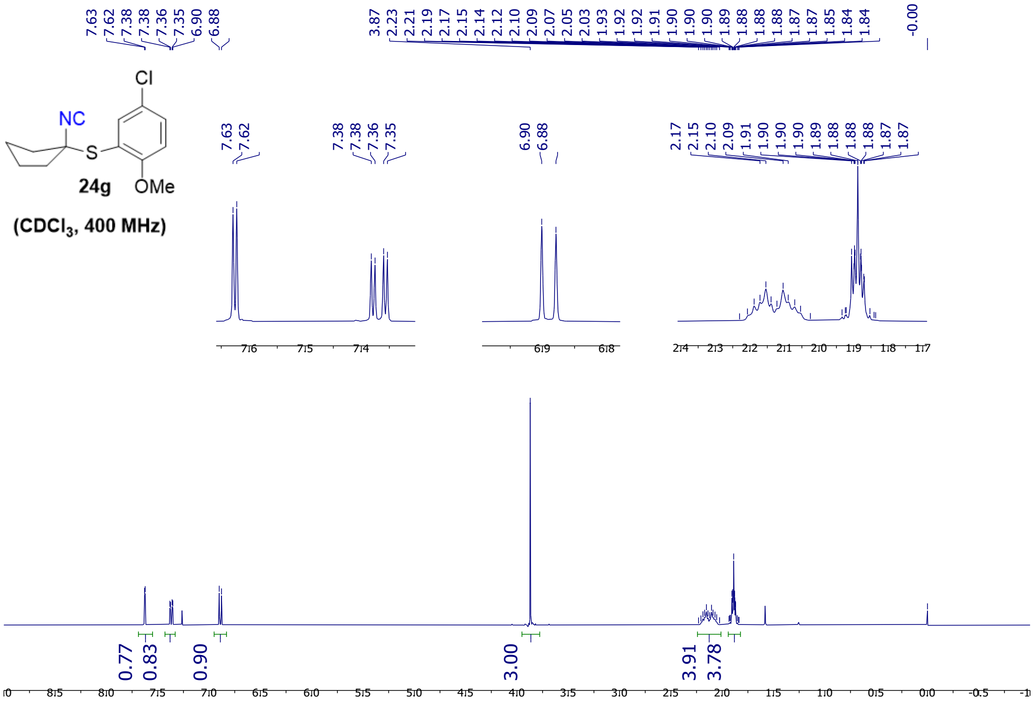

Supplementary Figure 70. ^{13}C NMR of Compound **24g** at 25 °C

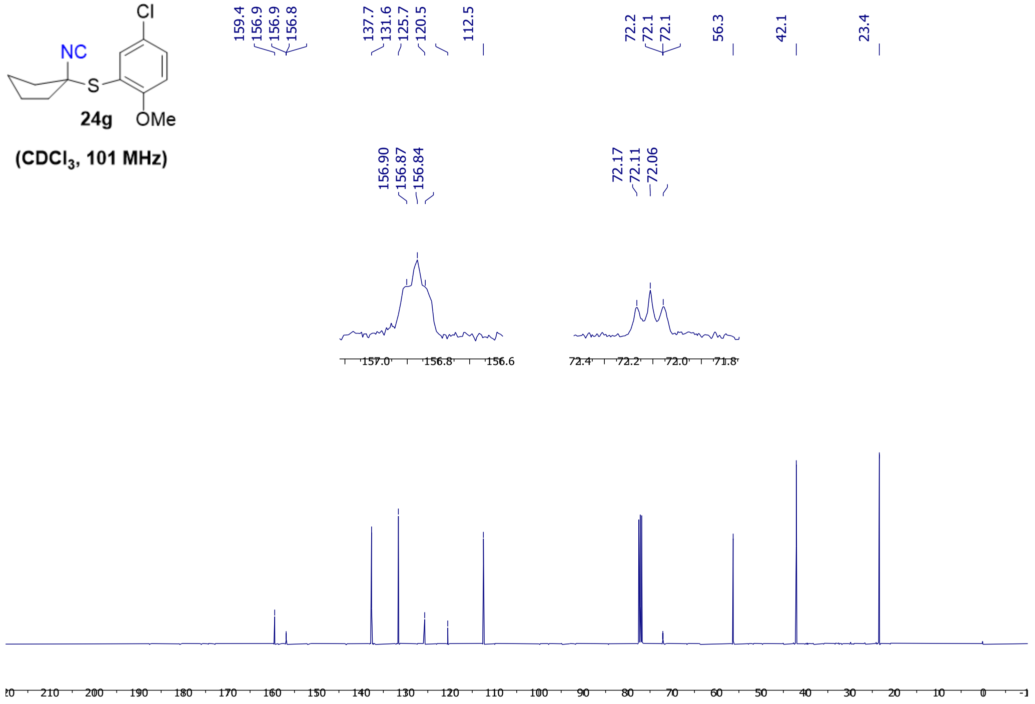

Supplementary Figure 71. ^{1}H NMR of Compound **24h** at 25 °C

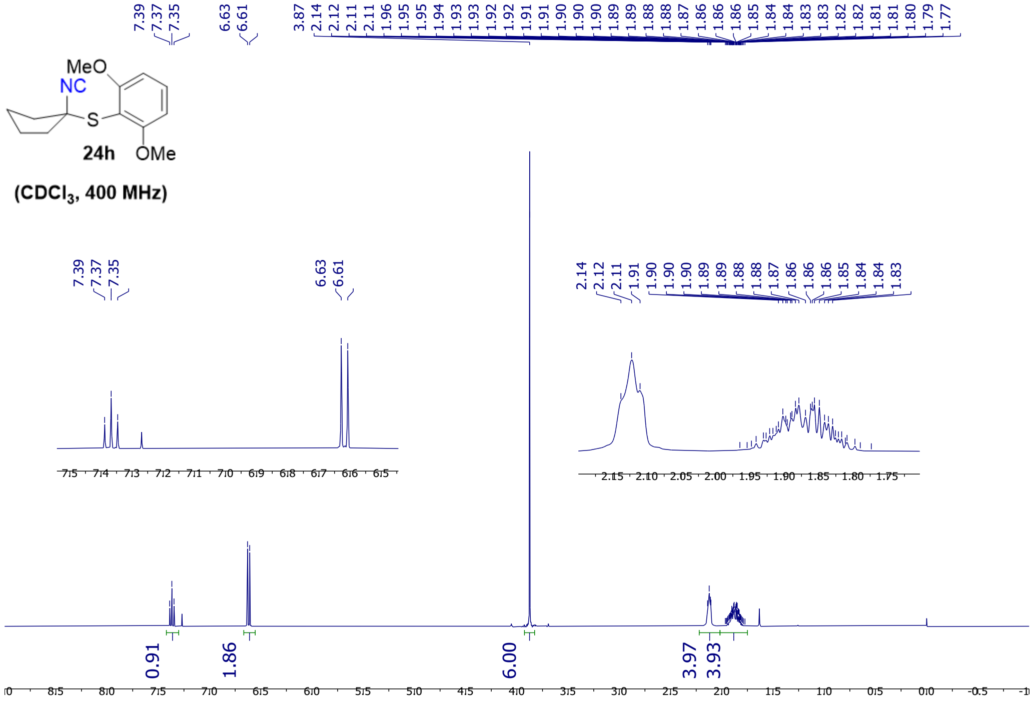

Supplementary Figure 72. ^{13}C NMR of Compound **24h** at 25 °C

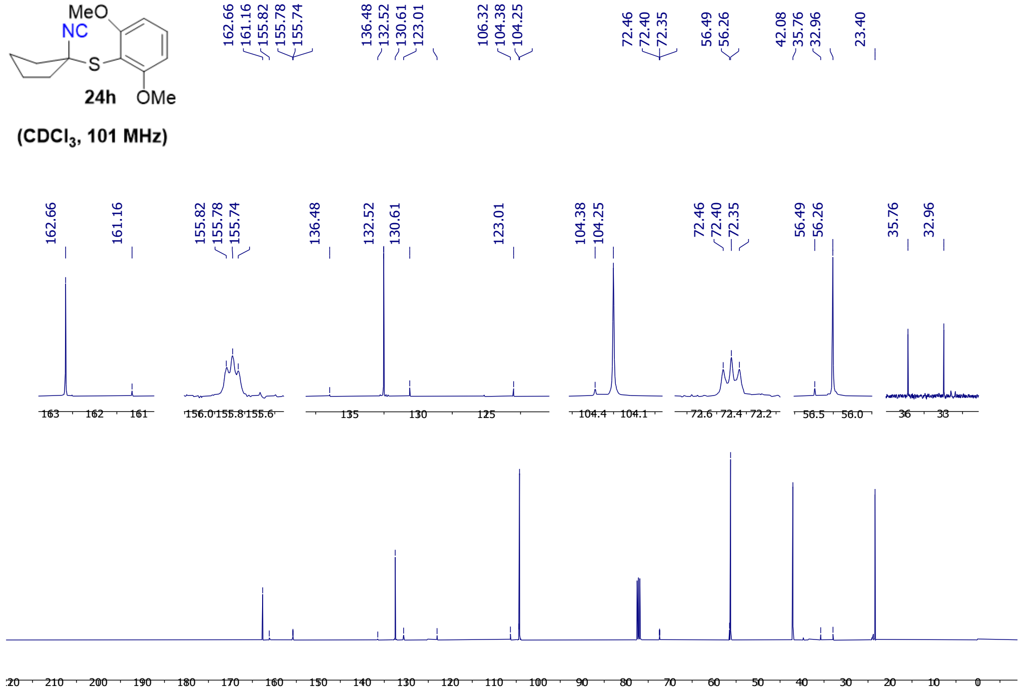

Supplementary Figure 73. ^{1}H NMR of Compound **24i** at 25 °C

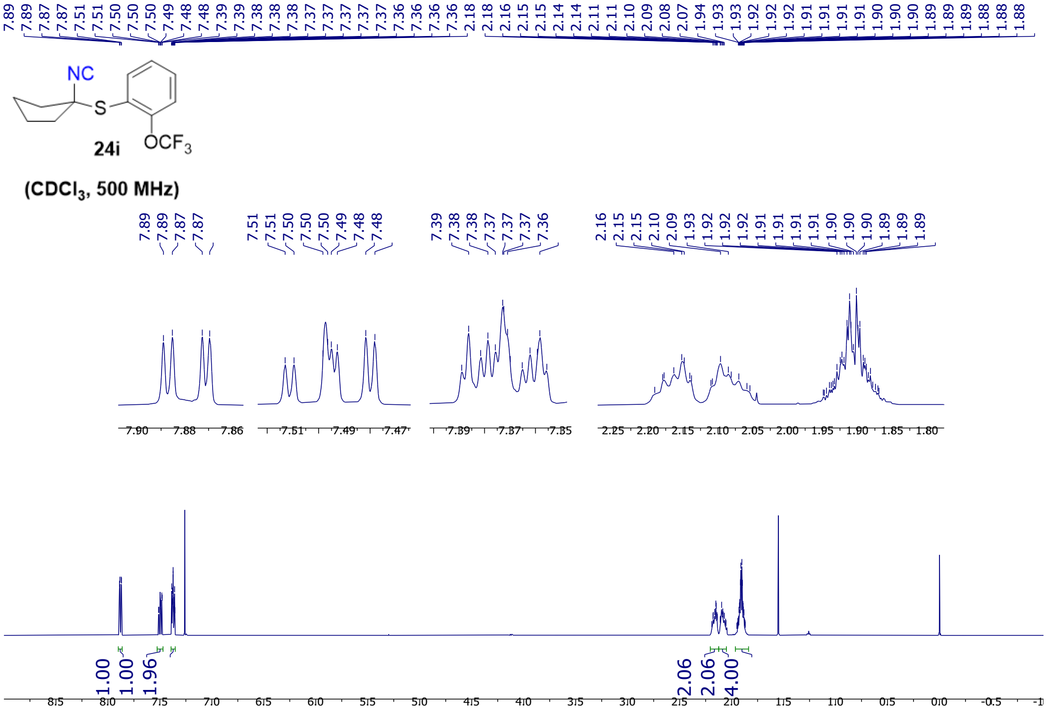

Supplementary Figure 74. ^{13}C NMR of Compound **24i** at 25 °C

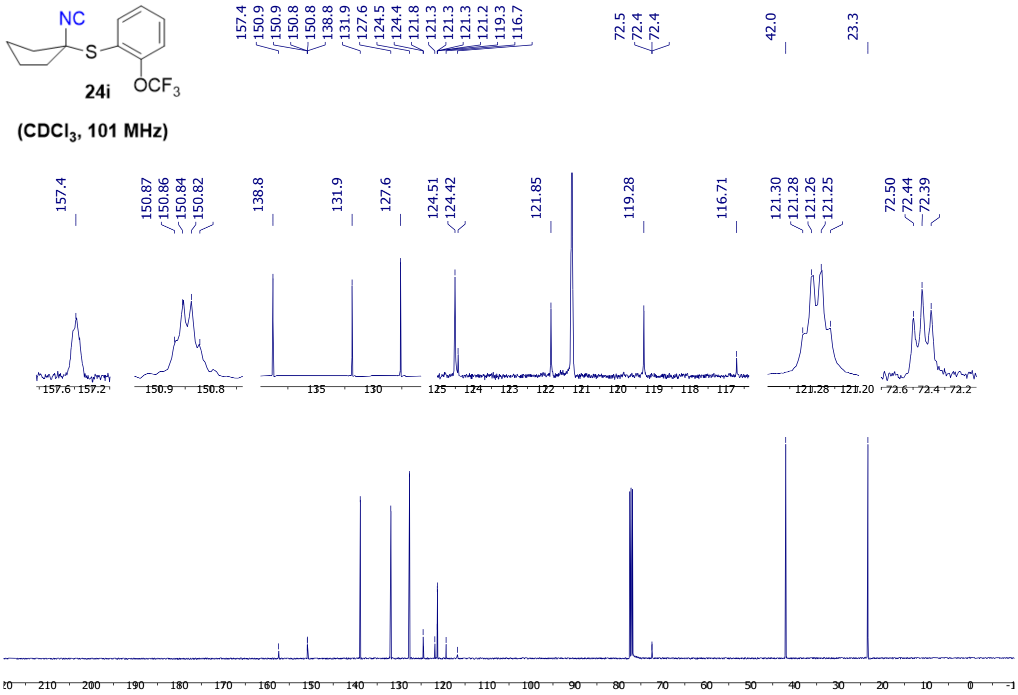

Supplementary Figure 75. ^{1}H NMR of Compound **24j** at 25 °C

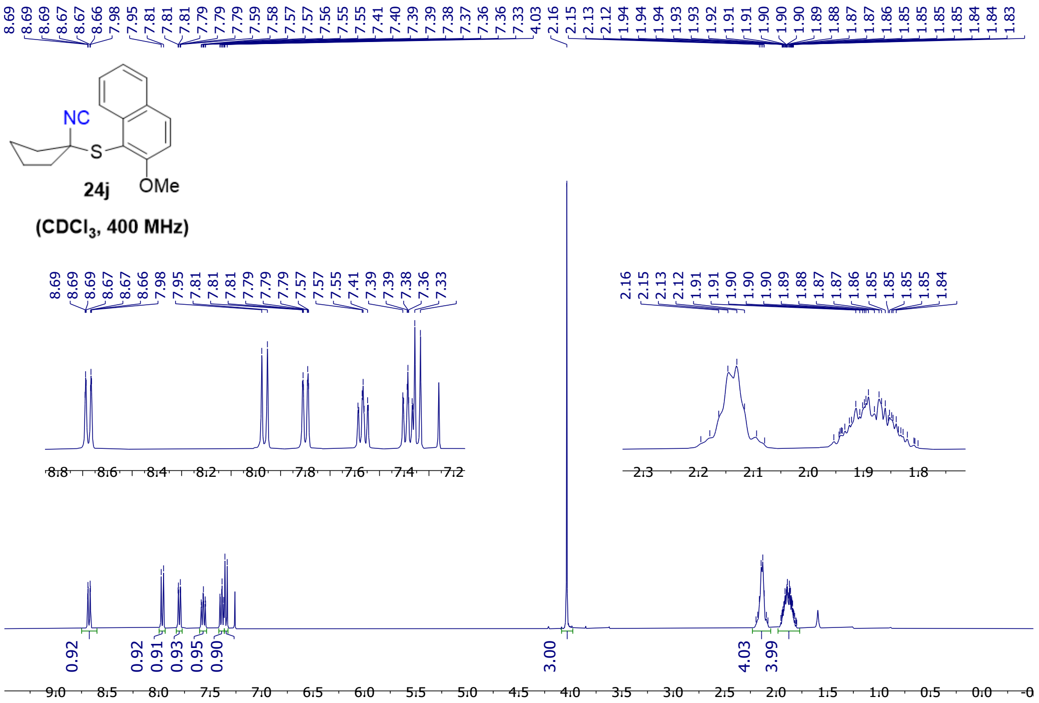

Supplementary Figure 76. ^{13}C NMR of Compound **24j** at 25 °C

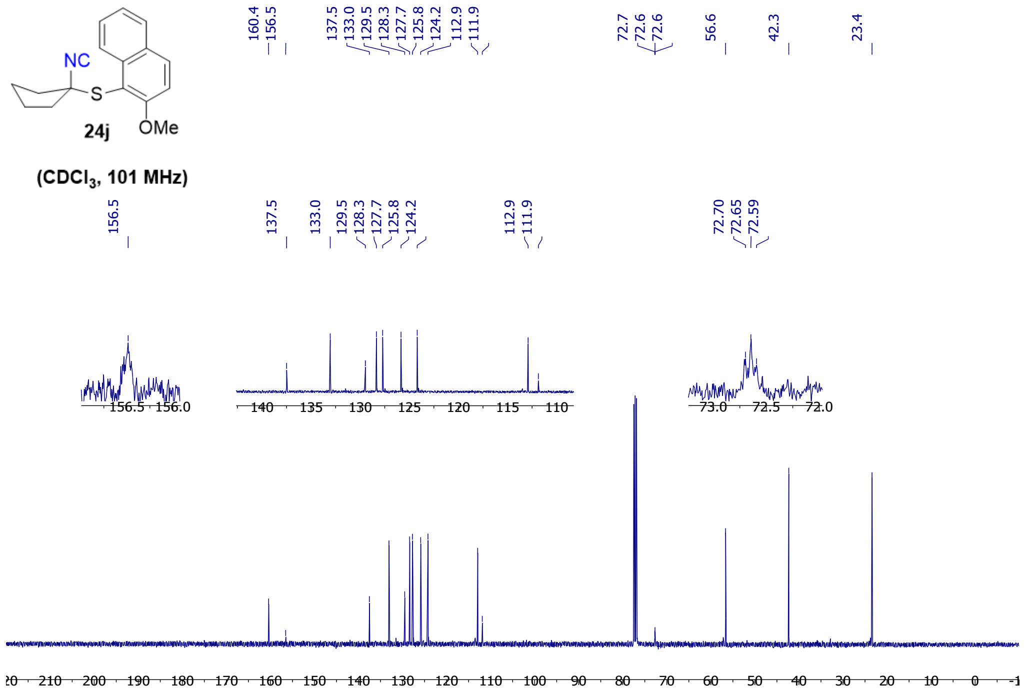

Supplementary Figure 77. ^{1}H NMR of Compound **24k** at 25 °C

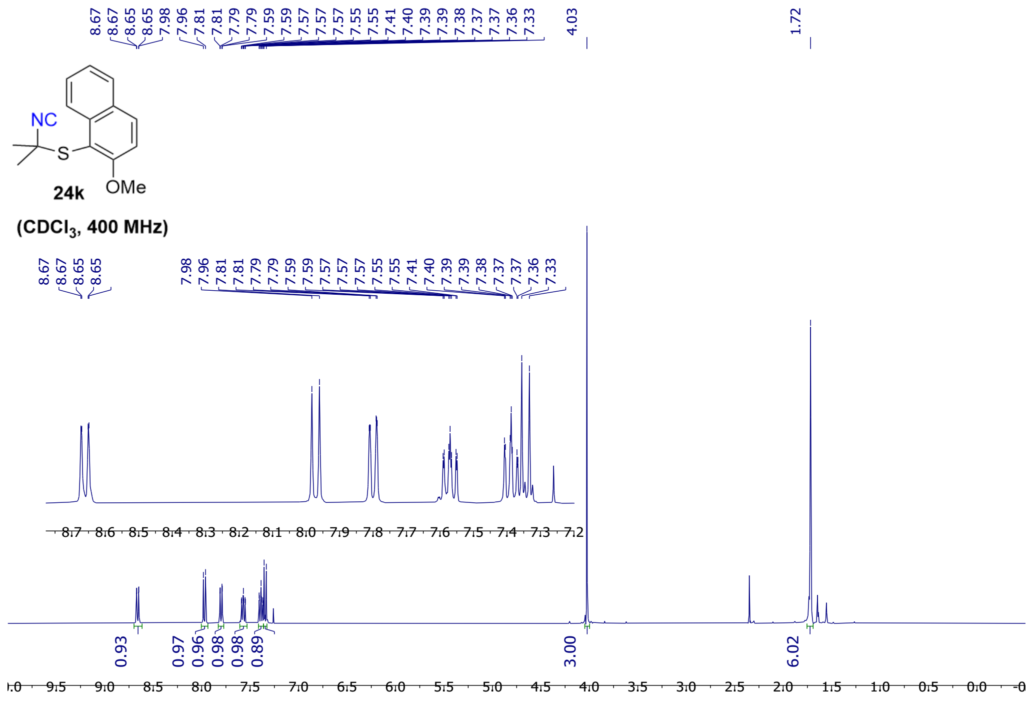

Supplementary Figure 78. ^{13}C NMR of Compound **24k** at 25 °C

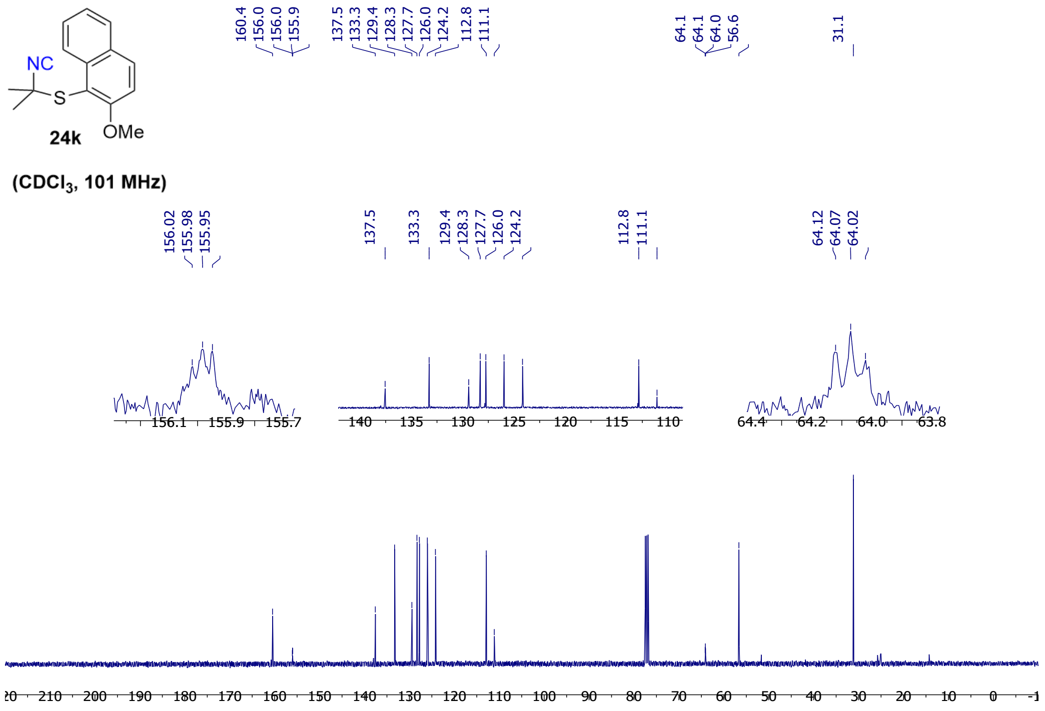

Supplementary Figure 79. ^{1}H NMR of Compound **25a** at 25 °C

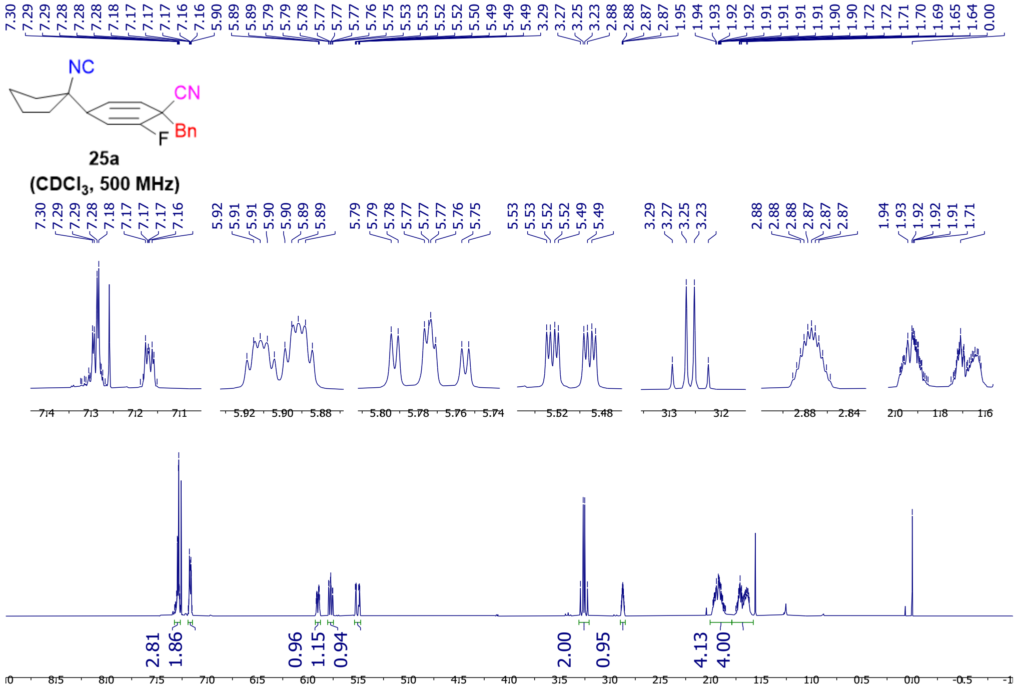

Supplementary Figure 80. ^{13}C NMR of Compound **25a** at 25 °C

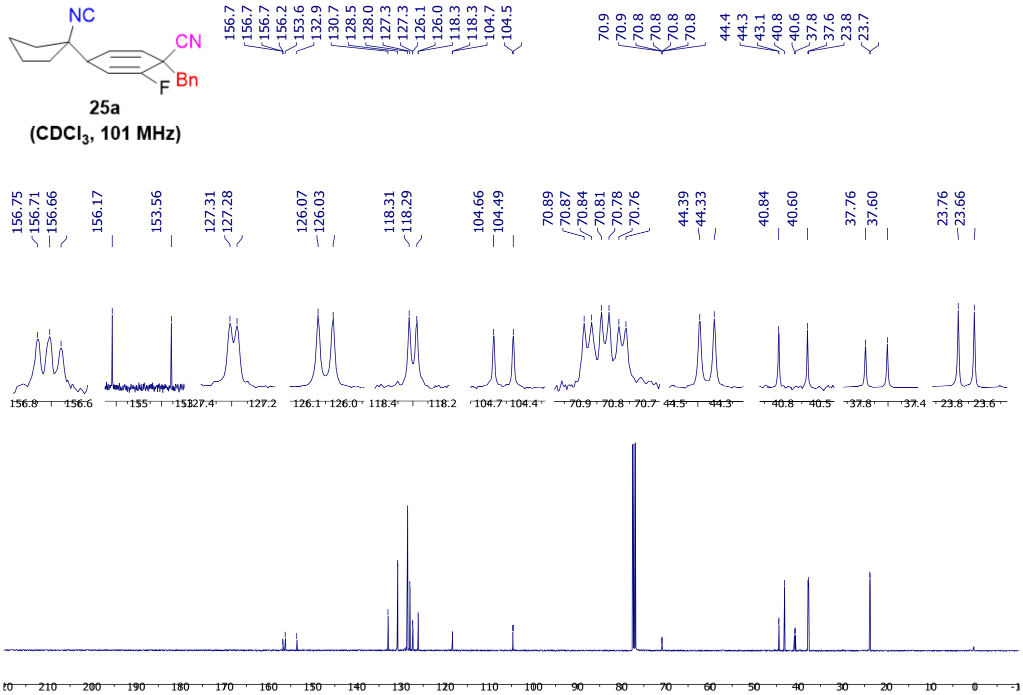

Supplementary Figure 81. ^{1}H NMR of Compound **25b** at 25 °C

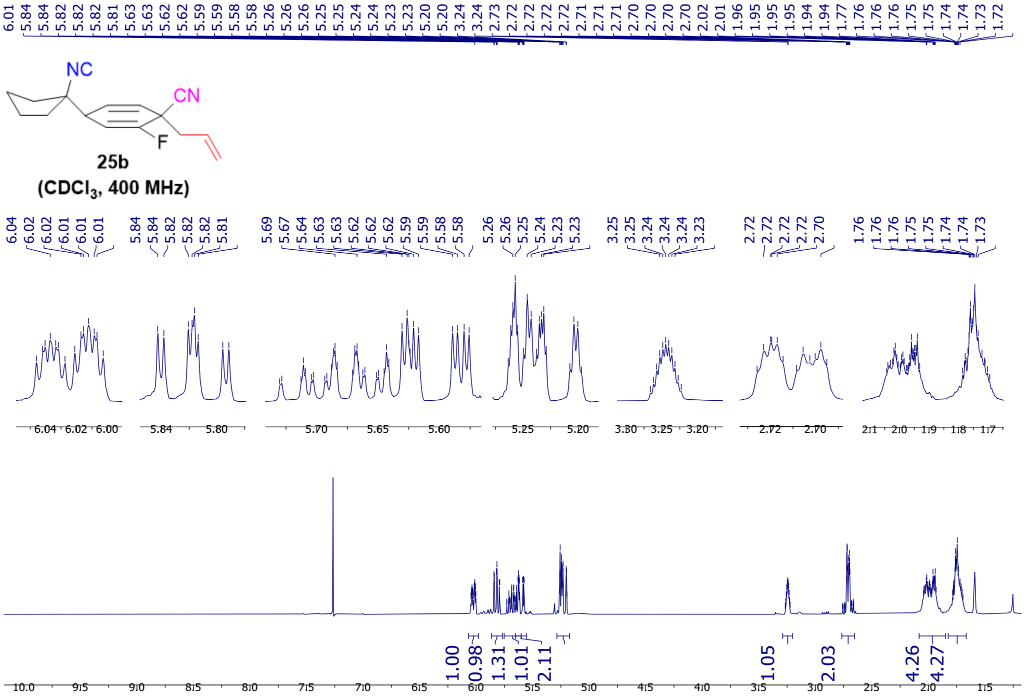

Supplementary Figure 82. ^{13}C NMR of Compound **25b** at 25 °C

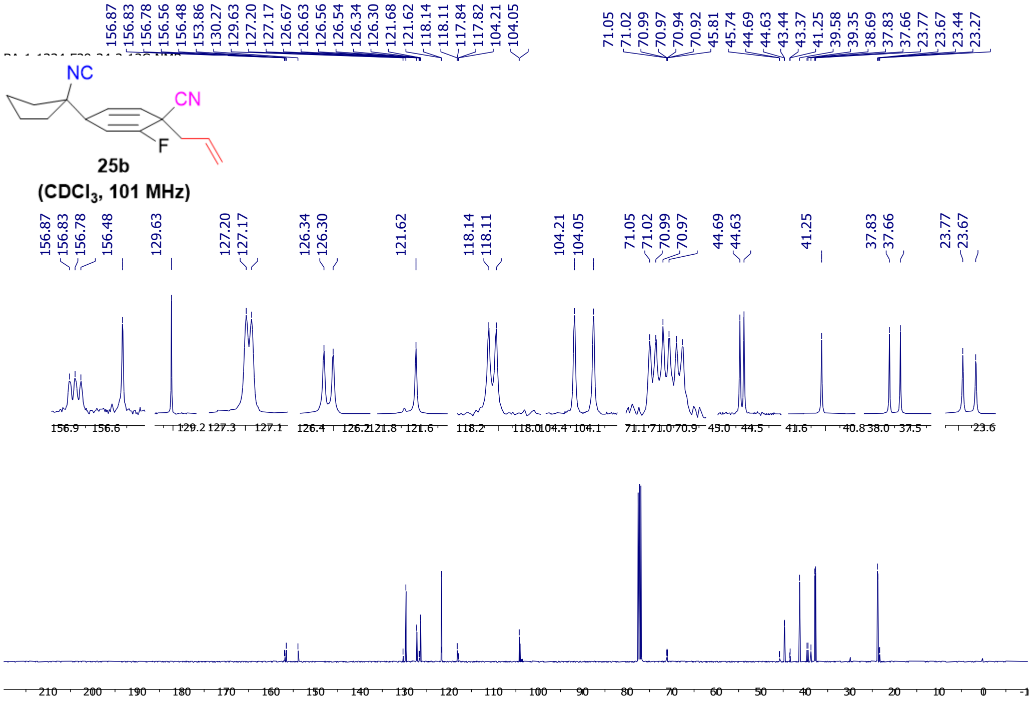

Supplementary Figure 83. ^{1}H NMR of Compound **25c** at 25 °C

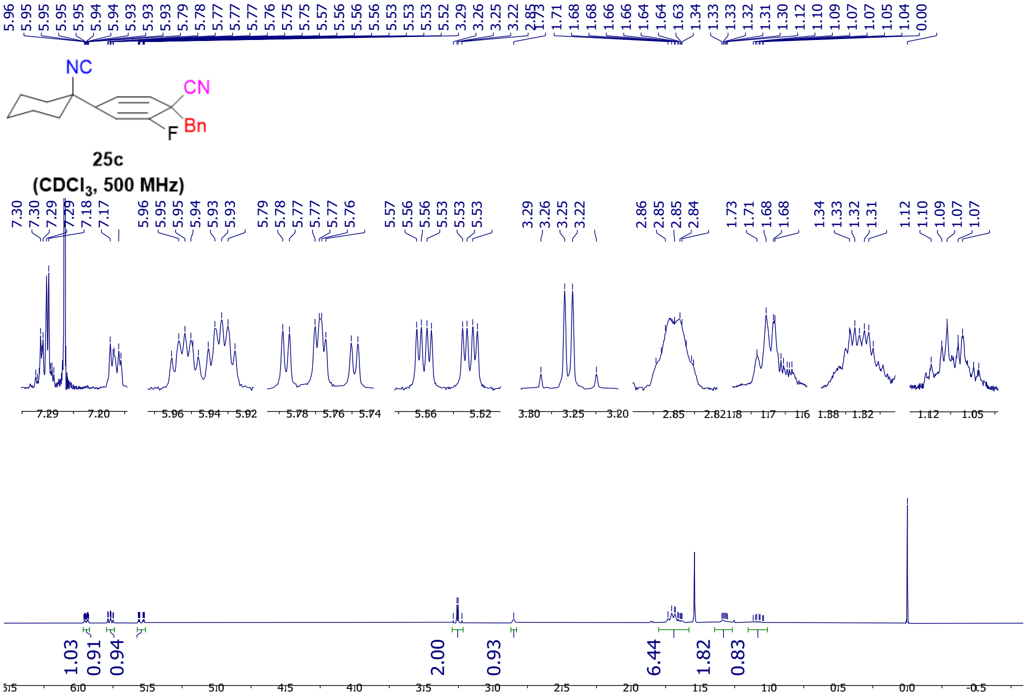

Supplementary Figure 84. ^{13}C NMR of Compound **25c** at 25 °C

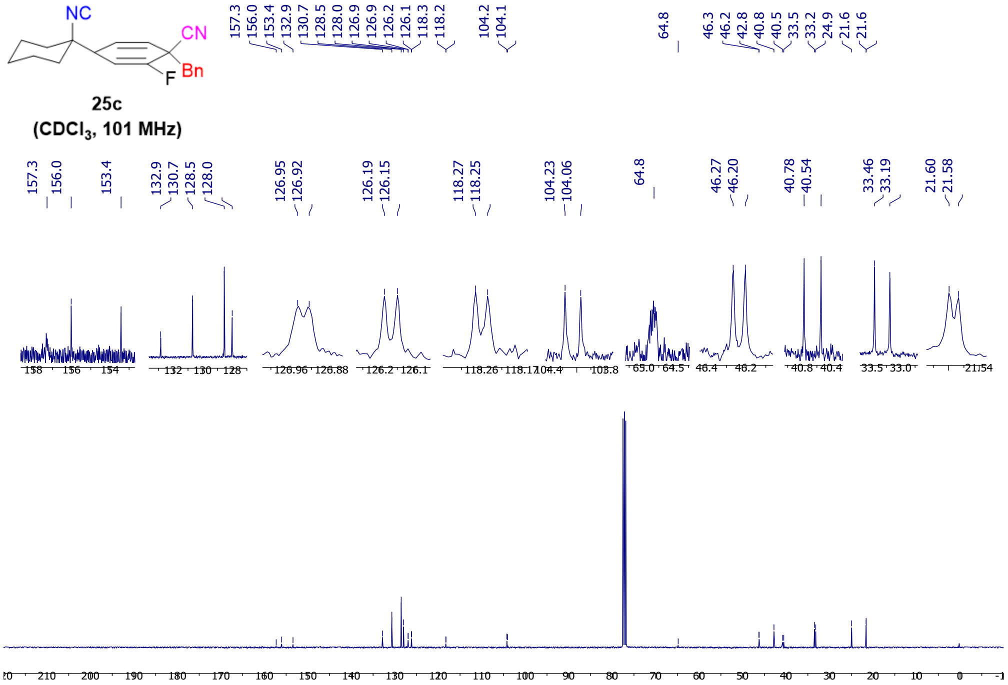

Supplementary Figure 85. ^{1}H NMR of Compound **25d** at 25 °C

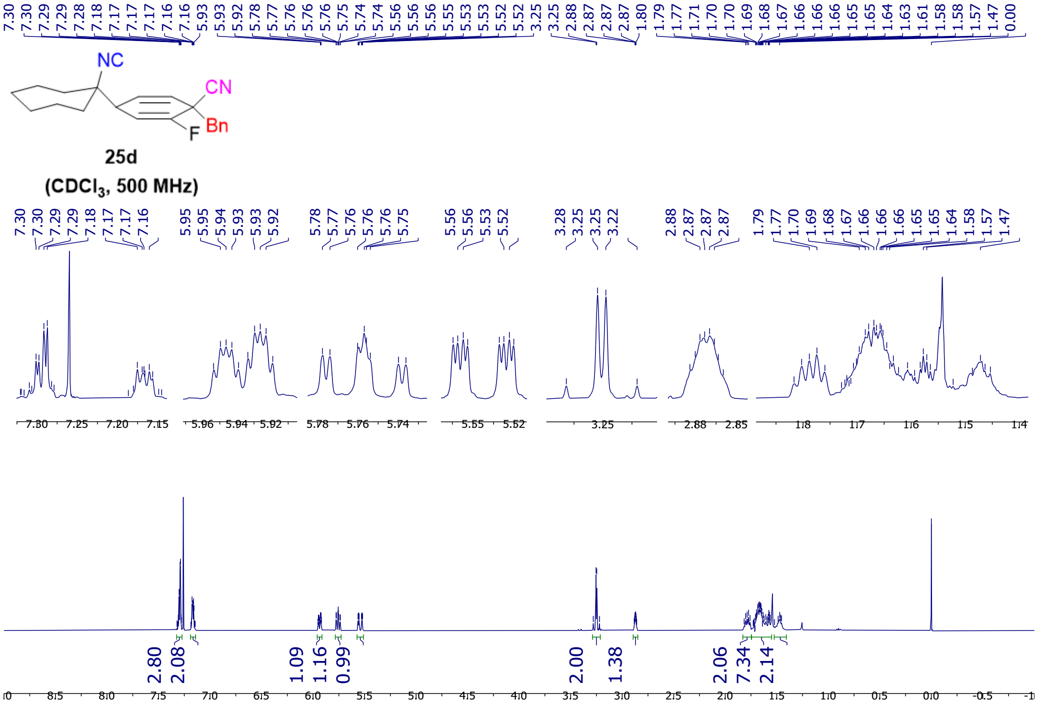

Supplementary Figure 86. ^{13}C NMR of Compound **25d** at 25 °C

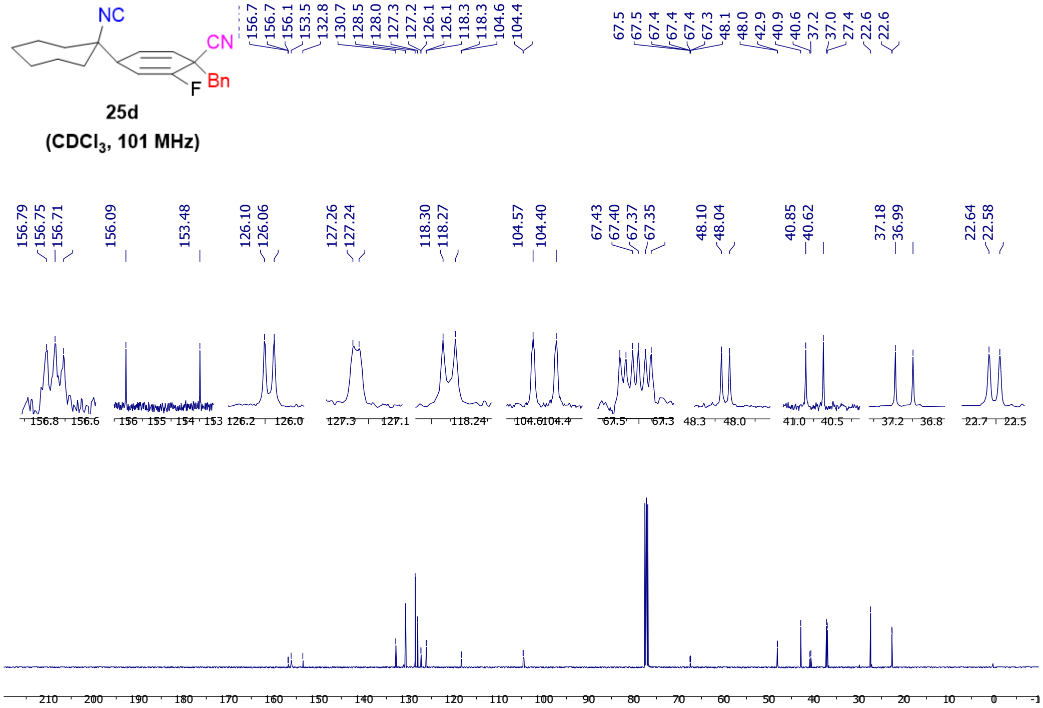

Supplementary Figure 87. ^{1}H NMR of Compound **25e** at 25 °C

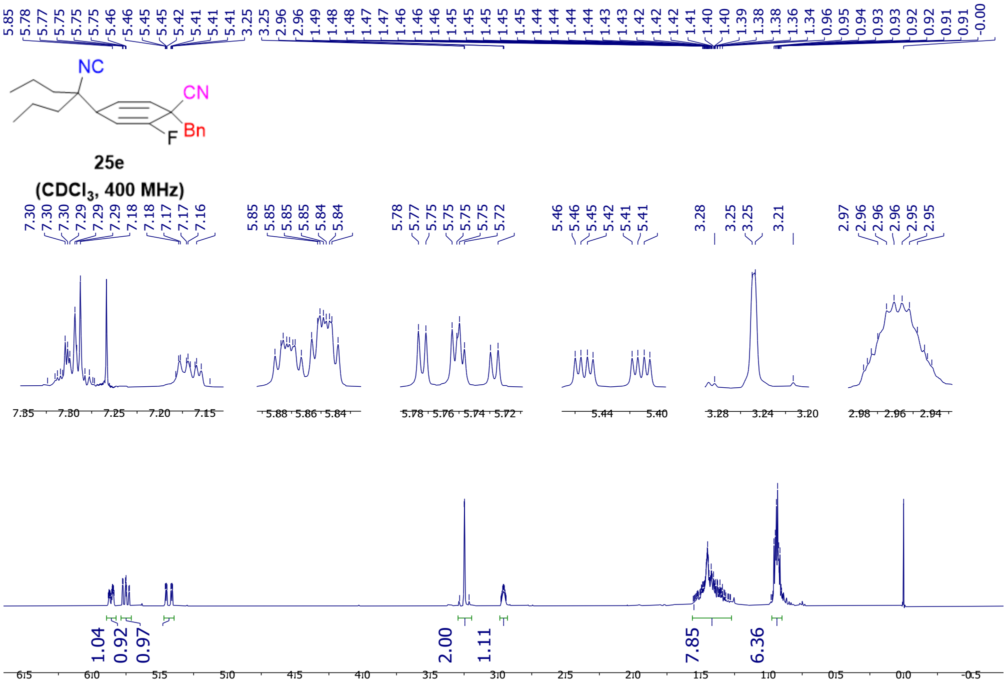

Supplementary Figure 88. ^{13}C NMR of Compound **25e** at 25 °C

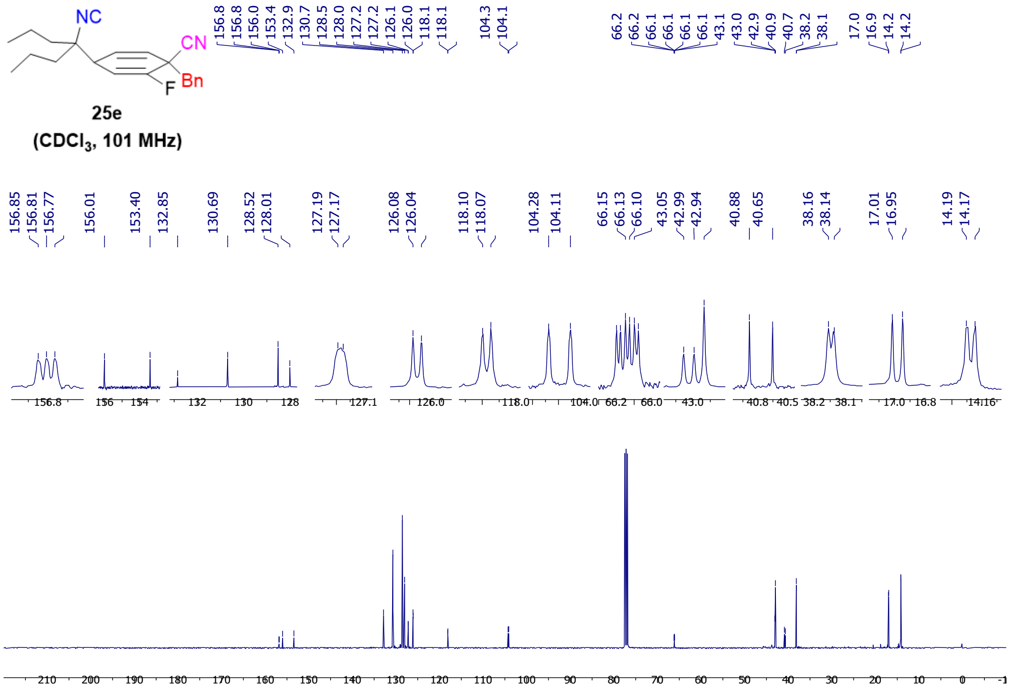

Supplementary Figure 89. ^{1}H NMR of Compound **25f** at 25 °C

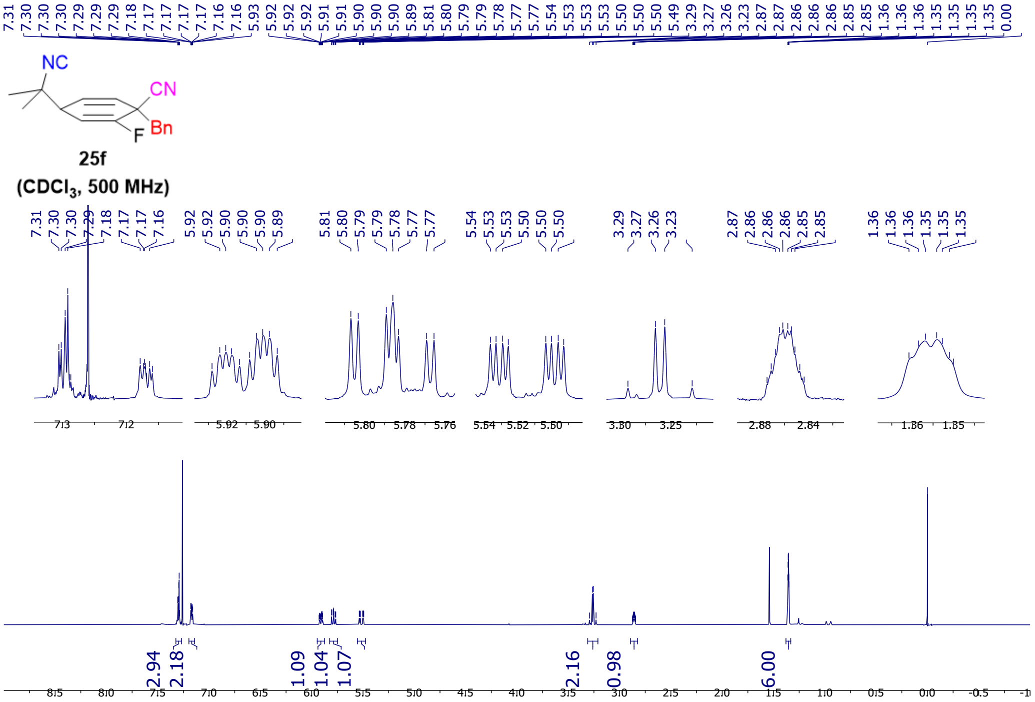

Supplementary Figure 90. ^{13}C NMR of Compound **25f** at 25 °C

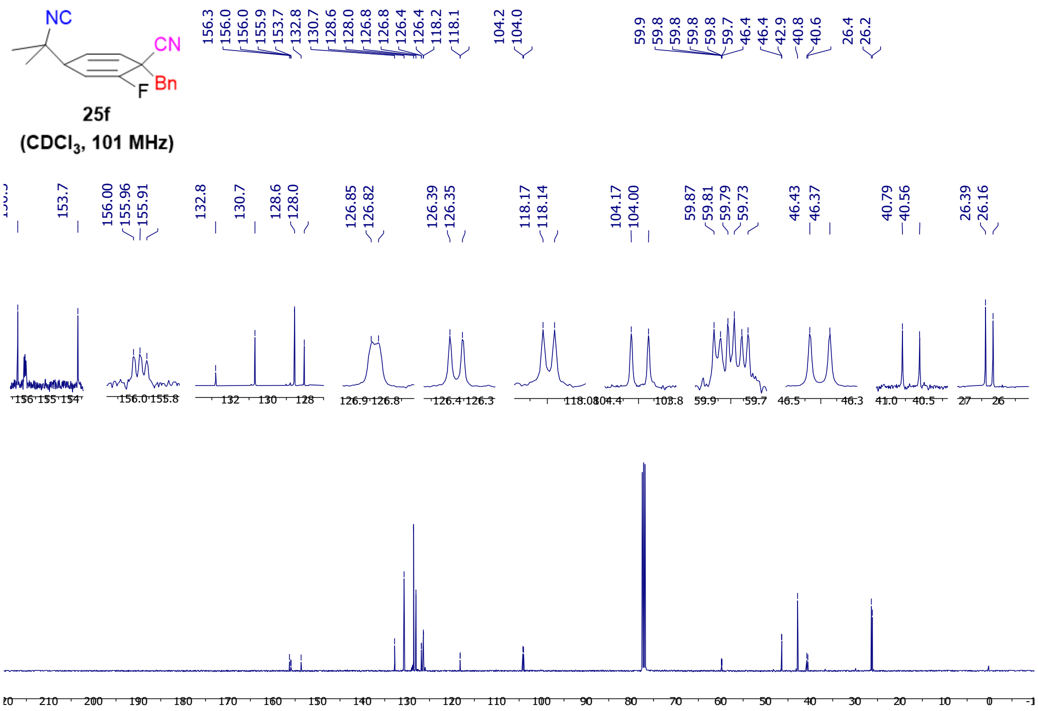

Supplementary Figure 91. ¹H NMR of Compound **25g** at 25 °C

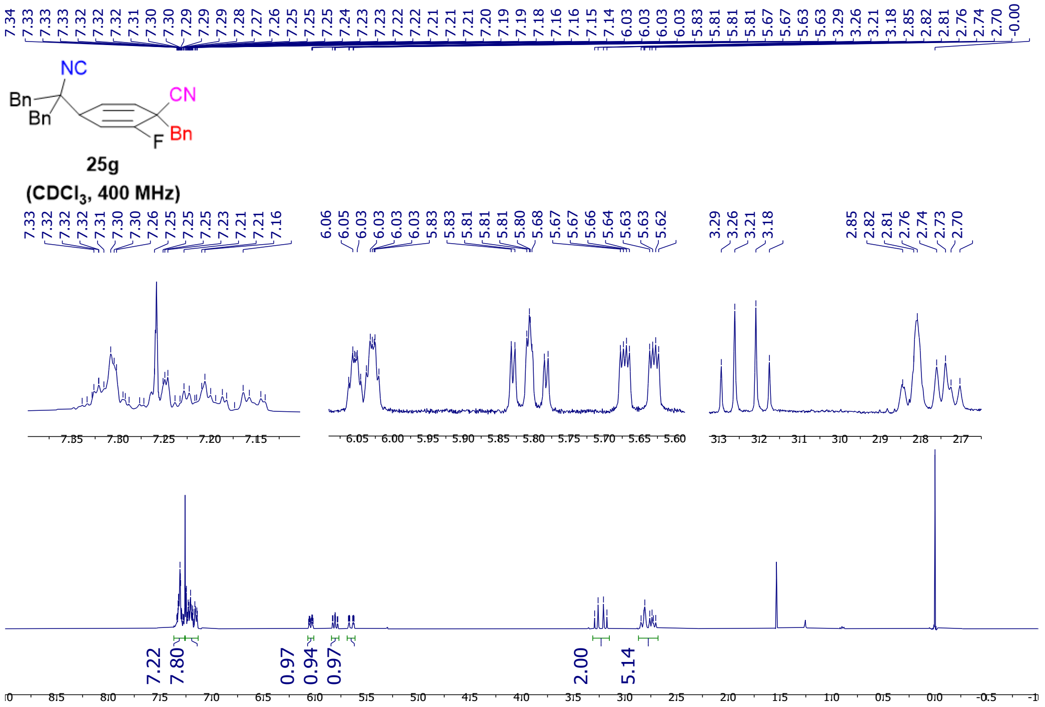

Supplementary Figure 92. ¹³C NMR of Compound **25g** at 25 °C

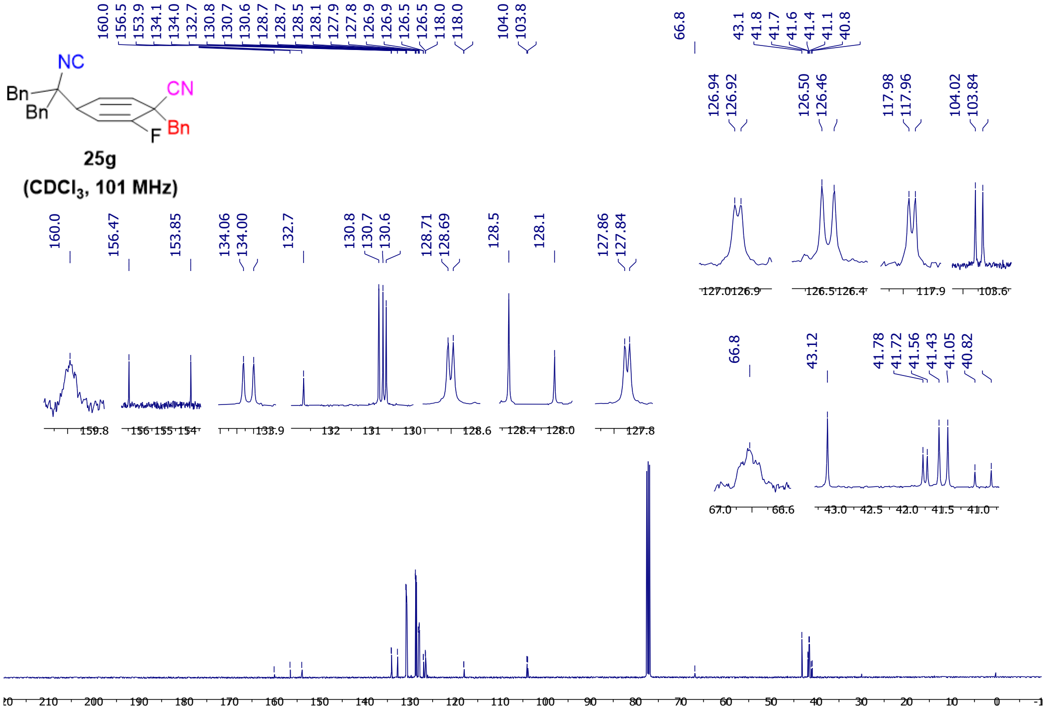

Supplementary Figure 93. ^{1}H NMR of Compound **25h** at 25 °C

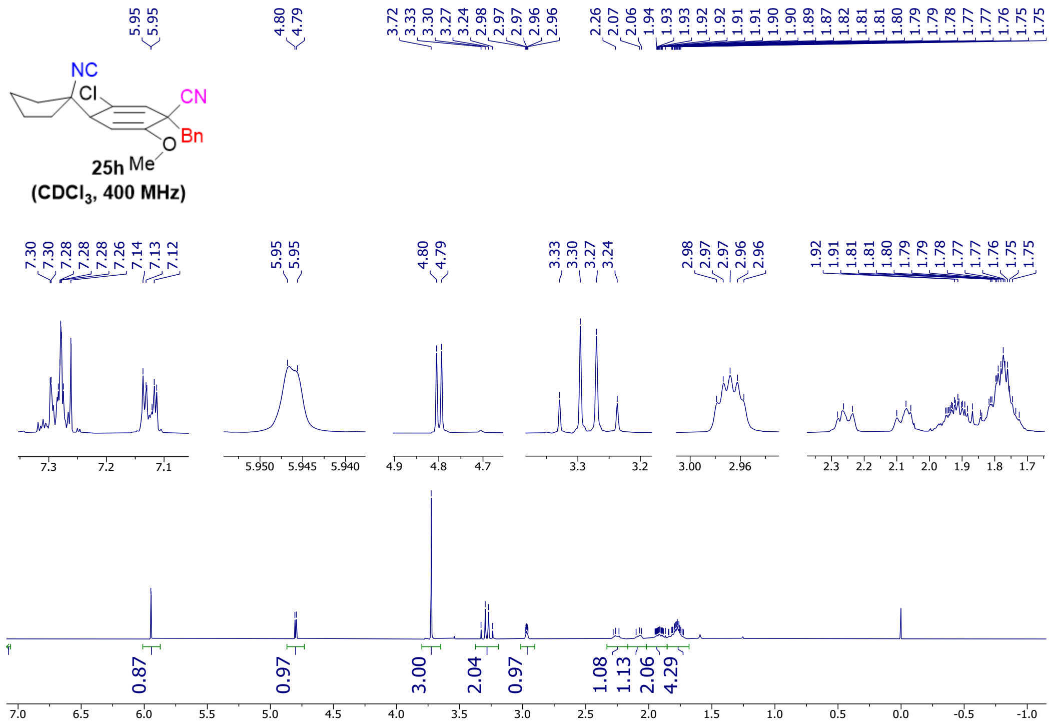

Supplementary Figure 94. ^{13}C NMR of Compound **25h** at 25 °C

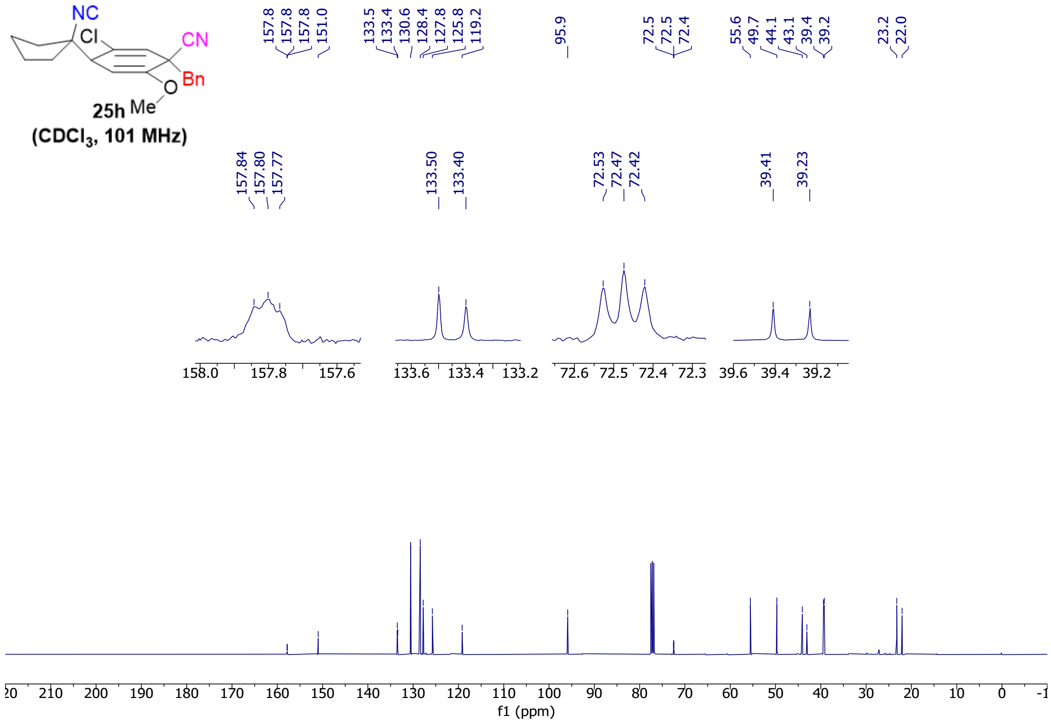

Supplementary Figure 95. ^{1}H NMR of Compound **25i** at 25 °C

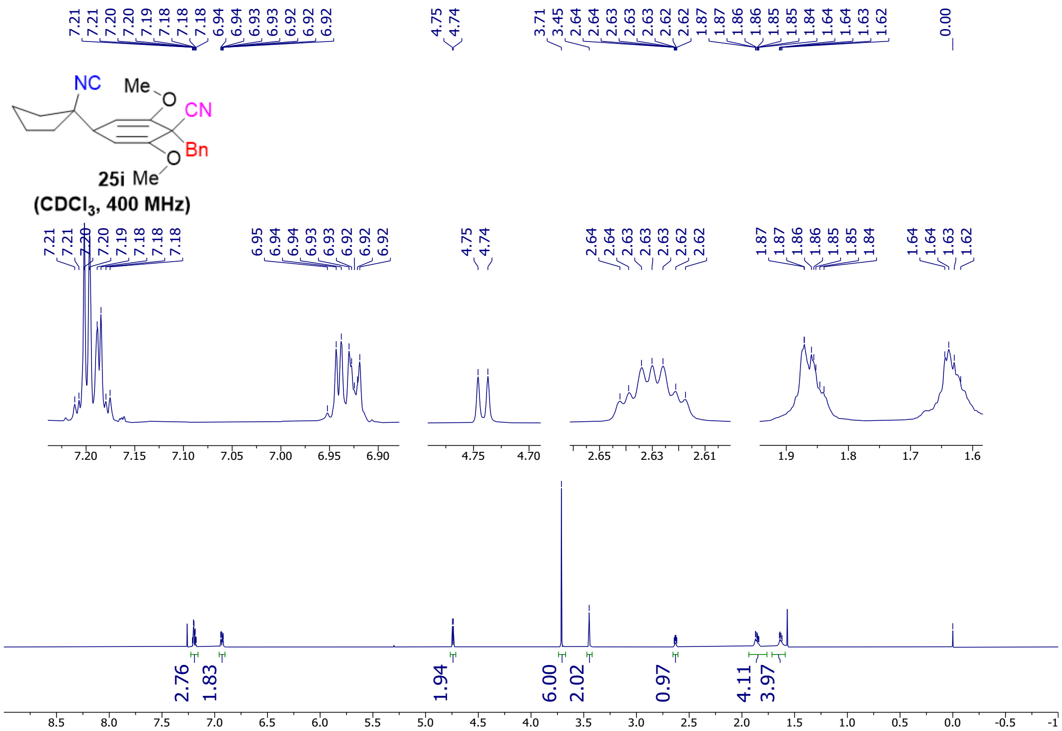

Supplementary Figure 96. ^{13}C NMR of Compound **25i** at 25 °C

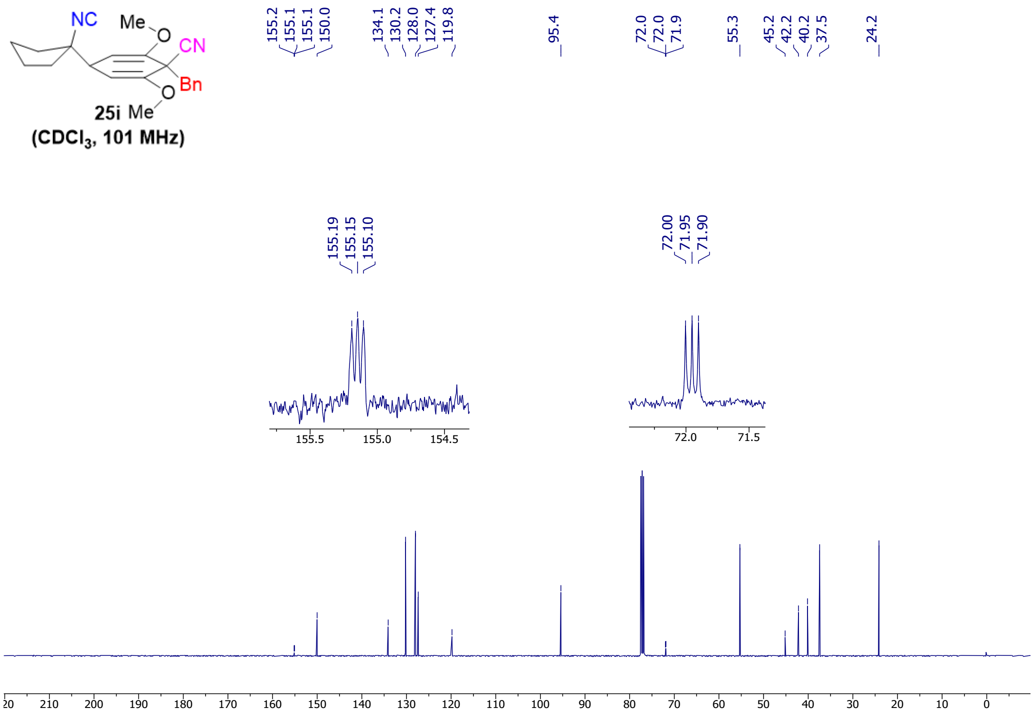

Supplementary Figure 97. ^{1}H NMR of Compound **25j** at 25 °C

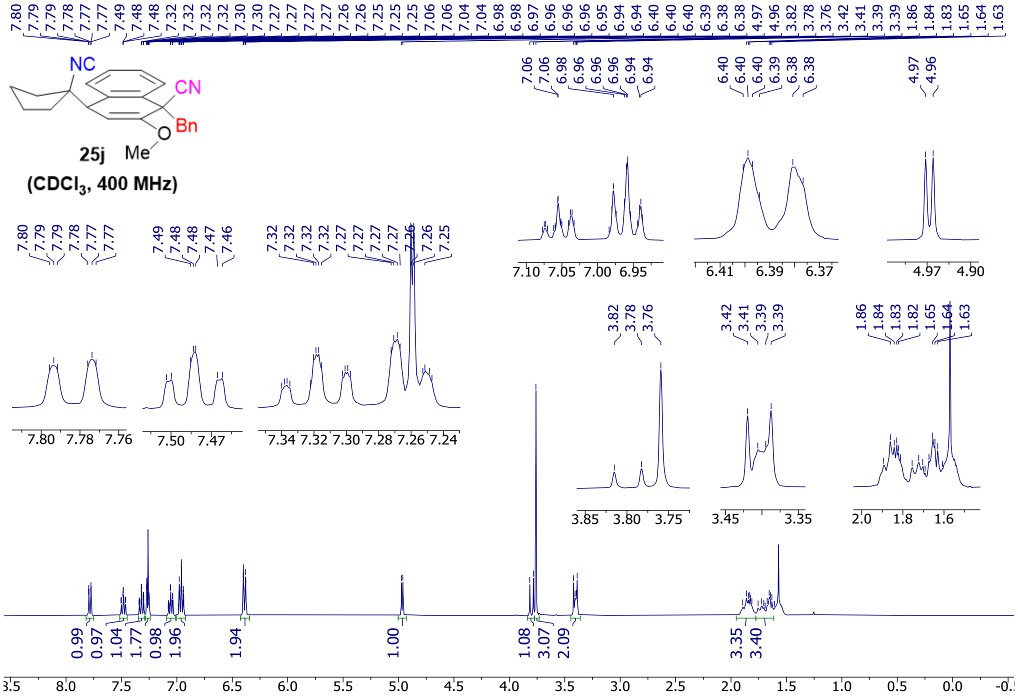

Supplementary Figure 98. ^{13}C NMR of Compound **25j** at 25 °C

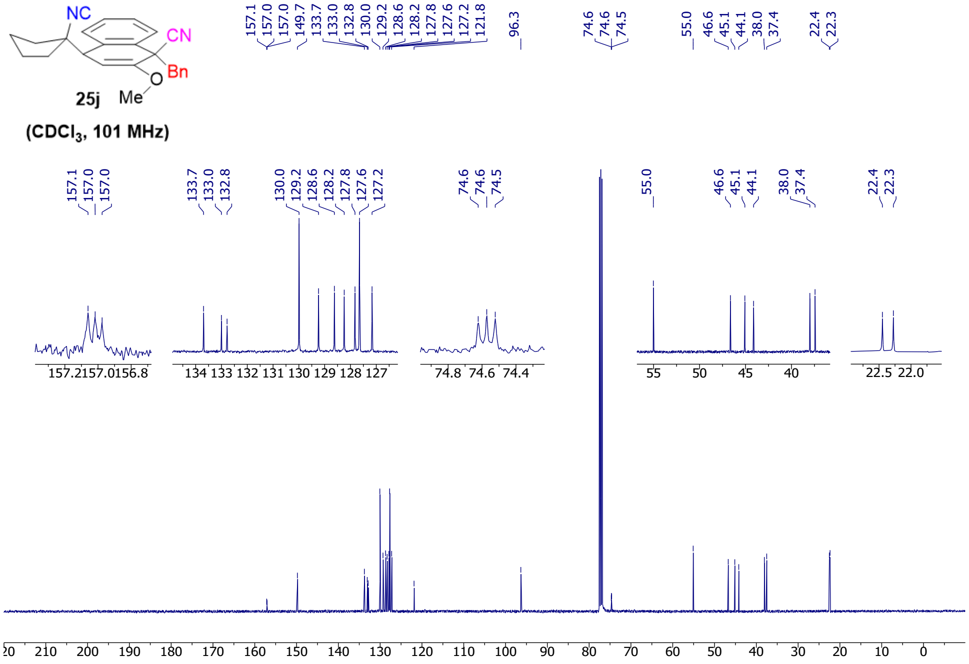

Supplementary Figure 99. ^{1}H NMR of Compound **25k** at 25 °C

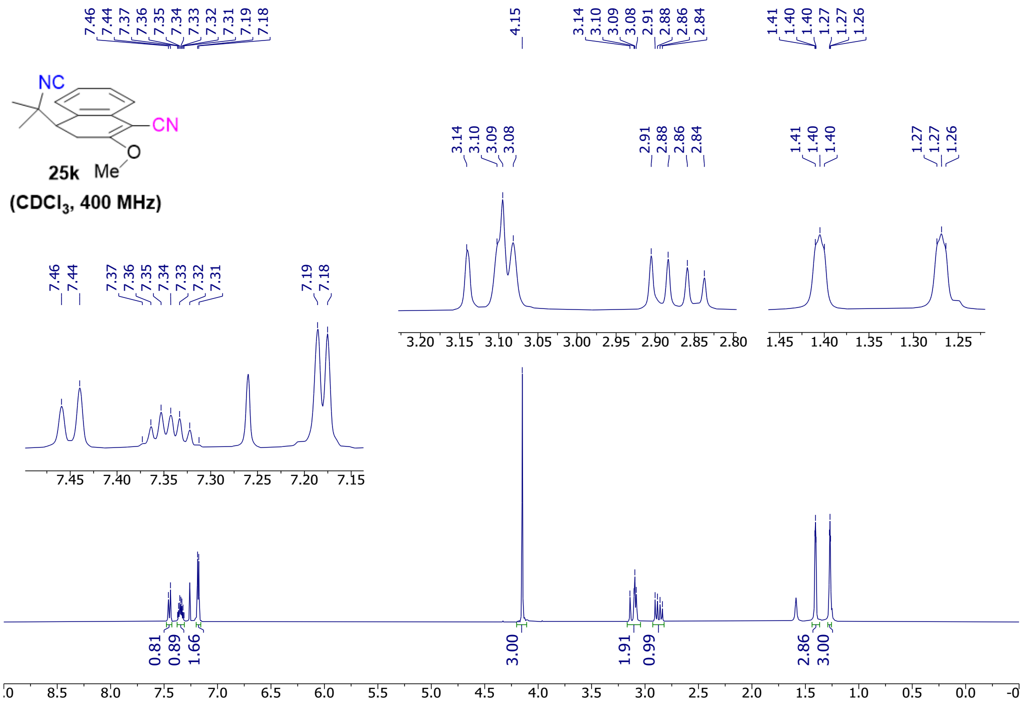

Supplementary Figure 100. ^{13}C NMR of Compound **25k** at 25 °C

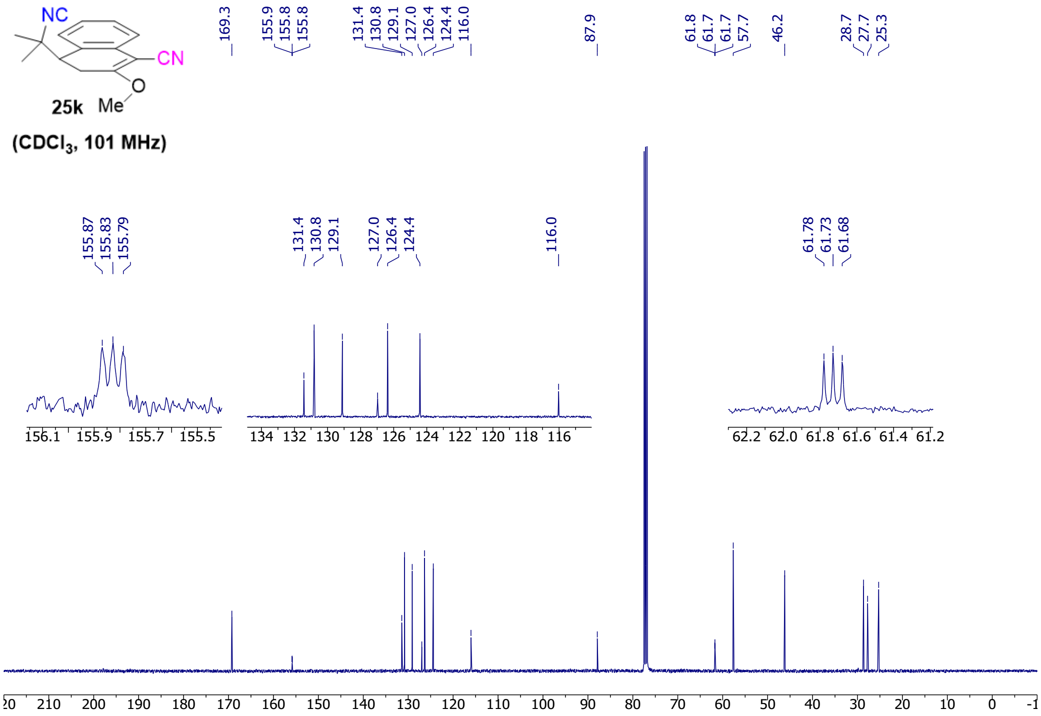

Supplementary Figure 101. ^{1}H NMR of Compound **25l** at 25 °C

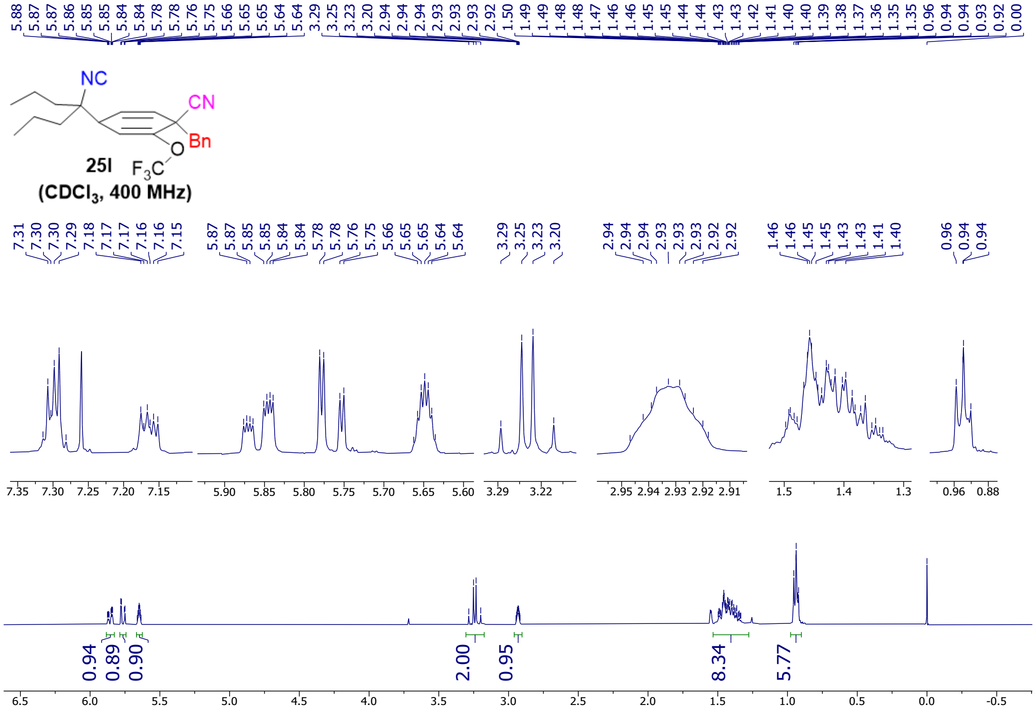

Supplementary Figure 102. ^{13}C NMR of Compound **25l** at 25 °C

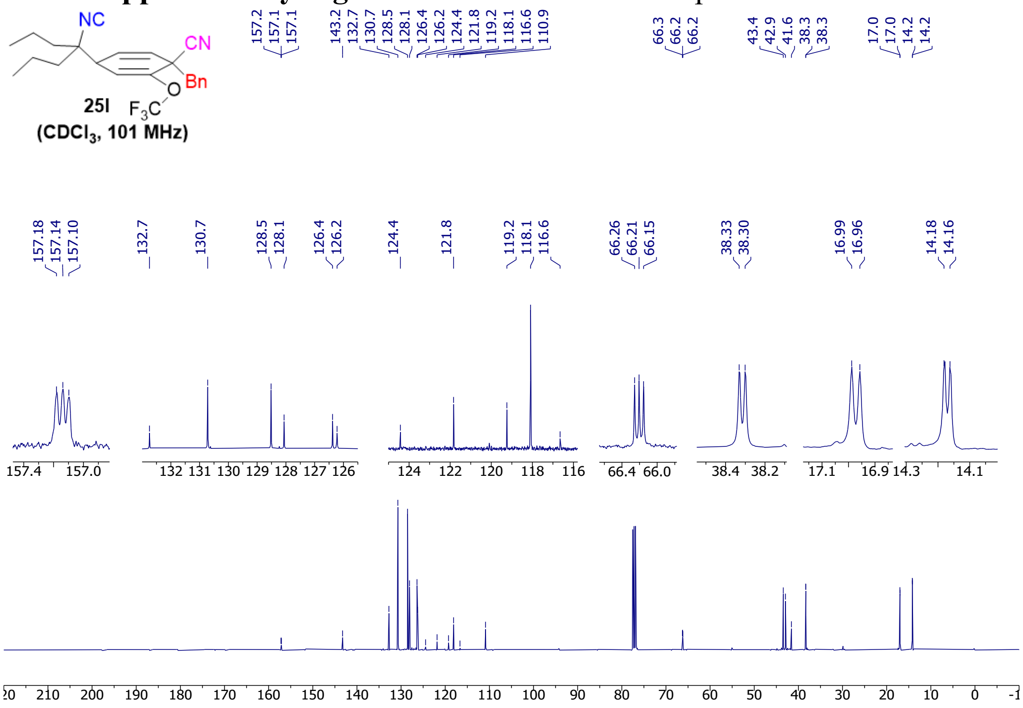

Supplementary Figure 103. ¹H NMR of Compound **27** at 25 °C

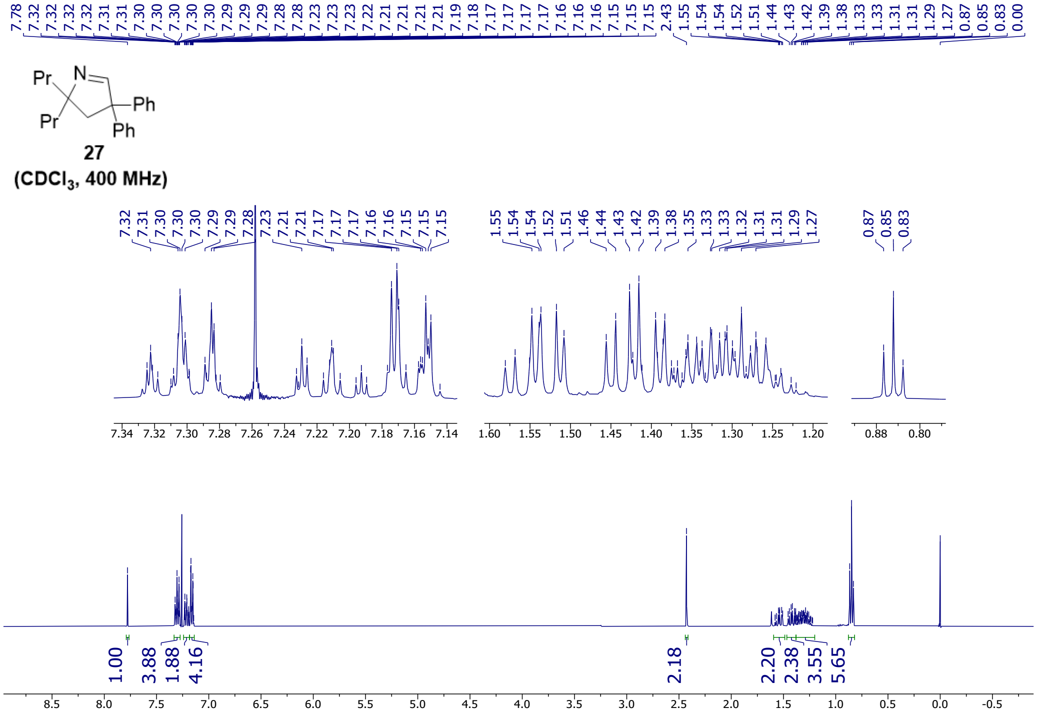

Supplementary Figure 104. ¹³C NMR of Compound **27** at 25 °C

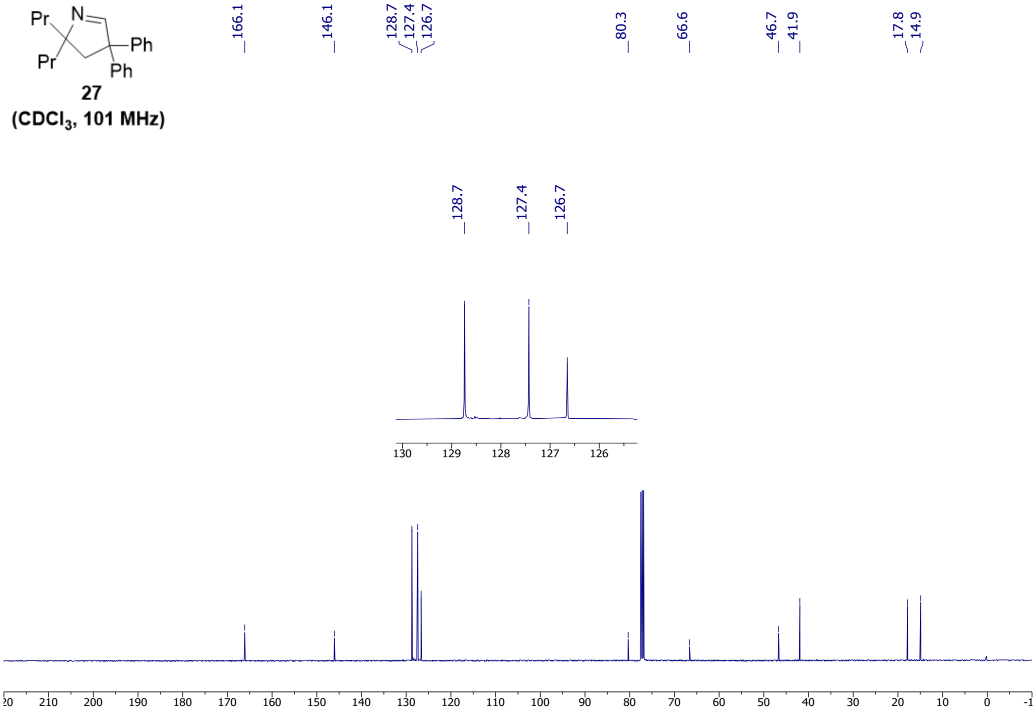

Supplementary Figure 105. ^{1}H NMR of Compound **28** at 25 °C

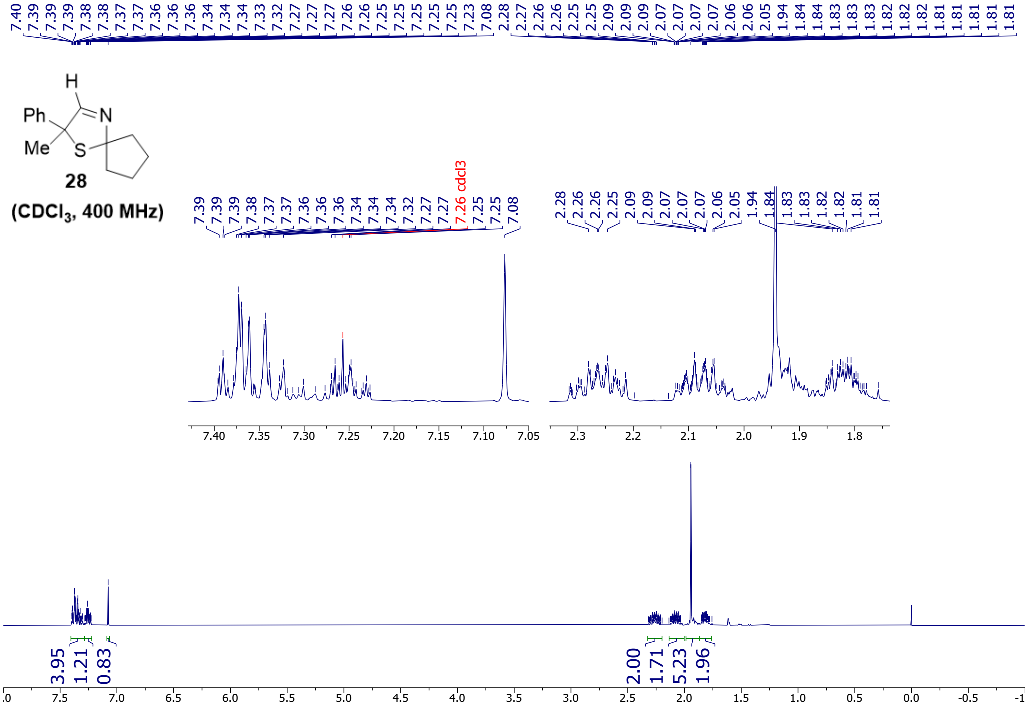

Supplementary Figure 106. ^{13}C NMR of Compound **28** at 25 °C

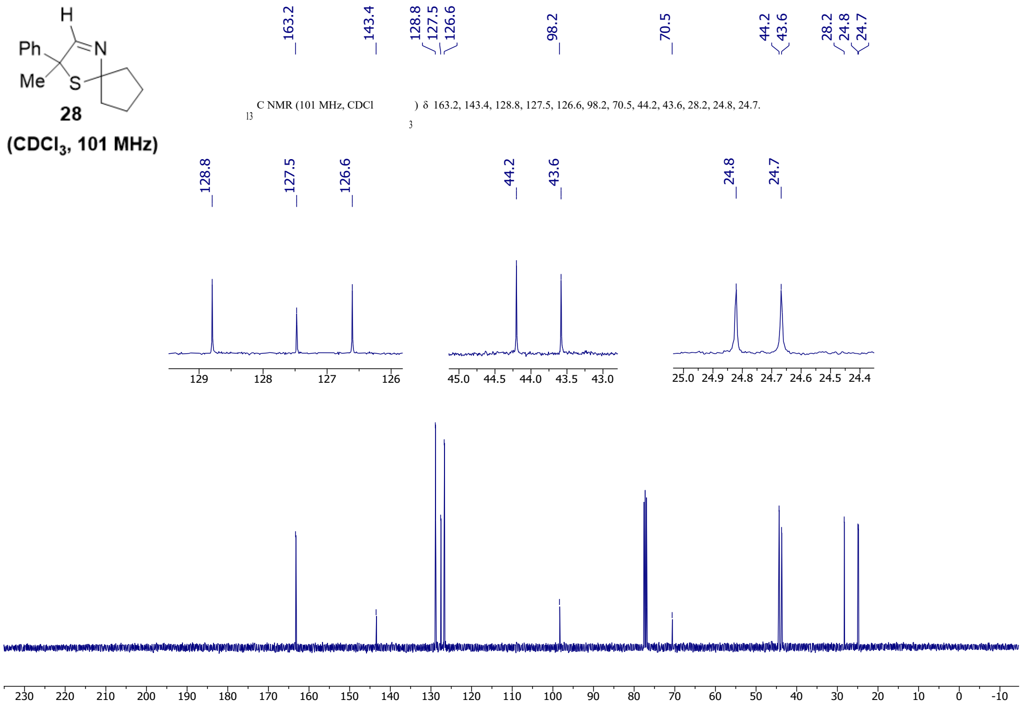

Supplementary Figure 107. ^{1}H NMR of Compound **30** at 25 °C

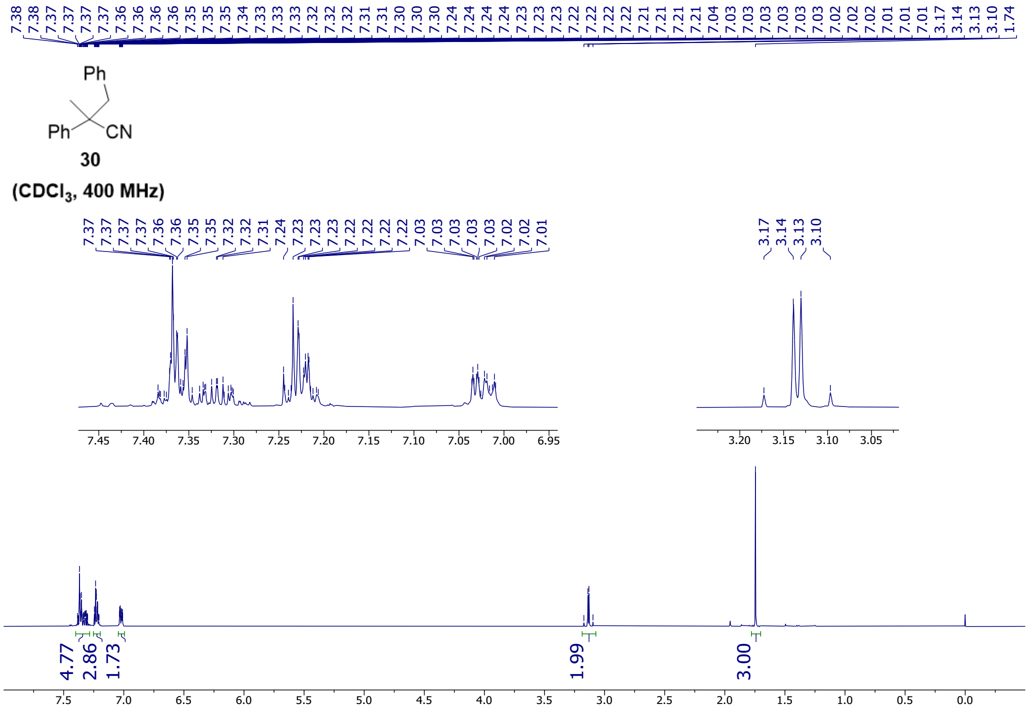

Supplementary Figure 108. ^{13}C NMR of Compound **30** at 25 °C

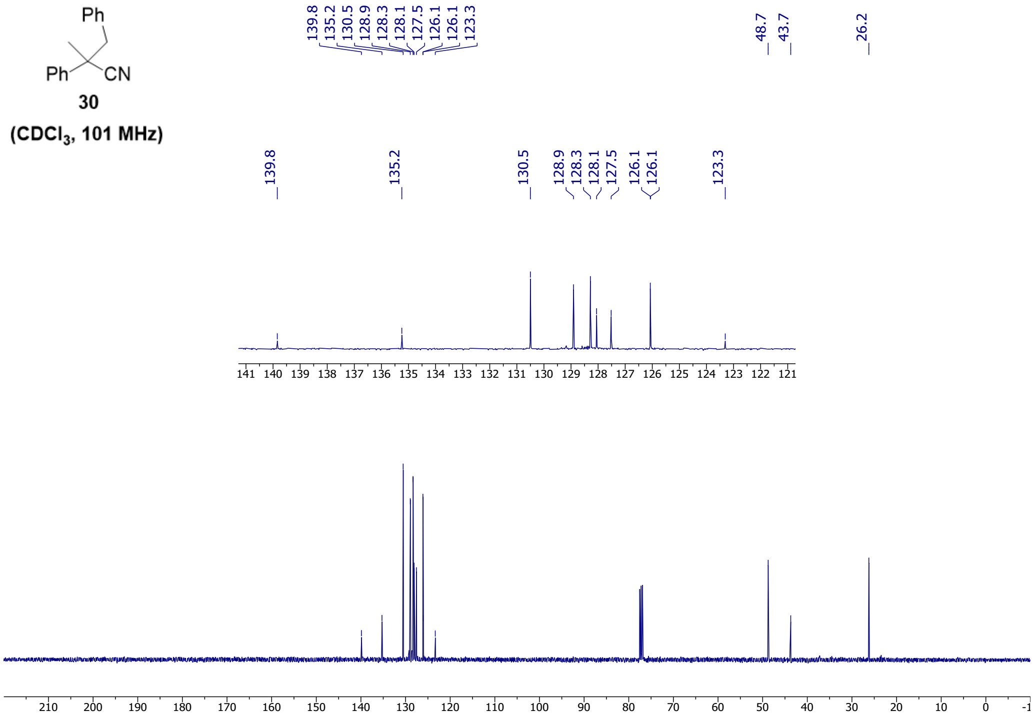

Supplementary Figure 109. ^{1}H NMR of Compound **31** at 25 °C

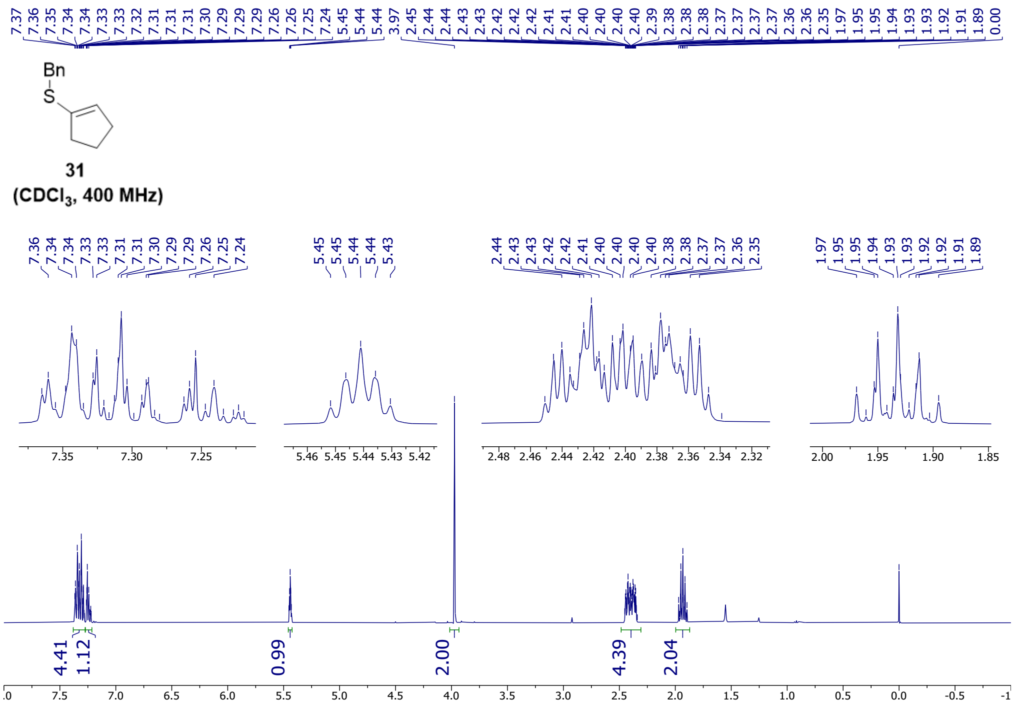

Supplementary Figure 110. ^{13}C NMR of Compound **31** at 25 °C

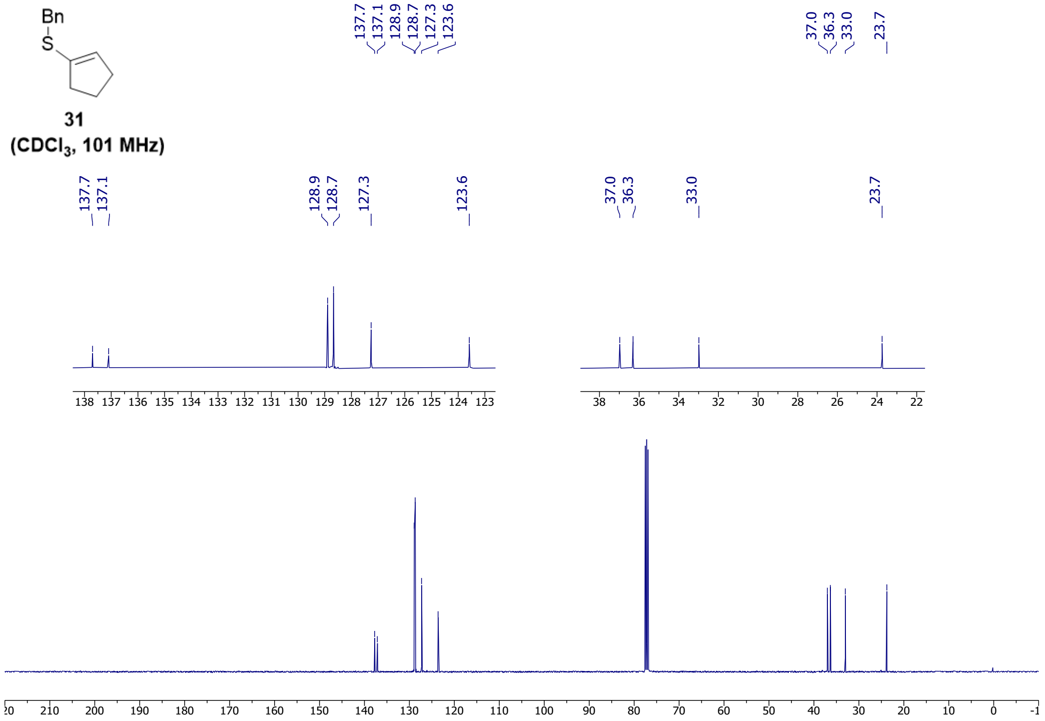

DFT Calculations Data

Computational Details

All geometry optimizations of intermediates and transition states were performed using unrestricted UB3LYP[11]-D3[12]/def2-SVP[13] method, in THF solvent using the SMD solvent model[14] with "opt=noeigen" and "guess=mix" keywords as implemented in Gaussian 16.[15] Frequency calculations were also conducted at the same level of theory to obtain vibrational frequencies to determine the identity of stationary points as intermediates (no imaginary frequencies) or transition states (only one imaginary frequency), as well as obtaining the thermal corrections to enthalpy ($H_{correction}$) and free energy ($G_{correction}$) at the temperature of 298 K. Energies were refined by computing single point energies with UB3LYP-D3/def2-TZVPP[13] and M062X[16]/def2-TZVPP method in THF solvent using the SMD solvent model. For comparison, domain-based local pair-natural orbital coupled-cluster calculations using single and double excitations with perturbative triple excitations (DLPNO-CCSD(T))[17] were performed with def2-TZVPP as basis set and def2-TZVPP/C as auxiliary basis set along with "TightSCF" and "NormalPNO" keywords using ORCA software.[18] Single electron transfer barriers were calculated using Nelsen's[19] four point scheme on the basis of Marcus-Hush theory.[20] All structural figures were generated with CYLview.[21] Distances in structural figures are shown in Å and energies are in kcal/mol.

Supplementary Discussion

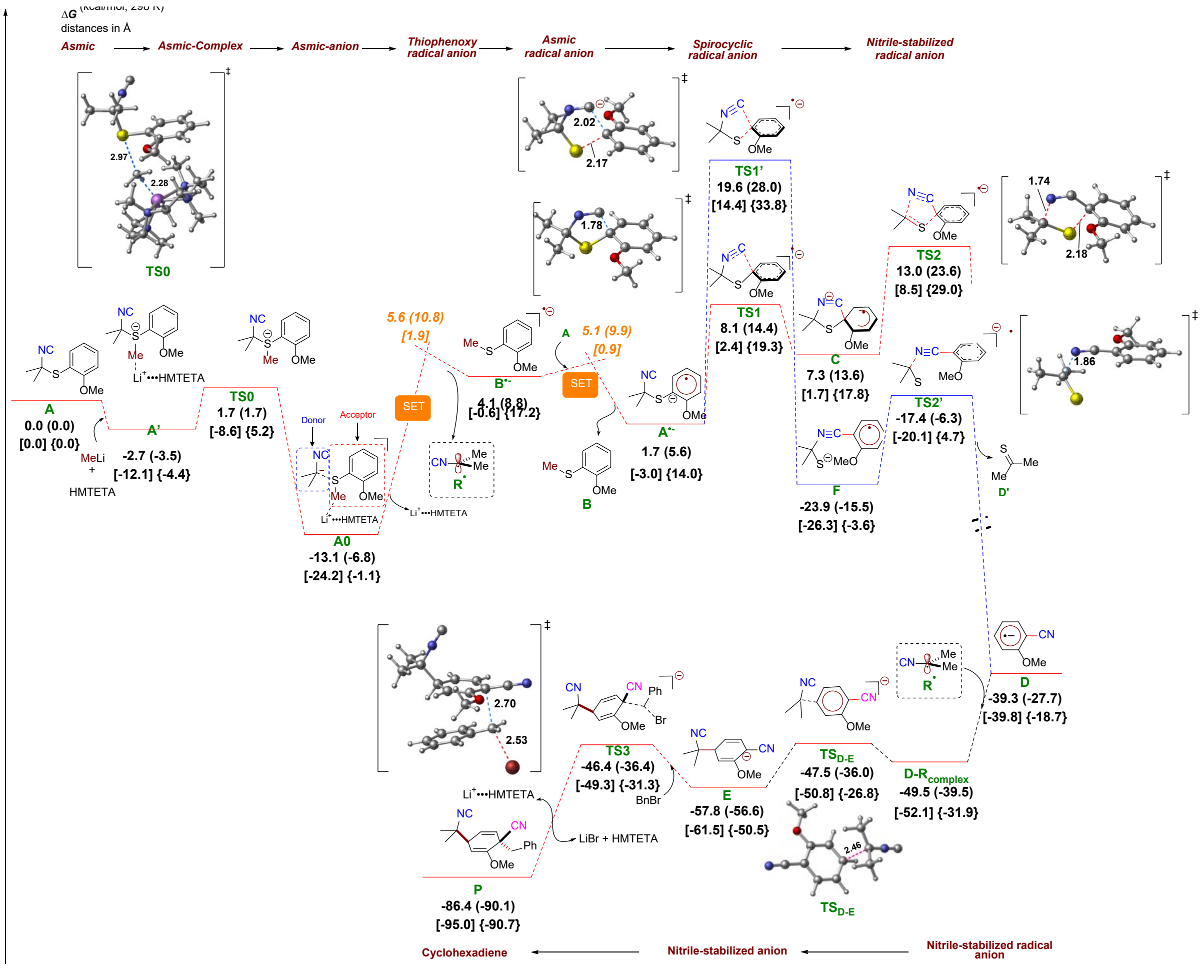

Supplementary Figure 111. Energetics with Different Methods and Conformational Search. Calculated energetics of dearomatization-dimerization-dislocation sequence. Free energies (kcal/mol) were computed at the UB3LYP-D3/def2-TZVPP-SMD(THF)//UB3LYP-D3/def2-SVP-SMD(THF), M062X/def2-TZVPP-SMD(THF)//UB3LYP-D3/def2-SVP-SMD(THF) (in parenthesis), UB3LYP-D3/def2-SVP-SMD(THF)(in bracket) and DLPNO-CCSD(T)/def2-TZVPP-SMD(THF)//UB3LYP-D3/def2-SVP-SMD(THF) (in brace) levels of theory.

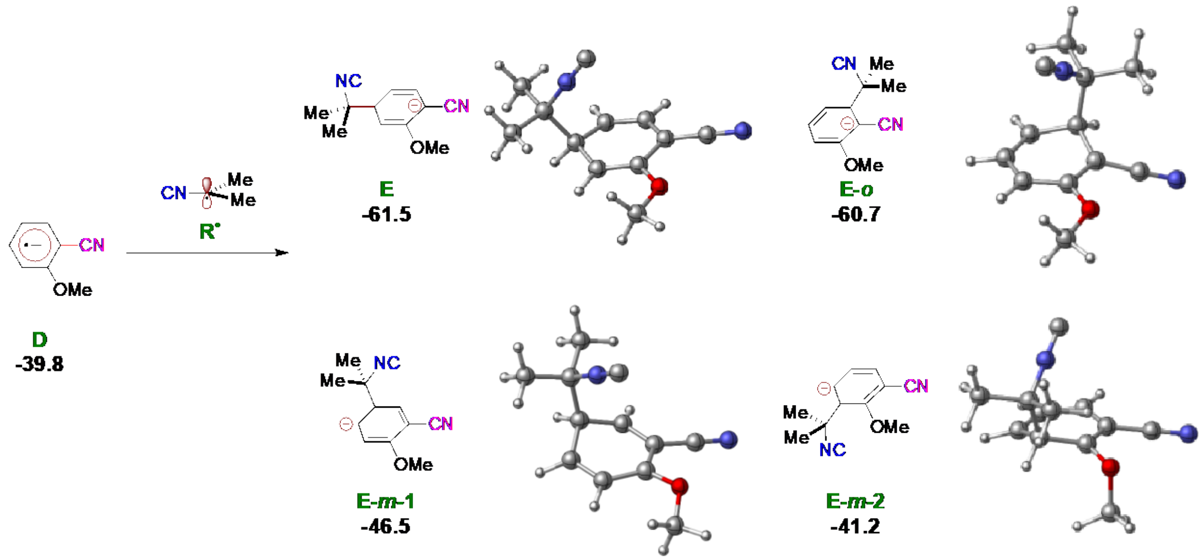

Supplementary Figure S112. Conformational search of key intermediates of radical anion-radical coupling. Relative free energies (kcal/mol) were calculated with respect to corresponding lowest-energy level structure at the UB3LYP-D3/def2-SVP-SMD(THF) level of theory.

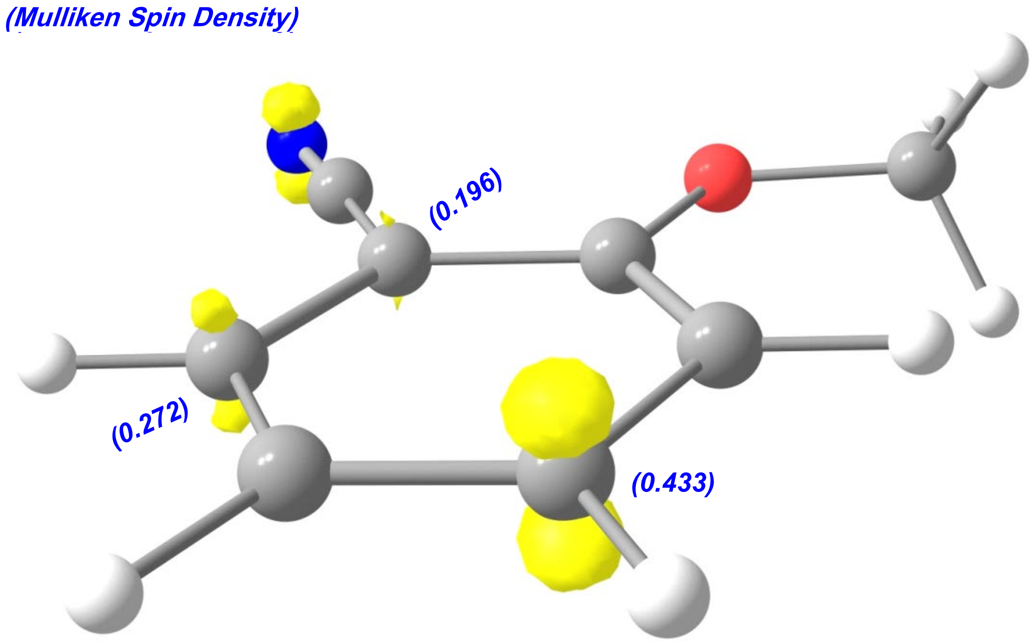

Supplementary Figure S113. Spin density plot for nitrile stabilized radical anion D with corresponding Mulliken spin density values at the UB3LYP-D3/def2-SVP-SMD(THF) level of theory.

As shown in Supplementary Figure S113, maximum spin density at the carbon para to nitrile group explains the preferred regioselectivity of the reaction.

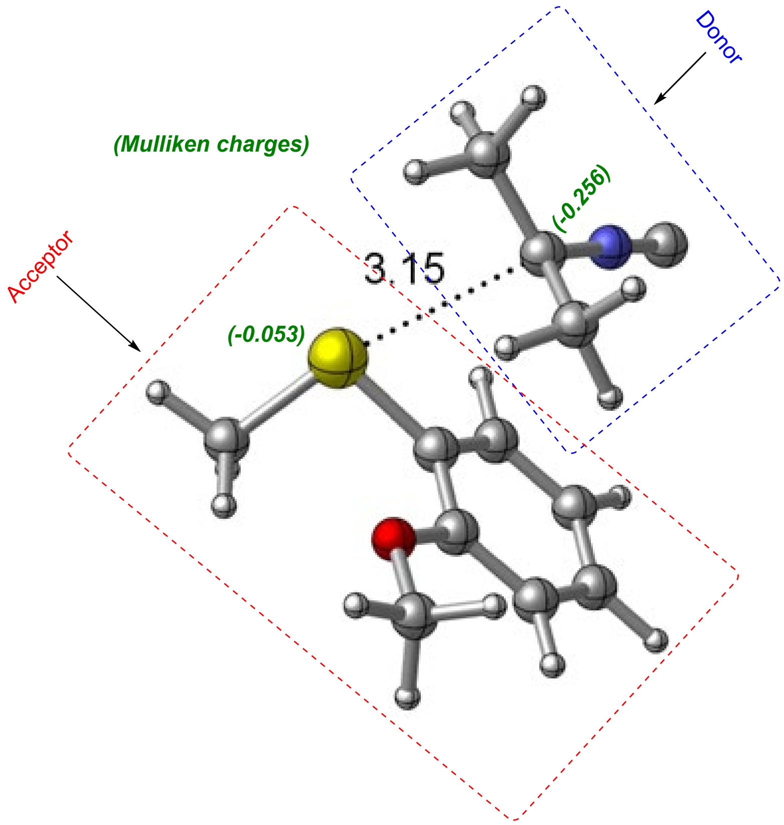

Supplementary Figure S114. Mulliken charge distribution on the S and C atom of A^- species calculated at the UB3LYP-D3/def2-SVP-SMD(THF) level of theory.

A higher concentration of negative charge on the carbon atom attached to the isonitrile group explains the donor and acceptor character which leads to a single electron transfer process from C to S resulting in radical anionic species **B**$^{\cdot -}$ and **R**$^{\cdot}$ radical as shown in Figure **S1**.

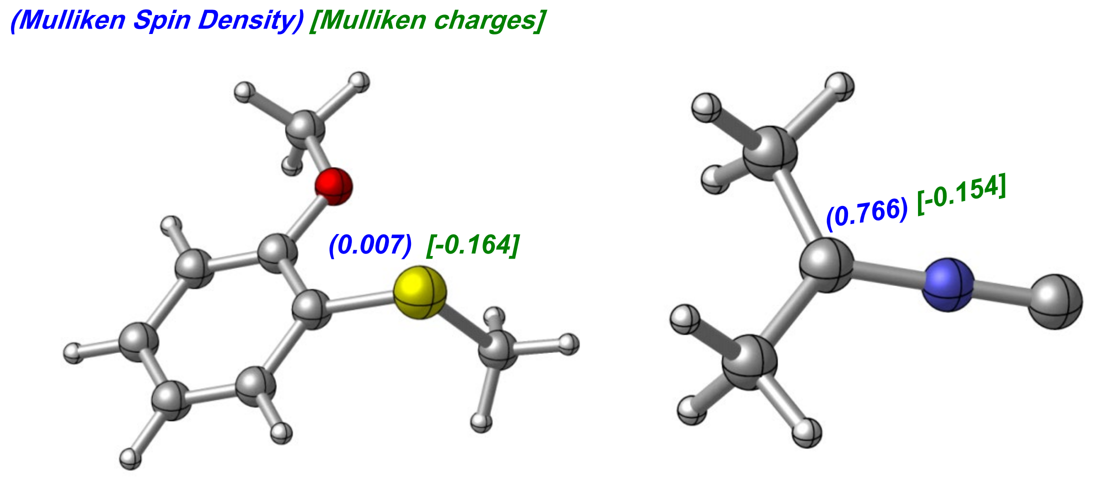

Supplementary Figure S115. Mulliken charge and spin density on the S and C atom of B$^{\cdot -}$ species and radical R$^{\cdot}$ species respectively after SET calculated at the UB3LYP-D3/def2-SVP-SMD(THF) level of theory.

Comparison of Figure **S3** (showing Mulliken charge on the C atom and S atom of A^{-} species before SET) and Figure **S4** (showing Mulliken charge and spin density on the C atom and S atom after SET) reveals existence of the SET process.

Supplementary References

[1] Bonnert, R. V.; Patel, A.; Thom, S. *Novel Compounds*. U. S. Patent WO 2005018529 A2, March 3, 2005.

[2] Lujan-Montelongo, J. A.; Estevez, A. O.; Fleming, F. F. *Eur. J. Org. Chem.* **2015**, *2015*, 1602-1605.

[3] Lujan-Montelongo, J. A.; Fleming, F. F. *Composition, Synthesis, and Use of New Arylsulfonyl Isonitriles*. U. S. Patent WO 2015127175 Al, August 27, 2015.

[4] Chao, A.; Alwedi, E.; Fleming, F. F. *Synthesis*, **2019**, *51*, 2122-2127.

[5] Alwedi, E.; Altundas, B.; Chao, A.; Ziminsky, Z. L.; Natrayan, M.; Fleming, F. F. *Org. Synth.* **2021**, *98*, 147-170.

[6] Alwedi, E.; Lujan-Montelongo, J. A.; Pitta, B. R.; Chao, A.; Cortés-Mejía, R.; del Campo, J. M.; Fleming, F. F. *Org. Lett.* **2018**, *20*, 5910–5913.

[7] Alwedi, E.; Lujan-Montelongo, J. A.; Cortés-Mejía, R.; del Campo, J. M.; Altundas, B.; Fleming, F. F. *Eur. J. Org. Chem*. **2019**, *2019*, 4644-4648.

[8] Stalling, T.; Brockmeyer, F.; Kröger, D.; Schwäblein, A.; Martens, J. Z. Naturforsch. **2012**, *67b*, 1045-1055.

[9] Masschelein, K. G. R.; Stevens, C. V. *Tetrahedron* Lett. **2008**, *49*, 4336–4338.

[10] Recio, III, A.; Heinzman, J. D.; Tunge, J. A. *Chem. Commun*. **2012**, *48*, 142-144.

[11] (a) Lee, C.; Yang, W.; Parr, R. G., Development of the Colle-Salvetti Correlation-Energy Formula into a Functional of the Electron Density. Phys. Rev. B 1988, 37, 785–789. (b) Becke, A. D., Density-Functional Thermochemistry. III. The Role of Exact Exchange. *J. Chem. Phys.* **1993**, *98*, 5648–5652.

[12] (a) Grimme, S. Accurate description of van der Waals complexes by density functional theory including empirical corrections. *J. Comput. Chem.* **2004**, *25*, 1463-1473. (b) Grimme, S.; Antony, J.; Ehrlich, S.; Krieg, H. A consistent and accurate ab initio parametrization of density functional dispersion correction (DFT-D) for the 94 elements H-Pu. *J. Chem. Phys.* **2010**, *132*, 154104. (c) Grimme, S. Density functional theory with London dispersion corrections. *WIRES Comput. Mol. Sci.* **2011**, *1*, 211-228. (d) Ehrlich, S.; Moellmann, J.; Grimme, S. Dispersion-Corrected Density Functional Theory for Aromatic Interactions in Complex Systems. *Acc. Chem. Res.* **2012**, *46*, 916-926.

[13] (a) Weigend, F.; Ahlrichs, R. Balanced basis sets of split valence, triple zeta valence and quadruple zeta valence quality for H to Rn: Design and assessment of accuracy. *Phys. Chem. Chem. Phys.* **2005**, *7*, 3297-3305. (b) Weigend, F. Accurate Coulomb-fitting basis sets for H to Rn. *Phys. Chem. Chem. Phys.* **2006**, *8*, 1057-1065.

[14] (a) Klamt, A.; Schüürmann, G. COSMO: a new approach to dielectric screening in solvents with explicit expressions for the screening energy and its gradient. *J. Chem. Soc. Perkin Trans.* 2 **1993**, *0*, 799-805. (b) Tomasi, J.; Persico, M. Molecular Interactions in Solution: An Overview of Methods Based on Continuous Distributions of the Solvent. *Chem. Rev.* **1994**, *94*, 2027-2094. (c) Andzelm, J.; Kölmel, C.; Klamt, A. Incorporation of solvent effects into density functional calculations of molecular energies and geometries. *J. Chem. Phys.* **1995**, *103*, 9312-9320. (d) Barone, V.; Cossi, M. Quantum Calculation of Molecular Energies and Energy Gradients in Solution by a Conductor Solvent Model. *J. Phys. Chem. A* **1998**, *102*, 1995-2001. (e) Cossi, M.; Rega, N.; Scalmani, G.; Barone, V. Energies, structures, and electronic properties

of molecules in solution with the C-PCM solvation model. *J. Comput. Chem.* **2003**, *24*, 669-681.

[15] Gaussian 16, Revision C.01, Frisch, M. J.; Trucks, G. W.; Schlegel, H. B.; Scuseria, G. E.; Robb, M. A.; Cheeseman, J. R.; Scalmani, G.; Barone, V.; Petersson, G. A.; Nakatsuji, H.; Li, X.; Caricato, M.; Marenich, A. V.; Bloino, J.; Janesko, B. G.; Gomperts, R.; Mennucci, B.; Hratchian, H. P.; Ortiz, J. V.; Izmaylov, A. F.; Sonnenberg, J. L.; Williams-Young, D.; Ding, F.; Lipparini, F.; Egidi, F.; Goings, J.; Peng, B.; Petrone, A.; Henderson, T.; Ranasinghe, D.; Zakrzewski, V. G.; Gao, J.; Rega, N.; Zheng, G.; Liang, W.; Hada, M.; Ehara, M.; Toyota, K.; Fukuda, R.; Hasegawa, J.; Ishida, M.; Nakajima, T.; Honda, Y.; Kitao, O.; Nakai, H.; Vreven, T.; Throssell, K.; Montgomery, J. A., Jr.; Peralta, J. E.; Ogliaro, F.; Bearpark, M. J.; Heyd, J. J.; Brothers, E. N.; Kudin, K. N.; Staroverov, V. N.; Keith, T. A.; Kobayashi, R.; Normand, J.; Raghavachari, K.; Rendell, A. P.; Burant, J. C.; Iyengar, S. S.; Tomasi, J.; Cossi, M.; Millam, J. M.; Klene, M.; Adamo, C.; Cammi, R.; Ochterski, J. W.; Martin, R. L.; Morokuma, K.; Farkas, O.; Foresman, J. B.; Fox, D. J. Gaussian, Inc., Wallingford CT, 2016.

[16] Zhao, Y.; Truhlar, D. G. The M06 Suite of Density Functionals for Main Group Thermochemistry, Thermochemical Kinetics, Noncovalent Interactions, Excited States, and Transition Elements: Two New Functionals and Systematic Testing of Four M06-Class Functionals and 12 Other Functionals. *Theor. Chem. Acc.* **2008**, *120*, 215–241.

[17] Riplinger, C.; Sandhoefer, B.; Hansen, A.; Neese, F. Natural Triple Excitations in Local Coupled Cluster Calculations with Pair Natural Orbitals. *J. Chem. Phys.* **2013**, *139*, 134101.

[18] Neese, F. The ORCA program system. *Wiley Interdiscip. Rev. Comput. Mol. Sci.* **2012**, *2*, 73–78.

[19] (a) Nelsen, S. F.; Blackstock, S. C.; Kim, Y. Estimation of Inner Shell Marcus Terms for Amino Nitrogen Compounds by Molecular Orbital Calculations J. Am. Chem. Soc. 1987, 109, 677–682. (b) López-Estrada, O.; Laguna, H. G.; Barrueta-Flores, C.; Amador-Bedolla, C. Reassessment of the Four-Point Approach to the Electron-Transfer Marcus-Hush Theory. *ACS Omega* **2018**, *3* , 2130–2140.

[20] (a) Marcus, R. A. On the Theory of Oxidation-Reduction Reactions Involving Electron Transfer. III. Applications to Data on the Rates of Organic Redox Reactions *J. Chem. Phys*. **1957**, *26*, 872– 877. (b) Marcus, R. A. On the Theory of Oxidation-Reduction Reactions Involving Electron Transfer. II. Applications to Data on the Rates of Isotopic Exchange Reactions *J. Chem. Phys*. **1957**, *26*, 867– 871. (c) Marcus, R. A. On the Theory of Oxidation–Reduction Reactions Involving Electron Transfer. V. Comparison and Properties of Electrochemical and Chemical Rate Constants *J. Phys. Chem*. **1963**, *67*, 853– 857. (d) Marcus, R. A. On the Theory of Electron-Transfer Reactions. VI. Unified Treatment for Homogeneous and Electrode Reactions *J. Chem. Phys*. **1965**, *43*, 679. (e) Marcus, R. A. Chemical and Electrochemical Electron-Transfer Theory *Annu. Rev. Phys. Chem*. **1964**, *15*, 155– 196. (f) Hush, N. S. Adiabatic Rate Processes at Electrodes. I. Energy-Charge Relationships *J. Chem. Phys*. **1958**, *28*, 962– 972. (g) Hush, N. S. Electron transfer in retrospect and prospect 1: Adiabatic electrode processes *J. Electroanal. Chem*. **1999**, *470*, 170– 195.

[21] CYLview, 1.0b; Legault, C. Y., Université de Sherbrooke, 2009 (http://www.cylview.org)
