# Supplementary material for: Dearomatization of aromatic asmic isocyanides to complex cyclohexadienes
Source: Nat Commun. 2022 Oct 28;13:6444. doi: 10.1038/s41467-022-33807-7 (PMC9616822; doi:10.1038/s41467-022-33807-7)
Supplement: Supplementary file 3 — Description of Additional Supplementary Files [file 41467_2022_33807_MOESM3_ESM.docx]

**Supplementary Data 1 Description**

File Name: Supplementary Data 1
Description: Contains coordinates and energies of all species identified in Fig. 6.
