# Supplementary material for: Dearomatization of aromatic asmic isocyanides to complex cyclohexadienes
Source: Nat Commun. 2022 Oct 28;13:6444. doi: 10.1038/s41467-022-33807-7 (PMC9616822; doi:10.1038/s41467-022-33807-7)
Supplement: Supplementary file 4 — Supplementary Data 1 [file 41467_2022_33807_MOESM4_ESM.pdf]

Supplementary Data 1

Coordinates and Energies

A

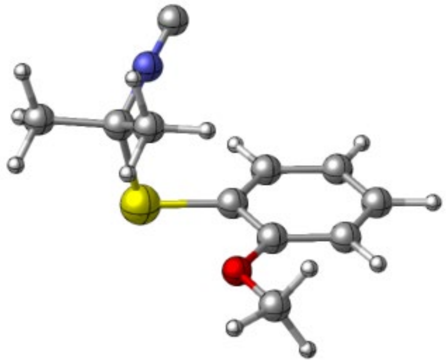

Zero-point correction= 0.216123 (Hartree/Particle)

Thermal correction to Energy= 0.230584

Thermal correction to Enthalpy= 0.231528

Thermal correction to Gibbs Free Energy= 0.174550

Sum of electronic and zero-point Energies= -954.417260

Sum of electronic and thermal Energies= -954.402799

Sum of electronic and thermal Enthalpies= -954.401855

Sum of electronic and thermal Free Energies= -954.458833

C	-3.16506500	1.65292600	1.76465600
C	-1.80640200	1.97649700	1.60435200
C	-1.41156700	3.30962100	1.45376500
C	-2.35224200	4.34150300	1.45852600
C	-3.70491200	4.02651700	1.61619800

C	-4.12574600	2.69902200	1.77560400
H	-1.05012500	1.19170800	1.59544800
H	-0.34910200	3.53659600	1.33014400
H	-2.04068000	5.38163600	1.33959300
H	-4.45815100	4.81738700	1.61929000
O	-3.62094400	0.39382300	1.92057700
C	-2.70318800	-0.68627100	1.96834500
H	-1.99378600	-0.58275100	2.80832700
H	-3.30385500	-1.59368500	2.11872000
H	-2.13637400	-0.78515400	1.02600300
C	-6.11471200	2.40495300	3.79714300
C	-7.61379900	2.18202300	4.02925800
H	-8.21305400	2.94681700	3.51336300
H	-7.90322900	1.18690200	3.66120900
H	-7.82830400	2.23417900	5.10786000
C	-5.25677800	1.36988100	4.52808300
H	-4.18711600	1.55300500	4.35823400
H	-5.45469000	1.43075000	5.61057600
H	-5.50681200	0.36319700	4.16875400
N	-5.75273500	3.71192700	4.25456900
C	-5.41803200	4.77494900	4.61522400
S	-5.86894200	2.34757400	1.93431100

UB3LYP-D3/def2-TZVPP-SMD(THF)//UB3LYP/def2-SVP-SMD(THF)

HF=-955.392678

M062X/def2-TZVPP-SMD(THF)// UB3LYP/def2-SVP-SMD(THF)

HF=-955.0975385

DLPNO-CCSD(T)/def2-TZVPP-SMD(THF)// UB3LYP/def2-SVP-SMD(THF)

HF= -953.69107

B$^{\cdot -}$

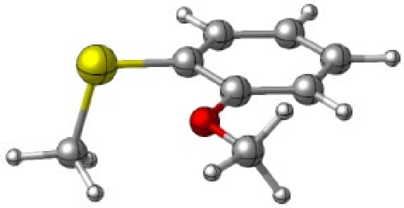

Zero-point correction= 0.155248 (Hartree/Particle)

Thermal correction to Energy= 0.166271

Thermal correction to Enthalpy= 0.167215

Thermal correction to Gibbs Free Energy= 0.117332

Sum of electronic and zero-point Energies= -783.778879

Sum of electronic and thermal Energies= -783.767856

Sum of electronic and thermal Enthalpies= -783.766912

Sum of electronic and thermal Free Energies= -783.816795

C	-3.20651000	1.86450200	1.86459600
C	-1.86316500	1.74425000	1.54245600
C	-0.97291000	2.88311100	1.63902400

C	-1.51666700	4.12757700	2.03568400
C	-2.86005500	4.25761000	2.35767400
C	-3.76610100	3.11702500	2.32598600
H	-1.46269600	0.78710200	1.20262200
H	0.08421900	2.77754900	1.38078300
H	-0.86629100	5.00985300	2.09231400
H	-3.26554300	5.22738200	2.66437400
O	-4.09983100	0.82768600	1.76022000
C	-3.64078100	-0.43206900	1.34759200
H	-2.87652500	-0.84792600	2.03319200
H	-4.51151600	-1.10596500	1.34490900
H	-3.20945000	-0.41197700	0.32731200
S	-5.46335700	3.29638700	2.71387100
C	-6.30304500	3.58603500	1.06425600
H	-7.38069900	3.74570400	1.24646300
H	-5.88610800	4.47438700	0.56212800
H	-6.17362900	2.70454700	0.41605800

UB3LYP-D3/def2-TZVPP-SMD(THF)//UB3LYP/def2-SVP-SMD(THF)

HF=-784.513203

M062X/def2-TZVPP-SMD(THF)// UB3LYP/def2-SVP-SMD(THF)

HF=-784.2907122

DLPNO-CCSD(T)/def2-TZVPP-SMD(THF)// UB3LYP/def2-SVP-SMD(THF)

HF=-783.168151997909

A$^{\cdot-}$

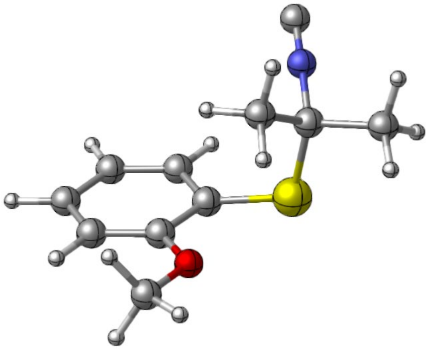

Zero-point correction= 0.211120 (Hartree/Particle)

Thermal correction to Energy= 0.225348

Thermal correction to Enthalpy= 0.226292

Thermal correction to Gibbs Free Energy= 0.169103

Sum of electronic and zero-point Energies= -954.455176

Sum of electronic and thermal Energies= -954.440948

Sum of electronic and thermal Enthalpies= -954.440004

Sum of electronic and thermal Free Energies= -954.497194

C	-3.39816700	1.98575900	-1.60249800
C	-2.01738000	1.88740400	-1.64379200
C	-1.19150700	3.06729000	-1.73790500
C	-1.82576100	4.33024400	-1.80353900
C	-3.20718100	4.44325700	-1.76887000
C	-4.06250200	3.27454500	-1.64521700
H	-1.53640700	0.90854700	-1.59548200

H	-0.10335400	2.97277500	-1.78014500
H	-1.21784500	5.23945600	-1.88666600
H	-3.68442900	5.42655600	-1.82386300
O	-4.23063300	0.89942900	-1.52048200
C	-3.67541400	-0.39048300	-1.53091100
H	-3.09481200	-0.58957400	-2.45261400
H	-4.51521500	-1.10051900	-1.48435800
H	-3.01369300	-0.57159900	-0.66180200
C	-6.32136300	3.33731300	-3.39135400
C	-7.85449700	3.20427000	-3.38917700
H	-8.31841200	4.03466100	-2.83325800
H	-8.14076700	2.25668000	-2.90930400
H	-8.24400400	3.21351200	-4.41944700
C	-5.66540900	2.17847000	-4.15504700
H	-4.57372600	2.30700200	-4.17795400
H	-6.03962400	2.13792300	-5.19146700
H	-5.88702100	1.23252600	-3.64259600
N	-5.99050000	4.55930700	-4.08412100
C	-5.69094000	5.57638600	-4.58549000
S	-5.79704700	3.42116300	-1.68338700

UB3LYP-D3/def2-TZVPP-SMD(THF)//UB3LYP/def2-SVP-SMD(THF)

HF=-955.433447

M062X/def2-TZVPP-SMD(THF)// UB3LYP/def2-SVP-SMD(THF)

HF=-955.1389725

DLPNO-CCSD(T)/def2-TZVPP-SMD(THF)// UB3LYP/def2-SVP-SMD(THF)

HF=-953.726727496664

TS1

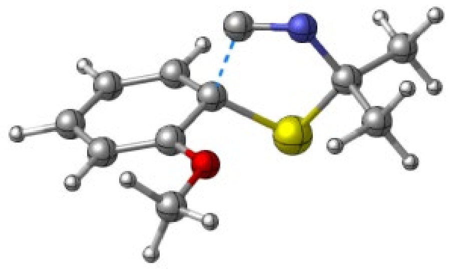

Zero-point correction= 0.212036 (Hartree/Particle)

Thermal correction to Energy= 0.225471

Thermal correction to Enthalpy= 0.226415

Thermal correction to Gibbs Free Energy= 0.172321

Sum of electronic and zero-point Energies= -954.448833

Sum of electronic and thermal Energies= -954.435399

Sum of electronic and thermal Enthalpies= -954.434455

Sum of electronic and thermal Free Energies= -954.488548

C	-6.96542900	0.75950500	-1.24037800
C	-7.06630900	-0.15866900	-0.21330600
C	-5.90441500	-0.71611800	0.38825400
C	-4.63250300	-0.30799400	-0.06735000
C	-4.50713600	0.60981700	-1.09024400

C	-5.66020400	1.17471600	-1.81961100
H	-8.04713400	-0.46921900	0.15214400
H	-6.00584300	-1.43310600	1.20680800
H	-3.73158100	-0.71365600	0.40567500
H	-3.51659600	0.92675800	-1.42922100
O	-8.03378200	1.30652900	-1.87873100
C	-9.33816300	0.97864700	-1.45904700
H	-9.55266500	-0.10011200	-1.57548300
H	-10.02915200	1.54673600	-2.09877200
H	-9.51762300	1.26059700	-0.40450700
C	-5.70192200	3.00303900	-3.88358300
C	-4.54885800	3.78295700	-4.52447600
H	-3.58355200	3.31572700	-4.27701700
H	-4.53237300	4.82732200	-4.17302900
H	-4.66182100	3.79123100	-5.62238000
C	-7.05836100	3.61780500	-4.25143300
H	-7.21283000	3.57023200	-5.34366900
H	-7.10989400	4.67345200	-3.93979000
H	-7.86375400	3.06116500	-3.75185200
N	-5.66307400	1.62266300	-4.28183100
C	-5.65324500	0.62708400	-3.50999800
S	-5.48032500	3.01725200	-1.98332800

UB3LYP-D3/def2-TZVPP-SMD(THF)//UB3LYP/def2-SVP-SMD(THF)

HF=-955.426459

M062X/def2-TZVPP-SMD(THF)// UB3LYP/def2-SVP-SMD(THF)

HF=-955.1281795

DLPNO-CCSD(T)/def2-TZVPP-SMD(THF)// UB3LYP/def2-SVP-SMD(THF)

HF=-953.721540615679

TS1'

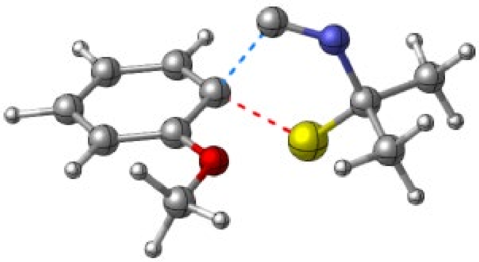

Zero-point correction= 0.211475 (Hartree/Particle)

Thermal correction to Energy= 0.223929

Thermal correction to Enthalpy= 0.224873

Thermal correction to Gibbs Free Energy= 0.172567

Sum of electronic and zero-point Energies= -954.430556

Sum of electronic and thermal Energies= -954.418102

Sum of electronic and thermal Enthalpies= -954.417158

Sum of electronic and thermal Free Energies= -954.469465

C	-6.78535200	0.79875900	-1.35077800
C	-7.00641800	0.05342500	-0.19186500
C	-5.92108200	-0.45570900	0.55204500
C	-4.61398100	-0.20035200	0.11709600

C	-4.39318100	0.55096400	-1.03847800
C	-5.45868600	1.03878500	-1.84782600
H	-8.02336100	-0.14887500	0.15066900
H	-6.10878500	-1.03352900	1.46116800
H	-3.76043700	-0.58236100	0.68728300
H	-3.36807800	0.75984100	-1.36164400
O	-7.79278100	1.29324700	-2.13037700
C	-9.13133700	1.09411000	-1.74352200
H	-9.40158300	0.02176800	-1.70593500
H	-9.75740500	1.58598000	-2.50264200
H	-9.35390700	1.54543300	-0.75785500
C	-5.37920100	2.87758600	-4.12369300
C	-4.29577300	3.59054600	-4.96326600
H	-4.49236700	3.45160200	-6.03991700
H	-3.30181800	3.17761400	-4.73115800
H	-4.28170100	4.67121000	-4.74751200
C	-6.77021400	3.40618600	-4.54521000
H	-6.94931200	3.22776700	-5.61965200
H	-6.84130700	4.48898200	-4.35235900
H	-7.54504300	2.89156200	-3.96042300
N	-5.34934800	1.44577200	-4.50240800
C	-5.38548000	0.45163200	-3.78167400
S	-5.07450400	3.08380900	-2.44791200

UB3LYP-D3/def2-TZVPP-SMD(THF)//UB3LYP/def2-SVP-SMD(THF)

HF=-955.4084269

M062X/def2-TZVPP-SMD(THF)// UB3LYP/def2-SVP-SMD(THF)

HF=-955.1067887

DLPNO-CCSD(T)/def2-TZVPP-SMD(THF)// UB3LYP/def2-SVP-SMD(THF)

HF=-953.698651122582

C

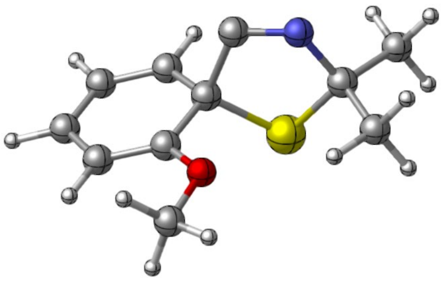

Zero-point correction= 0.212350 (Hartree/Particle)

Thermal correction to Energy= 0.226457

Thermal correction to Enthalpy= 0.227401

Thermal correction to Gibbs Free Energy= 0.171318

Sum of electronic and zero-point Energies= -954.448550

Sum of electronic and thermal Energies= -954.434443

Sum of electronic and thermal Enthalpies= -954.433499

Sum of electronic and thermal Free Energies= -954.489582

C -6.90836800 0.85952300 -1.25895700

C -7.01870300 -0.03445800 -0.21226200

C	-5.86296700	-0.60754000	0.38440700
C	-4.58481800	-0.24954600	-0.10051100
C	-4.44736700	0.64258800	-1.14125100
C	-5.59625600	1.25515100	-1.85294300
H	-8.00170800	-0.31397800	0.17167200
H	-5.97131500	-1.30659600	1.21743200
H	-3.69056900	-0.67887800	0.36382400
H	-3.45380100	0.91683200	-1.50741600
O	-7.96717600	1.41753000	-1.89769600
C	-9.27644000	1.13518200	-1.45754100
H	-9.52059300	0.06036900	-1.54646700
H	-9.95763200	1.70697700	-2.10418500
H	-9.43590200	1.44730200	-0.40854100
C	-5.57891700	3.20497900	-3.77184600
C	-4.39957000	3.98548700	-4.36337700
H	-3.44898800	3.48422100	-4.12529200
H	-4.36179400	5.01338700	-3.96747800
H	-4.49804200	4.04254800	-5.46132700
C	-6.91540300	3.87335300	-4.12097800
H	-7.06090900	3.87922300	-5.21553200
H	-6.94394200	4.91493200	-3.76188200
H	-7.73986400	3.31505900	-3.65524400
N	-5.57256900	1.84309200	-4.24255400

C -5.59619600 0.82108700 -3.49066900

S -5.38009600 3.11967200 -1.87379700

UB3LYP-D3/def2-TZVPP-SMD(THF)//UB3LYP/def2-SVP-SMD(THF)

HF=-955.426721

M062X/def2-TZVPP-SMD(THF)// UB3LYP/def2-SVP-SMD(THF)

HF=-955.1284854

DLPNO-CCSD(T)/def2-TZVPP-SMD(THF)// UB3LYP/def2-SVP-SMD(THF)

HF=-953.722903245872

F

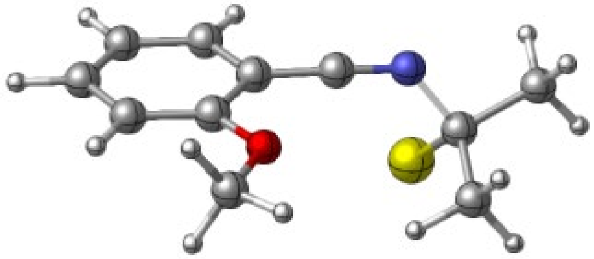

Zero-point correction= 0.213686 (Hartree/Particle)

Thermal correction to Energy= 0.228180

Thermal correction to Enthalpy= 0.229124

Thermal correction to Gibbs Free Energy= 0.170624

Sum of electronic and zero-point Energies= -954.491175

Sum of electronic and thermal Energies= -954.476681

Sum of electronic and thermal Enthalpies= -954.475737

Sum of electronic and thermal Free Energies= -954.534237

C -6.97262600 0.41380000 -1.30641700

C	-7.15495900	-0.19790600	-0.07522700
C	-6.04273800	-0.53988100	0.73275100
C	-4.74138300	-0.24805500	0.29076700
C	-4.52839500	0.36637000	-0.93608600
C	-5.63834700	0.71000100	-1.78513400
H	-8.16178400	-0.41612700	0.28355900
H	-6.20763700	-1.02190400	1.69931600
H	-3.88230900	-0.49608800	0.92111000
H	-3.52200500	0.62240500	-1.27322300
O	-7.96560400	0.79006000	-2.15340400
C	-9.31059000	0.57548100	-1.77658800
H	-9.53210900	-0.49821800	-1.63432000
H	-9.93212000	0.96045700	-2.59731200
H	-9.56865200	1.11765900	-0.84858100
C	-5.02206100	3.37991300	-4.25745900
C	-4.32834600	3.50273000	-5.62089600
H	-4.89882000	2.99508000	-6.42184500
H	-3.32294600	3.05694200	-5.57007800
H	-4.21298500	4.56493700	-5.88501200
C	-6.46077300	3.91848600	-4.34439200
H	-7.05374900	3.39411500	-5.11809500
H	-6.43510800	4.99173000	-4.58840800
H	-6.96577500	3.80183500	-3.37357400

N	-5.19617300	1.85700900	-4.04931000
C	-5.44744800	1.28112500	-3.01807400
S	-4.06300200	4.15179700	-2.92141100

UB3LYP-D3/def2-TZVPP-SMD(THF)//UB3LYP/def2-SVP-SMD(THF)

HF=-955.475659

M062X/def2-TZVPP-SMD(THF)// UB3LYP/def2-SVP-SMD(THF)

HF=-955.1741778

DLPNO-CCSD(T)/def2-TZVPP-SMD(THF)// UB3LYP/def2-SVP-SMD(THF)

HF=-953.756335974625

TS2

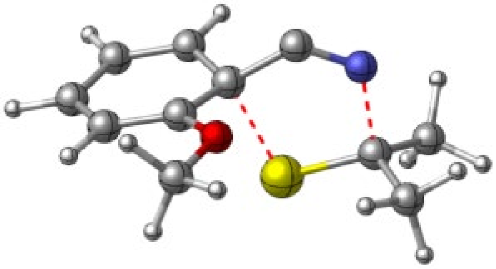

Zero-point correction= 0.210275 (Hartree/Particle)

Thermal correction to Energy= 0.223215

Thermal correction to Enthalpy= 0.224159

Thermal correction to Gibbs Free Energy= 0.170087

Sum of electronic and zero-point Energies= -954.438594

Sum of electronic and thermal Energies= -954.425654

Sum of electronic and thermal Enthalpies= -954.424709

Sum of electronic and thermal Free Energies= -954.478782

C	-6.98145100	0.61654000	-1.39500600
C	-7.01709900	0.15442900	-0.09037100
C	-5.81935700	-0.19922300	0.58175600
C	-4.58894200	-0.14091600	-0.10131800
C	-4.53459800	0.31392900	-1.40708300
C	-5.70000100	0.90336600	-2.06442800
H	-7.96863900	0.04344600	0.43213100
H	-5.86701700	-0.56187800	1.61193200
H	-3.67467600	-0.48106700	0.39539900
H	-3.58817100	0.34148600	-1.95294200
O	-8.07417800	0.93930600	-2.12980500
C	-9.35297400	0.84135300	-1.54291300
H	-9.58734400	-0.19242700	-1.22742100
H	-10.07758500	1.14951100	-2.31017600
H	-9.45493200	1.51003300	-0.66811600
C	-5.53926200	3.42650200	-3.62501300
C	-4.29669500	4.07314600	-4.24513300
H	-3.72623600	3.32769600	-4.82497100
H	-3.62923900	4.49228200	-3.47854400
H	-4.56708300	4.88763700	-4.94024700
C	-6.85547400	4.02035700	-4.10139200
H	-6.93453400	3.93868300	-5.20119200
H	-6.93126100	5.08875600	-3.83254700

H	-7.70288000	3.48445900	-3.64963700
N	-5.58580600	1.82156000	-4.29628300
C	-5.68688800	0.87789100	-3.51188800
S	-5.44758400	3.05863800	-1.86120100

UB3LYP-D3/def2-TZVPP-SMD(THF)//UB3LYP/def2-SVP-SMD(THF)

HF=-955.416464

M062X/def2-TZVPP-SMD(THF)// UB3LYP/def2-SVP-SMD(THF)

HF=-955.1113553

DLPNO-CCSD(T)/def2-TZVPP-SMD(THF)// UB3LYP/def2-SVP-SMD(THF)

HF=-953.703864892802

TS2’

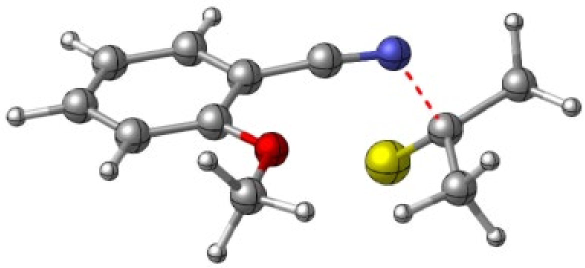

Zero-point correction= 0.211773 (Hartree/Particle)

Thermal correction to Energy= 0.224673

Thermal correction to Enthalpy= 0.225618

Thermal correction to Gibbs Free Energy= 0.171410

Sum of electronic and zero-point Energies= -954.484059

Sum of electronic and thermal Energies= -954.471158

Sum of electronic and thermal Enthalpies= -954.470214

Sum of electronic and thermal Free Energies= -954.524421

C	-6.91843900	0.52452200	-1.42345600
C	-7.12789600	-0.01118900	-0.15956700
C	-6.03329300	-0.40053200	0.64902200
C	-4.72278900	-0.23431000	0.17331600
C	-4.48857100	0.29986900	-1.08856300
C	-5.57785000	0.69090500	-1.93491500
H	-8.14241500	-0.12825500	0.22400800
H	-6.21811400	-0.82051600	1.64074400
H	-3.87305600	-0.52043400	0.80023000
H	-3.46956200	0.44427100	-1.45393800
O	-7.89936800	0.94206900	-2.26768200

C	-9.24899500	0.82490300	-1.86865600
H	-9.53359800	-0.22697100	-1.68123600
H	-9.85670500	1.21448100	-2.69767400
H	-9.46387600	1.41767200	-0.96067000
C	-4.97801200	3.57755600	-4.29534900
C	-4.30594000	3.75745300	-5.66634700
H	-3.99726400	2.78515800	-6.08320000
H	-3.41347700	4.39252300	-5.57914200
H	-4.98954200	4.22294800	-6.39908800
C	-6.46819100	3.90722100	-4.31460500
H	-6.99513700	3.39183000	-5.13804900
H	-6.59749100	4.99532500	-4.45265100
H	-6.94251300	3.62431600	-3.36319100
N	-5.14754900	1.72985900	-4.25485900
C	-5.37468800	1.16859800	-3.22060200
S	-4.08563400	4.15278200	-2.90755000

UB3LYP-D3/def2-TZVPP-SMD(THF)//UB3LYP/def2-SVP-SMD(THF)

HF=-955.466186

M062X/def2-TZVPP-SMD(THF)// UB3LYP/def2-SVP-SMD(THF)

HF=-955.1603115

DLPNO-CCSD(T)/def2-TZVPP-SMD(THF)// UB3LYP/def2-SVP-SMD(THF)

HF=-953.743923179003

D

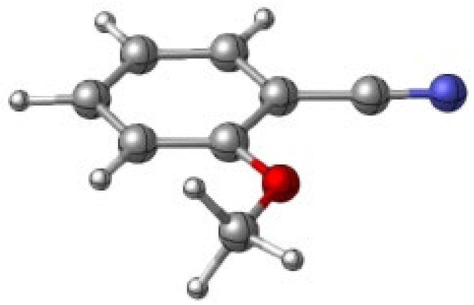

Zero-point correction= 0.127884 (Hartree/Particle)

Thermal correction to Energy= 0.136796

Thermal correction to Enthalpy= 0.137740

Thermal correction to Gibbs Free Energy= 0.093355

Sum of electronic and zero-point Energies= -438.646001

Sum of electronic and thermal Energies= -438.637089

Sum of electronic and thermal Enthalpies= -438.636145

Sum of electronic and thermal Free Energies= -438.680530

C	-2.22326200	-0.45852300	0.03170400
C	-0.84468800	-0.52863300	-0.02631700
C	-0.03164300	0.67288400	-0.10039800
C	-0.73995500	1.94522400	-0.10913900
C	-2.12461500	1.98990700	-0.04839200
C	-2.89752200	0.81142500	0.02147300
H	-2.81543000	-1.37372300	0.08595600
H	-0.15625700	2.86825400	-0.16381100
H	-2.62447300	2.96541500	-0.05725200

H	-3.98859500	0.85089600	0.06627700
O	-0.12495700	-1.69147000	-0.02013400
C	-0.80848000	-2.91886900	0.05018400
H	-1.48116000	-3.07224500	-0.81533100
H	-0.04442600	-3.71051700	0.04656800
H	-1.40833700	-3.01020900	0.97604500
C	1.36240400	0.60155300	-0.16336100
N	2.54372300	0.58237500	-0.21895900

UB3LYP-D3/def2-TZVPP-SMD(THF)//UB3LYP/def2-SVP-SMD(THF)

HF=-439.2731777

M062X/def2-TZVPP-SMD(THF)// UB3LYP/def2-SVP-SMD(THF)

HF=-439.0763983

DLPNO-CCSD(T)/def2-TZVPP-SMD(THF)// UB3LYP/def2-SVP-SMD(THF)

HF=-438.324406697736

E

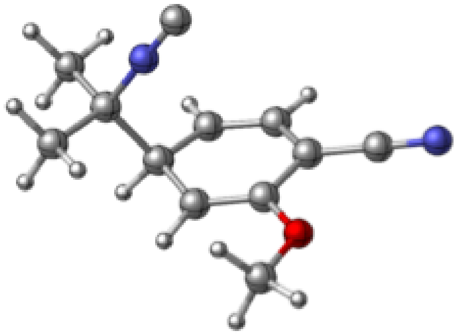

Zero-point correction= 0.222454 (Hartree/Particle)

Thermal correction to Energy= 0.237420

Thermal correction to Enthalpy= 0.238364

Thermal correction to Gibbs Free Energy= 0.180742

Sum of electronic and zero-point Energies= -649.179106

Sum of electronic and thermal Energies= -649.164140

Sum of electronic and thermal Enthalpies= -649.163196

Sum of electronic and thermal Free Energies= -649.220818

C	-1.59064100	0.21141200	1.53772400
C	-0.31391700	0.30883700	2.02093500
C	-0.47311700	2.75156100	2.00919800
C	-1.75134200	2.69800500	1.54439300
H	-1.98362200	-0.75888800	1.23571200
H	0.01474100	3.72759000	2.11137000
H	-2.25581900	3.62348200	1.25347700
O	0.51455000	-0.76314400	2.20444100
C	0.01914100	-2.06209700	1.98473100

H	-0.24858300	-2.23489300	0.92517900
H	0.82429000	-2.75988600	2.25948700
H	-0.86881200	-2.27896900	2.60800000
C	1.60862800	1.67215200	2.82424800
N	2.70415000	1.77855000	3.23923200
C	0.30108800	1.58165200	2.33104700
C	-2.51972400	1.40044800	1.43542600
H	-3.06287400	1.36721600	0.46653100
C	-3.73890400	1.31881000	2.46996100
N	-3.20429100	1.30973800	3.80639500
C	-2.74449400	1.29826600	4.88180600
C	-4.54181400	0.02424100	2.27207000
H	-5.39803100	-0.01325400	2.96367300
H	-4.92522400	-0.01468700	1.24045200
H	-3.91738000	-0.86273300	2.44920000
C	-4.66455200	2.53724000	2.33489800
H	-5.03444600	2.60322500	1.29968200
H	-5.53043500	2.44585800	3.00922000
H	-4.13507900	3.46883700	2.57865400

UB3LYP-D3/def2-TZVPP-SMD(THF)//UB3LYP/def2-SVP-SMD(THF)

HF=-650.1342696

M062X/def2-TZVPP-SMD(THF)// UB3LYP/def2-SVP-SMD(THF)

HF=-649.8416103

DLPNO-CCSD(T)/def2-TZVPP-SMD(THF)// UB3LYP/def2-SVP-SMD(THF)

HF=-648.736307596732

E-*o*

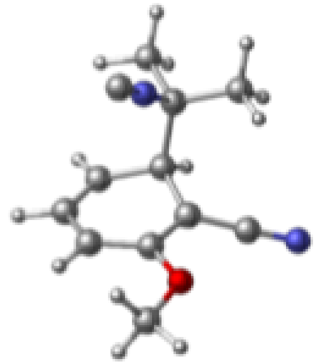

Zero-point correction= 0.222113 (Hartree/Particle)

Thermal correction to Energy= 0.237190

Thermal correction to Enthalpy= 0.238134

Thermal correction to Gibbs Free Energy= 0.180034

Sum of electronic and zero-point Energies= -649.177433

Sum of electronic and thermal Energies= -649.162357

Sum of electronic and thermal Enthalpies= -649.161412

Sum of electronic and thermal Free Energies= -649.219512

C	-2.05991100	-1.27139800	1.85553800
C	-0.71438100	-1.34111900	1.50559300
C	-0.14072200	-0.45546500	0.54926200
C	-2.38057400	0.57326400	0.25258000
C	-2.88045200	-0.34760000	1.12348200
H	-2.50533700	-1.94047000	2.58952100

H	-3.05840100	1.18894900	-0.34559700
H	-3.96870900	-0.42610600	1.24051400
O	0.14147100	-2.28720300	1.99201400
C	-0.36085600	-3.31329000	2.81779600
H	-1.15794400	-3.89649600	2.32044100
H	0.48362900	-3.98331100	3.03804700
H	-0.75714200	-2.92229000	3.77311800
C	1.11140200	-0.74007400	-0.01082200
N	2.15590400	-0.93633200	-0.51636600
C	-0.88969200	0.80355800	0.12114200
H	-0.63962600	1.03587000	-0.93157900
C	-0.42083300	2.13753100	0.86704300
C	-1.20681800	3.35401500	0.35281100
H	-2.28348300	3.24269200	0.54363500
H	-0.86187700	4.27709600	0.84441900
H	-1.05072400	3.45840200	-0.73228900
C	1.08684700	2.37491500	0.69997800
H	1.67135300	1.55495400	1.13794400
H	1.33258500	2.44193000	-0.37070400
H	1.38592300	3.31592000	1.18834200
N	-0.69117700	2.00273200	2.27664700
C	-0.91075300	1.88469300	3.41891400

E-*m*-1

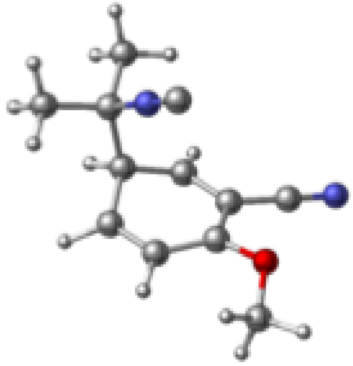

Zero-point correction= 0.220525 (Hartree/Particle)

Thermal correction to Energy= 0.235787

Thermal correction to Enthalpy= 0.236731

Thermal correction to Gibbs Free Energy= 0.178583

Sum of electronic and zero-point Energies= -649.146590

Sum of electronic and thermal Energies= -649.131328

Sum of electronic and thermal Enthalpies= -649.130384

Sum of electronic and thermal Free Energies= -649.188533

C	-1.77771700	-1.22340700	1.27876000
C	-0.49325000	-1.11338000	1.84938900
C	0.38354900	-0.17060700	1.25276400
C	-0.06146800	0.75889600	0.30155400
C	-2.23405400	-0.31532700	0.33289900
H	-2.39850600	-2.09356000	1.51582700
H	0.67115700	1.31654900	-0.28685900
H	-3.13953200	-0.54391100	-0.23876900

O	0.00071800	-1.88747600	2.89098400
C	-0.55304300	-3.16362900	3.06377600
H	-0.53368500	-3.76224800	2.12995300
H	0.04893700	-3.68281900	3.82656500
H	-1.60143300	-3.12993400	3.42335700
C	1.78231800	-0.24502500	1.55007500
N	2.92651900	-0.28209000	1.76690900
C	-1.53350100	1.00166900	0.10649100
H	-1.71656000	1.35550100	-0.92710300
C	-2.13242500	2.23322700	0.95575600
C	-3.62114400	2.41708100	0.64026600
H	-4.18054500	1.49674800	0.86735700
H	-4.05442700	3.24371200	1.22548500
H	-3.74426700	2.64463100	-0.43042500
C	-1.34068000	3.51245500	0.66527000
H	-0.27848400	3.37480800	0.91869900
H	-1.41417400	3.75392400	-0.40698300
H	-1.73200700	4.36448300	1.24349100
N	-2.01378500	1.94898100	2.36302400
C	-1.94269200	1.73195600	3.51011000

E-*m*-2

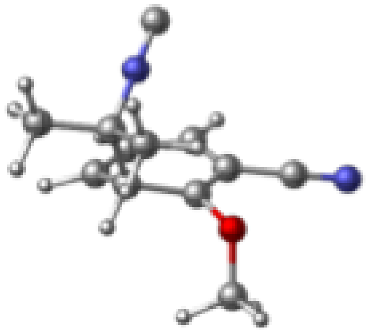

Zero-point correction= 0.220461 (Hartree/Particle)

Thermal correction to Energy= 0.235963

Thermal correction to Enthalpy= 0.236907

Thermal correction to Gibbs Free Energy= 0.177496

Sum of electronic and zero-point Energies= -649.154004

Sum of electronic and thermal Energies= -649.138502

Sum of electronic and thermal Enthalpies= -649.137558

Sum of electronic and thermal Free Energies= -649.196969

C	0.17398800	-0.68098900	0.76392600
C	0.78999200	0.55985300	0.87934100
C	0.25318600	1.74205400	0.27796100
C	-0.83254300	1.53457400	-0.59684100
C	-1.46129900	0.31020900	-0.74419900
H	0.73811000	2.70984000	0.40608800
H	-1.15347700	2.36394300	-1.24085100
H	-2.19241800	0.16871000	-1.54653800
O	0.82505400	-1.82386300	1.21160700
C	1.55675700	-2.52326700	0.21862800
H	2.37271100	-1.90460500	-0.20034200

H	1.99634200	-3.41536500	0.69327400
H	0.91023300	-2.85401900	-0.61730200
C	2.05744900	0.62296400	1.54538800
N	3.08863300	0.70329200	2.08054500
C	-1.21599400	-0.83874400	0.20807100
H	-1.27632400	-1.80999000	-0.32699700
C	-2.34730200	-1.03062500	1.33122400
C	-3.72665600	-1.18096400	0.67711300
H	-3.72458200	-2.06377000	0.01837400
H	-4.51094800	-1.31521500	1.43879900
H	-3.97385700	-0.29518300	0.07400400
C	-2.04169300	-2.23560500	2.23206300
H	-1.07156900	-2.11964000	2.73273900
H	-2.82798500	-2.36269200	2.99372800
H	-2.00030800	-3.14830800	1.61757100
N	-2.37709100	0.14609300	2.16081700
C	-2.40606100	1.10000300	2.83654200

TS3

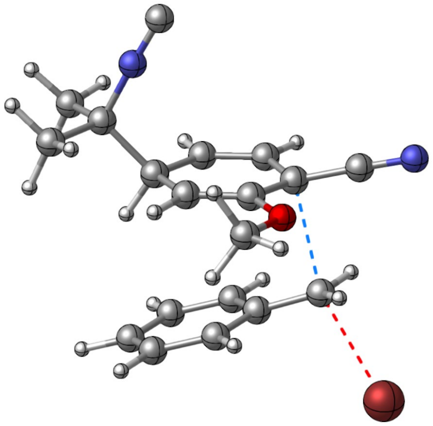

Zero-point correction= 0.342114 (Hartree/Particle)

Thermal correction to Energy= 0.365641

Thermal correction to Enthalpy= 0.366586

Thermal correction to Gibbs Free Energy= 0.287098

Sum of electronic and zero-point Energies= -3493.723782

Sum of electronic and thermal Energies= -3493.700254

Sum of electronic and thermal Enthalpies= -3493.699310

Sum of electronic and thermal Free Energies= -3493.778798

C	-1.54753100	0.13435800	1.67132900
C	-0.23674900	0.07870000	2.02301200
C	-0.15079000	2.54005800	2.16774300
C	-1.45038900	2.62978600	1.80500000
H	-2.07365900	-0.78489200	1.42250500
H	0.41985300	3.45138800	2.36952500
H	-1.90315100	3.61763600	1.70151000
O	0.49132400	-1.06733000	2.12526900
C	-0.15883600	-2.31097800	1.96475600

H	-0.56909200	-2.43450000	0.94627700
H	0.60049800	-3.08709300	2.13628800
H	-0.97837400	-2.44081700	2.69491000
C	1.80526600	1.20436700	2.88676600
N	2.85663100	1.17923800	3.39715200
C	0.55516900	1.28000700	2.22670200
C	1.71870900	1.24007500	-0.01672200
H	2.24466300	2.13215000	0.30641400
H	2.09880500	0.27655600	0.30484900
C	0.51805100	1.33414200	-0.79292600
C	-0.18505700	0.16885400	-1.18371200
C	-0.03919600	2.59157900	-1.12824600
C	-1.38360500	0.25741900	-1.88691100
H	0.23480900	-0.80803000	-0.93311900
C	-1.23973900	2.67791100	-1.82947100
H	0.49125500	3.50064100	-0.83556300
C	-1.92329700	1.51223200	-2.20483100
H	-1.91088400	-0.65455800	-2.18028000
H	-1.65467900	3.65850900	-2.07846700
H	-2.86648700	1.58139300	-2.75362900
C	-2.31112900	1.42618300	1.51746100
H	-2.67848700	1.49781200	0.46915700
C	-3.66440200	1.43491600	2.34573100

N	-3.33756300	1.34538300	3.74587000
C	-3.02939000	1.26762000	4.87177900
C	-4.54708500	0.22888600	1.98217400
H	-5.50963000	0.28636500	2.51280400
H	-4.74332900	0.23318500	0.89902600
H	-4.06719300	-0.72180300	2.25104700
C	-4.45575700	2.73380700	2.11737400
H	-4.64176600	2.86351500	1.04020500
H	-5.42516900	2.68649900	2.63638900
H	-3.91349900	3.61301700	2.49042400
Br	3.61698400	1.07300600	-1.93192600

UB3LYP-D3/def2-TZVPP-SMD(THF)//UB3LYP/def2-SVP-SMD(THF)

HF=-3495.4114843

M062X/def2-TZVPP-SMD(THF)// UB3LYP/def2-SVP-SMD(THF)

HF=-3494.9764885

DLPNO-CCSD(T)/def2-TZVPP-SMD(THF)// UB3LYP/def2-SVP-SMD(THF)

HF=-3491.88993048494

P

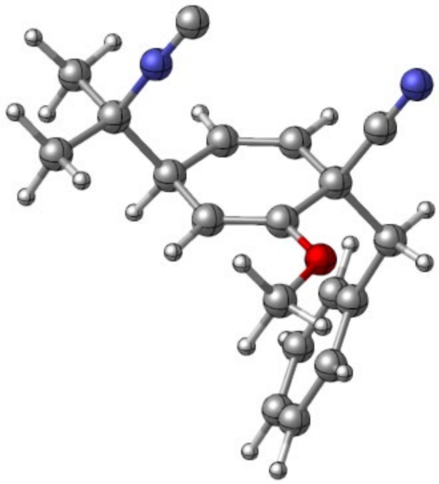

Zero-point correction= 0.345595 (Hartree/Particle)

Thermal correction to Energy= 0.366619

Thermal correction to Enthalpy= 0.367563

Thermal correction to Gibbs Free Energy= 0.295091

Sum of electronic and zero-point Energies= -919.747030

Sum of electronic and thermal Energies= -919.726006

Sum of electronic and thermal Enthalpies= -919.725061

Sum of electronic and thermal Free Energies= -919.797533

C	-1.94170200	0.18544900	1.63178400
C	-0.60165700	0.12576300	1.68367200
C	-0.51383300	2.63619200	1.76636700
C	-1.84691300	2.67891200	1.73352500
H	-2.51043300	-0.73695300	1.52497100
H	0.07235700	3.55921600	1.77331000
H	-2.33600000	3.65526700	1.70484600

O	0.15009700	-0.99872300	1.63671300
C	-0.49984000	-2.25750500	1.57398300
H	-1.09458100	-2.35858600	0.64912200
H	0.29223700	-3.01838100	1.57858800
H	-1.15906300	-2.41246100	2.44618100
C	1.10582900	1.29417800	2.97983100
N	1.75610600	1.27269900	3.93870700
C	0.29520700	1.35425800	1.74426300
C	1.29950900	1.36209800	0.52408900
H	1.91929200	2.26676500	0.61637400
H	1.95986200	0.49077200	0.62982600
C	0.60093400	1.33225000	-0.81365300
C	0.27896900	0.10846600	-1.42481700
C	0.22520200	2.52206300	-1.45822300
C	-0.39991700	0.07444300	-2.64625300
H	0.56433900	-0.82416500	-0.93240000
C	-0.45425400	2.49175200	-2.68018800
H	0.47165800	3.48256200	-0.99831600
C	-0.77072900	1.26690400	-3.27745100
H	-0.63864100	-0.88750800	-3.10818600
H	-0.73485900	3.42870000	-3.16910300
H	-1.30061800	1.24176600	-4.23350300
C	-2.73033400	1.46290300	1.72954500

H	-3.40667300	1.53312200	0.85695500
C	-3.72221500	1.45359000	2.96330500
N	-2.93111700	1.35903700	4.16358000
C	-2.25942800	1.27857200	5.11850900
C	-4.66755500	0.24114500	2.91011700
H	-5.37924400	0.28322000	3.74800900
H	-5.23534600	0.25952200	1.96767300
H	-4.12042700	-0.70866000	2.97724500
C	-4.55413600	2.74589800	3.03125200
H	-5.10196100	2.88055900	2.08643800
H	-5.28409800	2.67924900	3.85163700
H	-3.92690300	3.62983600	3.20724400

UB3LYP-D3/def2-TZVPP-SMD(THF)//UB3LYP/def2-SVP-SMD(THF)

HF=-921.1104721

M062X/def2-TZVPP-SMD(THF)// UB3LYP/def2-SVP-SMD(THF)

HF=-920.6861203

DLPNO-CCSD(T)/def2-TZVPP-SMD(THF)// UB3LYP/def2-SVP-SMD(THF)

HF=-919.103549357476

MeLi

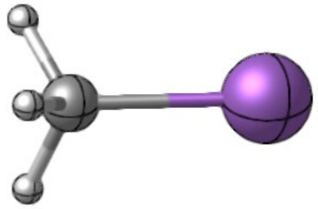

Zero-point correction= 0.032130 (Hartree/Particle)

Thermal correction to Energy= 0.035909

Thermal correction to Enthalpy= 0.036854

Thermal correction to Gibbs Free Energy= 0.009682

Sum of electronic and zero-point Energies= -47.357353

Sum of electronic and thermal Energies= -47.353574

Sum of electronic and thermal Enthalpies= -47.352630

Sum of electronic and thermal Free Energies= -47.379802

C	-6.16909400	1.03710300	0.04198200
H	-5.84087400	-0.02418500	-0.03366800
H	-5.84258300	1.49682900	-0.91801100
H	-7.27857000	0.98931200	-0.03740000
Li	-5.48879200	1.99325100	1.70643900

UB3LYP-D3/def2-TZVPP-SMD(THF)//UB3LYP/def2-SVP-SMD(THF)

HF=-47.4460154

M062X/def2-TZVPP-SMD(THF)// UB3LYP/def2-SVP-SMD(THF)

HF=-47.4079896

DLPNO-CCSD(T)/def2-TZVPP-SMD(THF)// UB3LYP/def2-SVP-SMD(THF)

HF=-47.328052358089

HMTETA

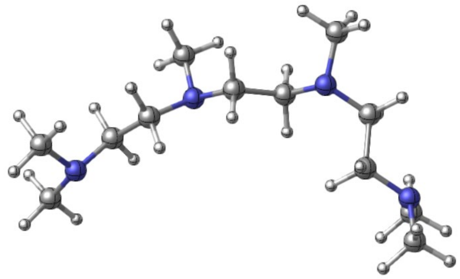

Zero-point correction= 0.424169 (Hartree/Particle)

Thermal correction to Energy= 0.445017

Thermal correction to Enthalpy= 0.445961

Thermal correction to Gibbs Free Energy= 0.371548

Sum of electronic and zero-point Energies= -693.410155

Sum of electronic and thermal Energies= -693.389307

Sum of electronic and thermal Enthalpies= -693.388363

Sum of electronic and thermal Free Energies= -693.462776

N	-4.72082000	1.33824900	0.18731200
C	-4.30657000	2.67587700	0.57559800
H	-3.63490300	3.11507900	-0.17700700
H	-5.18468100	3.33670000	0.66142200
H	-3.77199800	2.69595800	1.55476900
C	-5.65953300	0.78005000	1.14390800
H	-6.52587900	1.45167600	1.26223100
H	-6.03420300	-0.19476500	0.79218000
H	-5.21663000	0.62659700	2.15633800

C	-3.59483400	0.44580400	-0.06886300
H	-3.96318300	-0.59220400	-0.08235100
H	-2.84311500	0.49641600	0.75183000
C	-2.92481300	0.70248200	-1.42297200
H	-2.60318100	1.76333100	-1.51171900
H	-3.68876400	0.54881400	-2.20140700
N	-1.81400600	-0.21270300	-1.66846100
C	-0.58802100	0.15922100	-0.96379000
H	0.02604100	0.85430300	-1.57675900
H	-0.84240200	0.70803500	-0.04504300
C	0.23274100	-1.06564200	-0.54513100
H	0.43551800	-1.71538700	-1.42577700
H	-0.39599500	-1.65998500	0.13557400
N	1.46634500	-0.70241100	0.14827900
C	1.90814300	-1.73514800	1.08131900
H	2.26260400	-2.64067200	0.53751800
H	1.04360400	-2.04583300	1.68936800
C	2.98208400	-1.23976300	2.05701200
H	3.96028100	-1.11422300	1.53791800
H	2.67120800	-0.23703900	2.39386500
N	3.12276000	-2.09344200	3.23265100
C	3.88986100	-1.43988300	4.27785000
H	3.92035800	-2.07272500	5.17991100

H	4.94465500	-1.22815200	3.98193900
H	3.42286900	-0.48034800	4.55310300
C	3.66288500	-3.40660400	2.92338700
H	4.68706000	-3.36223500	2.48228000
H	3.72165200	-4.01488300	3.84043300
H	3.01720900	-3.94460100	2.21334300
C	2.51616300	-0.30045100	-0.77587300
H	3.36603400	0.15002400	-0.24163100
H	2.90674400	-1.15440600	-1.37759100
H	2.14070900	0.45677400	-1.47963200
C	-1.58633400	-0.44041700	-3.08520700
H	-1.32015400	0.49000500	-3.63900100
H	-0.76411000	-1.15724100	-3.23413900
H	-2.48633200	-0.86720900	-3.55670500

UB3LYP-D3/def2-TZVPP-SMD(THF)//UB3LYP/def2-SVP-SMD(THF)

HF=-694.6156581

M062X/def2-TZVPP-SMD(THF)// UB3LYP/def2-SVP-SMD(THF)

HF=-694.2315527

DLPNO-CCSD(T)/def2-TZVPP-SMD(THF)// UB3LYP/def2-SVP-SMD(THF)

HF=-693.079591639579

LiHMTETA

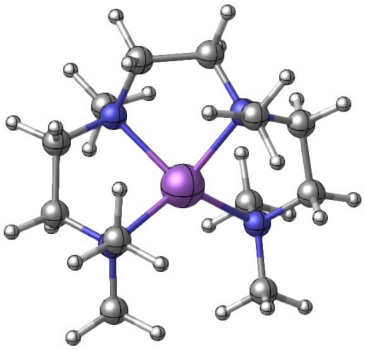

Zero-point correction= 0.432302 (Hartree/Particle)

Thermal correction to Energy= 0.452053

Thermal correction to Enthalpy= 0.452998

Thermal correction to Gibbs Free Energy= 0.386955

Sum of electronic and zero-point Energies= -700.934502

Sum of electronic and thermal Energies= -700.914751

Sum of electronic and thermal Enthalpies= -700.913807

Sum of electronic and thermal Free Energies= -700.979849

N	-4.15018000	-2.10191600	-2.00179400
C	-4.84638200	-3.03338300	-2.89357100
H	-4.24762400	-3.21233700	-3.79919600
H	-5.00195800	-3.99680000	-2.38765100
H	-5.83714800	-2.64459500	-3.20691700
C	-4.91283800	-1.92634200	-0.76058300
H	-5.07656300	-2.90585500	-0.28581000
H	-4.36605200	-1.29620800	-0.04432500
H	-5.90334300	-1.46067900	-0.94240600

C	-3.90007400	-0.82223400	-2.69432000
H	-4.84786700	-0.27149600	-2.86860100
H	-3.48653100	-1.06024500	-3.68732000
C	-2.92575600	0.08117400	-1.94015600
H	-3.37489600	0.39639400	-0.98778900
H	-2.76767100	1.00671200	-2.52751100
N	-1.65739100	-0.60927000	-1.65439900
C	-1.04931800	-0.24052000	-0.36613500
H	-0.41948100	0.66826700	-0.44995700
H	-1.85992600	0.00527000	0.33527400
C	-0.20853800	-1.38159600	0.21052600
H	0.20327700	-1.06960300	1.19147300
H	0.65453300	-1.56467200	-0.44568700
N	-0.98020100	-2.63119000	0.31108600
C	-0.16893200	-3.85327300	0.18378200
H	0.16301600	-4.23208700	1.17017100
H	0.74621200	-3.60917500	-0.37428800
C	-0.93219100	-4.96237700	-0.53893000
H	-0.28013100	-5.85617300	-0.62754400
H	-1.80066000	-5.26483900	0.06750400
N	-1.44069600	-4.54289700	-1.85975500
C	-2.41047200	-5.51839900	-2.36607900
H	-2.81592900	-5.18242700	-3.33114600

H	-1.95392500	-6.51887400	-2.51274300
H	-3.24653100	-5.62406100	-1.65840200
C	-0.36199400	-4.34792400	-2.83513300
H	0.21164800	-5.28095500	-3.01239900
H	-0.78503600	-4.01804100	-3.79619600
H	0.34189200	-3.57462500	-2.49596200
C	-1.84311400	-2.66457700	1.49274800
H	-2.45948100	-3.57621300	1.49264300
H	-1.25799300	-2.64552300	2.43513300
H	-2.52858100	-1.80450200	1.49897100
C	-0.71493700	-0.51095300	-2.76999600
H	-0.36767900	0.53065400	-2.92918700
H	0.16812800	-1.14221600	-2.59250100
H	-1.18622900	-0.85932000	-3.70115800
Li	-2.20674600	-2.64013000	-1.41418800

UB3LYP-D3/def2-TZVPP-SMD(THF)//UB3LYP/def2-SVP-SMD(THF)

HF=-702.1388095

M062X/def2-TZVPP-SMD(THF)// UB3LYP/def2-SVP-SMD(THF)

HF=-701.7490357

DLPNO-CCSD(T)/def2-TZVPP-SMD(THF)// UB3LYP/def2-SVP-SMD(THF)

HF=-700.577801576037

R·

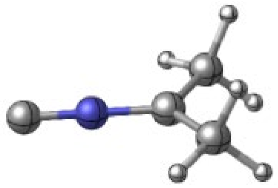

Zero-point correction= 0.086883 (Hartree/Particle)

Thermal correction to Energy= 0.093604

Thermal correction to Enthalpy= 0.094548

Thermal correction to Gibbs Free Energy= 0.055016

Sum of electronic and zero-point Energies= -210.473777

Sum of electronic and thermal Energies= -210.467056

Sum of electronic and thermal Enthalpies= -210.466112

Sum of electronic and thermal Free Energies= -210.505644

C	-4.24344100	2.19634600	-0.06284200
C	-3.51235200	3.49968700	-0.05946400
H	-2.74282700	3.51170000	-0.85043700
H	-4.19265200	4.35044700	-0.20672000
H	-2.98092100	3.64380700	0.90144700
C	-3.50998400	0.89432600	-0.06328300
H	-4.18909400	0.04253800	-0.21012200
H	-2.74244100	0.88483600	-0.85623800
H	-2.97586000	0.74983000	0.89605200
C	-6.76590100	2.19385100	0.14090000
N	-5.58166900	2.19498300	0.05041800

UB3LYP-D3/def2-TZVPP-SMD(THF)//UB3LYP/def2-SVP-SMD(THF)

HF=-210.7992528

M062X/def2-TZVPP-SMD(THF)// UB3LYP/def2-SVP-SMD(THF)

HF=-210.6867903

DLPNO-CCSD(T)/def2-TZVPP-SMD(THF)// UB3LYP/def2-SVP-SMD(THF)

HF=-210.328800209165

B

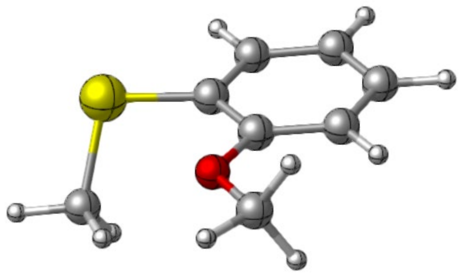

Zero-point correction= 0.161555 (Hartree/Particle)

Thermal correction to Energy= 0.171655

Thermal correction to Enthalpy= 0.172599

Thermal correction to Gibbs Free Energy= 0.125776

Sum of electronic and zero-point Energies= -783.746607

Sum of electronic and thermal Energies= -783.736506

Sum of electronic and thermal Enthalpies= -783.735562

Sum of electronic and thermal Free Energies= -783.782386

C	-3.23781900	2.01469500	1.79719900
C	-1.87550400	1.82789000	1.51052400
C	-0.96651800	2.87812900	1.68325100
C	-1.39987100	4.12654300	2.13152000

C	-2.75998000	4.32324100	2.39580300
C	-3.68969500	3.28770500	2.23548800
H	-1.51309800	0.86044100	1.16246300
H	0.09049700	2.70887900	1.46066000
H	-0.69139100	4.94742400	2.26577600
H	-3.11852800	5.30003400	2.73014900
O	-4.17004000	1.04255000	1.67785500
C	-3.78299000	-0.25381700	1.25413000
H	-3.05498500	-0.71073800	1.94741500
H	-4.69779300	-0.86215300	1.24714200
H	-3.35386700	-0.24157200	0.23658900
S	-5.39635700	3.57828800	2.68141200
C	-6.26174400	3.24487100	1.10465300
H	-7.32675200	3.45856900	1.28350600
H	-5.89075800	3.90774900	0.30820900
H	-6.14346700	2.19404300	0.80777000

UB3LYP-D3/def2-TZVPP-SMD(THF)//UB3LYP/def2-SVP-SMD(THF)

HF=-784.4793592

M062X/def2-TZVPP-SMD(THF)// UB3LYP/def2-SVP-SMD(THF)

HF=-784.2574067

DLPNO-CCSD(T)/def2-TZVPP-SMD(THF)// UB3LYP/def2-SVP-SMD(THF)

HF=-783.140587612

D’

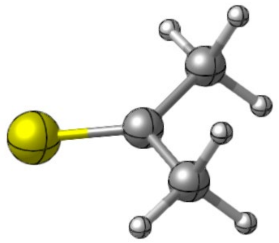

Zero-point correction= 0.081027 (Hartree/Particle)

Thermal correction to Energy= 0.086493

Thermal correction to Enthalpy= 0.087437

Thermal correction to Gibbs Free Energy= 0.052163

Sum of electronic and zero-point Energies= -515.846346

Sum of electronic and thermal Energies= -515.840881

Sum of electronic and thermal Enthalpies= -515.839937

Sum of electronic and thermal Free Energies= -515.875210

C	-3.66745000	-1.33271700	-0.09786600
H	-3.96362400	-1.96104900	-0.95743900
H	-4.06350300	-1.83816900	0.80161400
H	-4.13205600	-0.34281200	-0.19341100
C	-1.47494000	-2.58187100	0.11153400
H	-1.84098500	-3.10864700	1.01148000
H	-1.73715400	-3.22546200	-0.74777500
H	-0.38364500	-2.47837800	0.16883500
S	-1.35387500	0.16685000	-0.06824300

C -2.16941700 -1.25629500 -0.01795000

UB3LYP-D3/def2-TZVPP-SMD(THF)//UB3LYP/def2-SVP-SMD(THF)

HF=-516.2019604

M062X/def2-TZVPP-SMD(THF)// UB3LYP/def2-SVP-SMD(THF)

HF=-516.0921106

DLPNO-CCSD(T)/def2-TZVPP-SMD(THF)// UB3LYP/def2-SVP-SMD(THF)

HF=-515.430899008202

BnBr

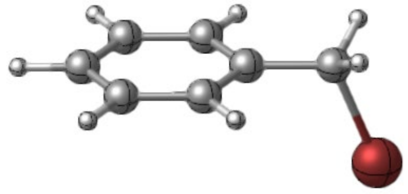

Zero-point correction= 0.119027 (Hartree/Particle)

Thermal correction to Energy= 0.126078

Thermal correction to Enthalpy= 0.127022

Thermal correction to Gibbs Free Energy= 0.085420

Sum of electronic and zero-point Energies= -2844.543907

Sum of electronic and thermal Energies= -2844.536856

Sum of electronic and thermal Enthalpies= -2844.535912

Sum of electronic and thermal Free Energies= -2844.577514

C	-2.17741500	-0.91617200	-0.00065400
C	-0.78040500	-0.91542200	0.00914600
C	-0.06761200	0.29539400	0.01884000
C	-0.78113500	1.50581200	0.01290000
C	-2.17813800	1.50574200	0.00304700
C	-2.87972000	0.29458000	-0.00436800
H	-2.72095400	-1.86473000	-0.00417000
H	-0.23340100	-1.86261700	0.00873500
H	-0.23473800	2.45335100	0.01542500
H	-2.72225000	2.45397800	0.00239900
H	-3.97298600	0.29426700	-0.01158200

C	1.42602200	0.29579800	0.02174000
H	1.84912000	1.19240700	0.49040700
H	1.84958300	-0.60265000	0.48645700
Br	2.17653600	0.30021300	-1.84243800

UB3LYP-D3/def2-TZVPP-SMD(THF)//UB3LYP/def2-SVP-SMD(THF)

HF=-2845.2743962

M062X/def2-TZVPP-SMD(THF)// UB3LYP/def2-SVP-SMD(THF)

HF=-2845.1462129

DLPNO-CCSD(T)/def2-TZVPP-SMD(THF)// UB3LYP/def2-SVP-SMD(THF)

HF=-2843.16335744874

LiBr·HMTEDA

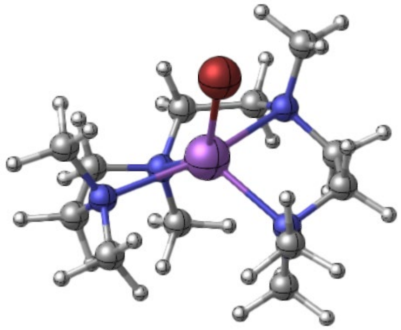

Zero-point correction= 0.431476 (Hartree/Particle)

Thermal correction to Energy= 0.453855

Thermal correction to Enthalpy= 0.454799

Thermal correction to Gibbs Free Energy= 0.380615

Sum of electronic and zero-point Energies= -3274.983196

Sum of electronic and thermal Energies= -3274.960818

Sum of electronic and thermal Enthalpies= -3274.959873

Sum of electronic and thermal Free Energies= -3275.034057

N	-4.37445000	-1.93543200	-2.23955400
C	-4.72007500	-2.92279200	-3.25647700
H	-3.83276600	-3.18737300	-3.85190600
H	-5.09442600	-3.83639600	-2.77118900
H	-5.50476300	-2.55813200	-3.95571600
C	-5.54557400	-1.63472000	-1.41616600
H	-5.88654400	-2.54670900	-0.90956900
H	-5.29118600	-0.91226400	-0.62749700
H	-6.37361800	-1.20835000	-2.02480900

C	-3.81132500	-0.71616000	-2.82782200
H	-4.60877600	-0.03901400	-3.20221200
H	-3.21260700	-0.99735100	-3.70719800
C	-2.93262100	0.04201800	-1.83532600
H	-3.51358000	0.25451200	-0.92528200
H	-2.65514800	1.02763800	-2.26610800
N	-1.74265300	-0.73010300	-1.45594800
C	-1.19025300	-0.31057100	-0.16264300
H	-0.75872300	0.71375100	-0.21646100
H	-2.01900800	-0.27198500	0.56315600
C	-0.12647600	-1.27638100	0.36108100
H	0.24815300	-0.88602500	1.32591000
H	0.74386900	-1.27753900	-0.31192800
N	-0.64953400	-2.64099000	0.50401900
C	0.15903500	-3.68870500	-0.12526700
H	0.91555600	-4.09727000	0.57869600
H	0.72686100	-3.24449400	-0.95537900
C	-0.67582400	-4.85757100	-0.64935000
H	0.01195000	-5.67665100	-0.95449600
H	-1.30257300	-5.24769900	0.16661800
N	-1.57949600	-4.50475600	-1.75953200
C	-2.52544400	-5.60104300	-1.99273000
H	-3.17998200	-5.35660400	-2.84158100

H	-2.00059000	-6.55118400	-2.23174900
H	-3.15649100	-5.74175200	-1.10377500
C	-0.84953500	-4.22021400	-2.99481000
H	-0.29872100	-5.11085800	-3.36752900
H	-1.55219000	-3.90242000	-3.78038700
H	-0.12331900	-3.41119200	-2.85230600
C	-0.96785100	-2.95032900	1.89406400
H	-1.47780000	-3.91886800	1.96794000
H	-0.05626400	-2.96178100	2.53235200
H	-1.67134000	-2.20687500	2.29555200
C	-0.73570100	-0.67674000	-2.51454100
H	-0.35056100	0.35474400	-2.66725400
H	0.11918900	-1.32384800	-2.28202800
H	-1.15522500	-1.02666000	-3.46726500
Li	-2.64250200	-2.79691600	-0.86937400
Br	-4.13260200	-3.93413600	0.82851900

UB3LYP-D3/def2-TZVPP-SMD(THF)//UB3LYP/def2-SVP-SMD(THF)

HF=-3276.5052067

M062X/def2-TZVPP-SMD(THF)// UB3LYP/def2-SVP-SMD(THF)

HF=-3276.1266934

DLPNO-CCSD(T)/def2-TZVPP-SMD(THF)// UB3LYP/def2-SVP-SMD(THF)

HF=-3273.46056309574

D-R$_{complex}$

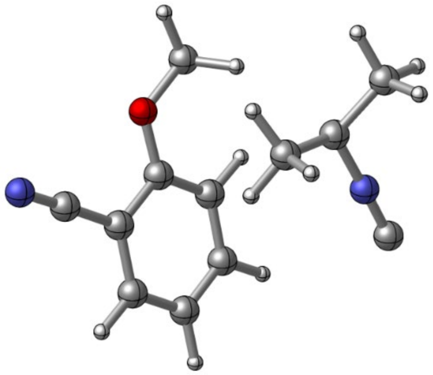

Zero-point correction= 0.220097 (Hartree/Particle)

Thermal correction to Energy= 0.236247

Thermal correction to Enthalpy= 0.237191

Thermal correction to Gibbs Free Energy= 0.175149

Sum of electronic and zero-point Energies= -649.16077

Sum of electronic and thermal Energies= -649.14462

Sum of electronic and thermal Enthalpies= -649.14367

Sum of electronic and thermal Free Energies= -649.20572

C	-1.72569600	0.46783200	0.68821400
C	-1.09618200	0.99317500	1.82757500
C	-1.07694700	3.19958800	0.75351400
C	-1.71566900	2.66839200	-0.36230300
H	-2.00913100	-0.58212400	0.65105300
H	-0.81695000	4.25937600	0.79709700
H	-1.96957100	3.31141100	-1.20754800
O	-0.78066300	0.28490200	2.92360500
C	-1.17587900	-1.08221400	3.01046300

H	-0.64482600	-1.69930300	2.26455700
H	-0.89387700	-1.41694300	4.01780500
H	-2.26813900	-1.18093200	2.87113200
C	-0.11787800	2.92322600	3.00386200
N	0.40896700	3.39568400	3.92657100
C	-0.76197800	2.37365200	1.85202700
C	-2.03479300	1.30330100	-0.38671700
H	-2.54808300	0.87829600	-1.25221400
C	-4.72647100	-0.07974100	2.49817800
N	-5.18569700	0.22700500	1.23895700
C	-5.53683200	0.48465500	0.13541300
C	-4.31876400	1.15090700	3.28355000
H	-5.18426400	1.77350100	3.63127900
H	-3.75089500	0.87435100	4.19028000
H	-3.67074900	1.81794100	2.69009400
C	-5.65045200	-1.01805800	3.24883700
H	-5.18037200	-1.36614800	4.18624800
H	-6.62707200	-0.55150100	3.54373700
H	-5.89985800	-1.91776300	2.65816600

UB3LYP-D3/def2-TZVPP-SMD(THF)//UB3LYP/def2-SVP-SMD(THF)

HF= -650.11538

M062X/def2-TZVPP-SMD(THF)// UB3LYP/def2-SVP-SMD(THF)

HF= -649.80868

DLPNO-CCSD(T)/def2-TZVPP-SMD(THF)// UB3LYP/def2-SVP-SMD(THF)

HF= -648.7010279

TS$_{D-E}$

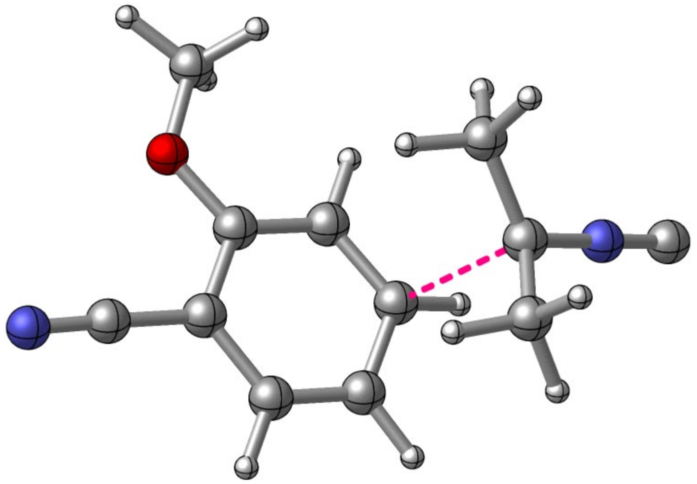

Zero-point correction=	0.219075	(Hartree/Particle)
Thermal correction to Energy=	0.234703	
Thermal correction to Enthalpy=	0.235648	

Thermal correction to Gibbs Free Energy= 0.175231

Sum of electronic and zero-point Energies= -649.15994

Sum of electronic and thermal Energies= -649.14431

Sum of electronic and thermal Enthalpies= -649.14337

Sum of electronic and thermal Free Energies= -649.20378

C	-1.82315200	0.25758200	1.53257300
C	-0.54898200	0.30836300	2.08502500
C	-0.72088300	2.75089200	2.20238700
C	-1.99051100	2.69475600	1.66518400
H	-2.25557100	-0.69754000	1.23670500
H	-0.26356200	3.71590100	2.43714100
H	-2.54179700	3.61871300	1.47584900
O	0.23949800	-0.77365400	2.29947200
C	-0.25000000	-2.06235600	1.98163800
H	-0.45741700	-2.16853700	0.90151500
H	0.53933100	-2.77376000	2.26266000
H	-1.16826100	-2.30379500	2.54625900
C	1.33458000	1.62966800	2.99361400
N	2.40698700	1.71001400	3.45074300
C	0.03060300	1.56665200	2.44430200
C	-2.61024900	1.43759700	1.40367800
H	-3.50223500	1.39973400	0.77634600

C	-4.32500100	1.24954000	3.15301300
N	-5.42062500	1.17221700	2.34622600
C	-6.37375300	1.10494800	1.64436900
C	-4.17251600	2.52802000	3.92592800
H	-4.78858500	2.56184300	4.85327400
H	-3.12056800	2.66795600	4.23279900
H	-4.45166600	3.40361700	3.31694700
C	-3.92288900	-0.02566900	3.83520600
H	-2.86091200	0.01865000	4.13626600
H	-4.51303600	-0.24396800	4.75399800
H	-4.03167600	-0.89379700	3.16423700

UB3LYP-D3/def2-TZVPP-SMD(THF)//UB3LYP/def2-SVP-SMD(THF)

HF= -650.11219

M062X/def2-TZVPP-SMD(THF)// UB3LYP/def2-SVP-SMD(THF)

HF= -649.80322

DLPNO-CCSD(T)/def2-TZVPP-SMD(THF)// UB3LYP/def2-SVP-SMD(THF)

HF= -648.6930035

A’

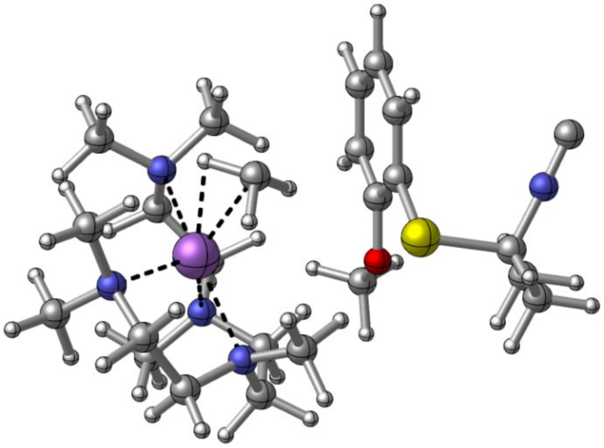

Zero-point correction= 0.680965 (Hartree/Particle)

Thermal correction to Energy= 0.720817

Thermal correction to Enthalpy= 0.721761

Thermal correction to Gibbs Free Energy= 0.609626

Sum of electronic and zero-point Energies= -1695.249356

Sum of electronic and thermal Energies= -1695.209503

Sum of electronic and thermal Enthalpies= -1695.208559

Sum of electronic and thermal Free Energies= -1695.320695

N	-8.38489300	0.87844900	0.94558800
C	-8.67490600	-0.55817000	0.94173200
H	-8.67006700	-0.94845100	-0.08479800
H	-7.90696400	-1.09790100	1.51512400
H	-9.66318000	-0.78075600	1.39786300
C	-8.34866000	1.36466400	2.32560800
H	-7.54439600	0.86002600	2.88194200
H	-8.15411100	2.44308800	2.35904900

H	-9.30549800	1.17401400	2.85763200
C	-9.38034800	1.60476800	0.13709000
H	-10.35907200	1.64483400	0.66237600
H	-9.54594800	1.02956100	-0.78608400
C	-8.95945200	3.02977200	-0.22283400
H	-8.76996600	3.59483700	0.70068100
H	-9.81950300	3.52952900	-0.71750600
N	-7.75376500	3.07912100	-1.05855500
C	-6.92571000	4.28194100	-0.87135200
H	-6.94480100	4.92112600	-1.77300300
H	-7.34647100	4.90015200	-0.06391800
C	-5.46625500	3.94274400	-0.56809000
H	-4.89588100	4.88973500	-0.43993000
H	-5.02711100	3.43162000	-1.43744000
N	-5.31734500	3.06742600	0.60209000
C	-3.94459100	2.56123600	0.73316100
H	-3.27500900	3.33200200	1.17051800
H	-3.55989300	2.35181200	-0.27283100
C	-3.86623900	1.29060000	1.57522300
H	-2.79737100	1.05785700	1.77493500
H	-4.32851500	1.46772900	2.55810500
N	-4.54821100	0.16407500	0.93587200
C	-4.94736100	-0.85306600	1.90010900

H	-5.49654100	-1.65809500	1.38841400
H	-4.07845400	-1.31347800	2.42129900
H	-5.60861300	-0.41847800	2.66544100
C	-3.72112900	-0.42386700	-0.11650700
H	-2.77790500	-0.84904700	0.29140600
H	-4.26856000	-1.22173400	-0.63489600
H	-3.45526700	0.32935300	-0.86796600
C	-5.73354100	3.73712900	1.83338700
H	-5.65388500	3.05660600	2.69157300
H	-5.11023300	4.63261300	2.04588700
H	-6.77904600	4.06297500	1.77636900
C	-8.05415400	2.85159700	-2.46990400
H	-8.64286000	3.68683900	-2.90778100
H	-7.12386600	2.74087400	-3.04341600
H	-8.62756500	1.92474600	-2.60361300
Li	-6.51732300	1.26769900	-0.15133000
C	-2.17414800	2.33568100	-2.52735500
C	-1.05320800	1.70779500	-1.96053800
C	-0.78597500	0.36296100	-2.23873100
C	-1.62237400	-0.37567600	-3.07713600
C	-2.73689500	0.24587600	-3.64769600
C	-3.02160700	1.59367000	-3.39090000
H	-0.38924800	2.25951400	-1.29525500

H	0.08859000	-0.10870300	-1.78268000
H	-1.41690500	-1.42801200	-3.28423000
H	-3.41117600	-0.31637400	-4.29631100
O	-2.51138500	3.62248700	-2.29027800
C	-1.66753800	4.43911500	-1.49634600
H	-0.66107900	4.53353800	-1.94032200
H	-2.13951700	5.43039300	-1.46552500
H	-1.57442100	4.05602500	-0.46619200
C	-3.81364000	3.11010900	-5.68026900
C	-2.76884300	4.19268700	-5.40088500
H	-1.89327900	3.77430300	-4.88699200
H	-2.43755900	4.63348700	-6.35524900
H	-3.20948900	4.97732200	-4.77217300
C	-5.03969500	3.66638700	-6.41418500
H	-5.52027500	4.44153400	-5.79936300
H	-4.72238700	4.11911000	-7.36622100
H	-5.76887700	2.87089500	-6.62702100
N	-3.21231400	2.07894900	-6.46847700
C	-2.69022300	1.22563000	-7.07813100
S	-4.48159900	2.32328600	-4.10933500
C	-6.58055900	-0.32396600	-1.87702700
H	-5.76592600	0.37469700	-2.15505200
H	-7.54165400	0.21700400	-1.90122100

H -6.40845600 -0.69776200 -0.85323800

UB3LYP-D3/def2-TZVPP-SMD(THF)//UB3LYP/def2-SVP-SMD(THF)

HF= -1697.506396

M062X/def2-TZVPP-SMD(THF)// UB3LYP/def2-SVP-SMD(THF)

HF= -1696.789159

DLPNO-CCSD(T)/def2-TZVPP-SMD(THF)// UB3LYP/def2-SVP-SMD(THF)

HF= -1694.1596496

TS0

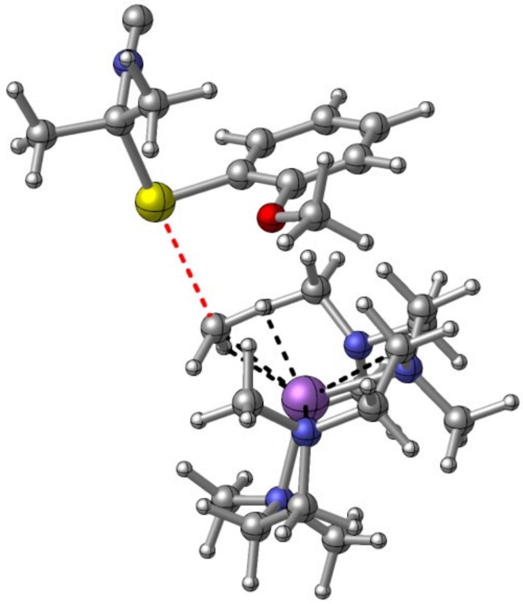

Zero-point correction= 0.680224 (Hartree/Particle)

Thermal correction to Energy= 0.719192

Thermal correction to Enthalpy= 0.720136

Thermal correction to Gibbs Free Energy= 0.610687

Sum of electronic and zero-point Energies= -1695.245615

Sum of electronic and thermal Energies= -1695.206647

Sum of electronic and thermal Enthalpies= -1695.205703

Sum of electronic and thermal Free Energies= -1695.315152

N	-8.33205300	0.89121100	0.87560100
C	-8.56132000	-0.47002800	0.37941500
H	-8.21977500	-0.55663400	-0.66392300
H	-8.00262500	-1.19380400	0.98734900
H	-9.63424100	-0.74938600	0.41974400
C	-8.60463100	0.96270400	2.31584600

H	-7.94536300	0.26722000	2.85705200
H	-8.41300800	1.97489200	2.70078700
H	-9.65575200	0.69867800	2.55367500
C	-9.15528200	1.85183800	0.11362900
H	-10.21567500	1.80366000	0.43586900
H	-9.13703000	1.54368000	-0.94230800
C	-8.65694500	3.28961400	0.22977900
H	-8.69459100	3.60662000	1.28241700
H	-9.35434500	3.95336900	-0.32184200
N	-7.27497600	3.42903400	-0.25140800
C	-6.54169100	4.55770600	0.35026600
H	-6.47875100	5.41554900	-0.34475100
H	-7.09108700	4.92147500	1.23051000
C	-5.12786700	4.14385900	0.75102700
H	-4.58414900	5.01781200	1.16753100
H	-4.58923300	3.82601100	-0.15352600
N	-5.13385700	3.00859900	1.68425100
C	-3.82619000	2.34094600	1.77940400
H	-3.16527500	2.83790800	2.51778100
H	-3.32172900	2.42531900	0.80547000
C	-3.98225500	0.86991100	2.15114700
H	-2.97888100	0.40936100	2.26057300
H	-4.47088000	0.78796600	3.13430800

N	-4.79933300	0.12660500	1.17351700
C	-5.27100300	-1.12885100	1.76276800
H	-5.84810600	-1.69770900	1.02024800
H	-4.43029600	-1.76950700	2.10129500
H	-5.91889700	-0.92692300	2.62925600
C	-4.03438400	-0.15647400	-0.04731800
H	-3.12782900	-0.75827100	0.16641900
H	-4.65637000	-0.71603100	-0.75722000
H	-3.71562000	0.76990600	-0.54102500
C	-5.65225600	3.36684700	3.00567600
H	-5.67398500	2.48386000	3.65998500
H	-5.03169700	4.14479700	3.49683000
H	-6.68149300	3.74513200	2.93737800
C	-7.20633100	3.47469200	-1.71567200
H	-7.69474500	4.38777700	-2.11646200
H	-6.16450100	3.45717200	-2.06154000
H	-7.70987900	2.60527200	-2.15631200
Li	-6.35288500	1.60797300	0.67912300
C	-3.20340900	2.49707200	-2.72337000
C	-1.94897500	2.45365100	-2.09887500
C	-1.20931900	1.25954300	-2.06910500
C	-1.71216400	0.10970700	-2.67287600
C	-2.96207700	0.15214200	-3.30521000

C	-3.72738500	1.32820500	-3.33848300
H	-1.53741000	3.34902200	-1.63247000
H	-0.23441400	1.24614600	-1.57426700
H	-1.13587000	-0.81893400	-2.66859200
H	-3.35836200	-0.74059300	-3.79161500
O	-3.95957800	3.62564400	-2.77187400
C	-3.37659500	4.87271200	-2.44472100
H	-2.48247700	5.07429900	-3.05861200
H	-4.13865100	5.63560000	-2.65815500
H	-3.09860300	4.94333000	-1.37852400
C	-3.36053300	2.44398500	-6.14552300
C	-2.45308600	3.58872400	-5.77992400
H	-1.75643700	3.31533900	-4.96794100
H	-1.82212100	3.96455300	-6.62571600
H	-3.04556500	4.44948500	-5.42540200
C	-4.49374800	2.75012600	-7.09169000
H	-5.10646400	3.58625200	-6.70902100
H	-4.16606000	3.04441400	-8.12154200
H	-5.16762400	1.88336200	-7.20855600
N	-2.72625600	1.26425600	-6.37935000
C	-2.19446500	0.21081000	-6.52305600
S	-5.33471900	1.31432200	-4.11881700
C	-6.27773100	0.31321700	-2.88035400

H	-6.28515700	0.78832100	-1.88896300
H	-7.31276500	0.25284300	-3.25259500
H	-5.87776600	-0.70876400	-2.79378300

UB3LYP-D3/def2-TZVPP-SMD(THF)//UB3LYP/def2-SVP-SMD(THF)

HF= -1697.506396

M062X/def2-TZVPP-SMD(THF)// UB3LYP/def2-SVP-SMD(THF)

HF= -1696.789159

DLPNO-CCSD(T)/def2-TZVPP-SMD(THF)// UB3LYP/def2-SVP-SMD(THF)

HF= -1694.145247

A^{-}

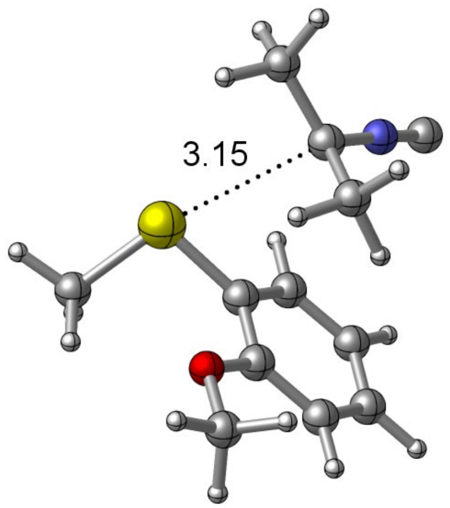

Zero-point correction=	0.248687	(Hartree/Particle)
Thermal correction to Energy=	0.266596	
Thermal correction to Enthalpy=	0.26754	
Thermal correction to Gibbs Free Energy=	0.202352	

Sum of electronic and zero-point Energies= -994.315226

Sum of electronic and thermal Energies= -994.297318

Sum of electronic and thermal Enthalpies= -994.296373

Sum of electronic and thermal Free Energies= -994.361561

C	-3.78320100	1.71288100	1.52788800
C	-2.44598300	1.56107300	1.92317400
C	-1.71078900	2.66909700	2.36904100
C	-2.29945700	3.93338900	2.41737400
C	-3.62911800	4.08827600	2.00990500
C	-4.39010900	2.99578600	1.57678400
H	-1.96903500	0.58091500	1.89375200
H	-0.67193800	2.52997000	2.68106900
H	-1.73599200	4.79804400	2.77616900
H	-4.10487100	5.06962200	2.05584700
O	-4.55700700	0.68878900	1.08541000
C	-4.05032900	-0.62934600	1.12252100
H	-3.76997300	-0.93236100	2.14762300
H	-4.85914700	-1.28451300	0.76881600
H	-3.17521200	-0.75867700	0.45945900
C	-6.04410300	3.00648300	4.26815700
C	-7.53181600	3.21907700	4.34385600
H	-7.80590400	4.26514600	4.11833400

H	-8.05490300	2.58437400	3.60450600
H	-7.98746700	2.97893700	5.34078800
C	-5.54231400	1.60005300	4.45685500
H	-4.45033400	1.53971700	4.30969100
H	-5.75331200	1.16226800	5.46806600
H	-6.00011300	0.91706400	3.71864100
N	-5.27519800	3.97899400	4.81691900
C	-4.56687500	4.83692100	5.24332500
S	-6.10448800	3.21571800	1.12800900
C	-5.96527600	2.98126700	-0.69667300
H	-6.97889800	3.09348000	-1.11433600
H	-5.30524900	3.73551500	-1.15462100
H	-5.59273800	1.97164900	-0.92744300

UB3LYP-D3/def2-TZVPP-SMD(THF)//UB3LYP/def2-SVP-SMD(THF)

HF= -995.376117

M062X/def2-TZVPP-SMD(THF)// UB3LYP/def2-SVP-SMD(THF)

HF= -995.043042

DLPNO-CCSD(T)/def2-TZVPP-SMD(THF)// UB3LYP/def2-SVP-SMD(THF)

HF= -993.563487

A0

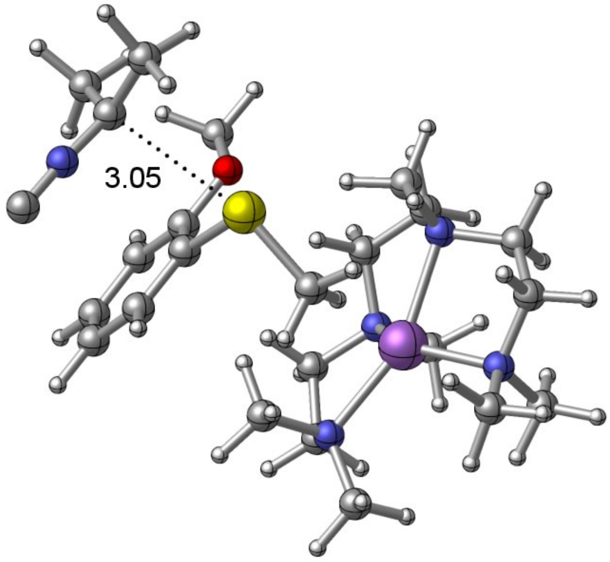

Zero-point correction= 0.683133 (Hartree/Particle)

Thermal correction to Energy= 0.722262

Thermal correction to Enthalpy= 0.723206

Thermal correction to Gibbs Free Energy= 0.613451

Sum of electronic and zero-point Energies= -1695.270289

Sum of electronic and thermal Energies= -1695.231161

Sum of electronic and thermal Enthalpies= -1695.230217

Sum of electronic and thermal Free Energies= -1695.339971

N	-8.33205300	0.89121100	0.87560100
C	-8.56132000	-0.47002800	0.37941500
H	-8.21977500	-0.55663400	-0.66392300
H	-8.00262500	-1.19380400	0.98734900
H	-9.63424100	-0.74938600	0.41974400

C	-8.60463100	0.96270400	2.31584600
H	-7.94536300	0.26722000	2.85705200
H	-8.41300800	1.97489200	2.70078700
H	-9.65575200	0.69867800	2.55367500
C	-9.15528200	1.85183800	0.11362900
H	-10.21567500	1.80366000	0.43586900
H	-9.13703000	1.54368000	-0.94230800
C	-8.65694500	3.28961400	0.22977900
H	-8.69459100	3.60662000	1.28241700
H	-9.35434500	3.95336900	-0.32184200
N	-7.27497600	3.42903400	-0.25140800
C	-6.54169100	4.55770600	0.35026600
H	-6.47875100	5.41554900	-0.34475100
H	-7.09108700	4.92147500	1.23051000
C	-5.12786700	4.14385900	0.75102700
H	-4.58414900	5.01781200	1.16753100
H	-4.58923300	3.82601100	-0.15352600
N	-5.13385700	3.00859900	1.68425100
C	-3.82619000	2.34094600	1.77940400
H	-3.16527500	2.83790800	2.51778100
H	-3.32172900	2.42531900	0.80547000
C	-3.98225500	0.86991100	2.15114700
H	-2.97888100	0.40936100	2.26057300

H	-4.47088000	0.78796600	3.13430800
N	-4.79933300	0.12660500	1.17351700
C	-5.27100300	-1.12885100	1.76276800
H	-5.84810600	-1.69770900	1.02024800
H	-4.43029600	-1.76950700	2.10129500
H	-5.91889700	-0.92692300	2.62925600
C	-4.03438400	-0.15647400	-0.04731800
H	-3.12782900	-0.75827100	0.16641900
H	-4.65637000	-0.71603100	-0.75722000
H	-3.71562000	0.76990600	-0.54102500
C	-5.65225600	3.36684700	3.00567600
H	-5.67398500	2.48386000	3.65998500
H	-5.03169700	4.14479700	3.49683000
H	-6.68149300	3.74513200	2.93737800
C	-7.20633100	3.47469200	-1.71567200
H	-7.69474500	4.38777700	-2.11646200
H	-6.16450100	3.45717200	-2.06154000
H	-7.70987900	2.60527200	-2.15631200
Li	-6.35288500	1.60797300	0.67912300
C	-3.20340900	2.49707200	-2.72337000
C	-1.94897500	2.45365100	-2.09887500
C	-1.20931900	1.25954300	-2.06910500
C	-1.71216400	0.10970700	-2.67287600

C	-2.96207700	0.15214200	-3.30521000
C	-3.72738500	1.32820500	-3.33848300
H	-1.53741000	3.34902200	-1.63247000
H	-0.23441400	1.24614600	-1.57426700
H	-1.13587000	-0.81893400	-2.66859200
H	-3.35836200	-0.74059300	-3.79161500
O	-3.95957800	3.62564400	-2.77187400
C	-3.37659500	4.87271200	-2.44472100
H	-2.48247700	5.07429900	-3.05861200
H	-4.13865100	5.63560000	-2.65815500
H	-3.09860300	4.94333000	-1.37852400
C	-3.36053300	2.44398500	-6.14552300
C	-2.45308600	3.58872400	-5.77992400
H	-1.75643700	3.31533900	-4.96794100
H	-1.82212100	3.96455300	-6.62571600
H	-3.04556500	4.44948500	-5.42540200
C	-4.49374800	2.75012600	-7.09169000
H	-5.10646400	3.58625200	-6.70902100
H	-4.16606000	3.04441400	-8.12154200
H	-5.16762400	1.88336200	-7.20855600
N	-2.72625600	1.26425600	-6.37935000
C	-2.19446500	0.21081000	-6.52305600
S	-5.33471900	1.31432200	-4.11881700

C	-6.27773100	0.31321700	-2.88035400
H	-6.28515700	0.78832100	-1.88896300
H	-7.31276500	0.25284300	-3.25259500
H	-5.87776600	-0.70876400	-2.79378300

UB3LYP-D3/def2-TZVPP-SMD(THF)//UB3LYP/def2-SVP-SMD(THF)

HF= -1697.53301

M062X/def2-TZVPP-SMD(THF)// UB3LYP/def2-SVP-SMD(THF)

HF= -1696.805705

DLPNO-CCSD(T)/def2-TZVPP-SMD(THF)// UB3LYP/def2-SVP-SMD(THF)

HF= -1694.15827
